# Characterizing subgenome recombination and chromosomal imbalances in banana varietal lineages

**DOI:** 10.1093/aob/mcad192

**Published:** 2023-12-14

**Authors:** Janet Higgins, Jaime Andrés Osorio-Guarín, Carolina Olave-Achury, Deisy Lisseth Toloza-Moreno, Ayda Enriquez, Federica Di Palma, Roxana Yockteng, Jose J De Vega

**Affiliations:** Earlham Institute, Norwich Research Park, Norwich NR4 7UZ, UK; Centro de Investigación Tibaitatá, Corporación Colombiana de Investigación Agropecuaria, AGROSAVIA, km 14 vía Mosquera, Bogotá, Colombia; Earlham Institute, Norwich Research Park, Norwich NR4 7UZ, UK; Centro de Investigación Tibaitatá, Corporación Colombiana de Investigación Agropecuaria, AGROSAVIA, km 14 vía Mosquera, Bogotá, Colombia; Centro de Investigación Palmira, Corporación Colombiana de Investigación Agropecuaria, AGROSAVIA, Palmira, Colombia; Earlham Institute, Norwich Research Park, Norwich NR4 7UZ, UK; Centro de Investigación Tibaitatá, Corporación Colombiana de Investigación Agropecuaria, AGROSAVIA, km 14 vía Mosquera, Bogotá, Colombia; Muséum National d’Histoire Naturelle, UMR-CNRS 7205, Paris, France; Earlham Institute, Norwich Research Park, Norwich NR4 7UZ, UK

**Keywords:** *Musa*, recombination, introgressions, tools, chromosomal imbalances, homoeologous, banana, plantains

## Abstract

**Background:**

Bananas and plantains (*Musa* spp.) are among the most important crops worldwide. The cultivated varieties are vegetatively propagated, so their genetic diversity is essentially fixed over time. *Musa acuminata*, *M. balbisiana* and *M. schizocarpa* have provided the named A, B and S subgenomes that predominantly constitute these varieties. Here we aimed to characterize intergenetic recombination and chromosomal imbalances between these A/B/S subgenomes, which often result in copy-number variants (CNVs) leading to changes in gene dosage and phenotype, in a diverse panel of bananas and plantains. This will allow us to characterize varietal lineages better and identify sources of genetic variation.

**Methods:**

We delimited population structure and clonal lineages in a diverse panel of 188 banana and plantain accessions from the most common cultivars using admixture, principal component and phylogenetic analyses. We used new scalable alignment-based methods, Relative Averaged Alignment (RAA) and Relative Coverage, to infer subgenome composition (AA, AAB, etc.) and interspecific recombination.

**Results:**

In our panel, we identified ten varietal lineages composed of somatic clones, plus three groups of tetraploid accessions. We identified chromosomal exchanges resulting in gains/losses in chromosomal segments (CNVs), particularly in AAB and ABB varieties.

**Conclusions:**

We demonstrated alignment-based RAA and Relative Coverage can identify subgenome composition and introgressions with similar results to more complex approaches based on single nucleotide polymorphism (SNP) databases. These *ab initio* species-agnostic methods can be used without sequencing a panel of wild ancestors to find private SNPs, or in recently diverged pools where private SNPs are uncommon. The extensive A/B/S exchanges and the variation in the length of some introgressions between lineages further support multiple foundational events of hybridization and residual backcrossing. Imbalances between A/B/S may have resulted in CNVs and gene dosage variation. Since most edible banana genomes are fixed on time, these CNVs are stable genetic variations probably associated with phenotypic variation for future genetic studies.

## INTRODUCTION

Bananas, part of the genus *Musa*, are large herbaceous plants grown in tropical and subtropical regions of Southeast Asia, Africa and America belonging to the family Musaceae. Bananas are one of the most important crops cultivated worldwide, with annual production exceeding 124 million tonnes in 2021 (FAO, 2021). However, despite hundreds of banana cultivars worldwide, only a few are grown commercially for large-scale production, with the main commercial banana being triploid somatic clones from the Cavendish subgroup.

The domestication and selection of seedless fruit have resulted in the fixation of parthenocarpy and sterility in cultivated bananas. Therefore, the crop is primarily vegetatively propagated, and the diversity of cultivated bananas is essentially fixed over time. The introduction of genetic diversity is limited to the somatic accumulation of mutations, somaclonal variation introduced via tissue culture, breeding crosses between sexual (usually diploid) accessions or, in recent years, genome editing. Nevertheless, millennia of diversification of wild genotypes and human selection of hybrids have led to the current existence of hundreds of edible banana and plantain varieties (Heslop-Harrison and Schwarzacher, 2007).

The origin of cultivated bananas is believed to involve up to 11 genetic pools, mainly through natural inter(sub)specific hybridization with variable levels of contribution from various subspecies of *Musa acuminata* Colla and *M. balbisiana* Jacq., which are the contributors of the named A and B subgenomes, respectively (Heslop-Harrison and Schwarzacher, 2007; D’Hont *et al.*, 2012; Martin *et al.*, 2023). In addition, Martin *et al.* (2023) recently reported all edible cultivars carried contributions from *M. schizocarpa* N.W.Simmonds (S subgenome).

Cultivated bananas have been classified into groups based on qualitative morphological descriptors and genome composition (AA, AB, AAA, AAB, ABB) (Simmonds and Shepherd, 1955). Among them, the most common and widespread are the allotriploids, such as the exported commercial Cavendish varieties (AAA) or the cooking plantains (AAB). Edible diploid bananas (AA, AB) are also cultivated, especially in subsistence farming systems.

Given their socioeconomic importance, over 6800 *Musa* accessions are currently managed in 30 collections (Ruas *et al.*, 2017; Houwe *et al.*, 2020), with a large collection maintained at the International Musa Germplasm Transit Centre (ITC), comprising more than 1600 accessions (Houwe *et al.* 2020). Its genetic diversity and population structure have been well-characterized using several genotyping methods (Perrier *et al.*, 2011; Florez *et al.*, 2012; Christelová *et al.*, 2017; Němečková *et al.*, 2018; Igwe *et al.*, 2021; Sardos *et al.*, 2022), flow-cytometric analysis to determine ploidy level (Christelová *et al.* 2017), chromosome painting to analyse karyotyping (Šimoníková *et al.* 2019, 2022) and sequencing-based analysis to characterize the inter(sub)specific hybridization patterns that gave rise to cultivated bananas (Baurens *et al.*, 2019; Martin *et al.*, 2020, 2023; Cenci *et al.*, 2021).

Recurrent chromatin exchanges between homoeologous chromosomes have been described, tentatively originating following backcrosses between hybrids with residual fertility and the parental donors (Baurens *et al.*, 2019; Martin *et al.*, 2020, 2023; Cenci *et al.*, 2021). Questions remain concerning the donors and intergenomic recombination among *Musa* spp., particularly following the growing evidence that most varietal clonal groups may be the product of complex multiple hybridization events (Baurens *et al.*, 2019; Cenci *et al.*, 2021). To clarify the origins of cultivated bananas, Martin *et al*. (2023) recently set up a diversity panel including 55 wild accessions and used them to identify single nucleotide polymorphisms (SNPs) exclusively present in each of the potential wild ancestral subspecies (private SNPs). They then used them as markers to identify ancestral genetic groups (i.e. the subspecies) contributing to cultivars. They built chromosome ancestry mosaics of wild diploids, cultivated diploids and cultivated polyploids, allowing them to catalogue segmental aneuploidy in the panel.

The objectives of the present study were to clarify the extent of, and diversity within, banana varietal clonal groups, and to identify lineage-specific intergenic recombination and chromosomal imbalances between the A/B/S homoeologous chromosomes. These objectives aim to bring new insights into the evolution of banana clonal lineages and clarify regions where genome composition deviates from that generally described for the varietal group. Since the edible banana genomes are clonal and fixed on time, intergenic recombination and chromosomal imbalances between the A/B/S subgenomes are a stable source of copy-number variants (CNVs). CNVs often affect gene dosage and are associated with phenotypic plasticity (Beckmann *et al.*, 2007; Christie *et al.*, 2016). This is well-studied in immune responses and the arms race between host and pathogen (Jia *et al.*, 2015; Baggs *et al.*, 2017). Consequently, these regions constitute a primary source in clonal crops of allelic diversity associated with phenotypic plasticity. These can be exploited to inform breeding crosses and provide candidate targets for genome editing.

## METHODS

### DNA extraction and sequencing

A total of 190 accessions from the *in situ* banana collection managed by AGROSAVIA in its research centre in Palmira, Colombia (3.51424, −76.3158), were used for this study (Supplementary Data Table S1). The passport information is available in *MGIS: Musa Germplasm Information System* (Ruas *et al.*, 2017) as ‘COL004’. We also identified clones at the ITC genebank for 80 of the accessions, including at least one accession from each clonal lineage, and most of the unclustered cultivars and wild accessions. Genomic DNA of the accessions was extracted from liquid nitrogen macerated young leaf material using the DNeasy Plant Mini Kit (Qiagen, Germany) and shipped to the Genomic Pipelines sequencing service at the Earlham Institute (Norwich, UK), where DNA libraries were constructed and assessed using a modified version of Illumina’s ‘Nextera DNA library Prep’ protocol, known as the ‘Low Input Transposase Enabled (LITE)’ protocol (Beier *et al.*, 2017; Perez-Sepulveda *et al.*, 2021), and sequenced in an Illumina Novaseq S4 lane (150-bp paired-end reads) aiming for ~7× average depth per accession.

### Read alignment and relative averaged alignment metric

Raw reads in FastQ format were pre-processed using Trim Galore v0.5 (Krueger, 2021) with the options for Illumina paired reads and to remove the Nextera adaptors, any bases with quality under 20, and a minimum read length of 80 bp. Processed reads were aligned using BWA MEM v0.7.17 (Li, 2013), with the options -M and -R to define read-groups, against the four genome references.

Several banana reference genome assemblies are available (Droc *et al.*, 2013). We used the high-quality chromosome-level assemblies of an *M. acuminata* doubled-haploid cv. ‘Pahang’ accession version 4 (Liu *et al.*, 2023), an *M. balbisiana* cv. ‘Pisang Klutuk Wulung’ accession (Wang *et al.*, 2019), and an *M. schizocarpa* wild accession (Belser *et al.*, 2018). These chromosome-level assemblies are respectively referred to as ‘A-genome reference’, ‘B-genome reference’ and ‘S-genome reference’ in this paper. The length of the reference for the A-genome, B-genome and S-genome references was 479.219, 457.198 and 525.284 Mb, respectively. In addition, in some analyses we used an unpublished Nanopore long-read assembly of accession ITC0643 from the Bluggoe subgroup (ABB) kindly made available to us (M. Rouard, pers. comm.).

Coverage and alignment statistics were obtained using Samtools flagstat v1.7 (Li *et al.*, 2009) for the complete genome and separately for each of the 11 chromosomes in each reference. The ‘relative averaged alignment’ (RAA) is a normalized percentage of properly paired reads in a sample and reference that accounts for variation in sample quality (PCR duplications, DNA quality, etc.) and differences in the genetic distance between varieties and the reference (reference bias). RAA was calculated by dividing the percentage of properly paired reads from a sample in a reference by a weight factor (in the range 0.95–1.05). The weight factor was obtained by averaging the ratios in each of the reference genomes between the properly paired reads in the sample and variety cluster (example in Supplementary Data Table S2). RAA per chromosome was similarly calculated except for each chromosome’s alignment statistics instead of the total genome. A step by step protocol and associated code to calculate and plot RAA is included in the authors’ Github repository (see Data Availability).

### SNP calling and population structure analysis

SNP calling was carried out against the A-genome and B-genome references. The alignment BAM files were sorted using Samtools v1.7, and duplicate reads were marked using Picard tools v1.128. SNP calling was done using GATK HaplotypeCaller v3.7.0 (McKenna *et al.*, 2010) using all the BAM files as input (multisample mode). The resulting VCF files were filtered for bi-allelic SNP calls with a minimal quality value of 100 and a read depth of >10 and <300 reads using BCFtools v1.9 (Danecek *et al.*, 2021). GATK SelectVariants v3.7.0 was then used to remove SNP sites with over 30 % missing samples, and, finally, SNP sites were filtered for minimum allele frequency (MAF) of 1 % using BCFtools.

The population structure of the diversity panel was estimated using STRUCTURE v2.3.4 (Pritchard *et al.*, 2000). SNP calls were transformed to diploid (0/0, 0/1 and 1/1). Heterozygous calls (0/1) were changed to missing (./.) if either allele was supported by fewer than two reads. The files were then thinned to one SNP site within 50 bp (--thin 50) using VCFtools v0.1.13 (Danecek *et al.*, 2011). The admixture model was used with a burn-in period length of 10 000 and 50 000 MCMC iterations. Twenty independent runs were performed for each *K* from 2 to 10. Ten replicated Q-matrices belonging to the largest cluster were aligned using the R package POPHELPER v2.2.7 (Francis, 2017), and then merged using CLUMPP v1.1.2 (Jakobsson and Rosenberg, 2007). Delta *K* (Δ*K*) was estimated using the Evanno method (Evanno *et al.*, 2005) within POPHELPER.

Principal component analysis (PCA) was carried out using Tassel v5.2.41 (Bradbury *et al.*, 2007). A neighbour-joining (NJ) phylogenetic tree was built with SNPrelate (Zheng *et al.* 2012) using identity-by-descent (IBS) and hierarchical clustering (functions snpgdsIBS and snpgdsHCluster), and plotted with iTOL v6 (Letunic and Bork, 2021).

### Relative Coverage to synthetic AB and AS references

Trimmed reads were aligned using BWA-MEM v0.7.17, using the options -M and -k 35, to the concatenated A- and B-genomes, or the concatenated A- and S-genome. BAM files were sorted and duplicated reads were removed. Only uniquely mapped reads were retained by excluding reads with the tags ‘XA:Z:’ and ‘SA:Z:’, and further filtered to retain only properly mapped paired reads (-f 0x2). BEDtools genomeCoverageBed and BEDtools map v1.7 (Quinlan and Hall, 2010) were used to obtain the median of the read coverage or read depth values in the positions within a given 100-kb window. Repetitive regions resulted in ‘peaks’ that made the median a better metric that the average. All 100-kb windows within the A-genome and B-genome were aligned to each other using minimap2 v2.22 (-x asm10) to identify homologous windows (Li, 2018). Because of the conserved sequences between the A- and B-genomes, a ‘background coverage’ was observed between genomes; that is, a low proportion of reads from accessions consistently aligned in regions that have not diverged between the A- and the B-reference. When it happens, read aligners randomly assign these reads to one of the equally conserved sequences, generating a background signal. However, conserved sequences between A/B/S are uncommon because of divergence between the original genome reference species. This background signal is much lower than the mapping coverage and easily distinguished during analysis.

The Relative Coverage between the A-genome and B-genome was plotted using R, and the library ggplot2 (Wickham, 2016). Two plots were produced in parallel to account only for the regions conserved and represent the differences in chromosome length between genomes A and B, one containing all the windows/regions in the A-genome and the conserved homologous windows in the B-genome below (B aligned to A), and another containing all the windows in the B-genome and the conserved windows in the A-genome below (A aligned to B). Coverage was normalized by dividing the window coverage by the chromosome average coverage to obtain values in the 0–2 range, named Relative Coverage. The A-genome is always plotted in blue and coverage in the B-genome is always plotted in red. This analysis was completed in individual samples and on each varietal clonal group by merging the BAM files from the group’s accessions with Samtools v1.7 (Li *et al.*, 2009). The same analysis was completed using the A-genome and S-genome combined. A step by step protocol and associated code to calculate and plot Relative Coverage is included in the authors’ Github repository (see Data Availability).

## RESULTS

### Genotyping of the diversity panel

The average coverage (read depth) per accession was 7.7×, 7.4× and 6.8×, against the A-, B- and S-genomes, respectively. Two samples failed during sequencing, reducing the panel to 190 accessions for analysis. The average percentage of properly aligned paired reads (at the right insert length and read orientations) was 81.8, 70.9, and 76.0 % against the A-, B,- and S-genomes, respectively. We also used a genome assembly from an ABB cultivar (587.01 Mb), where the coverage per accession was 6.0, and the average percentage of properly aligned paired reads was 80.1 % (Supplementary Data Table S3).

Single nucleotide variants (SNVs) were called against the A-genome and B-genome, as these are the main donor genomes in cultivated bananas, for population analysis. In total, 39 518 945 variants were obtained against the A-genome reference, of which 35 038 465 were SNPs. In total, 33 401 534 of these SNPs were biallelic. The equivalent metrics of the B-genome were 34 104 907 variants and 29 625 724 SNPs, of which 28 161 490 SNPs were biallelic. After filtering, we obtained 187 133 and 220 451 SNP loci against the A-genome and B-genome, respectively. These datasets were used for coverage, principal component and phylogenetic analyses. These datasets were further thinned by physical distance (50 bp) and allele frequency (1 %) into 35 246 and 42 745 SNP sites, respectively, for admixture analysis.

### Delimitation of varietal clonal clusters using admixture analysis

**Fig. 1.**
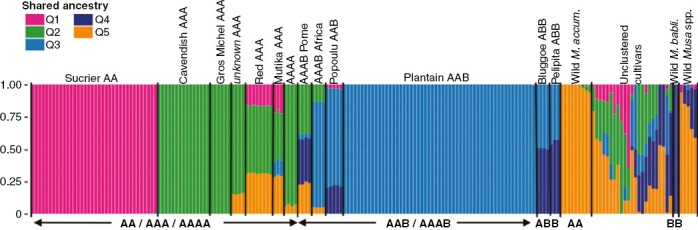
Admixture analysis of the genetic ancestry inferred in the complete set of 190 *Musa* accessions. Each accession is represented by a stacked column partitioned by the proportion of the ancestral genetic component, where each identified ancestral genetic component is represented by a different colour. Genetic composition was used to assign accessions to groups.

A set of 35 246 SNP loci called against the A-genome was used to place 151 accessions into 13 genetic clusters based on five genetically distinct ancestries (*K* = 5) using STRUCTURE analysis (Fig. 1). We ran the analysis for 2–10 distinct sources (*K* values) based on an estimation of recognized genetic groups. The Evanno Δ*K* method indicated that the most likely value of *K* was 5 (Supplementary Data Fig. S1), after which no further meaningful genetic clusters were detected (Fig. S2). The STRUCTURE analysis was repeated using a set of 42 745 SNP loci called against the B-genome. Again, the Evanno Δ*K* method indicated that the most likely value of *K* was 5 (Fig. S1), and again no further varietal groups were resolved until *K* = 6 (Fig. S3). The partitioning between the Cavendish and Gros Michel AAA clusters was only observed against the A-genome reference (Fig. 1), while the separation between the Bluggoe ABB and Pelipita ABB clusters was more clearly obtained against the B-genome (Fig. S4).

The 13 genetic clusters obtained were later labelled using the passport information registered in MGIS (Ruas *et al.*, 2017) for the accessions in the AGROSAVIA genebank (COL004), which enabled all the genetic clusters to be associated with a variety, except for one cluster that was labelled ‘unknown AAA’ (Table 1). Ten genetic clusters corresponded to clonal clusters (i.e. somatic clones from a lineage established in the same event). The remaining three genetic clusters were synthetic tetraploids generated in breeding crosses. We did not place 39 accessions into clusters, either single representatives of *M. acuminata* or *M. balbisiana* subspecies, or cultivars from varieties poorly represented in our panel, as we required at least three samples to establish a cluster. Among these 39, five were annotated as wild bananas from other *Musa* species in MGIS (Supplementary Data Table S1).

**Table 1.** Summary of the 13 genetic clusters identified*.*

Cluster name	Subgroup	Composition	Accessions
Sucrier AA	Sucrier	AA	36
Cavendish AAA	Cavendish	AAA	15
Gros Michel AAA	Gros Michel	AAA	6
Unknown AAA	–	AAA	4
Red AAA	Red	AAA	8
Mutika AAA	Mutika/Lujugira	AAA	3
AAAA	Breeding material	AAAA	4
AAAB Pome	Breeding material	AAAB	4
AAAB Africa	Breeding material	AAAB	4
Popoulu AAB	Maia Maoli/Popoulu	AAB	5
Plantain ABB	Plantain	AAB	55
Bluggoe ABB	Bluggoe	ABB	4
Pelipita ABB	Pelipita	ABB	3
Other wild *Musa* spp.			5
Wild *M. accuminata*		AA	10
Wild *M. balbisiana*		BB	2
Unclustered^*^			22

^*^See Supplementary Data Table S1 for further details.

The five identified ancestry sources were named Q1–Q5, corresponding to phylogenetically distinct ancestries (Fig. 1). The clusters ‘Cavendish AAA’, ‘GrosMichel AAA’ and ‘unknown AAA’ shared A-genome ancestry (Q2). The A-genome donor in ‘Sucrier AA’ was distinguishable from the previous source (Q1). Based on the ancestry and passport associated with the unclustered *M. accuminata* and *M. balbisiana* accessions, Q5 is a third A-genome ancestry contributing to the ‘Red AAA’, ‘unknown AAA’ and ‘Mutika AAA’ clusters but absent in the other AA/AAA groups. The B-genome ancestry named Q4 was the major component in the ABB clusters, namely ‘Bluggoe ABB’ and ‘Pelipita ABB’, but was fully absent in plantains (AAB), since the cluster ‘Plantain AAB’ evidenced a homogeneous and independent B-genome origin (Q3). This genetic cluster included the African-origin Plantain accessions (De Langhe, 2015) in our study. The cluster ‘Popoulu AAB’ (with the ‘pacific plantains’) was an admixture of both Q4 and Q3. Remarkably, the ‘Mutika AAA’ cluster evidenced a minor presence of Q3 ancestry, and it was generally highly admixed from four of the ancestries. The synthetic tetraploid clusters directly evidenced the genetic composition of their contemporary parental crosses reported in MGIS.

### Relation among the genetic clusters using principal component and phylogenetic analyses

**Fig. 2.**
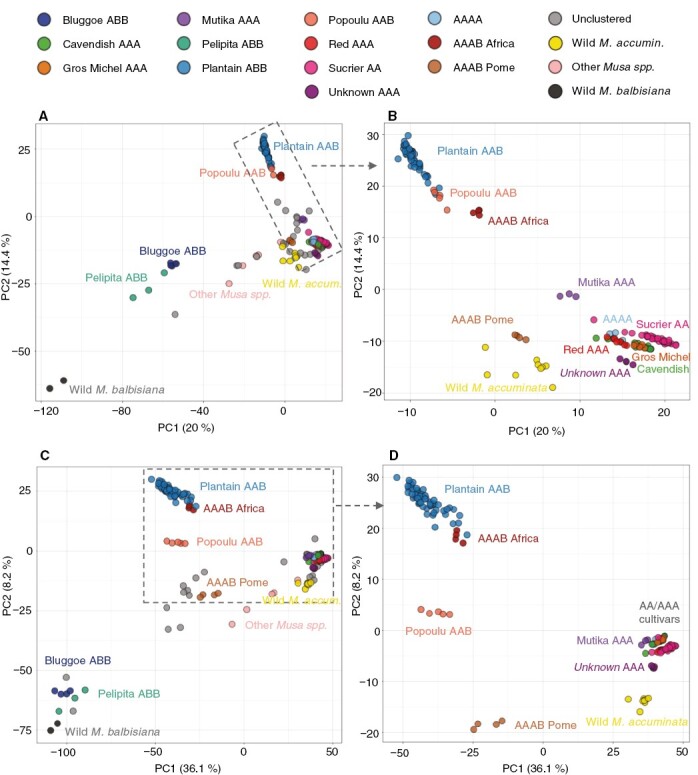
Population structure by principal component analysis (PCA) using the top two principal components to separate the 188 accessions, which were coloured by genetic clusters. (A, B) SNPs called against the A-genome. (C, D) SNPs called against the B-genome. (B, D) Expanded PCA for the 142 accessions in the 11 clusters excluding the ABB groups.

**Fig. 3.**
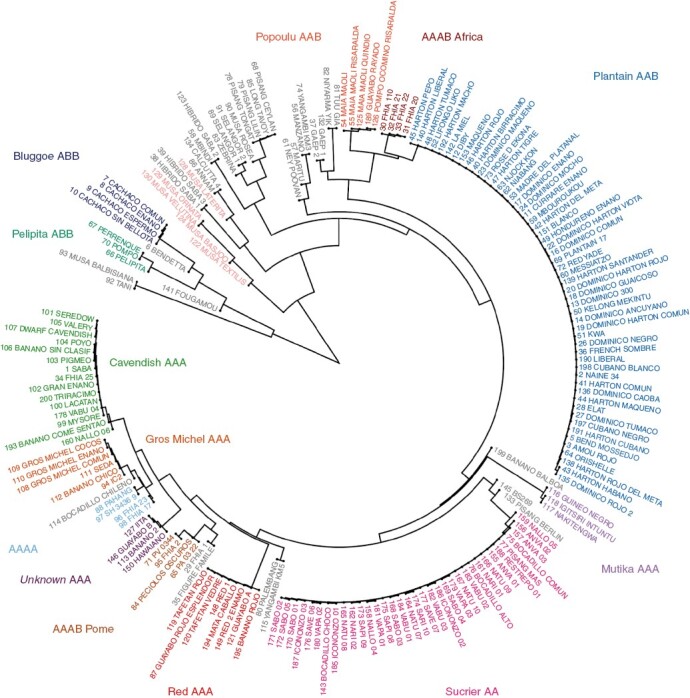
Phylogenetic tree using hierarchical clustering of the complete set of 190 accessions separated the accessions into similar divisions as the principal components and admixture analyses and revealed groups of somatic clones.

Both PCA (Fig. 2) and the phylogenetic tree (Fig. 3) showed that the genetic clusters were generally placed together based on the number of B genomes, namely AA/AAA/AAAA, then AAB clusters, and finally ABB clusters were more distant.

‘Cavendish AAA’, ‘Red AAA’, ‘Gros Michel AAA’, ‘Sucrier AA’ and the tetraploid ‘AAAA’ genetic clusters, with no B genome, were placed closely togther (Figs 2 and 3). Diploid AA cultivars and polyploid AA/AAA accessions could be more clearly separated in the phylogenetic tree. Tetraploids (AAAA/AAAB) clustered with their main contributor in the phylogenetic tree, so ‘Pome AAAB’ was close to AA/AAA. However, in the PCA, tetraploids were in between their contributors, so ‘Pome AAAB’ are halfway between the AA/AAA and BB clusters and separated from any other group. ‘Popoulu AAB’ and ‘Plantain AAB’ could only be separated when using the B-genome as reference (Fig. 2D). The cluster of synthetic tetraploids ‘AAAB Africa’ overlapped with its B-progenitors, the ‘Plantain AAB’ group (Fig. 2C, D). Two unclustered accessions, ‘FHIA 1’ (sample 29) and the Pome cultivar ‘Figue Famile’ (sample 35), were placed close to the ‘AAAB Pome’ cluster (Fig. 2C).

### Inferring subgenome composition based on alignment metrics

**Fig. 4.**
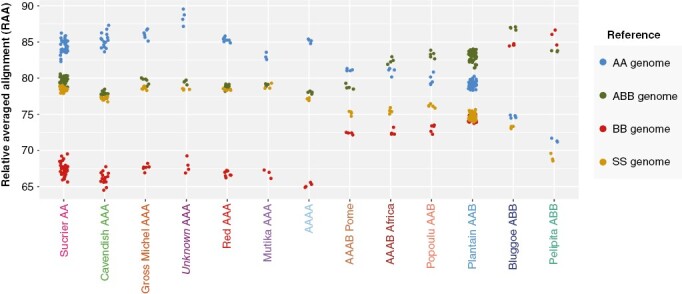
Relative averaged alignment (RAA) metric per accession (dots) grouped into 13 clusters against four reference genomes (colours) representing the donor A, B and S contributors to cultivated banana, plus a long-read assembly of a hybrid ABB accession.

The RAA was calculated for each sample and plotted by genetic cluster (Fig. 4). For comparison, the percentage of aligned paired-reads per sample and reference before RAA normalization are shown in Supplementary Data Fig. S5, and decomposed by genome reference in Fig. S6.

The AA/AAA/AAAA accessions showed the highest RAA to the A-genome (Fig. 4, blue dots) and a significantly lower RAA to the B-genome (red dots), while RAA to the ABB-genome (green dots) and the S-genome (yellow dots) were similar. The RAA to the A-genome was higher in the ‘unknown AAA’ cluster than in the other AA/AAA/AAAA accessions, and lower in ‘Mutika AAA’. This supports a higher contribution of *M. acuminata* subsp. *malaccensis* (A-genome reference) to the ‘unknown AAA’ accessions. The RAA decreased for the A-genome and increased for the B-genome for accessions with a B-genome contribution (AAB, AAAB, ABB). ‘Plantain AAB’ had a higher RAA to the B-genome than ‘Popoulu AAB’. The ‘Bluggoe ABB’ accessions had the highest alignment rate to the ABB-genome and not the B-genome since the former was generated from an accession from the Bluggoe subgroup. ‘Pelipita ABB’ was the only cluster with the highest RAA to the B-genome.

**Fig. 5.**
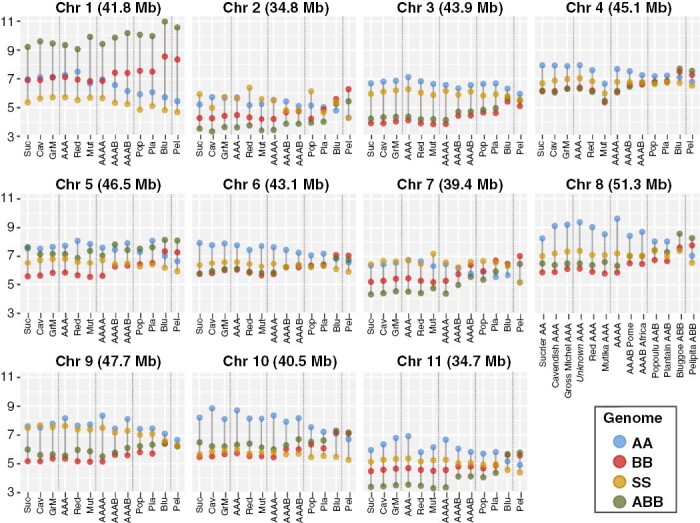
Relative averaged alignment (RAA) metric per accession (dots) grouped into 13 clusters against each of the 11 base chromosomes of the four reference genomes (colours) representing the donor A, B and S contributors to cultivated banana, plus a long-read assembly of a hybrid ABB accession.

The RAA to each of the reference genomes was also calculated for each of the individual 11 chromosomes (Fig. 5) to clarify the contribution from each A-, B- and S-genome donor. We decided not to normalize RAA by chromosome lengths, as results could be plotted and interpreted together without adding an extra transformation. Most chromosomes in a genetic cluster showed similar RAA values, as evidenced by similar patterns in Fig. 5. Deviating from the general pattern, chromosome 2 in ‘Sucrier AA’, ‘Red AAA’, ‘Popoulu AAB’ and ‘Mutika AAA’ showed the highest alignment rate to the S-genome (instead of to the A-genome); chromosome 7 in ‘Mutika AAA’ and ‘Popoulu AAB’ showed a notably higher RAA to the S-genome than to the A-genome; and chromosome 7 in ‘Plantain AAB’, ‘Bluggoe ABB’ and ‘Pelipita ABB’ showed the highest RAA to the B-genome. RAA values between ‘AAAB Pome’ and ‘AAAB Africa’ were very similar except in chromosome 7 (Fig. 5). Notably, the clusters ‘unknown AAA’ and ‘Gros Michel AAA’ did not differ (Fig. 5).

The previous RAA metrics were also calculated in each individual accession to confirm somatic clones. These multiple plots are available in Supplementary Data File S2. RAA values to each reference genome were similar among individual accessions in a genetic cluster, as expected among somatic clones. Exceptions were sample 142 (accession ‘LAMIEL’) within the ‘Plantain AAB’ cluster, particularly in chromosome 4, and sample 66 (accession ‘PELIPITA’) in the ‘Pelipita ABB’ cluster, particularly in chromosome 9. While the tetraploid genetic clusters (AAAA/AAAB) were not clonal, RAA values among accessions within these clusters were also very close to each other.

### Contribution from the A- and B-genomes along chromosomes

Using simultaneous alignments to the A- and B-genome references, we could compare changes in read depth between A and B references (named ‘Relative Coverage’) and estimate the changes in donor contribution along chromosomal regions. For example, an AAB accession needs to align twice to the A reference for each alignment to the B reference *on average*, it is the deviation from this average that allows us to identify introgressions.

**Fig. 6.**
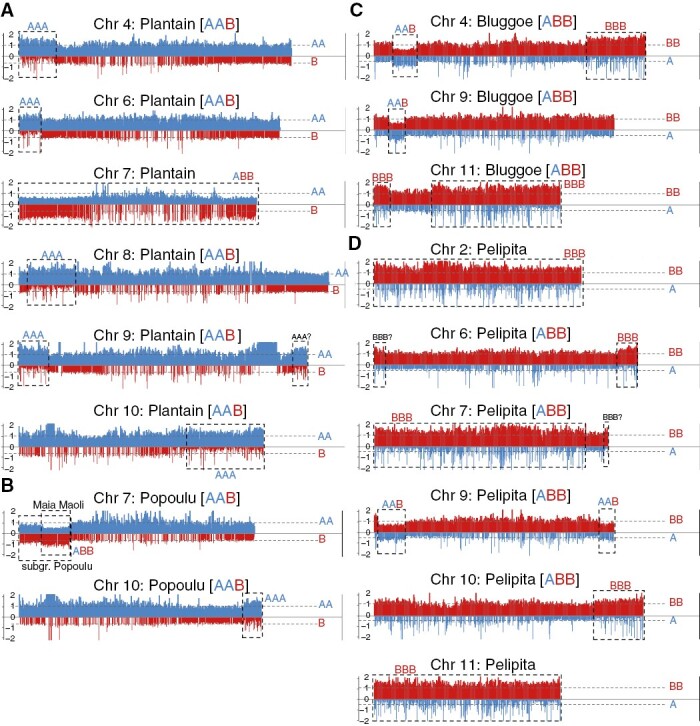
Introgression (boxes) identified in the four AAB and ABB clusters (A–D) based on relative read coverage or depth to the A- and B-genome references. The A-genome is always plotted in blue and coverage in the B-genome is always plotted in red. Two plots were produced in parallel to account only for the regions conserved, either containing all the windows/regions in the A-genome and the conserved homologous windows in the B-genome below (blue on top of red) or the inverse (red on top of blue). Coverage was normalized by dividing the window coverage by the chromosome average coverage to obtain values in the range 0–2. Because of the conservation between the A- and B-genomes, ‘background coverage’ was always observed between genomes.

The Relative Coverage for the AAB and ABB genetic clusters is shown in Fig. 6 and Supplementary Data File S3. The ‘Plantain AAB’ cluster, which includes the Africa-origin plantains (De Langhe, 2015), evidenced a 2:1 proportion between the A- and B-genomes (AAB) in all chromosomes except in chromosome 7 (Fig. 6A). The proportion in chromosome 7 was 1:2 (ABB) along the whole chromosome. In addition, several A-donor introgressions, evidenced by 3:0 ratios (AAA), were observed in chromosomes 4, 6, 8, 9 and 10 (highlighted in boxes in Fig. 6A).

The ‘Popoulu AAB’ cluster (Fig. 6B), which included the Pacific-origin plantains (De Langhe, 2015), had a 2:1 (AAB) proportion along all chromosomes, except for an A-donor introgression at the end of chromosome 10, evidenced by a 3:0 coverage ratio (AAA), and a B-donor introgression at the start of chromosome 7, evidenced by a 1:2 coverage ratio (ABB). This B-donor introgression at the start of chromosome 7 started at the beginning of chromosome 7 in accessions 126 and 189, but was shorter and started 1/8th within chromosome 7 in the three ‘Maia maoli’ accessions in the genetic cluster (accessions 54, 55 and 125). These accessions are individually plotted in Supplementary Data Fig. S7.

The ‘Bluggoe ABB’ cluster (Fig. 6C) had a 1:2 ratio (ABB) along most chromosomes, except for five introgressions: three B-donor introgressions, evidenced by a coverage ratio of 0:3 (BBB), in chromosomes 4 and 11 (×2); and two A-donor introgressions, evidenced by a 2:1 coverage ratio (AAB), in chromosomes 4 and 9.

The ‘Pelipita ABB’ cluster had the expected 1:2 (ABB) coverage ratio, except the complete chromosomes 2, 7 and 11 showed a 0:3 (BBB) coverage ratio (Fig. 6D). These full chromosomal exchanges were identified in all three accessions in the cluster. In addition, two A-donor introgressions, evidenced by a 2:1 coverage ratio (AAB), were noticed in chromosome 9, and two B-donor introgression in chromosomes 6 and 10 (Fig. 6D).

The relative coverage for the synthetic tetraploids was consistent with their progenitors’ composition, as shown in Supplementary Data Fig. S8 (AAAB clusters) and Fig. S9 (AAAA cluster). All 11 chromosomes in ‘AAAB Pome’ had a coverage ratio consistent with an AAAB composition (i.e. there was no evidence for introgressions). However, chromosome 7 in the ‘AAAB Africa’ cluster showed a 2:2 ratio (AABB), which was not observed in the ‘AAAB Pome’ cluster (Fig. S8). This is similar to the introgression observed in the ‘Plantain AAB’ cluster, indicating the ancestral origin via parental contribution of the observed introgression.

The relative coverage for the AA/AAA genetic clusters did not evidence B-donor introgressions, as shown in Supplementary Data File S4. We also generated plots for the individual accessions to identify any rearrangements specific to one individual (File S5).

### Contribution of the A- and S-genome along chromosomes

**Fig. 7.**
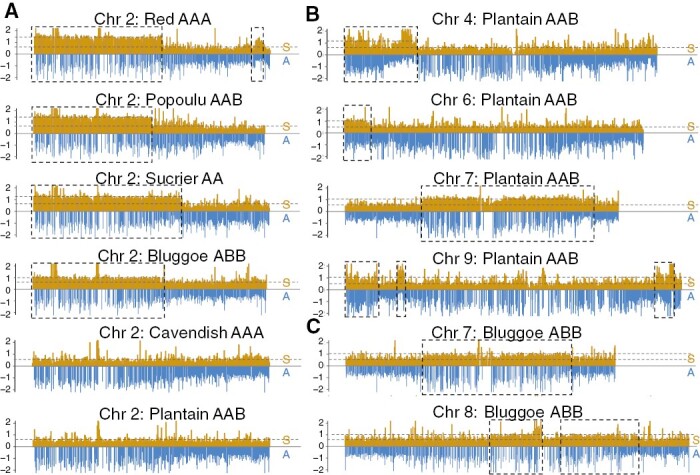
Introgression (boxes) in chromosome 2 in four varieties with different compositions based on relative read coverage or depth to the A- and S-genome references (blue and yellow, respectively). Two plots were produced in parallel containing all the windows/regions in the S-genome and the conserved homologous windows in the A-genome below (yellow on top of blue). Coverage was normalized by dividing the window coverage by the chromosome average coverage to obtain values in the range 0–2.

Relative Coverage to the A- and S-genomes revealed an introgression from the S-genome on the first half of chromosome 2 in the ‘Red AAA’, ‘Popoulu AAB’, ‘Bluggoe ABB’ and ‘Sucrier AA’ genetic clusters (Fig. 7). We also identified S-genome introgressions in chromosomes 4, 6, 7 and 9 in ‘Plantain AAB’, and in chromosomes 7 and 8 in ‘Bluggoe ABB’ (Fig. 7). No further intergenomic recombinations were found in any other chromosome or genetic cluster (Supplementary Data File S6). The coverage and length of this introgression in chromosome 2 were not similar among clusters, but overall the result suggests these genetic clusters originated from closely related A-donor ancestors.

In addition, we calculated the Relative Coverage between A and S for each chromosome (Supplementary Data Table S4). The ‘Mutika AAA’ cluster had the lowest A/S coverage ratio over all the chromosomes compared to the other clusters, except in chromosome 2, where the four clusters containing the introgression had the lowest ratio.

## DISCUSSION

To verify the extent of the varietal groups in our diversity panel, our analyses first established the relationship between banana accessions, which showed phenotypic diversity before genotyping analysis. Edible banana crops are vegetatively propagated, so their genetic diversity is essentially fixed over time, and the genotypes in a varietal group putatively share a common origin and are somatic clones of each other (Heslop-Harrison and Schwarzacher, 2007). Since the genetic diversity of the varietal groups is fixed over time, the phylogeny observed between the varietal clusters reflects the genetic distance between the donor ancestors that established each clonal lineage. Nevertheless, it was unsurprising that we found a genetic variation despite the absence of sexual reproduction: millennia of diversification of wild genotypes and human selection of hybrids have led to hundreds of edible banana varieties (Christelová *et al.*, 2017).

We later characterized subgenome composition and identified introgressions in each varietal clonal cluster and individual accession. These results clarify shared foundational events between lineages and add to the growing evidence (Baurens *et al.*, 2019; Martin *et al.*, 2020, 2023; Cenci *et al.*, 2021) that most clonal lineages are probably the product of several hybridization and backcrossing events.

In particular, we identified and resolved variety-specific chromosomal exchanges and imbalances between A/B/S homoeologous chromosomes, as part of a forward genetics pipeline to discover target genes for traits of interest. Since the edible banana genomes are clonal and fixed on time, these introgressions are a primary source of diversity for genetic advances (e.g. for genome editing) as they usually result in CNVs. Some genome regions are naturally more prone to CNVs as a probable natural evolution to enable the emergence of new gene copies and expression profiles. CNVs alter gene dosage (Beckmann *et al.*, 2007; Christie *et al.*, 2016), so these regions constitute a fixed source of genetic diversity associated with phenotypic plasticity (Jia *et al.*, 2015; Baggs *et al.*, 2017).

### Defining clonal varietal groups in a panel representative of 13 varietal clonal lineages

We genotyped most of the accessions held by AGROSAVIA in Palmira, Colombia, using short-read whole-genome sequencing. This is a comprehensive collection of introduced varieties for local and international cropping, containing a good range of cultivars, subspecies and wild relatives with observed phenotypic diversity. This diversity panel includes most of the common edible bananas. While it was sourced from a Colombian genebank, clonal crops cannot adapt to the local environment and these accessions are found worldwide. Somatic clones are available from the ITC in most cases. However, our population structure analysis using principal component, phylogenetic and admixture analysis evidenced extensive redundancy among clonal accessions.

We divided 151 accessions into 13 genetic clusters, containing between three and 55 accessions, based on five genetically distinct ancestries using STRUCTURE analysis (Fig. 1). The ‘admixture model’ of STRUCTURE assumes that each individual has a genetic make-up from one or more of *K* distinct ancestries. In clonally propagated species, such as bananas, the *K* distinct sources reflect the shared ancestry (i.e. the phylogenetic relationship) among the donors involved in establishing each clonal lineage. Seventeen accessions were wild *M. acuminata* (ten accessions), *M. balbisiana* (two accessions) or other wild *Musa* spp. (five accessions), and 23 were unclustered hybrid cultivars that were members of varietal clonal lineages poorly represented in our diversity panel (Silk AAB, Pisang, etc.).

Passport information was used to label each genetic cluster to a specific cultivar variety and define *clonal varietal groups*. We could not link one genetic cluster to a banana variety, because the passport data were inconclusive, and therefore we referred to it as ‘unknown AAA’ in downstream analysis. Three clusters corresponded to the synthetic tetraploids from breeding programmes, which grouped based on the composition of the paternal varieties from which they originated. Common cultivars were still absent in our panel (Ambon, Rio, Orotava, etc.). Since accessions held in genebanks other than the ITC have not been genotyped (Rouard *et al.* 2021), we verified or corrected the passport information based on the genetic analysis in Supplementary Data Table S1.

### Alignment metrics to identify subgenome composition

We established a new method, called RAA, by quantifying the normalized relative alignment from each accession to three reference banana genomes, which are representative of the A-, B- and S-genome donors. We called this normalized alignment metric ‘relative averaged alignment’ (RAA). The RAA accounts for the technical variation between samples and reference bias, namely the phylogenetic distance between a variety and a genome reference. The RAA allowed us to identify subgenome composition (AA, AB, AAA, AAB, ABB, etc.) and compare it with the data available in the MGIS database (Ruas *et al.*, 2017).

Later, we used comparisons of read depth, which we called Relative Coverage, to identify introgressions. Our method is different from methods previously used in banana (Baurens *et al.*, 2019; Martin *et al.*, 2020, 2023) for introgression detection, which were based on a database of SNPs found to be exclusively present (private SNPs) in a panel of accessions of a given donor ancestor. We believe our alignment-based methods (RAA and Relative Coverage) offer several advantages: (1) the alignment-based methods do not require sequencing a large number of donors to find private SNPs exclusive to each donor gene pools; (2) the alignment-based methods can be used in closely related gene pools, even within a single species, where private SNPs can be hard to find because of recent divergence – this has allowed us to identify introgressions between indica and japonica rice (Higgins *et al.*, 2021), or between Andean and Mesoamerican beans (our unpubl. data); and (iii) the alignment-based methods can be easily scaled up, as they do not require SNP calling and analysis, allowing quick incorporation in the studies of new diversity panels and the increasing number of long-read genome assemblies. On the other hand, the main disadvantage of Relative Coverage is it requires experience to distinguish introgressions in low donor ratios.

### Lineage-exclusive introgressions in the AAB varietal groups

Traditional starchy bananas are dominated by two geographically discrete groups of AAB cultivars (De Langhe, 2015): plantains from the rainforest zone of Africa and the Maoli-Popo’ulu subgroup of plantains. The Popoulu subgroup shows maximum diversity in Melanesia and is often called Pacific Plantains. In our study, these groups correspond to the named ‘Plantain AAB’ and ‘Popoulu AAB’ clusters, comprising 55 and five accessions, respectively. Both subgroups would have their origin in Indonesia and the Philippines, and would share *M. acuminata* subsp*. banksii* as an A-genome donor (Perrier, 2009; Perrier *et al.*, 2011).

The main difference we observed between these two AAB plantain clusters was a B-donor (ABB composition) exchange of the complete chromosome 7 in ‘Plantain AAB’ that was not present in ‘Popoulu AAB’ (Fig. 6). This resulted in ABB composition in chromosome 7 in ‘Plantain AAB’, which had been previously described in a single sample, ‘French Clair’, representative of the French division of African Plantains (Baurens *et al.*, 2019). The results are also consistent with the compositions reported for Plantains (samples 146–149) in Martin *et al.* (2023: fig. S3).

We also observed a B-donor introgression (ABB) at the start of chromosome 7 in ‘Popoulu AAB’ (Fig. 6). The Maoli-Popo’ulu subgroup of plantains can be divided into ‘Maoli’ and ‘Popoulu’ subdivisions based on the shape of the fruit (Ploetz, 2007). This B-donor introgression (ABB) at the start of chromosome 7 was shorter in the three ‘Maia Maoli’ accessions (samples 54, 55 and 125) compared to the two ‘Popoulu subdivision’ accessions in the cluster (Supplementary Data Fig. S7). Martin *et al.* (2023) observed the ‘shorter version’ of the introgression in one ‘Popoulu’ accession (sample 151) but reported no introgressions in chromosome 7 in a ‘Maia Maoli’ accession. By contrast, we propose that this B-donor introgression (ABB) at the start of chromosome 7 is present in all of the Maoli-Popo’ulu subgroup but has different length in the two subdivisions.

In addition, we observed five A-donor telomeric introgressions (AAA) in African plantains (‘Plantain AAB’) that were not present in Pacific plantains (‘Popoulu ABB’). These AAA introgressions were at the start of chromosomes 4, 6, 8 and 9, and at the end of chromosomes 9 and 10 (Fig. 6). All these regions showed an AAA composition instead of AAB (Fig. 6). The results are also consistent with the compositions reported for Plantains (samples 146–149) in Martin *et al.* (2023: fig. S3). The genome proportion plots by individual sample and chromosome (Supplementary Data File S2) show that these introgressions are similar in all 30 ‘Plantain AAB’ evaluated except for cultivar Lamiel (sample 142), which notably showed a B-donor introgression (ABB composition) in the second half of chromosome 4 that was not observed in any other plantain accession by us or in the literature. Variation in a single accession was uncommon in our analysis (File S2). We hypothesize that genetic diversity observed in this single accession could be associated with somaclonal variation associated with *in vitro* propagation of germplasm (Šimoníková *et al.*, 2019, 2022; Houwe *et al.* 2020).

### Full chromosome exchanges and introgressions in the ABB varietal groups

The full exchanges in chromosomes 2, 7 and 11 (Fig. 6) in the ‘Bluggoe ABB’ and ‘Pelipita ABB’ clusters in our study resulted in BBB composition in these loci. These compositions have already been described for a single accession in both groups (Cenci *et al.*, 2021) and another Pelipita accession (Martin *et al.*, 2023). Differences between these two ABB groups can be confirmed in chromosomes 2 and 7 based on RAA alone (Fig. 5), but not in chromosome 11 because B-donor exchanges were present in both groups.

We also observed two A-donor introgressions (AAB) in chromosome 9, and three B-donor introgressions (BBB) in chromosomes 6 and 9. All these introgressions were noticed in all ‘Pelipita ABB’ accessions (Fig. 6) and initially reported by Baurens *et al.* (2019). The results are also consistent with the compositions reported for sample 157 in Martin *et al.* (2023: fig. S3). Notably, three ABB accessions did not cluster with the others on the PCA (Fig. 2) and our sample 66 showed a slightly different alignment pattern on chromosomes 9 and 10 (Supplementary Data File S2). However, read coverage was not sufficient to confirm recombination in this individual accession (sample 66).

In ‘Bluggoe ABB’, we observed two A-donor introgressions (AAB) in chromosomes 4 and 7, and three B-donor introgressions (BBB) in chromosomes 4 and 11. These introgressions were previously reported (Baurens *et al.*, 2019). The BBB contribution in chromosome 11 may have a different origin between ‘Pelipita ABB’ and ‘Bluggoe ABB’, as it did not include the complete chromosome 11 in the latter.

### Relationship between the synthetic tetraploid groups

Three genetic clusters were synthetic tetraploid hybrids based on the MGIS passport data and our observations of alignment and coverage properties, one with AAAA composition and two with AAAB composition. We were able to find the parentage of some of these accessions (Supplementary Data Table S5). The AAAA genetic cluster grouped with the AAA Cavendish and Gros Michel triploid groups, which agrees with the parentage of sample 98 (FHIA17).

The ‘AAAB Pome’ tetraploid cluster was named after the parental contributors of the B-genome, which is confirmed in the case of the subgr. Pome plantain ‘Prata ana’ in sample 65. As a result of this origin, these tetraploid accessions clustered close to FHIA-1 (also with a subgr. Pome parental contributor) and the AAB subgr. Pome cultivar ‘Figue Famile’. Similarly, the ‘AAAB Africa’ tetraploid cluster was named after the B-genome donor of accessions FHIA-31 and FHIA-20, namely AVP-67, a French AAB Plantain from Africa. Consequently, these accessions inherited and showed the ABB composition in chromosome 7 representative of ‘Plantain AAB’ members (Supplementary Data Figs S8 and S9). All the other chromosomes showed an AAB composition.

### Relationship between the AA/AAA varietal groups

We verified the close relationship between the AA/AAA varietal groups previously described. In particular, we identified no B-genome introgression in any AA/AAA group (Supplementary Data Fig. S7). This matches the results of Martin *et al.* (2023) in a parallel study. In detail, our PCA evidenced Cavendish (AAA) and Gros Michel (AAA) overlapped (Fig. 2). The close genetic relationship between Gros Michel and Cavendish has been previously evidenced by genotyping (Christelová *et al.*, 2017), a common Mchare donor (Raboin *et al.*, 2005), and a reciprocal translocation between chromosomes 3 and 8 (Šimoníková *et al.*, 2019). The ‘Sucrier AA’ and ‘Red AAA’ clusters were close but distinguishable from the former despite Sucrier and Cavendish sharing ancestry (Martin *et al.*, 2020). Diploid Sucrier accessions comprise a varying mosaic of *M. acuminata* subsp*. banksii*, *zebrina* and *malaccensis* and wild ‘Pisang Madu’ banana (Martin *et al.*, 2020, 2023). While we did not observe genetic variation among Sucrier accessions in our tree (Fig. 3) or ancestry analysis, there were three Sucrier samples separated from the others in the PCA (Fig. 2).

Mutika has been described as genetically distinct from the other triploid AAA bananas, which was also evidenced in our phylogenetic and principla components analyses. Generally, the Mutika (AAA) cultivars (East African highland bananas) have diversified by somatic mutation to have several end uses (Kitavi *et al.*, 2016, 2020), and now harbour significant epigenetic diversity (Kitavi *et al.*, 2020). However, Mutika cultivars are genetically uniform, since they probably arose from a single ancestral clone introduced from Asia into Africa that subsequently underwent population expansion by vegetative propagation (Kitavi *et al.*, 2016). Mutika cultivars distinctively contain two chromosome sets from subsp. *Zebrina* and one chromosome set from subsp. *Banksii* (Šimoníková *et al.*, 2019), and a Vε cytoplasmic type (Perrier, 2009).

### Contribution of the S-genome to cultivated banana

*Musa schizocarpa* (S genome) has contributed to cultivated banana (Heslop-Harrison and Schwarzacher, 2007; D’Hont *et al.*, 2012; Martin *et al.*, 2023). *Musa schizocarpa* and *M. acuminata* subsp. *banksii* are sympatric in Papua New Guinea (Christelová *et al.*, 2017), where AS natural hybrid cultivated bananas are found (Daniells, 2001). *Musa schizocarpa* is more closely related to *M. acuminata* than to *M. balbisiana* (Li *et al.*, 2010, 2013), which explains the relatively high coverage in the S-genome in all the accessions, particularly the AAA samples, when using our alignment-based techniques (RAA and Relative Coverage). However, the much-increased S-genome coverage in chromosomes clearly evidenced intergenomic recombination between these subgenomes, when present, by RAA alone (Fig. 5). For example, we confirmed S-donor introgressions (Fig. 7) in chromosome 2 in four clonal lineages: Bluggoe (ABB), Sucrier (AA), Popoulu (AAB) and Red (AAA); in chromosomes 4, 6, 7 and 9 in ‘Plantains ABB’; and in chromosomes 2, 7 and 8 in ‘Bluggoe ABB’. ITS analysis revealed *M. schizocarpa* S-contribution to the Mutika subgroup (Němečková *et al.*, 2018). However, we did not find S-genome introgressions in the Mutika group (not reported in the literature) but the alignment statistics suggested this group was less closely related to the donor of the A-genome reference.

## CONCLUSIONS

We identified the extent of ten varietal clonal groups of banana composed of somatic clones (i.e. varietal clonal lineages), using admixture, principal component and phylogenetic analyses, and later linked each clonal lineage to a common variety. We established alignment-based metrics, RAA and Relative Coverage, as direct and scalable methods to infer subgenome composition and chromosomal exchanges in hybrids, and demonstrated they can be used in the total genome and individual chromosomes in bananas or other crops without requiring sequencing multiple ancestors in the panel. We identified A/B introgressions among the AAB and ABB varieties, and A/S introgressions among all groups, using these methods. While these introgressions have been previously reported, we used a different computational approach (based on alignment coverage instead of private SNPs). As both papers are computational only, our results (obtained independently) reinforce the results from Martin *et al.* (2023). Our diversity panel also included multiple accessions from each cultivar, allowing us to delimit the extent of the clonal lineages. We confirmed previously reported intergenomic recombination for the Bluggoe and Pelipita clonal groups, and discussed some differences in the compositions reported for these groups by us and others in the literature that require further analysis.

## SUPPLEMENTARY DATA

Supplementary data are available at *Annals of Botany* online and consist of the following.

**Figure S1.** Estimation of the optimal number of *K* using the Evanno method for *K* = 2 to *K* = 10 against the A- and B-genomes.

**Figure S2.** Admixture analysis for *K* = 4 to *K* = 8 against the A-genome.

**Figure S3.** Admixture analysis for *K* = 4 to *K* = 8 against the B-genome.

**Figure S.:** Comparison of the clusters identified using admixture analysis in the A- vs. B-genomes.

**Figure S5.** The unnormalized percentage of properly paired reads aligning for the 13 genetic clusters, unclustered and wild accessions for the A-genome, B-genome, ABB-genome and S-genome reference.

**Figure S6**. The unnormalized percentage of properly paired reads aligning for the 13 genetic clusters, unclustered and wild accessions plotted separately for the A-genome, B-genome, ABB-genome and S-genome reference.

**Figure S7.** Relative read coverage plots against the A- and B-subgenomes in chromosome 7 for the five accessions in the ‘Popoulu AAB’ cluster.

**Figure S8.** Coverage plots showing A- and B-subgenome structure for the AAAB synthetic tetraploid clusters. The blue and red bars represent median coverage depth (normalized by mean) over 100 000-bp windows, for the A- and B-subgenomes, respectively.

**Figure S9.** Coverage plots showing A- and B-subgenome structure for the AAAA synthetic tetraploid clusters. The blue and red bars represent median coverage depth (normalized by mean) over 100 000-bp windows, for the A- and B-subgenomes, respectively.

**File S1.** Supplementary Tables S1–S5.

**File S2.** RAA metrics calculated in each individual accession to confirm somatic clones.

**File S3.** Relative coverage in the A- and B-subgenomes for the AAB and ABB genetic clusters.

**File S4.** Relative coverage in the A- and B-subgenomes for the AA and AAA genetic clusters.

**File S5.** Relative coverage in the A- and B-subgenomes for each individual accession.

**File S6.** Relative coverage in the A- and B-subgenomes for all genetic clusters.

**File S7.** Step by step guide and code to calculate RC and RAA.

mcad192_suppl_Supplementary_Figure_S1

mcad192_suppl_Supplementary_Figure_S2

mcad192_suppl_Supplementary_Figure_S3

mcad192_suppl_Supplementary_Figure_S4

mcad192_suppl_Supplementary_Figure_S5

mcad192_suppl_Supplementary_Figure_S6

mcad192_suppl_Supplementary_Figure_S7

mcad192_suppl_Supplementary_Figure_S8

mcad192_suppl_Supplementary_Figure_S9

mcad192_suppl_Supplementary_File_S1

mcad192_suppl_Supplementary_File_S2

mcad192_suppl_Supplementary_File_S3

mcad192_suppl_Supplementary_File_S4

mcad192_suppl_Supplementary_File_S5

mcad192_suppl_Supplementary_File_S6

mcad192_suppl_Supplementary_Data_S1

mcad192_suppl_Supplementary_Data_S2

## Data Availability

Germplasm is held in AGROSAVIA’s collection (MGIS: COL004) and is available on request. All sequence data used in this paper have been deposited as study PRJEB62882 in the European Nucleotide Archive at https://www.ebi.ac.uk/ena/browser/view/PRJEB62882. A step by step guide to calculate and plot RAA and RC metrics is included (Supplementary Data File S7). Additionally, this step by step guide and all scripts and code used in the computational analyses are available in a Github repository at https://github.com/jjdevega/Structural-diversity-in-banana-cultivars
