# Supplementary figures and images for: Characterizing subgenome recombination and chromosomal imbalances in banana varietal lineages
Source: Ann Bot. 2023 Dec 14;133(2):349–64. doi: 10.1093/aob/mcad192 (PMC11005773; doi:10.1093/aob/mcad192)

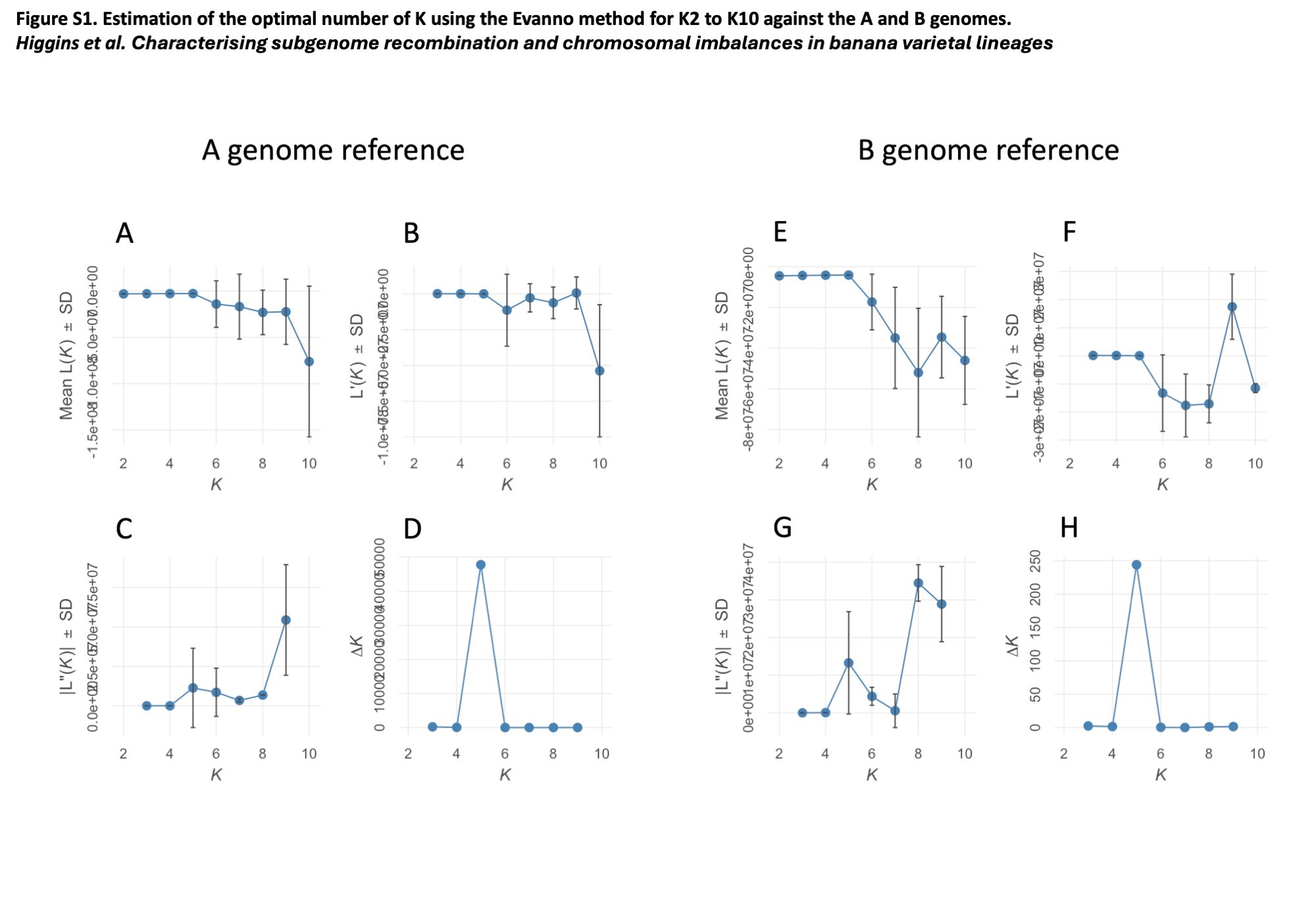

Supplement: mcad192_suppl_Supplementary_Figure_S1 [file mcad192_suppl_supplementary_figure_s1.jpeg]

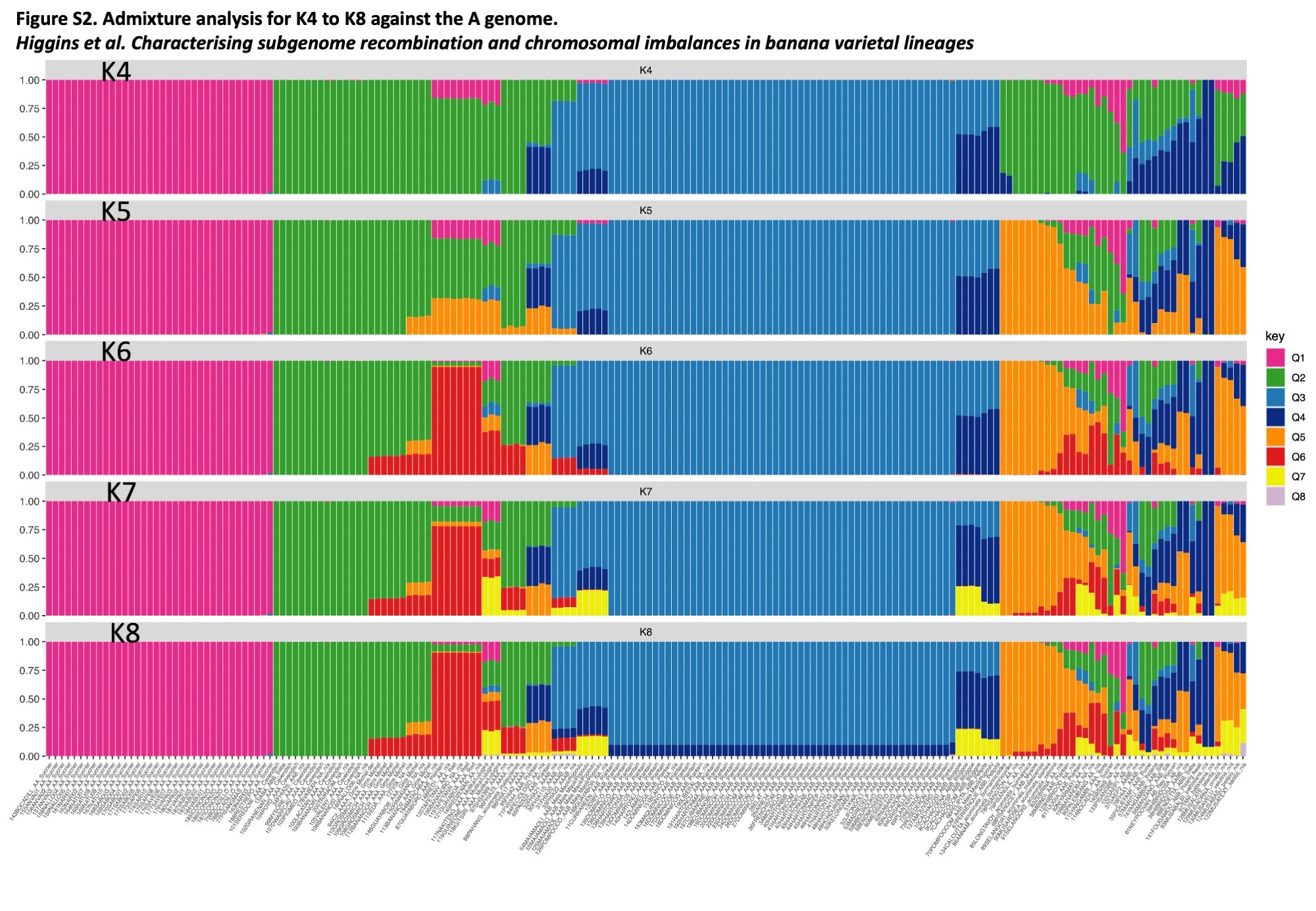

Supplement: mcad192_suppl_Supplementary_Figure_S2 [file mcad192_suppl_supplementary_figure_s2.jpeg]

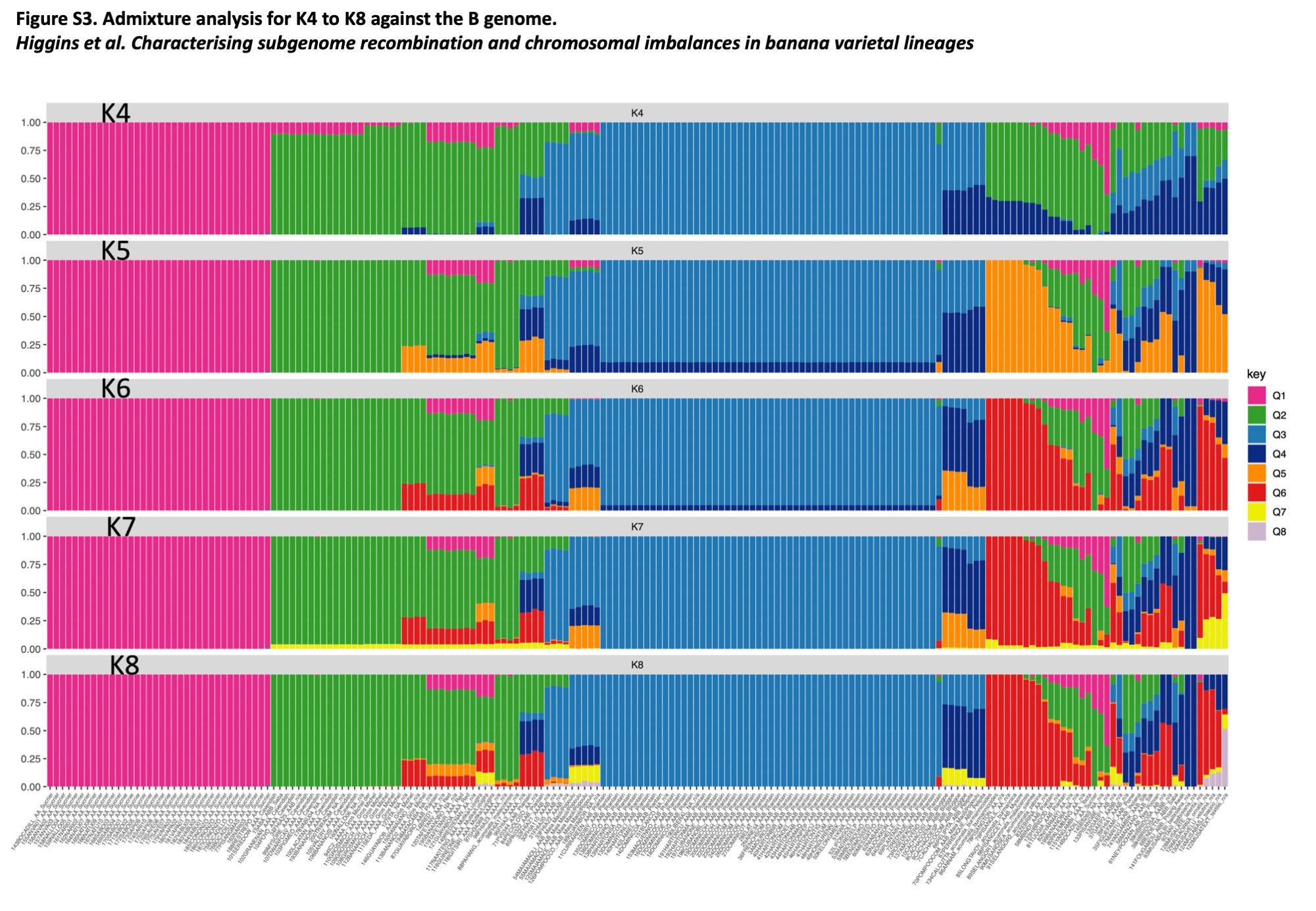

Supplement: mcad192_suppl_Supplementary_Figure_S3 [file mcad192_suppl_supplementary_figure_s3.jpeg]

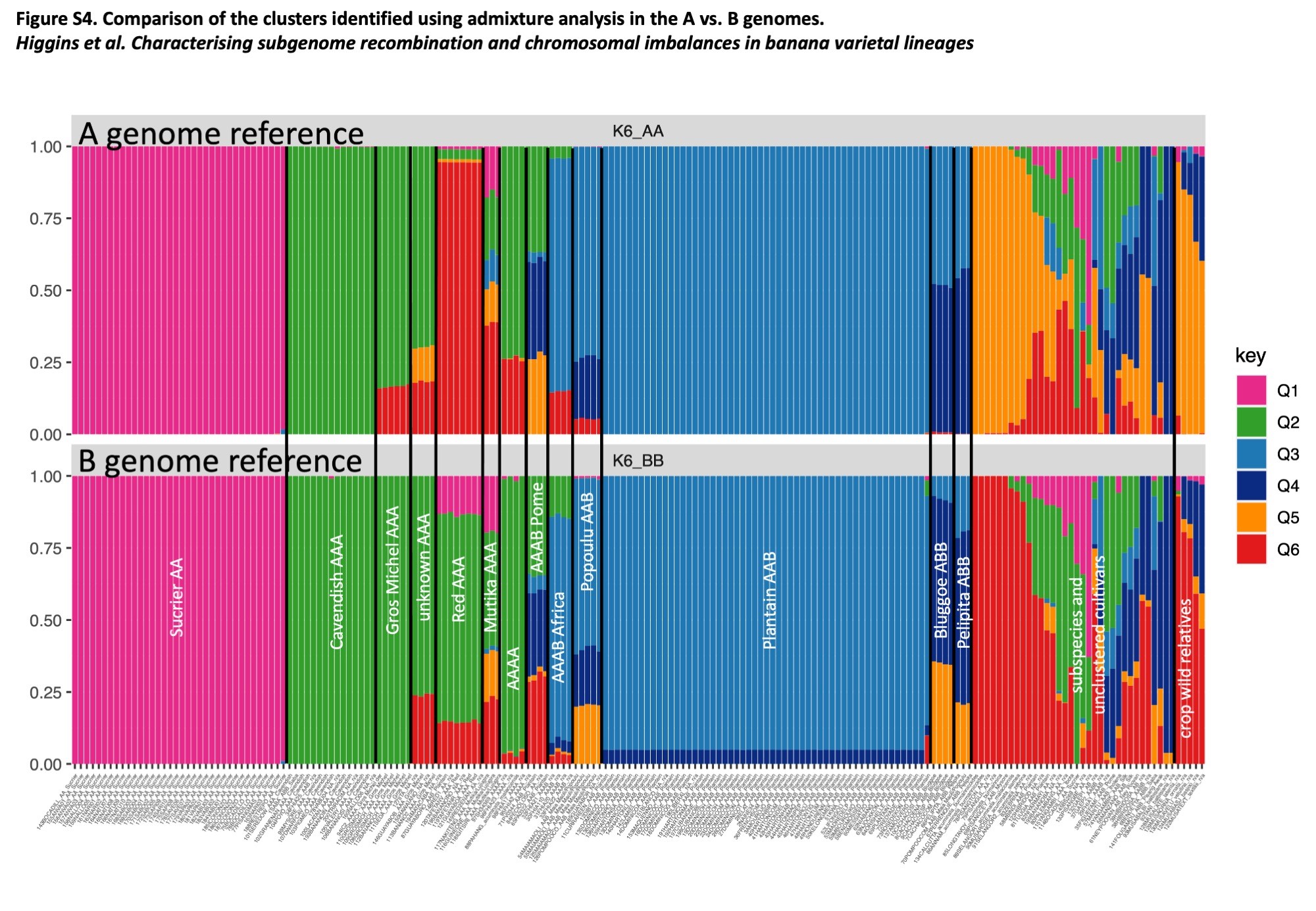

Supplement: mcad192_suppl_Supplementary_Figure_S4 [file mcad192_suppl_supplementary_figure_s4.jpeg]

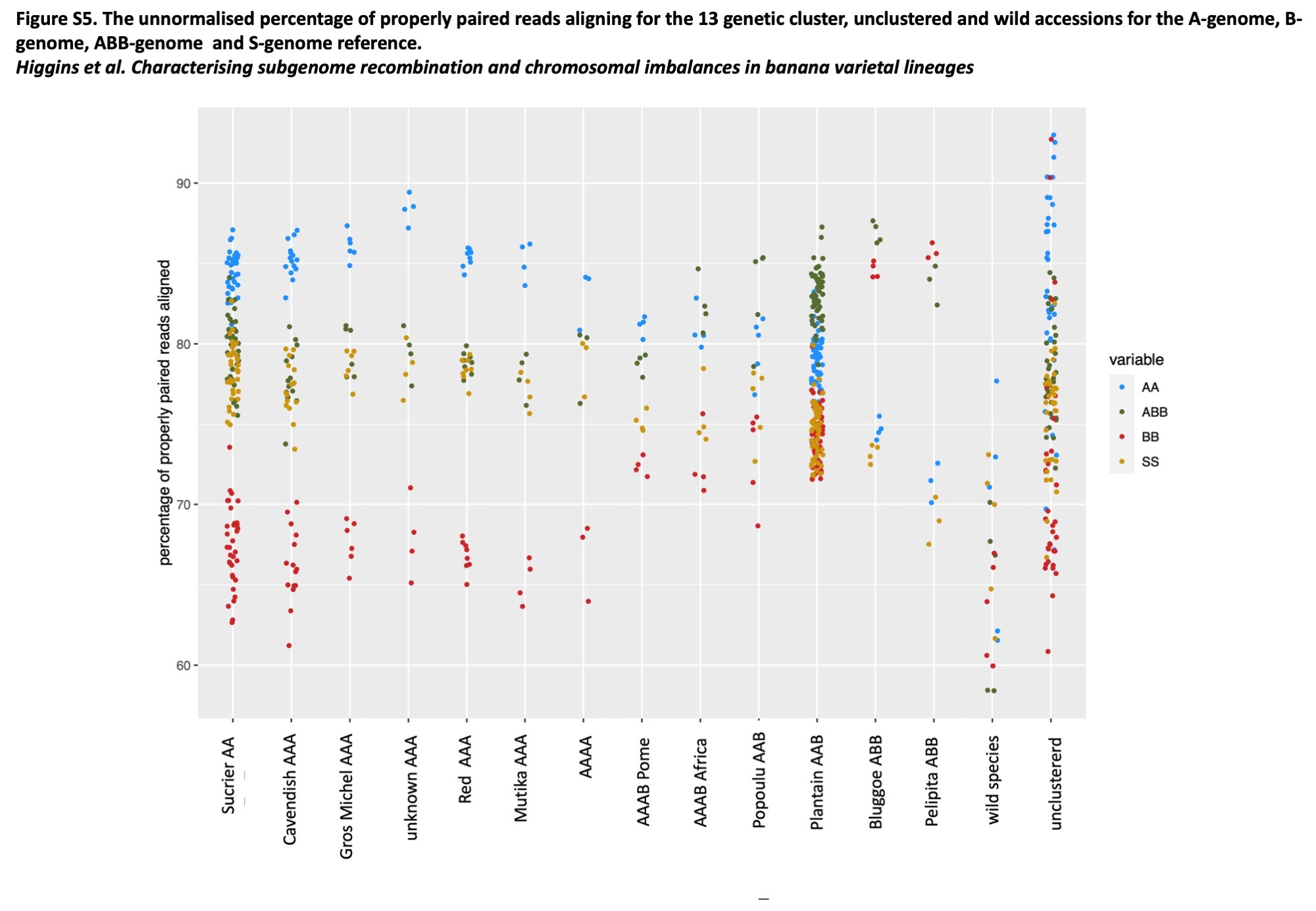

Supplement: mcad192_suppl_Supplementary_Figure_S5 [file mcad192_suppl_supplementary_figure_s5.jpeg]

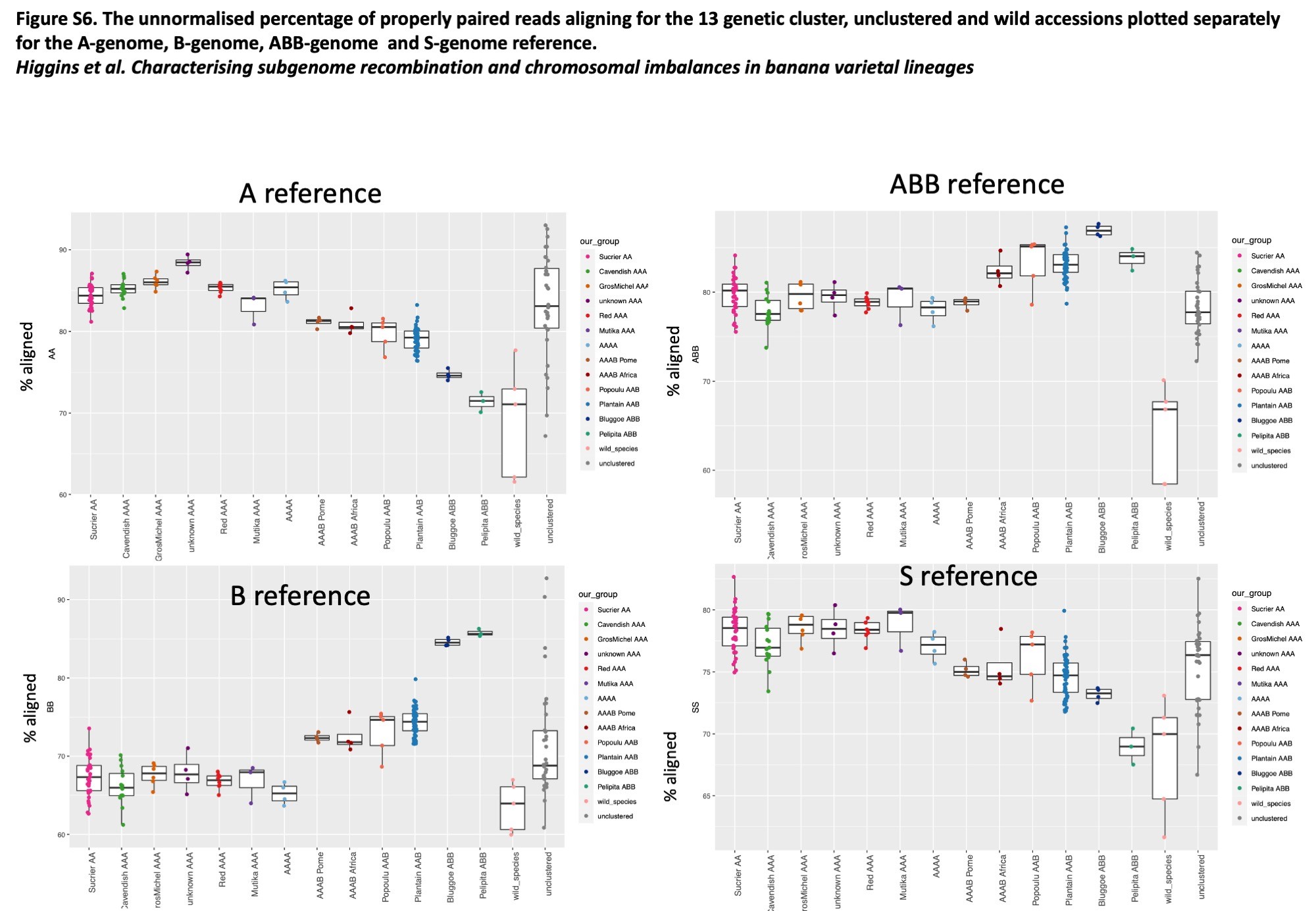

Supplement: mcad192_suppl_Supplementary_Figure_S6 [file mcad192_suppl_supplementary_figure_s6.jpeg]

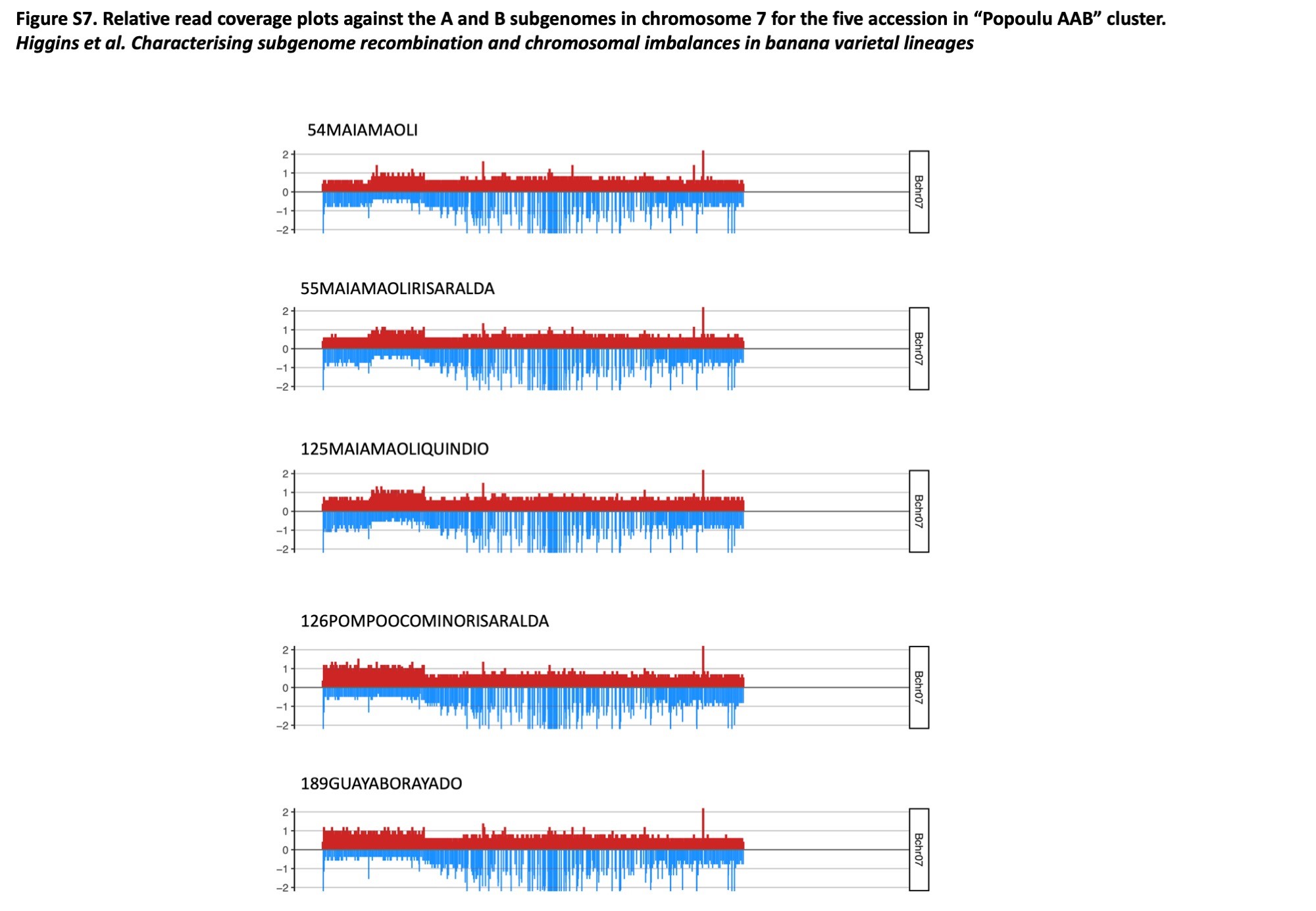

Supplement: mcad192_suppl_Supplementary_Figure_S7 [file mcad192_suppl_supplementary_figure_s7.jpeg]

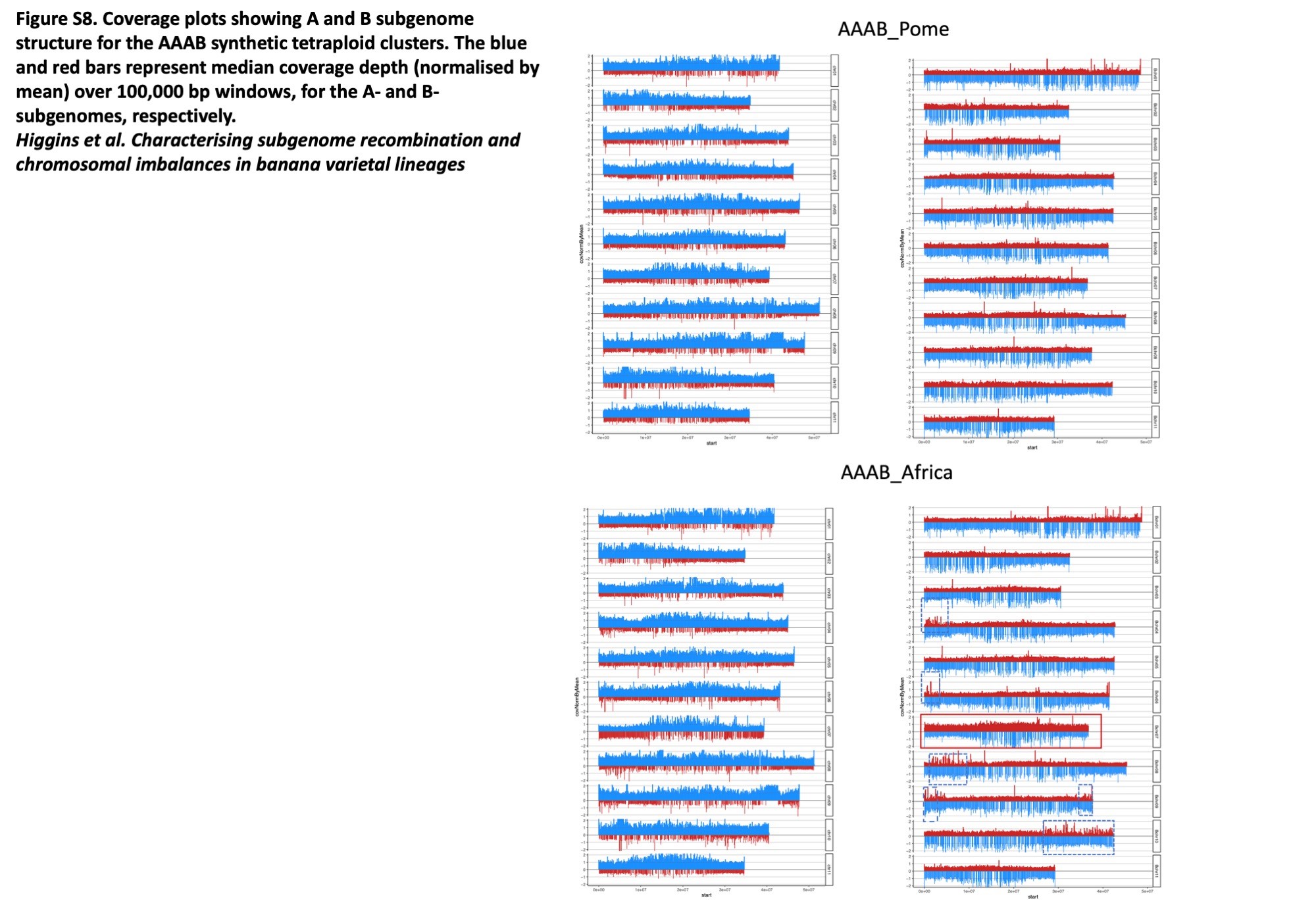

Supplement: mcad192_suppl_Supplementary_Figure_S8 [file mcad192_suppl_supplementary_figure_s8.jpeg]

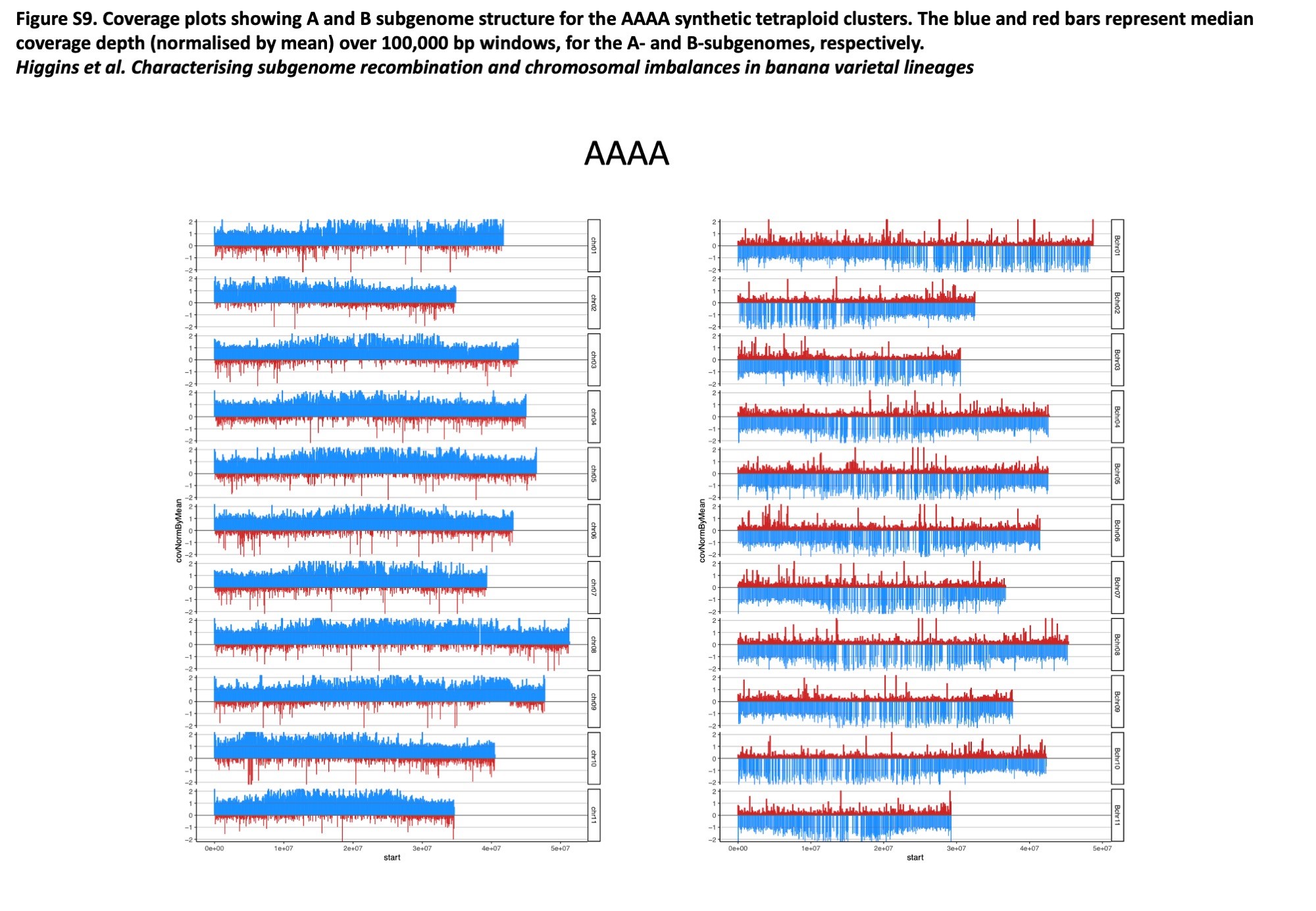

Supplement: mcad192_suppl_Supplementary_Figure_S9 [file mcad192_suppl_supplementary_figure_s9.jpeg]

## Sucrier AA

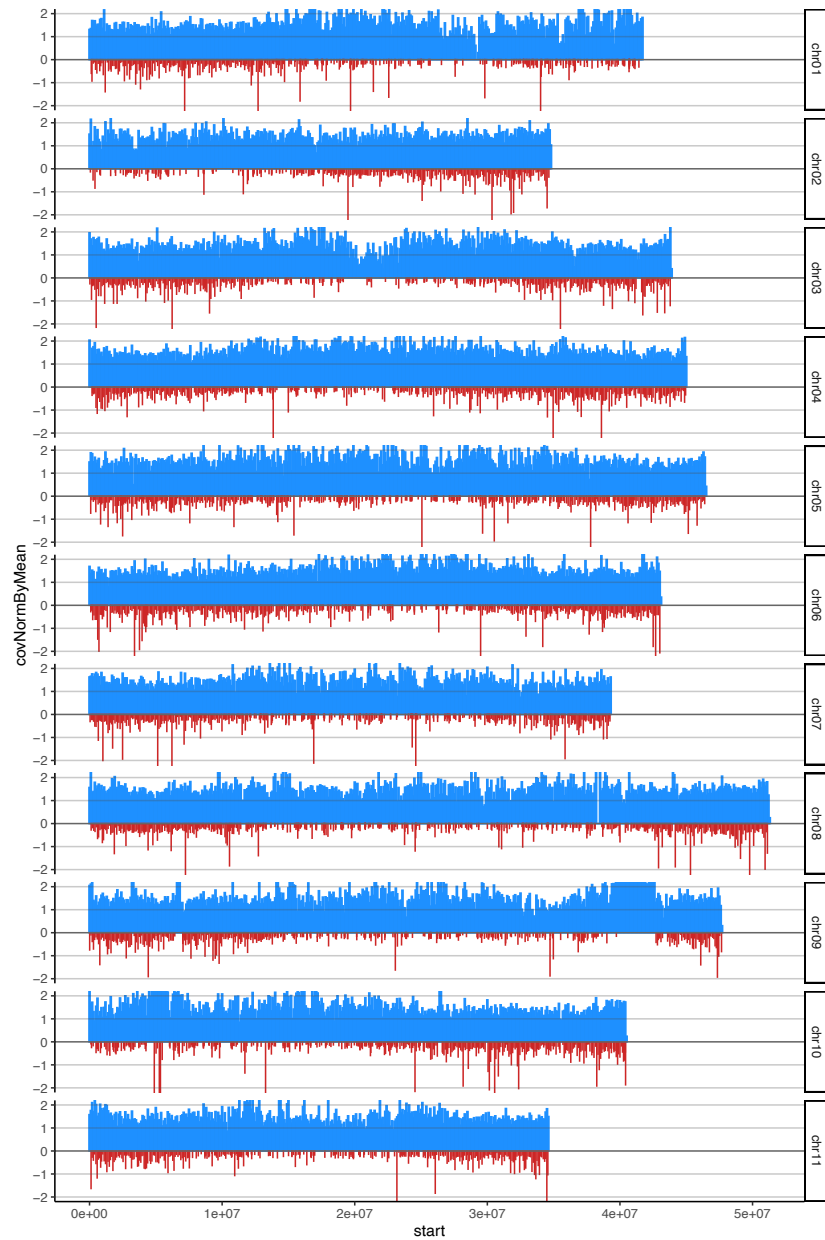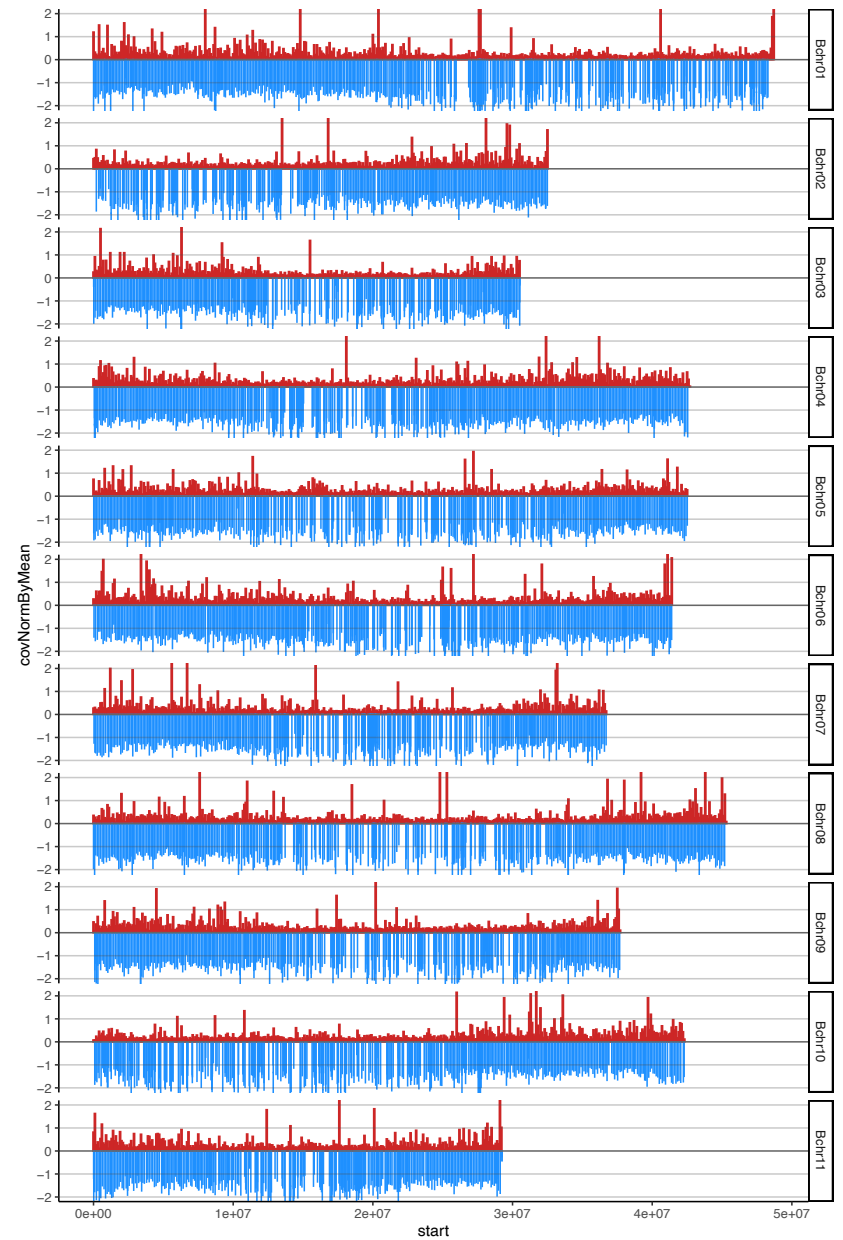

## Cavendish\_AAA

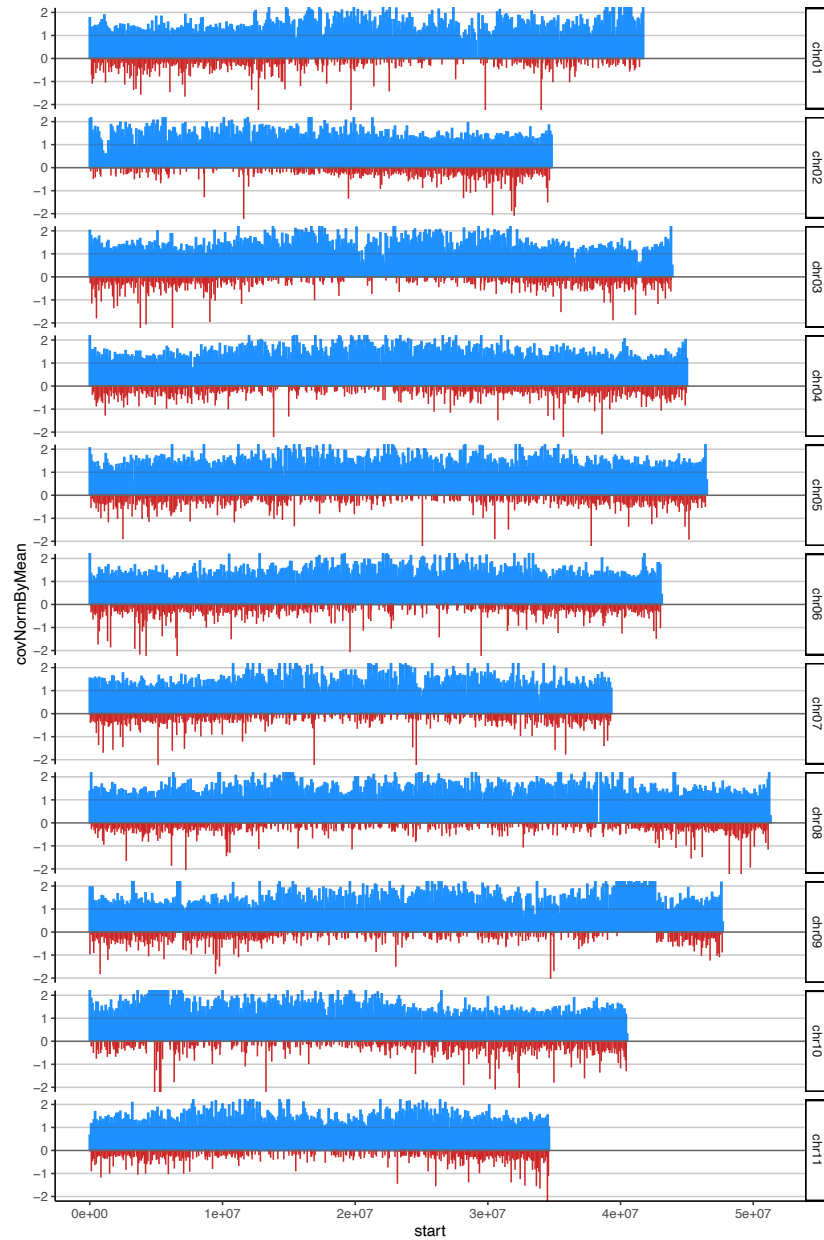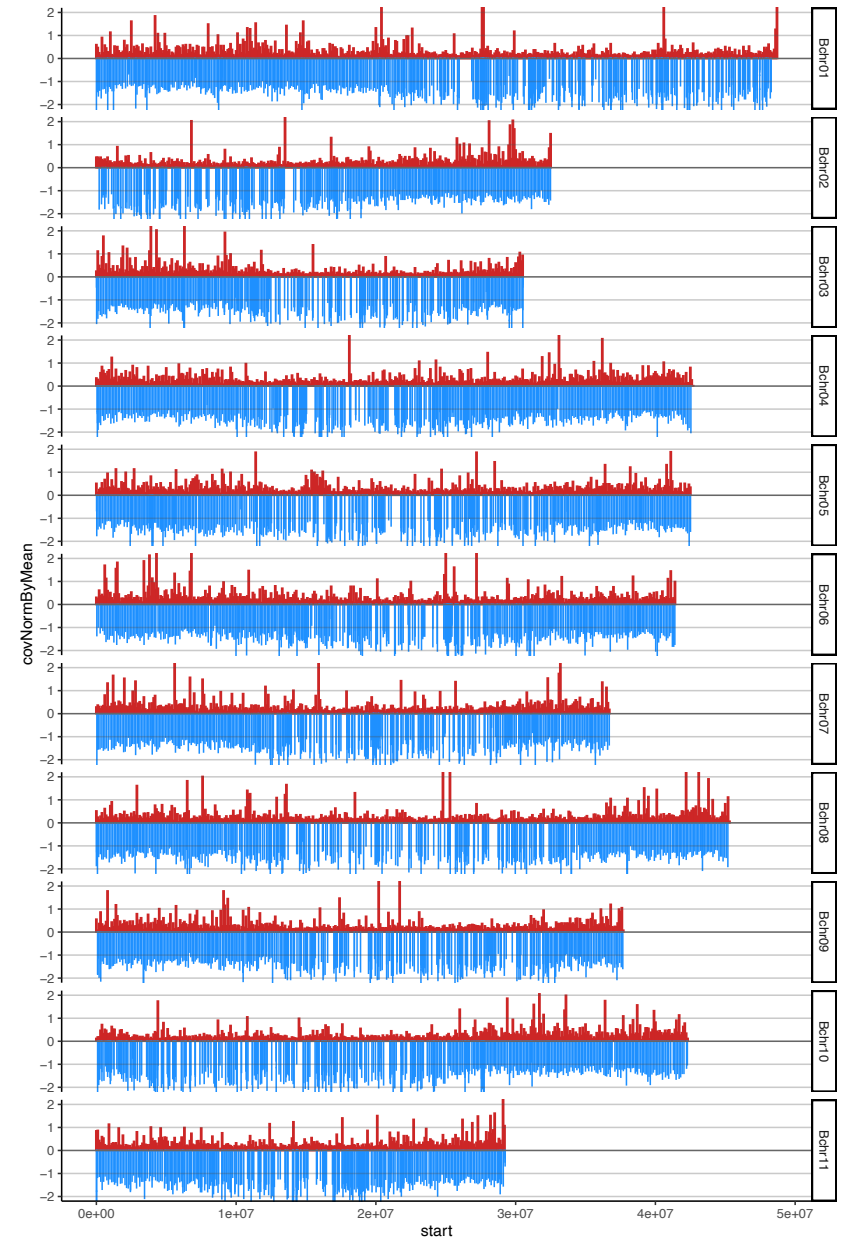

Gros Michel\_AAA

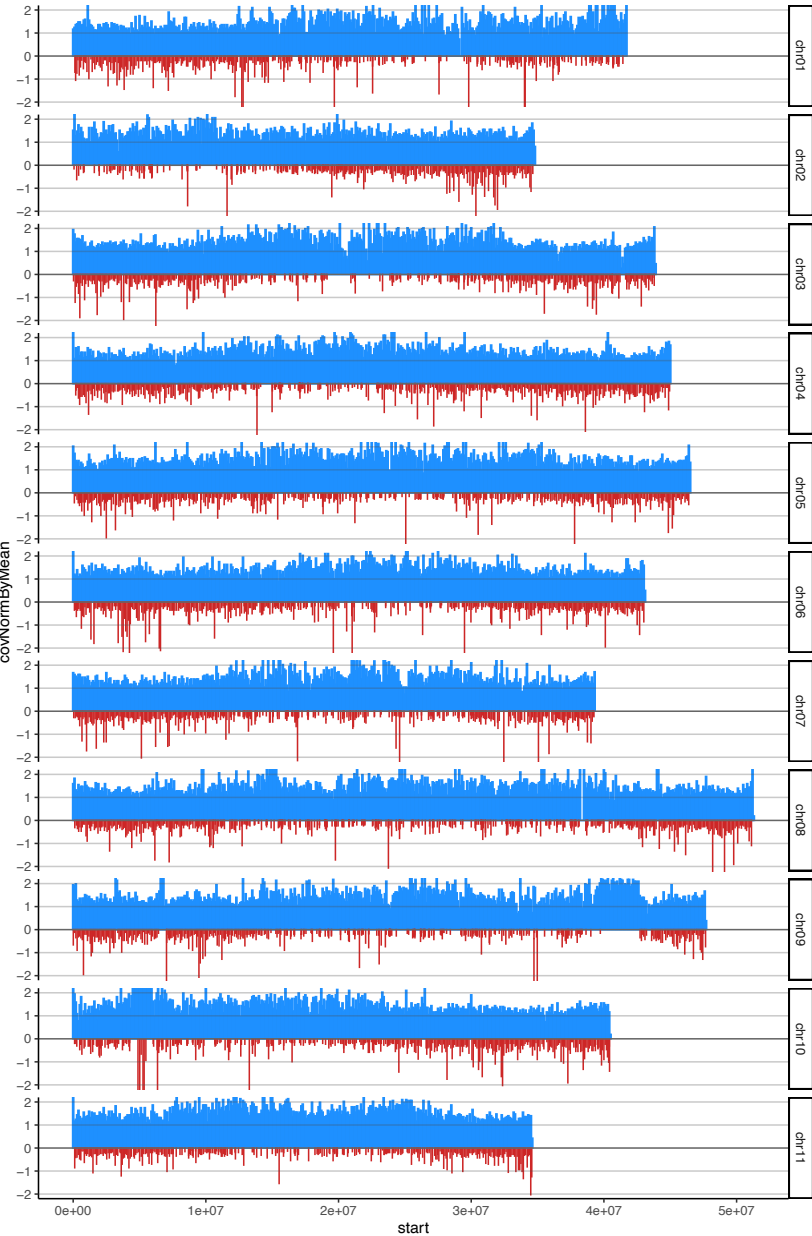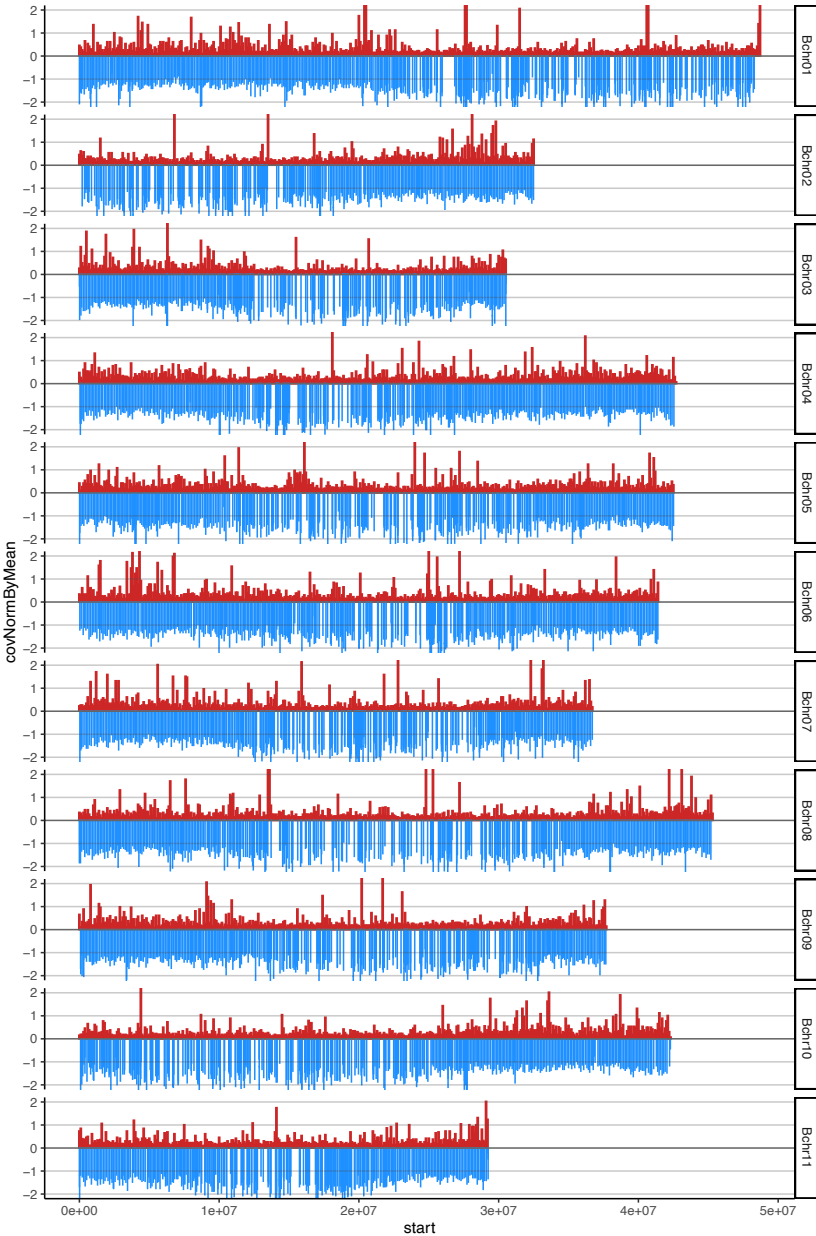

unknown\_AAA

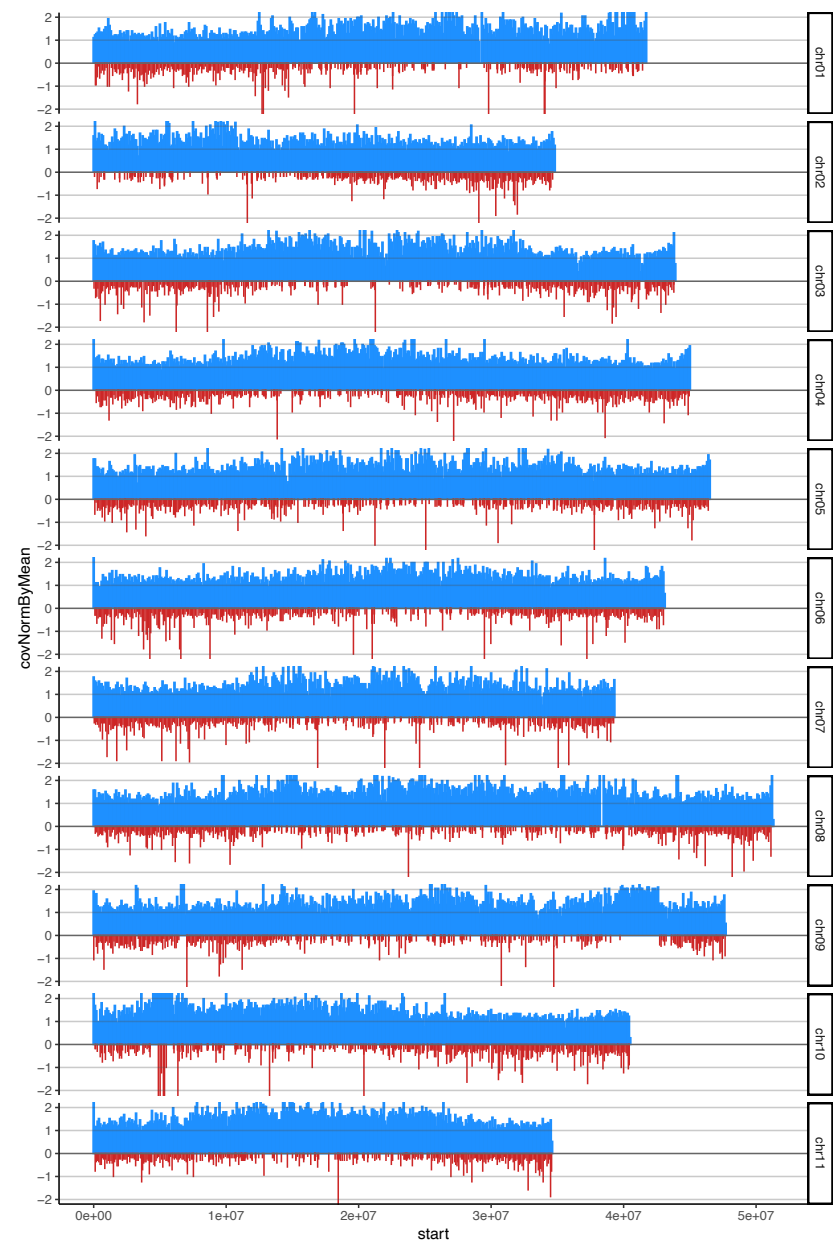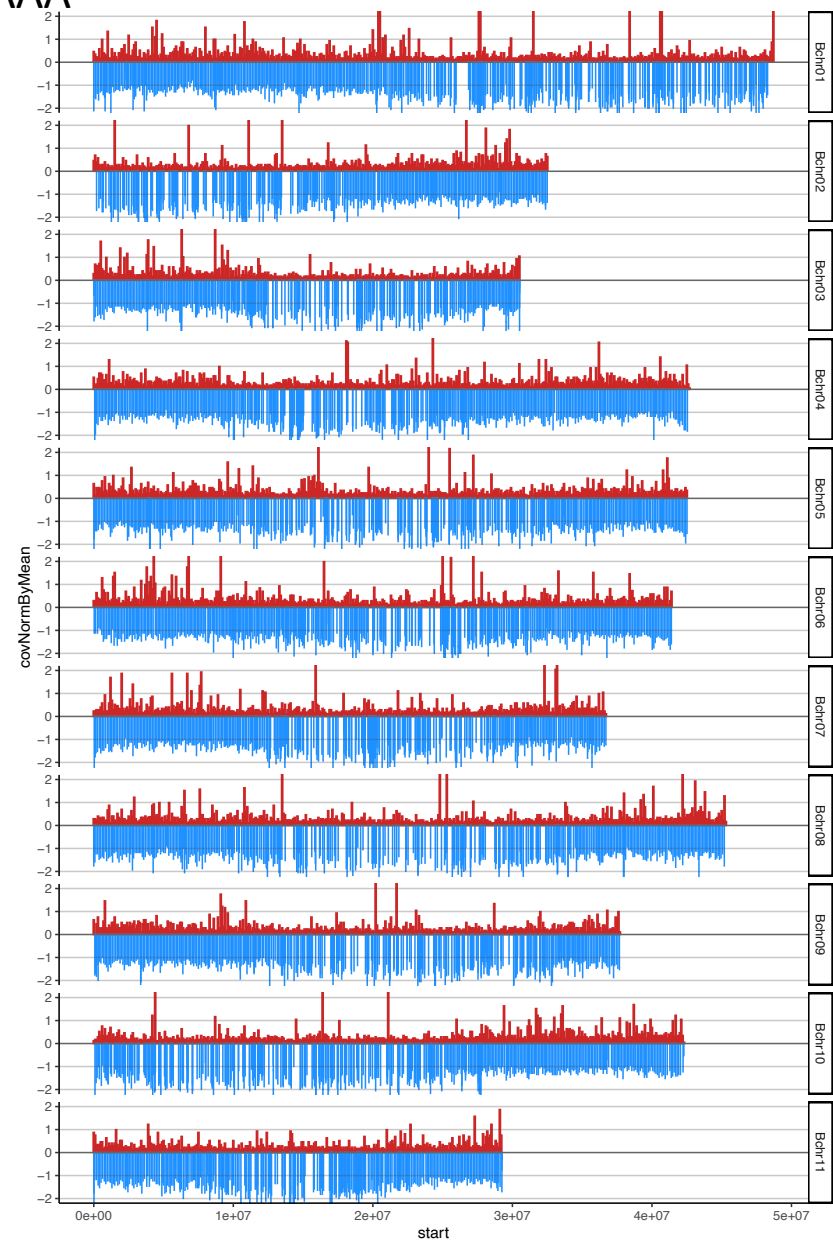

Mutika\_AAA

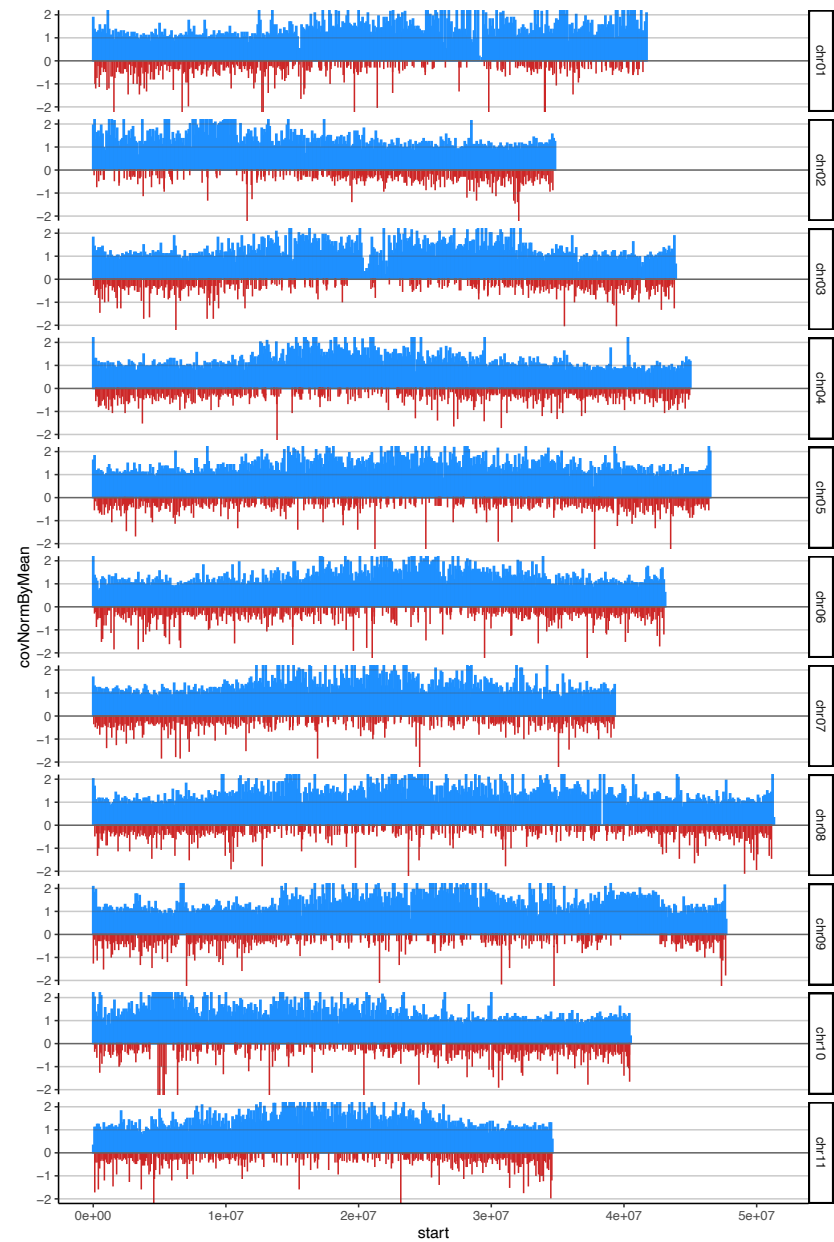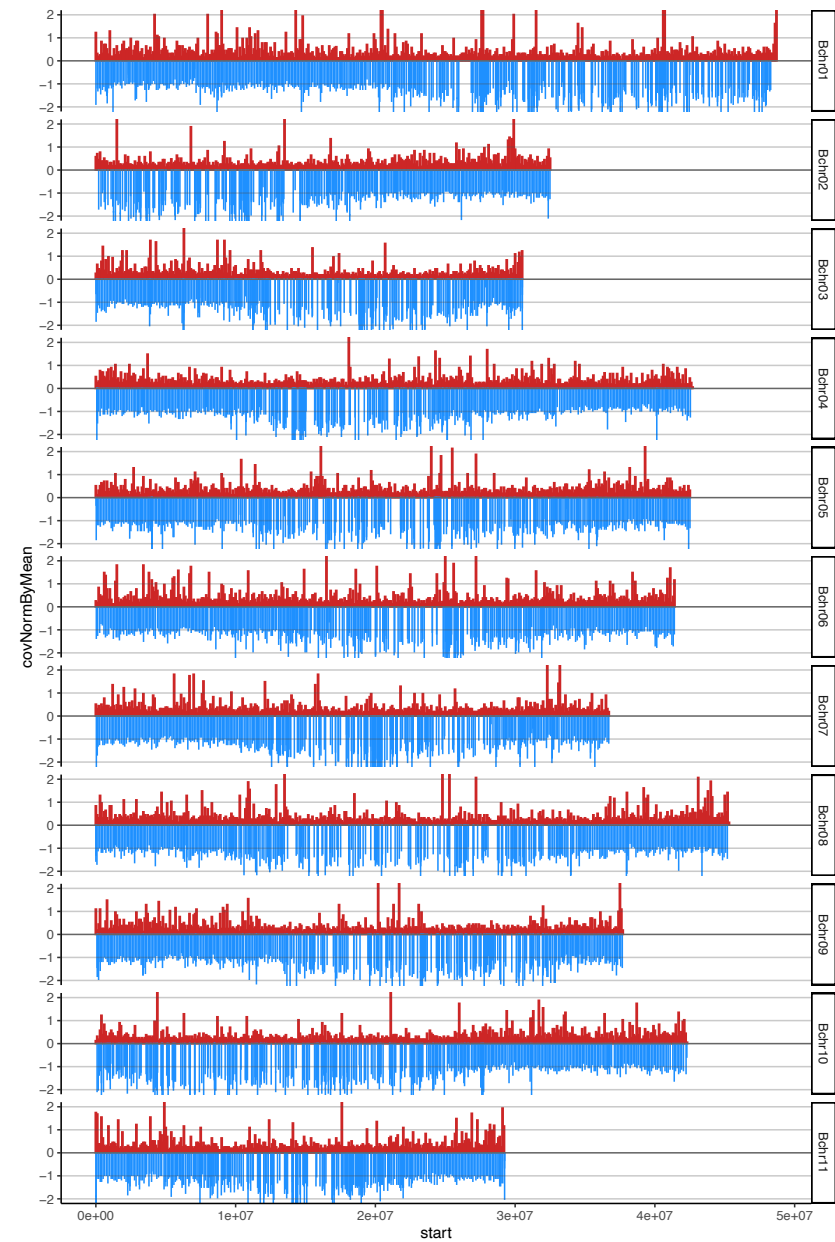

Supplement: mcad192_suppl_Supplementary_File_S4 [file mcad192_suppl_supplementary_file_s4.pdf]

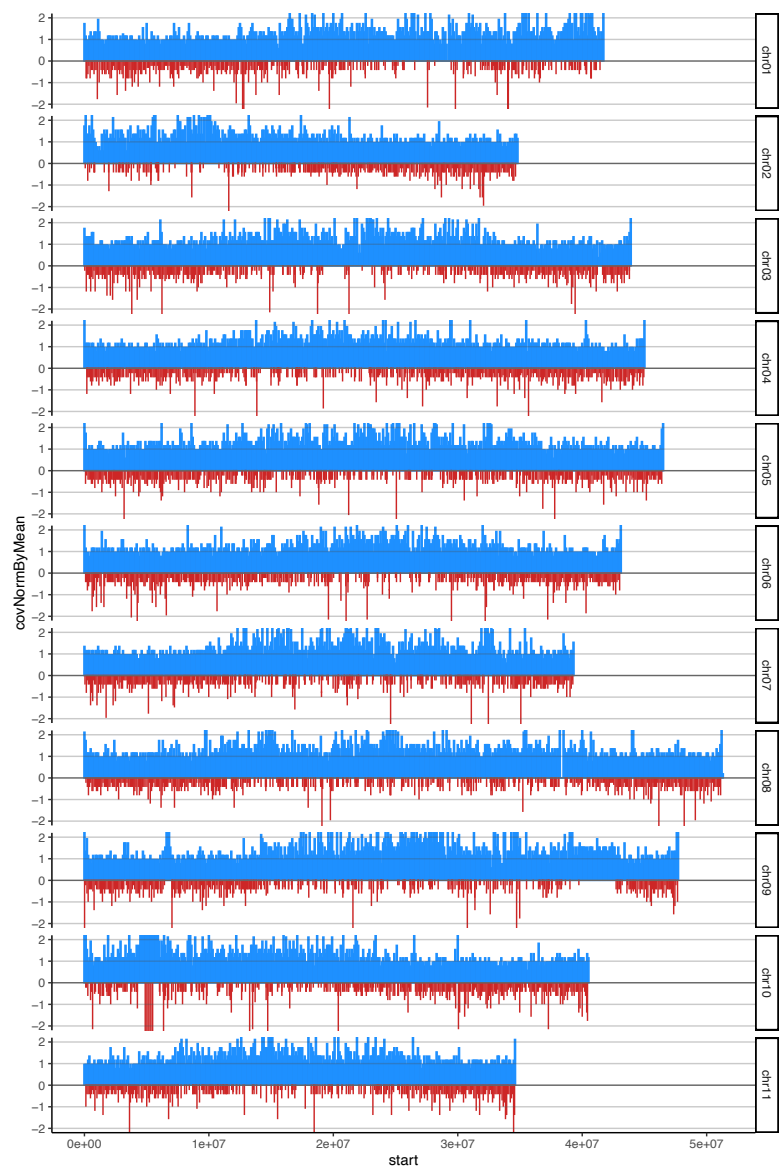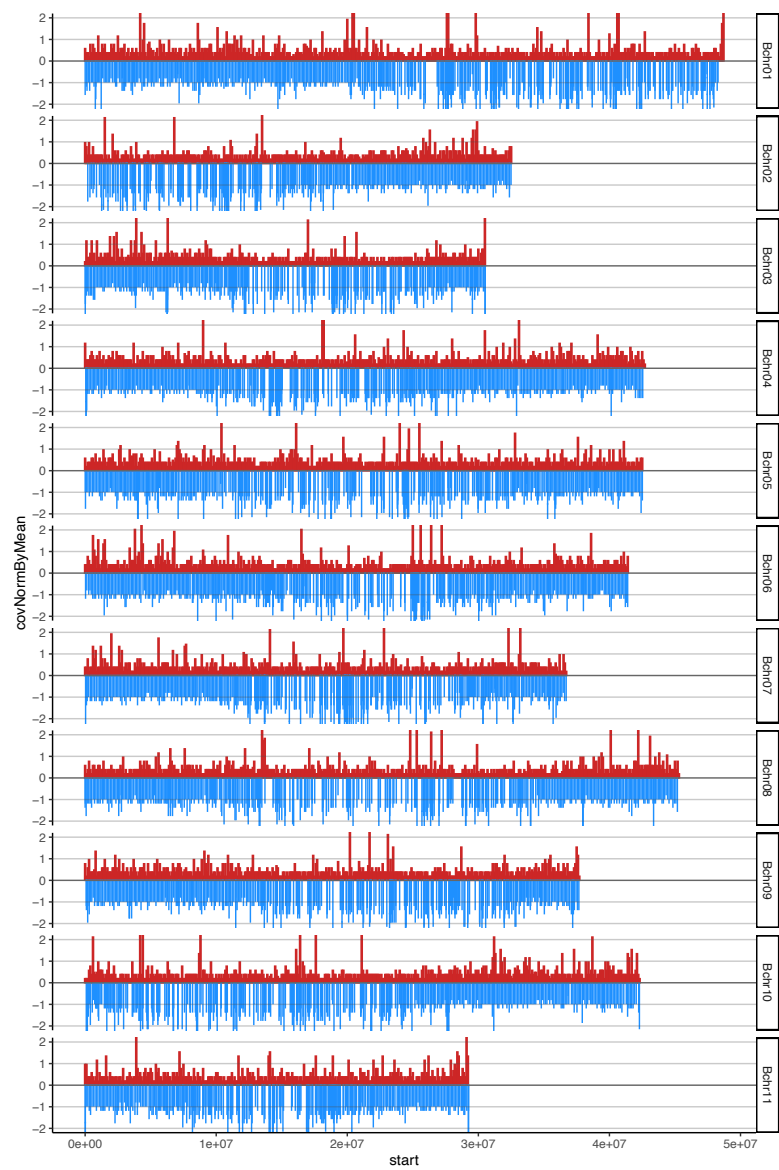

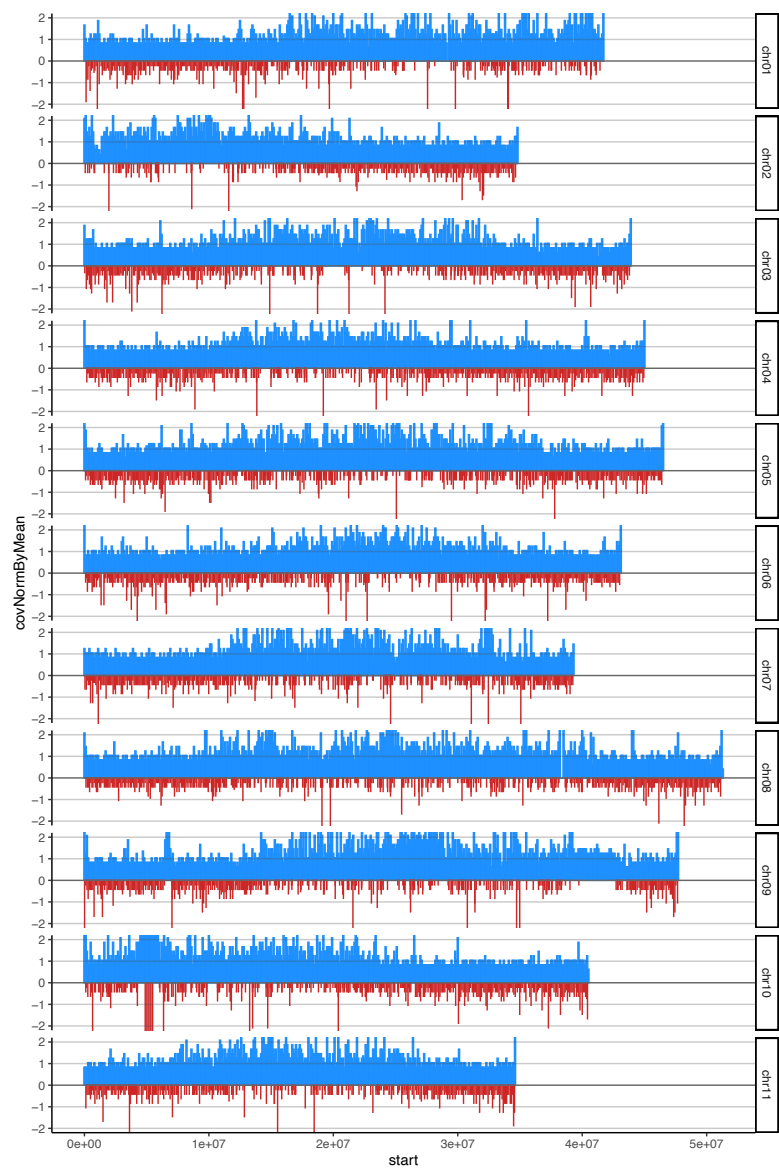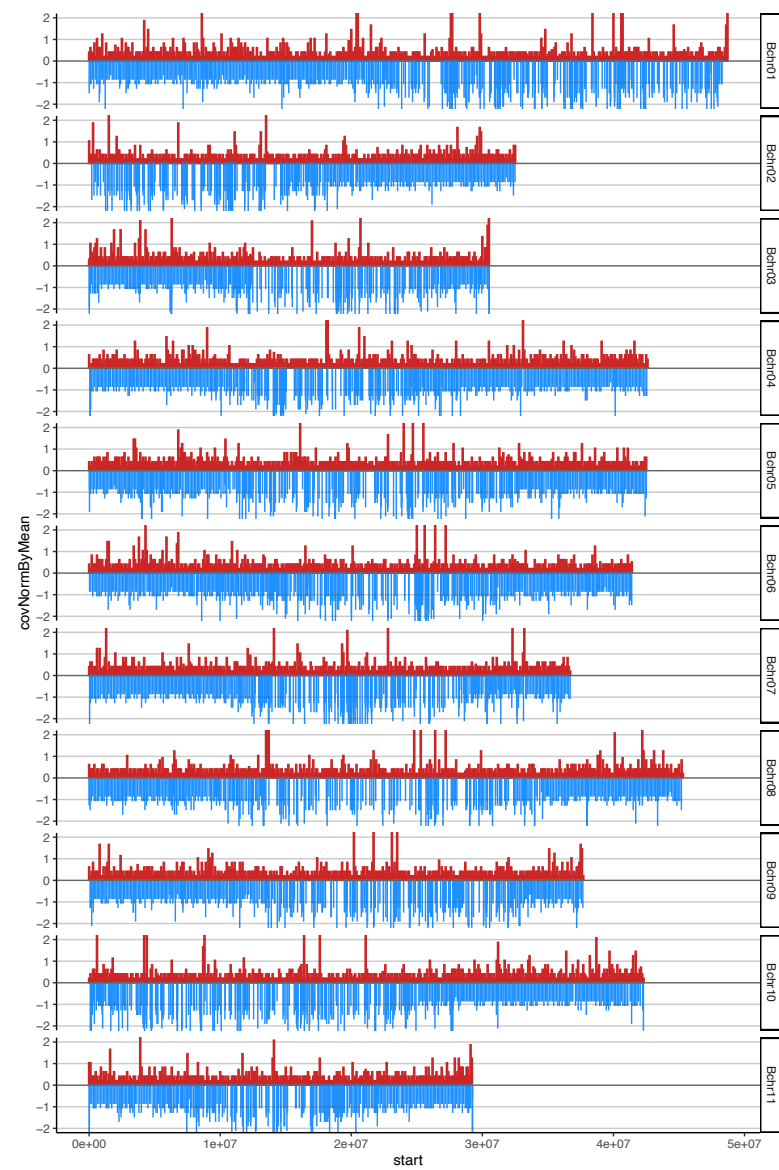

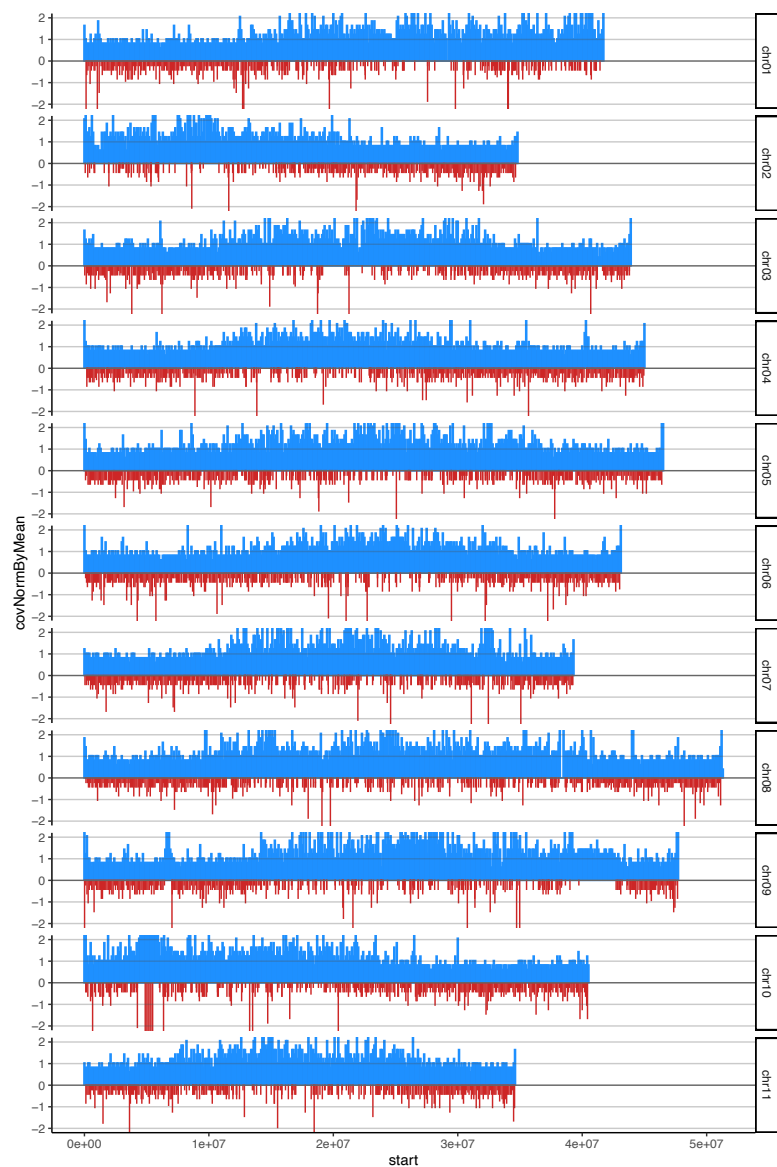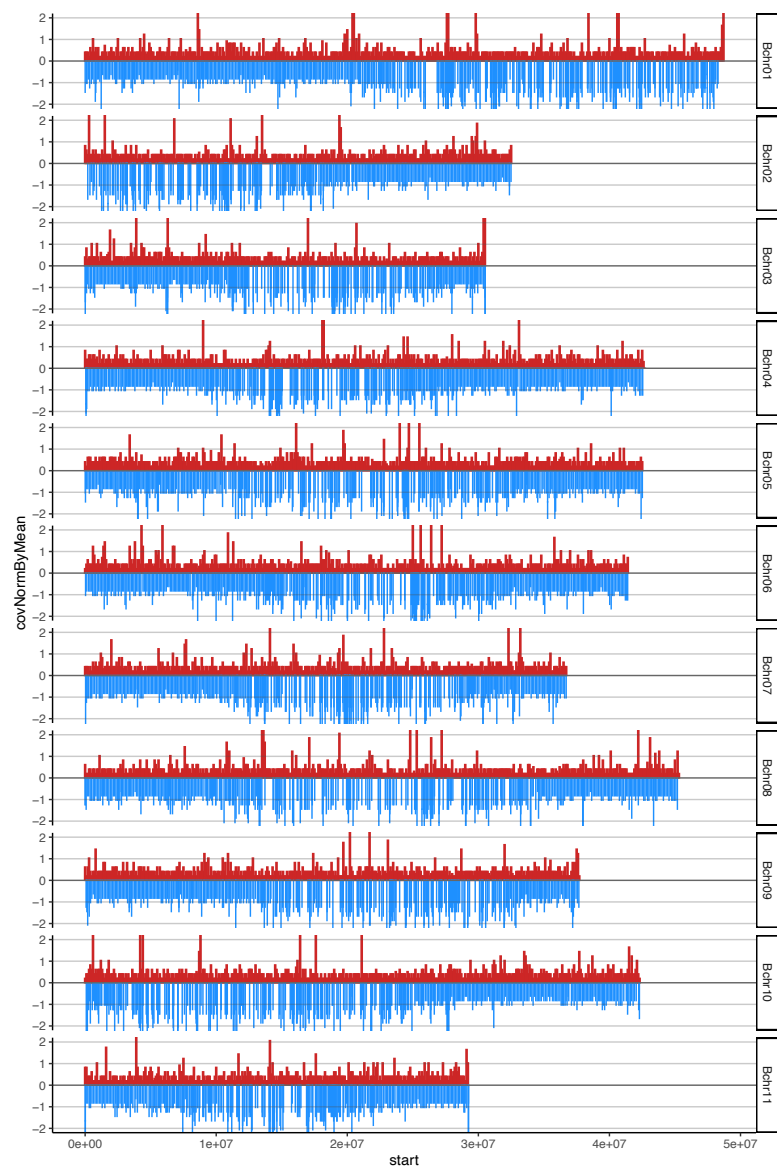

100 LACATAN

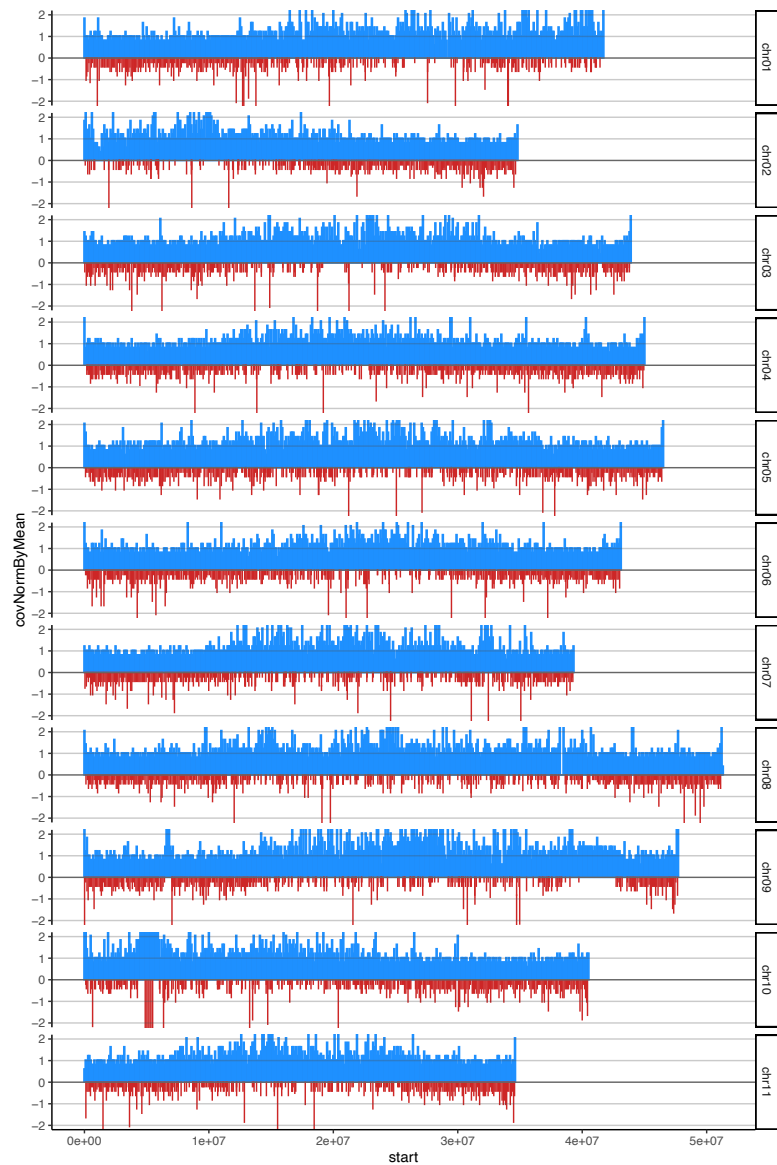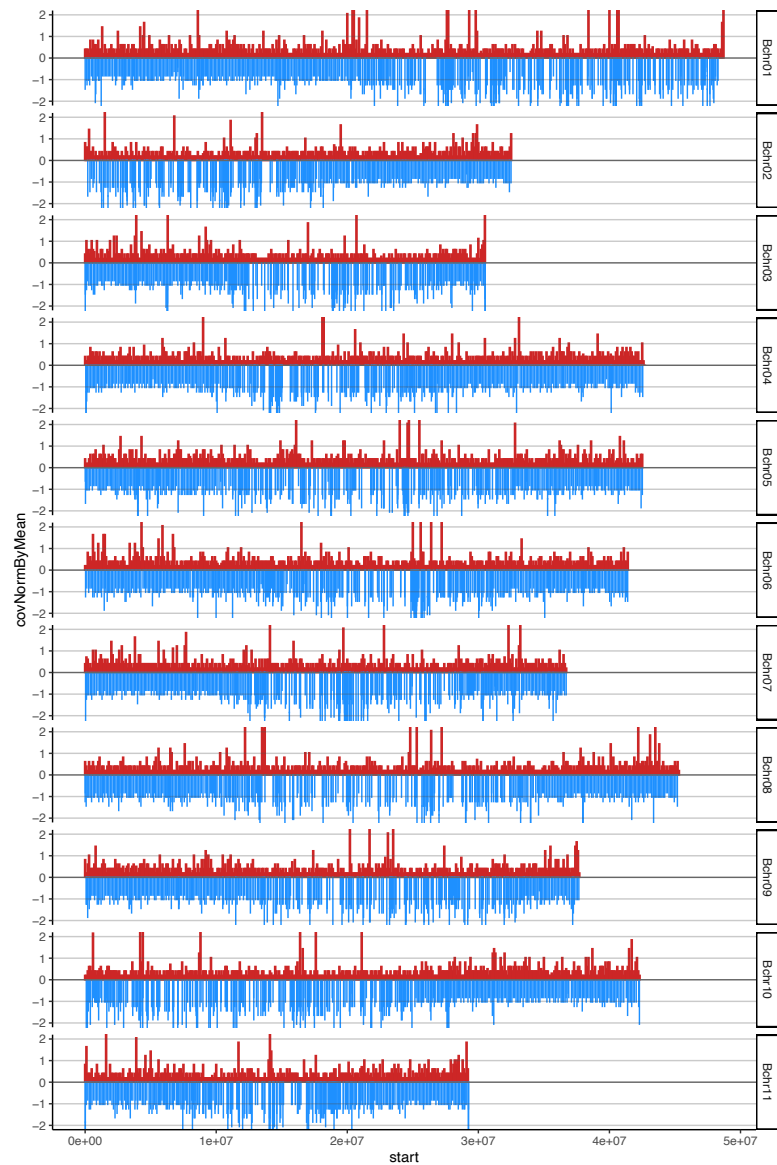

101 SEREDOW

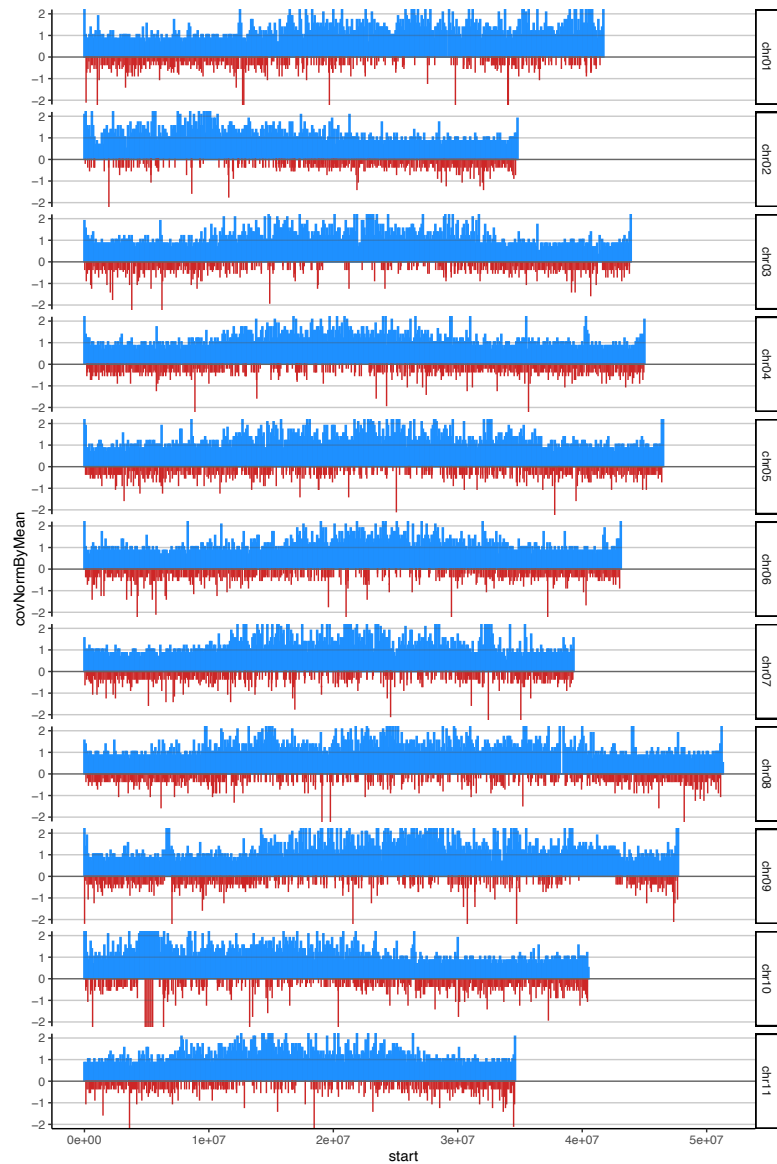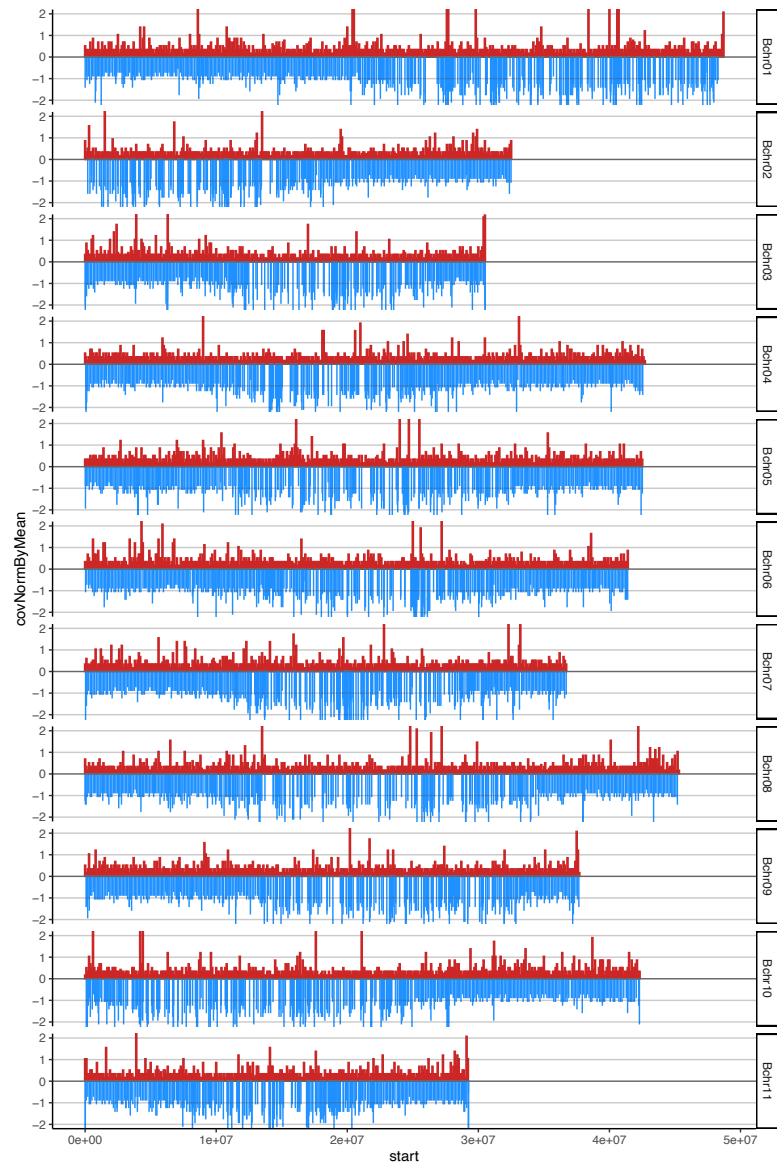

102 GRANENANO

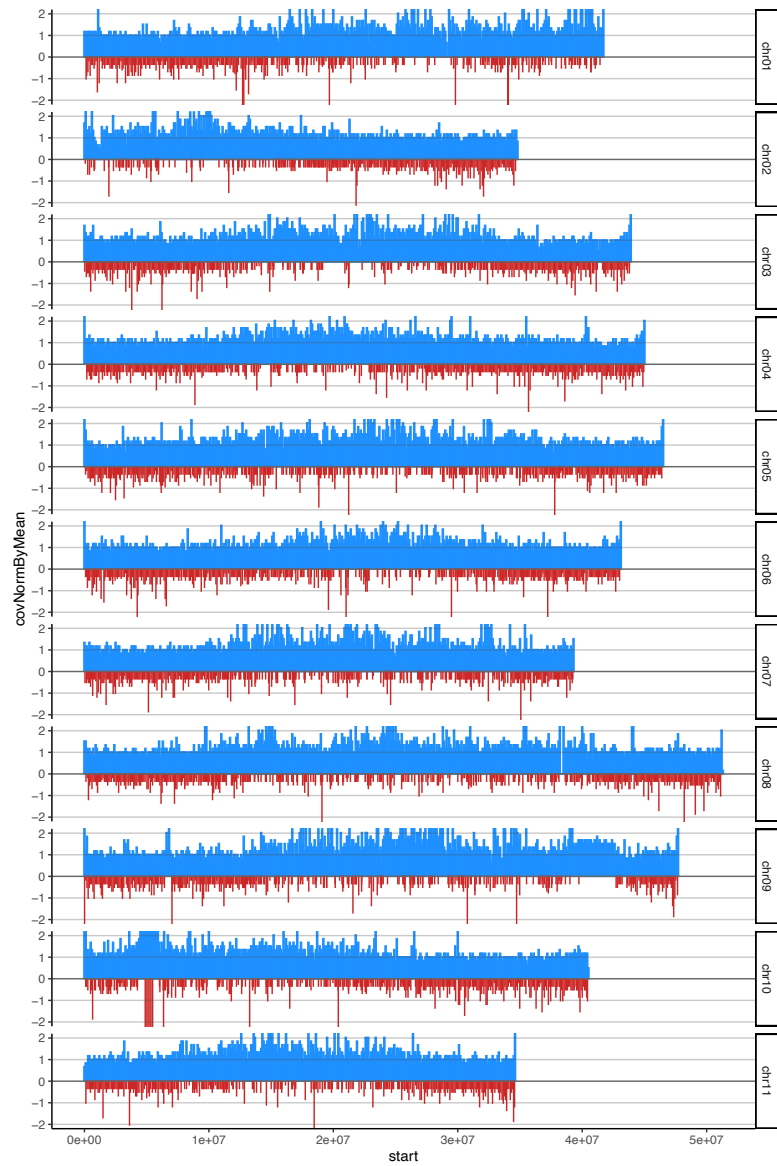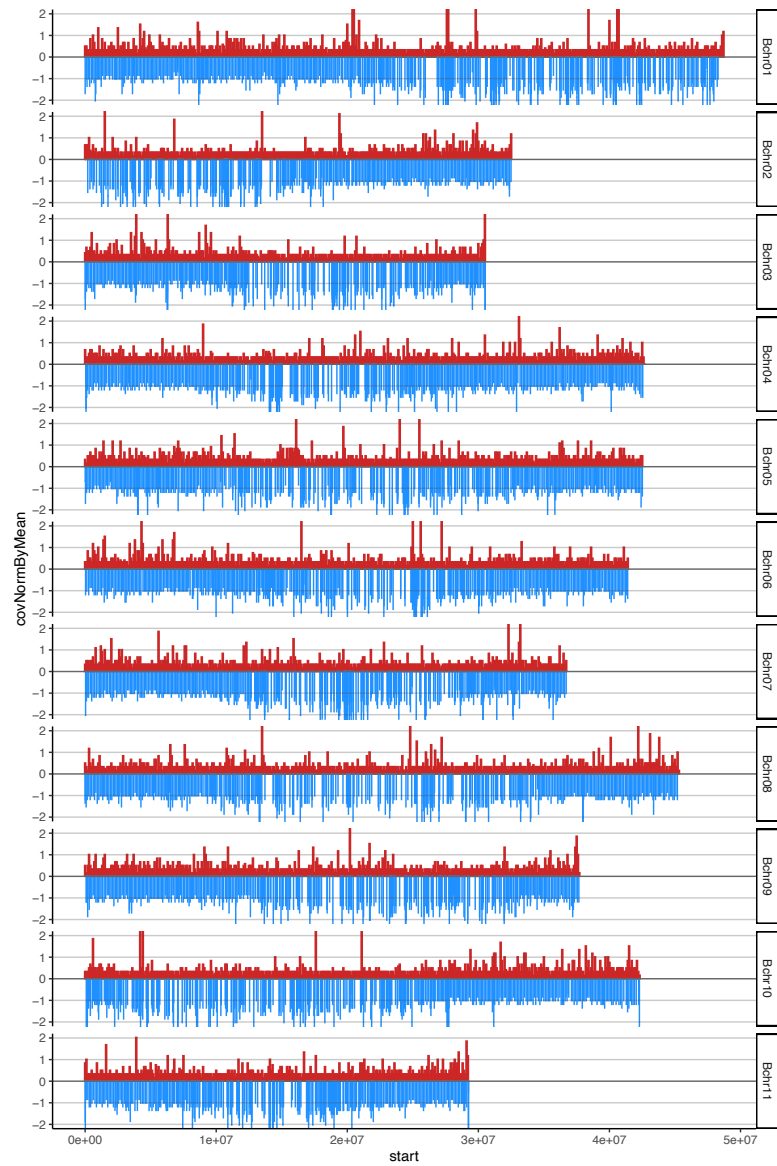

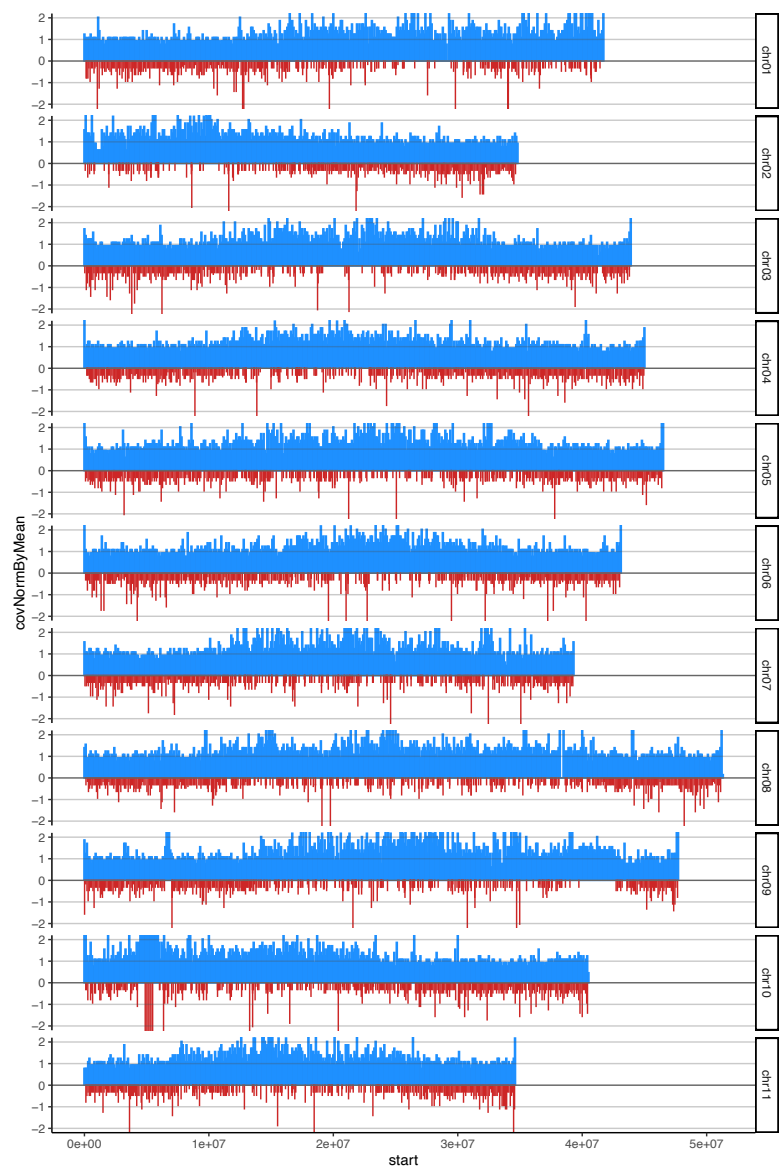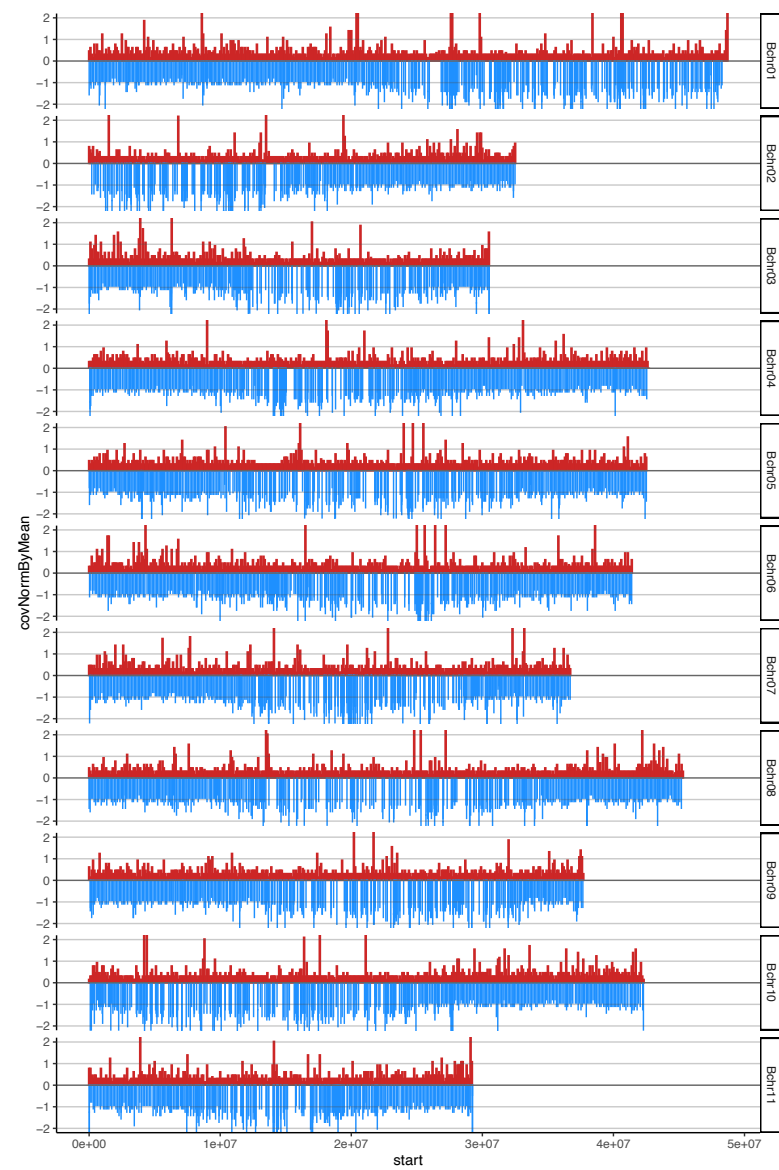

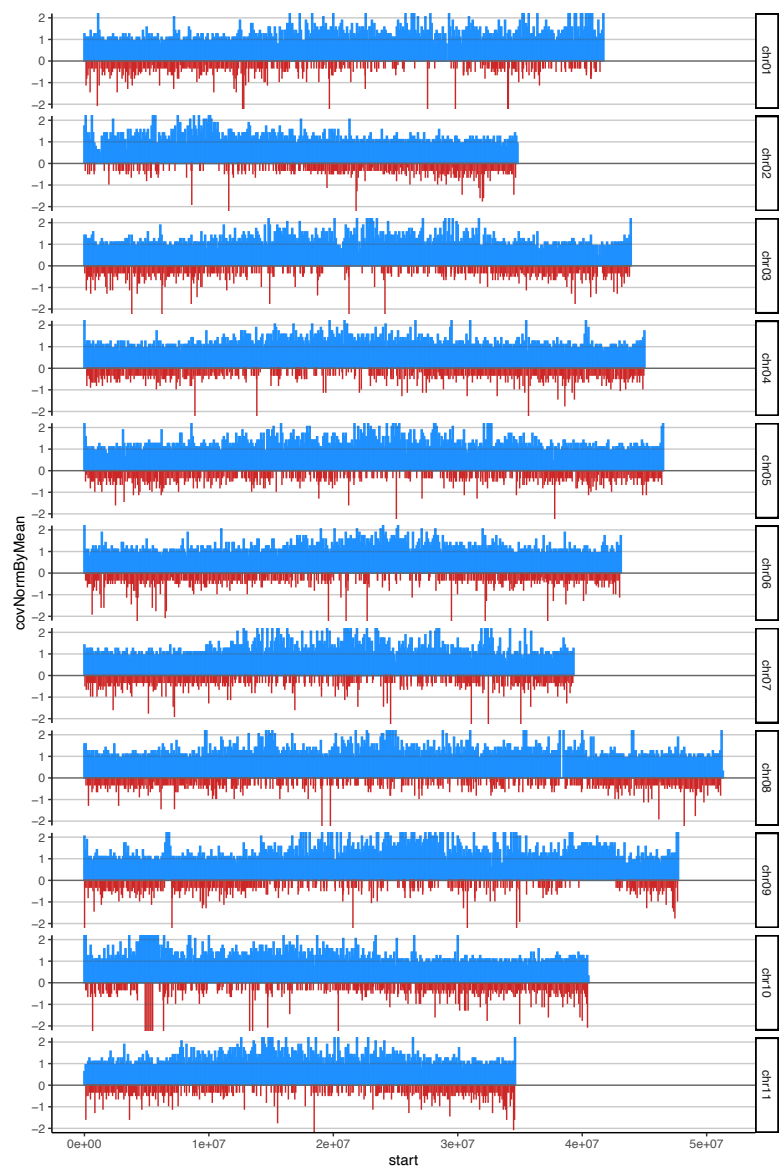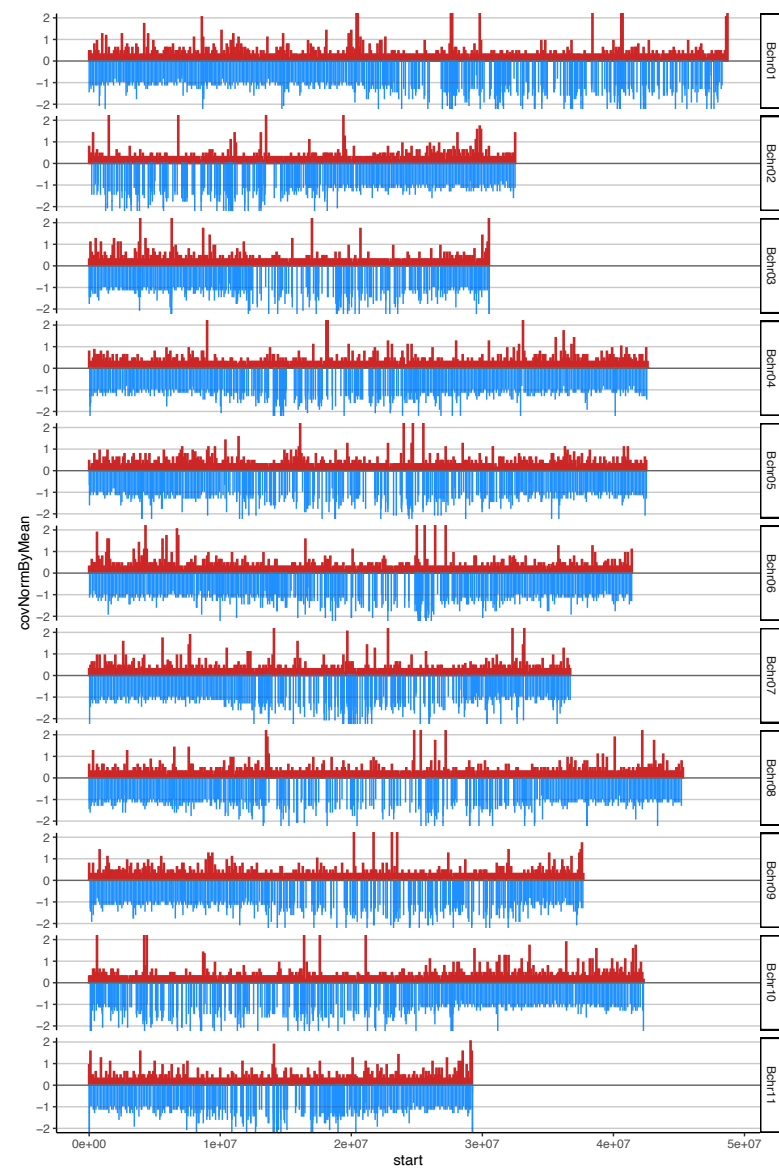

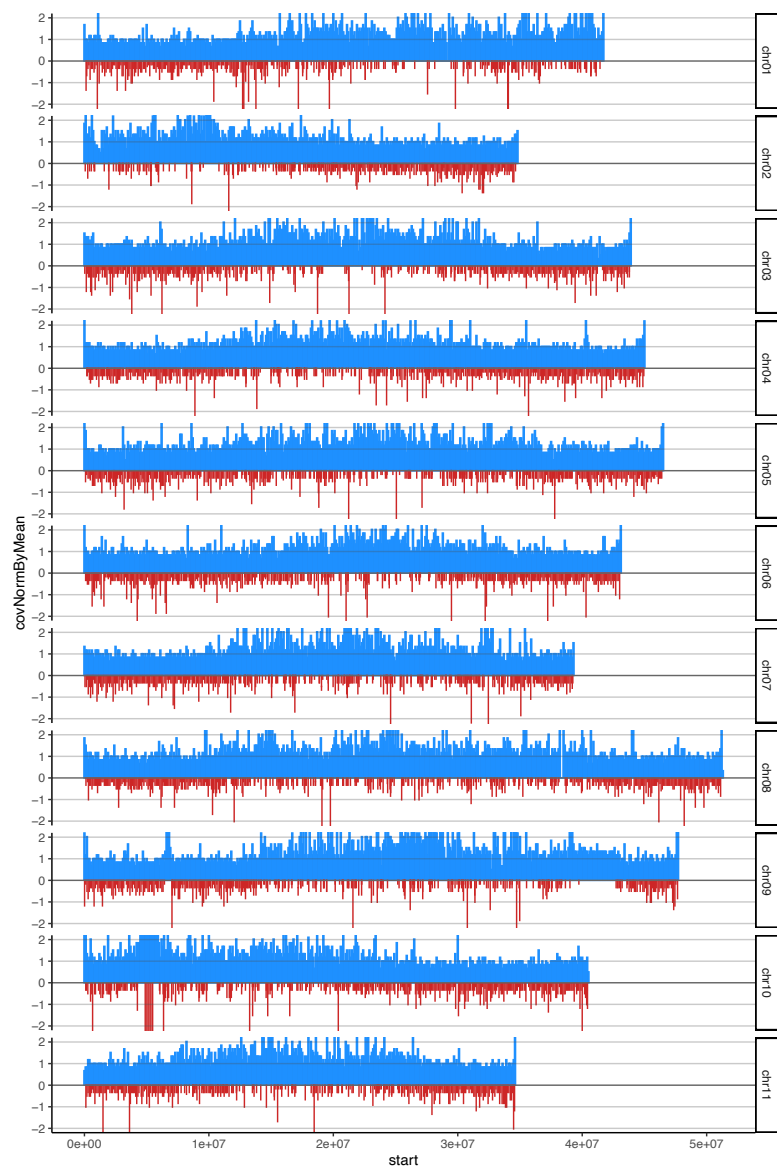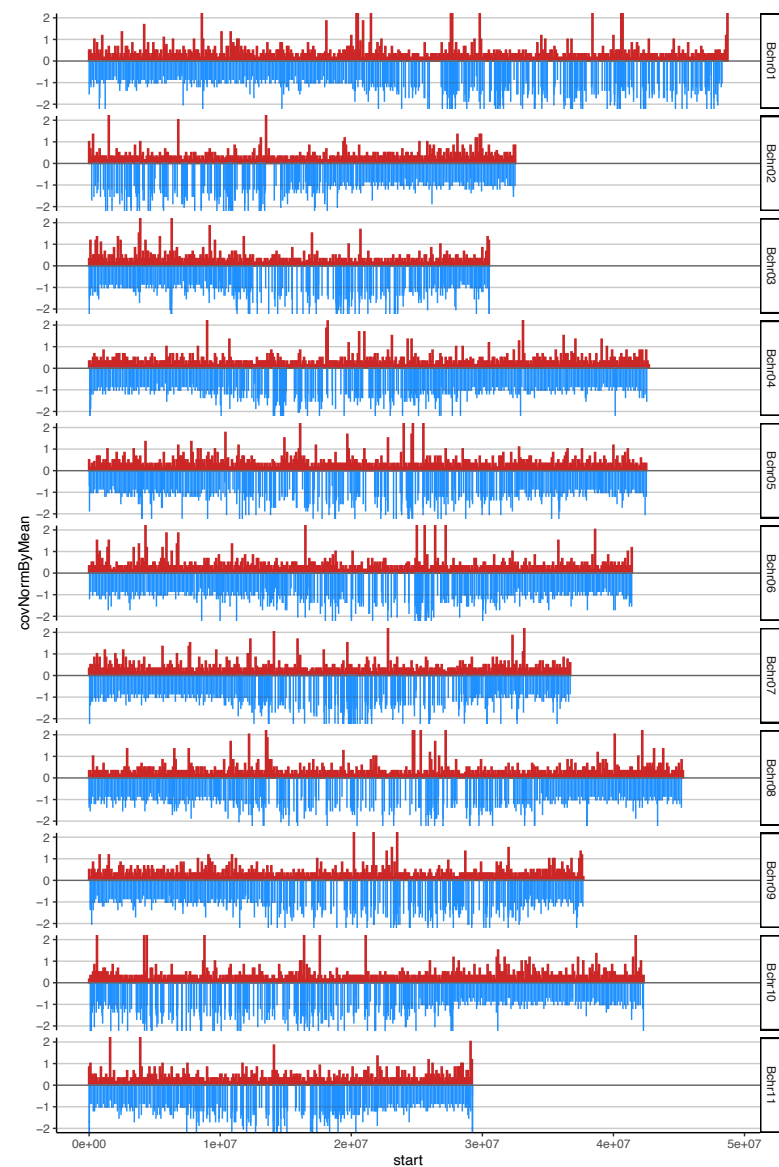

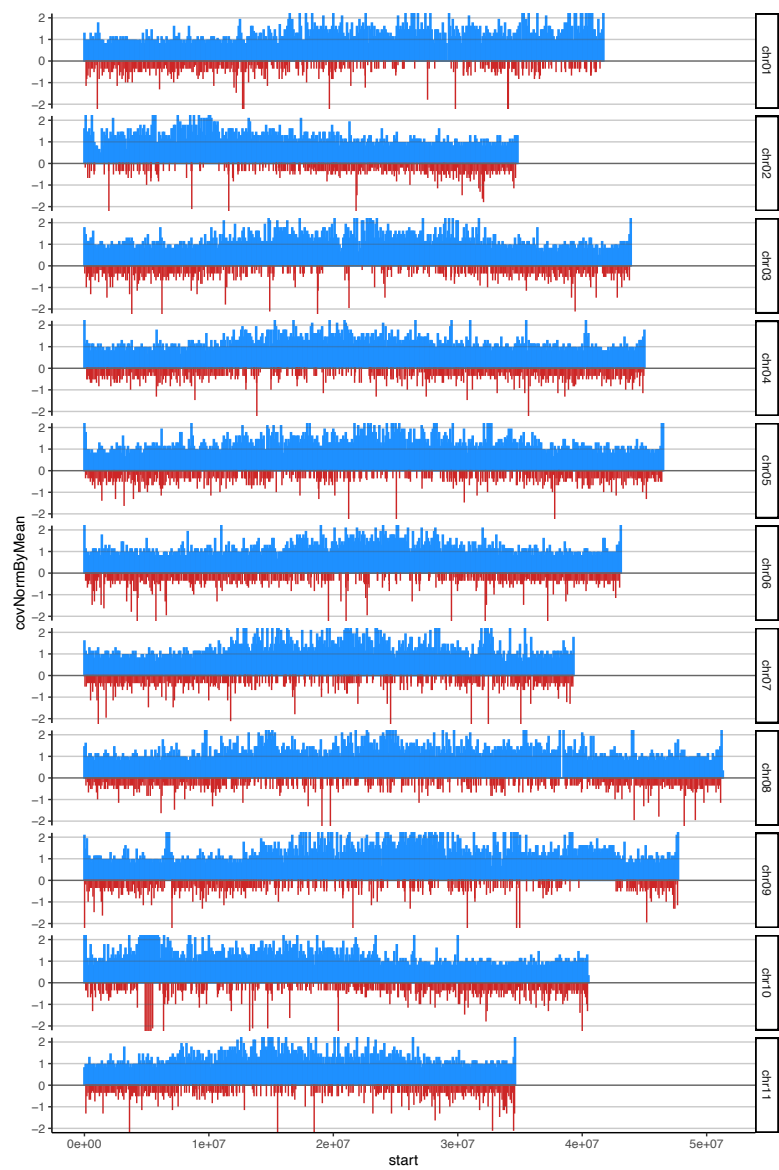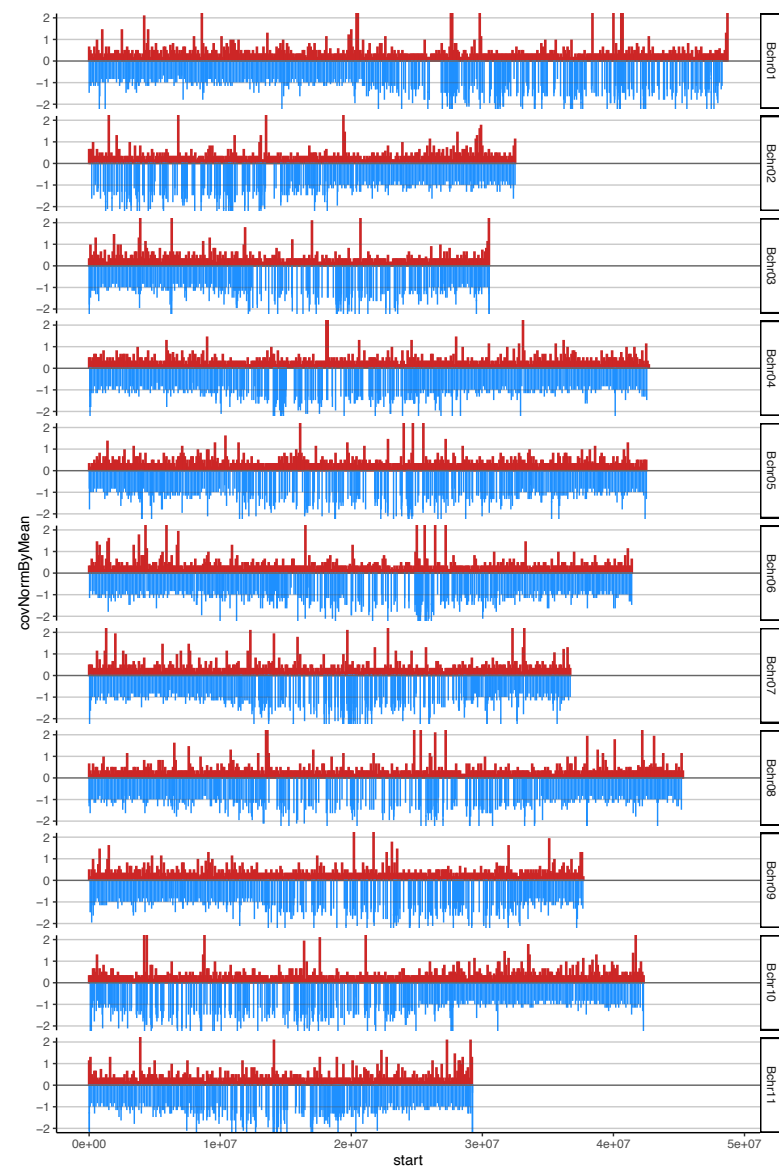

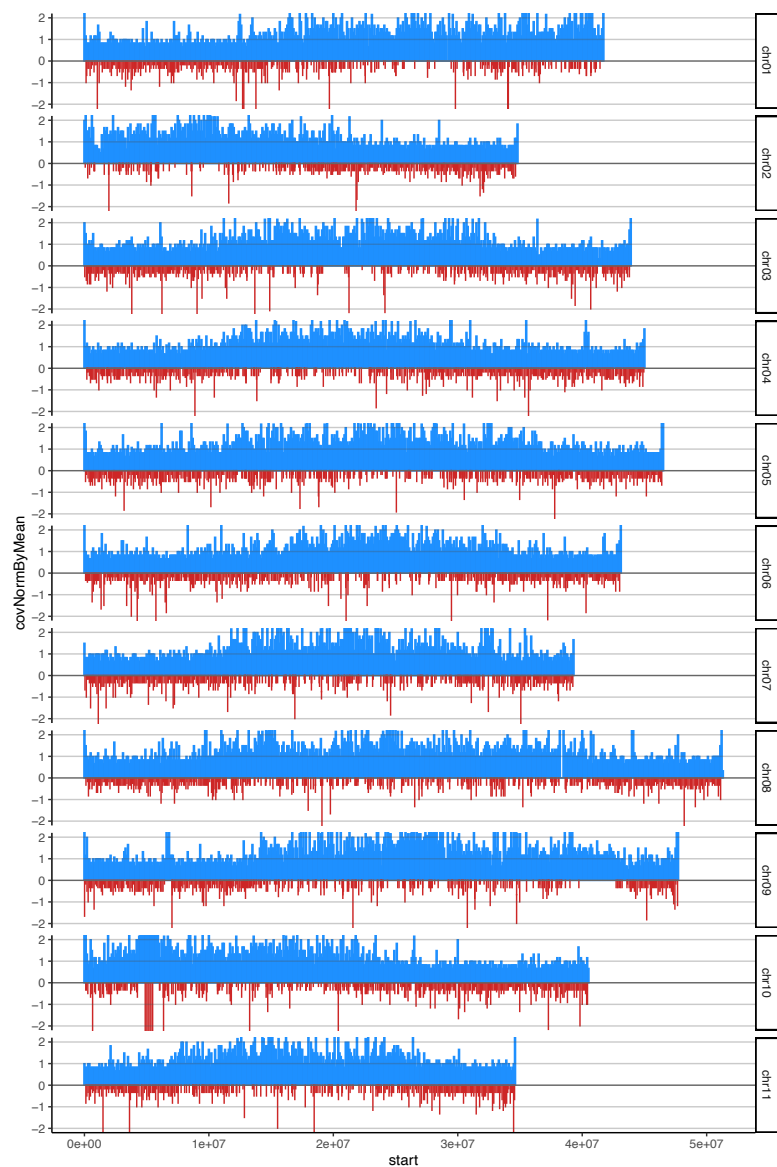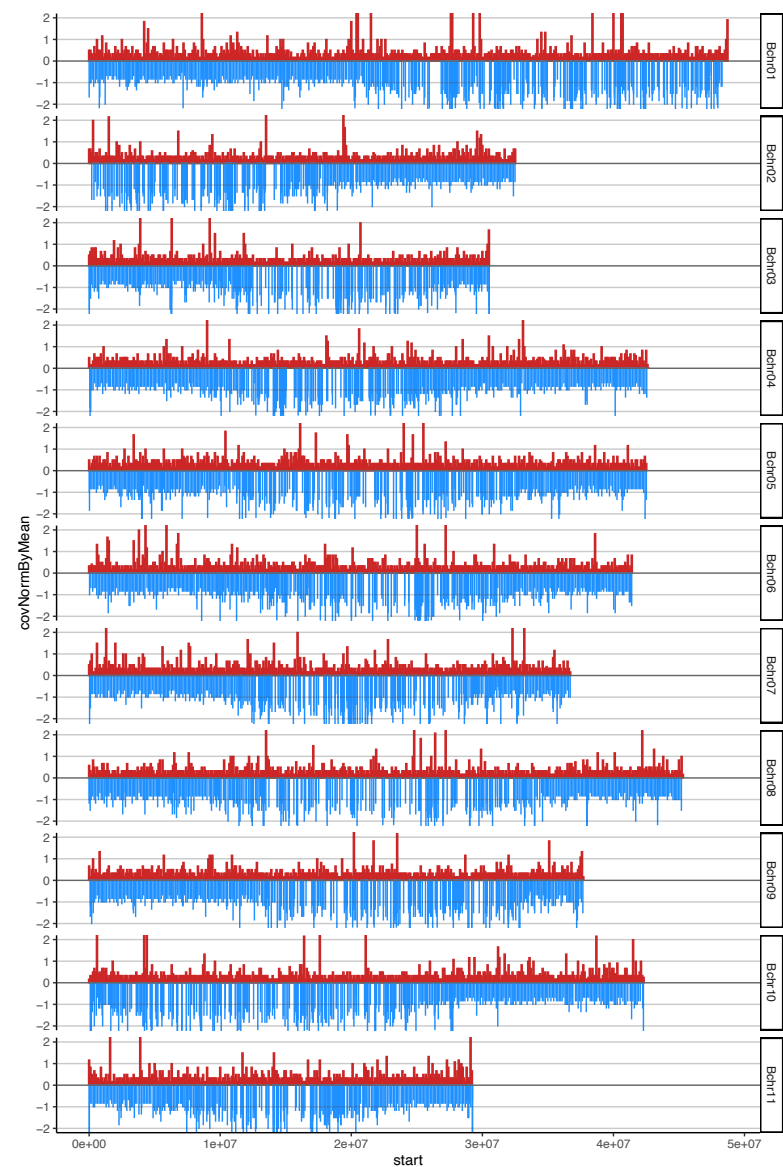

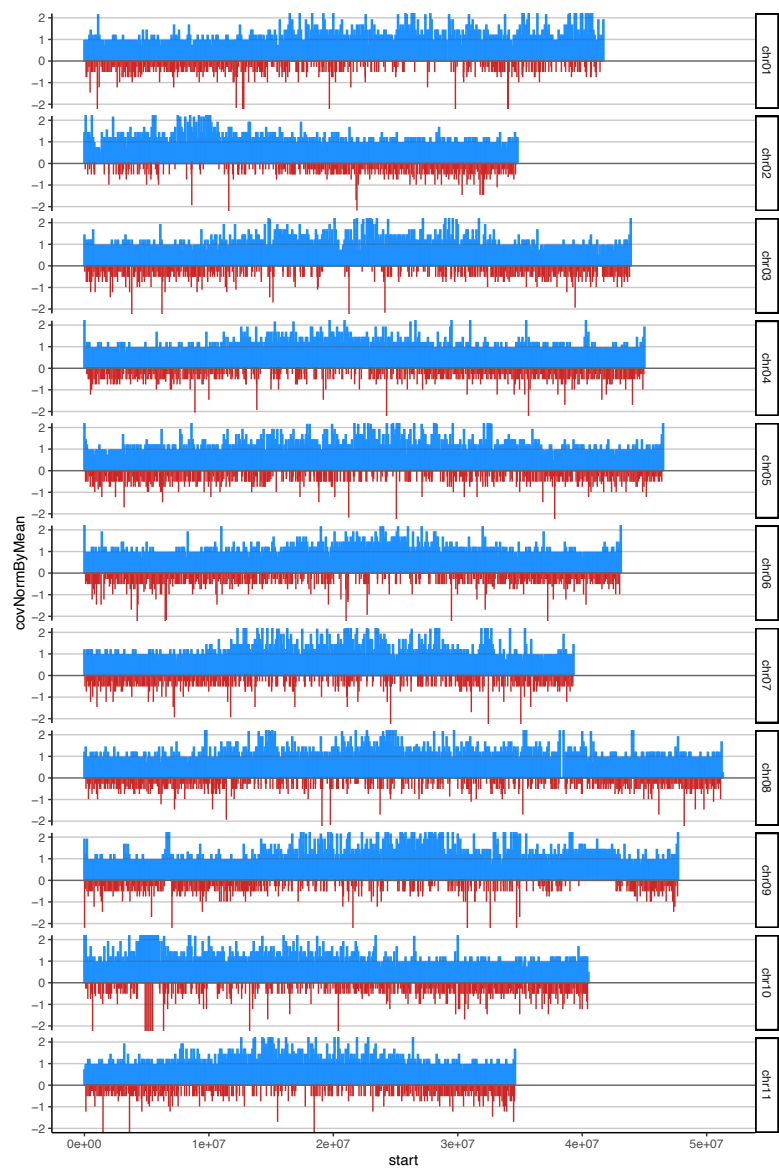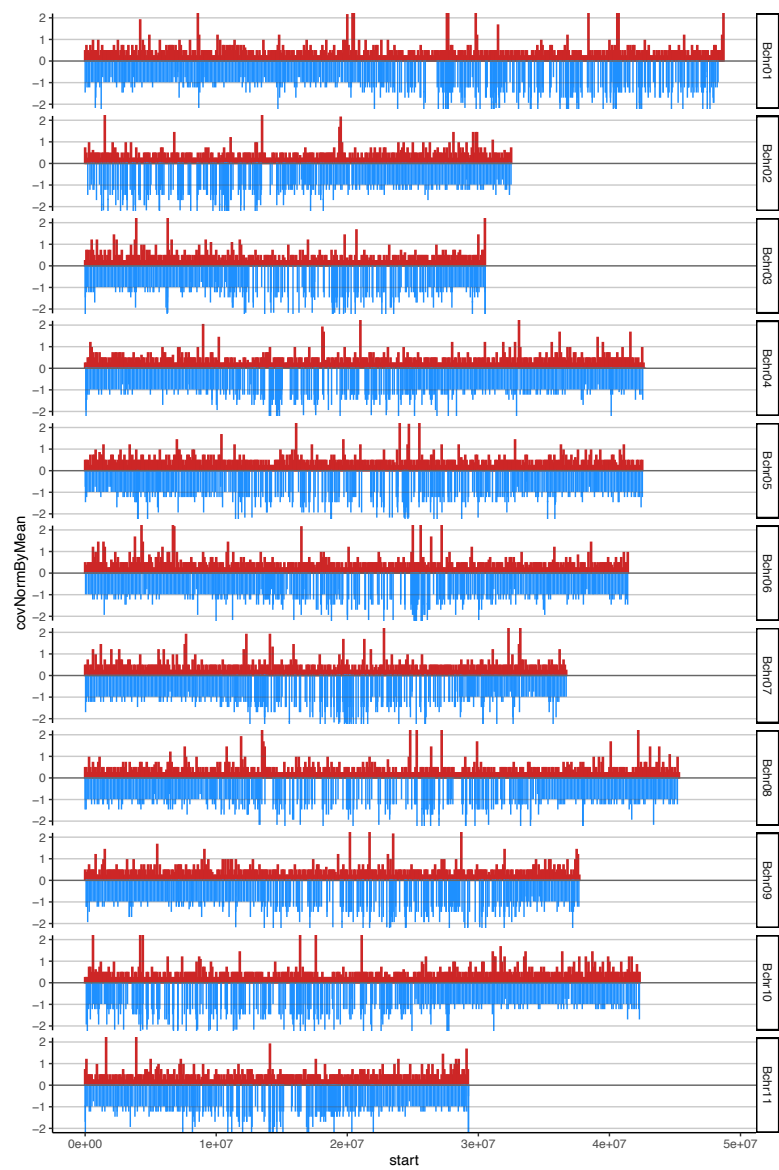

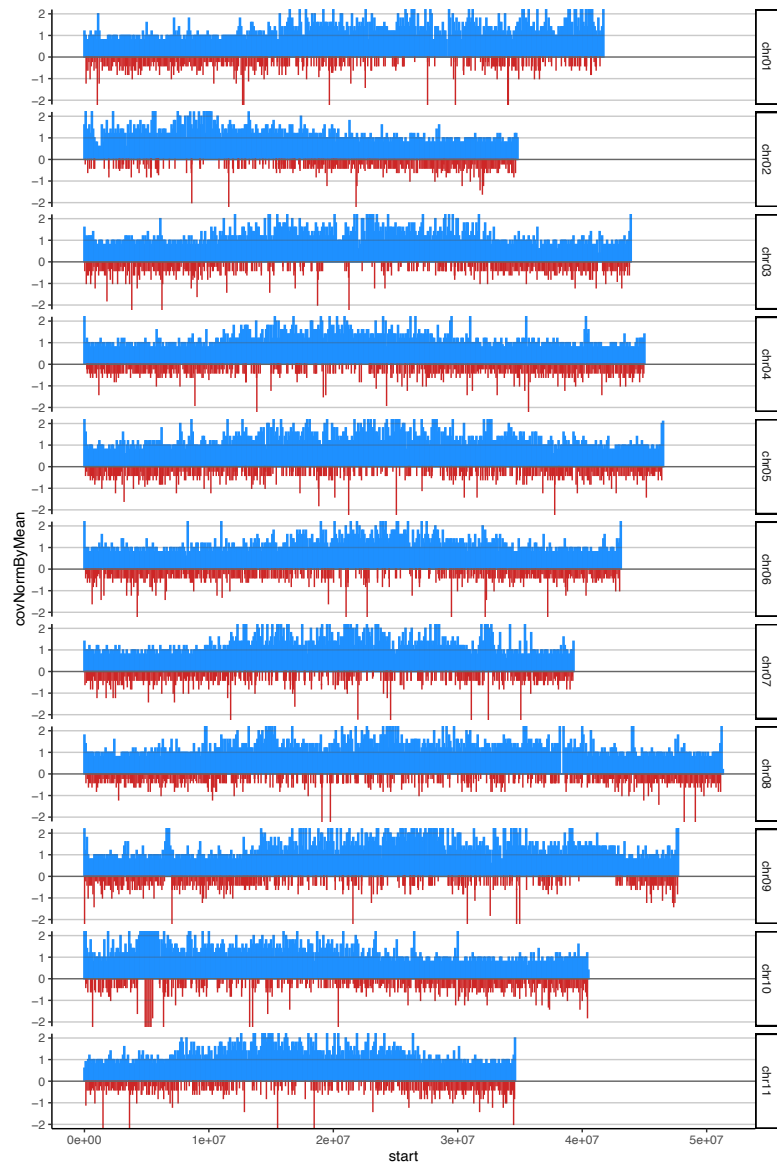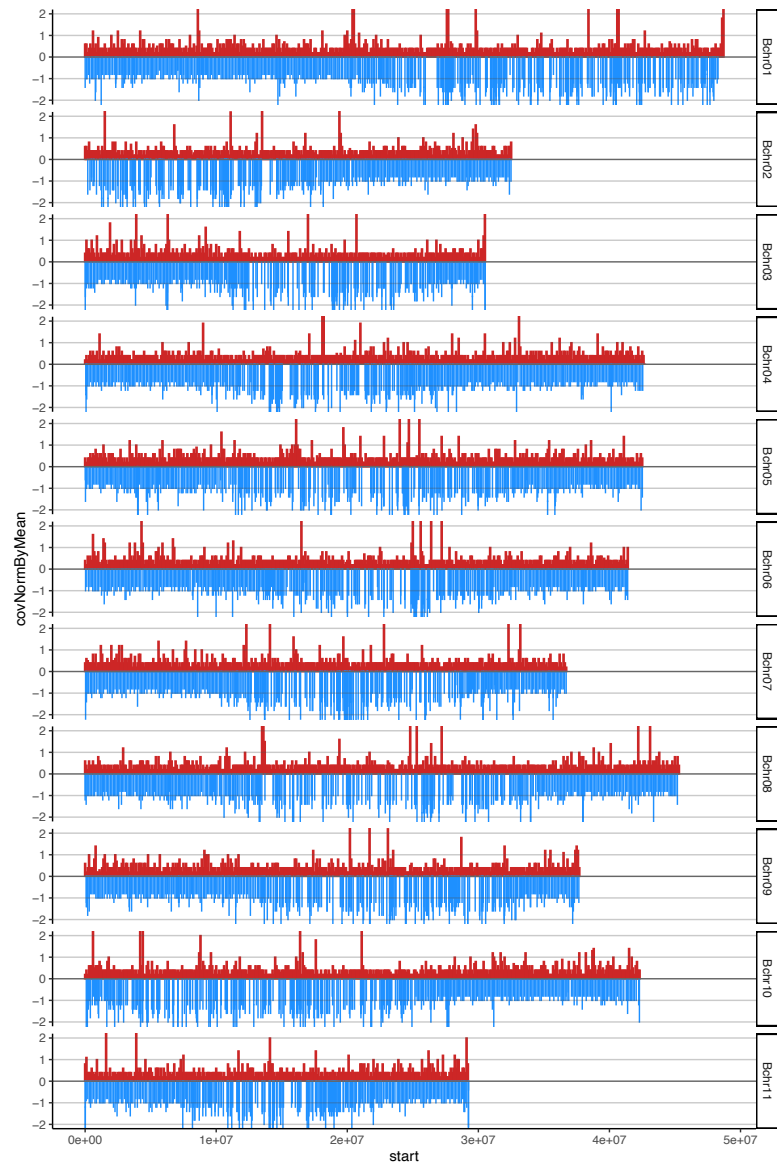

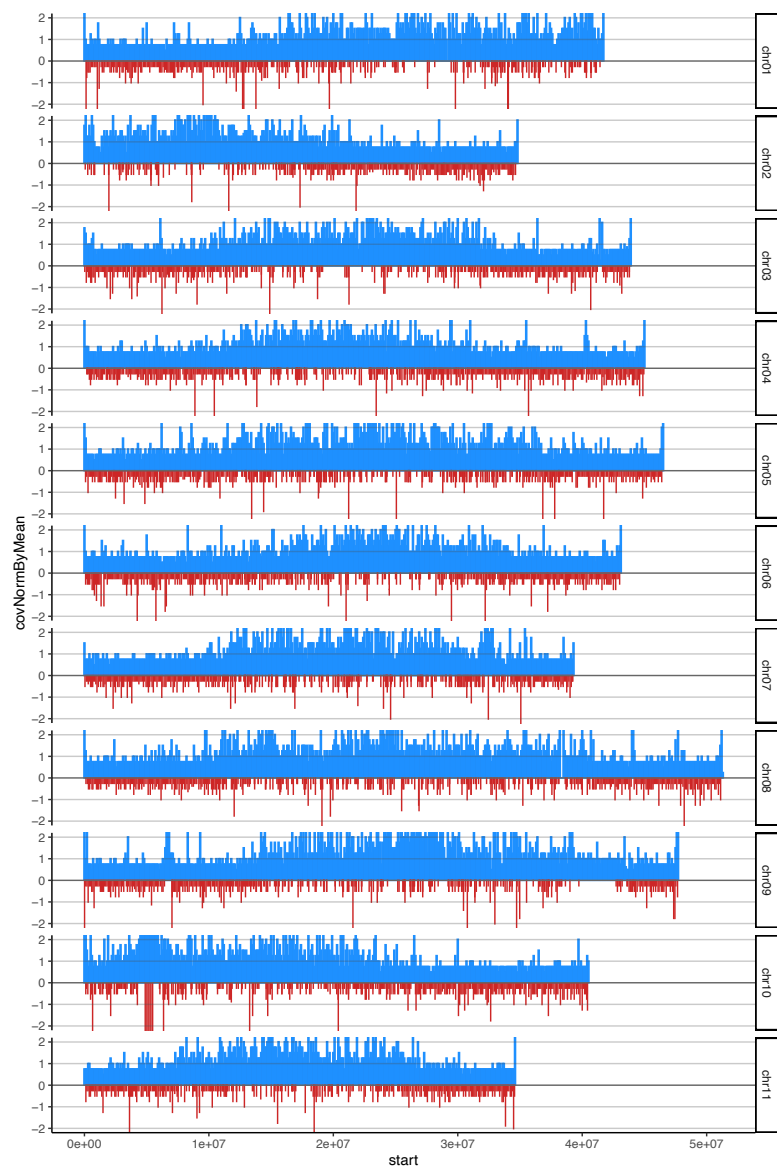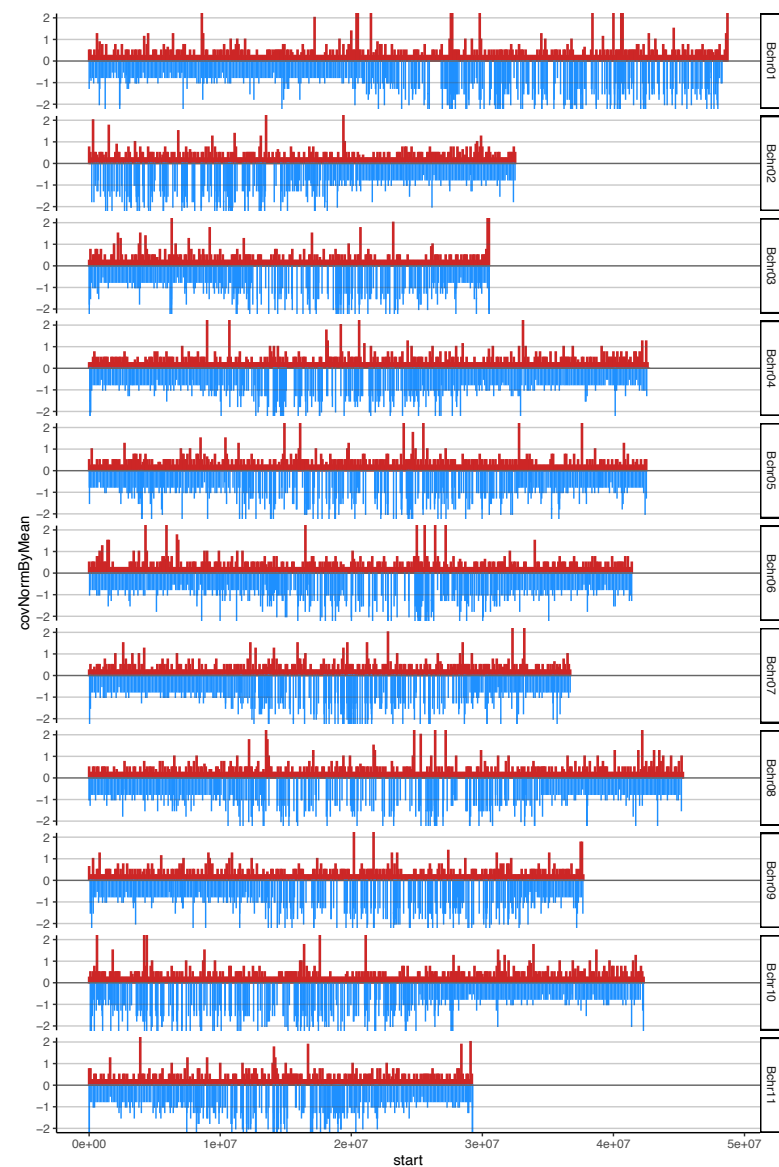

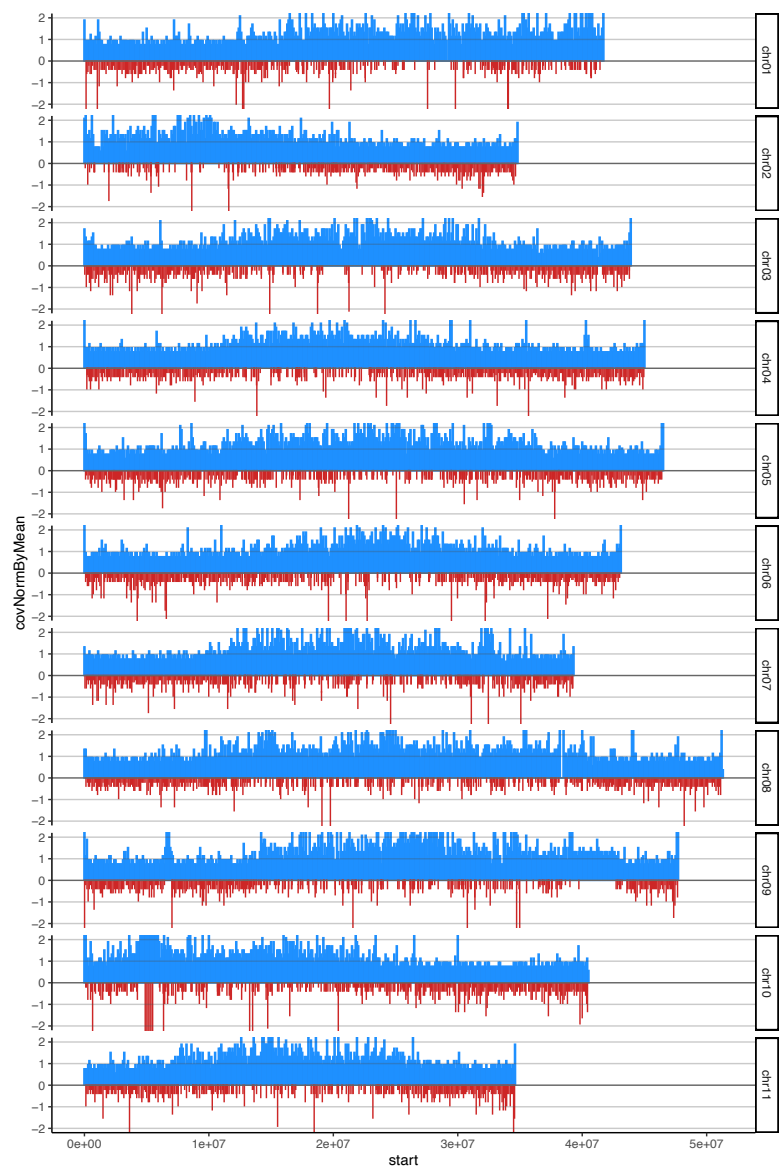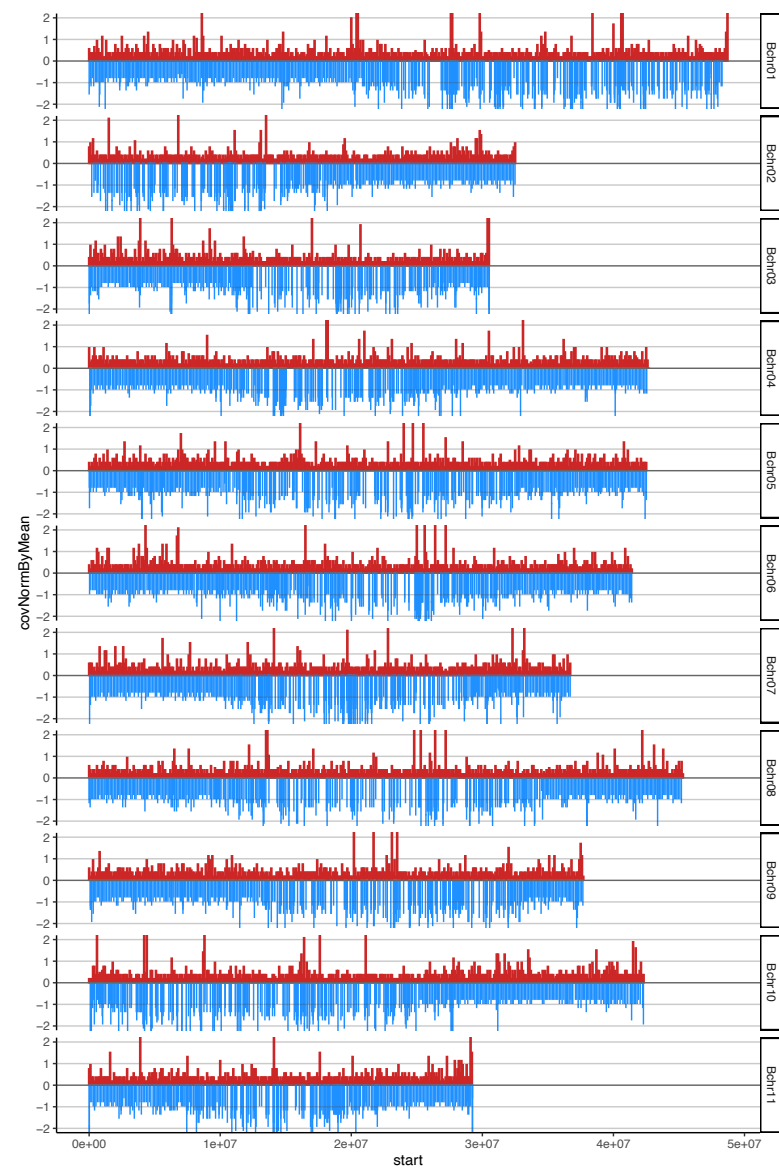

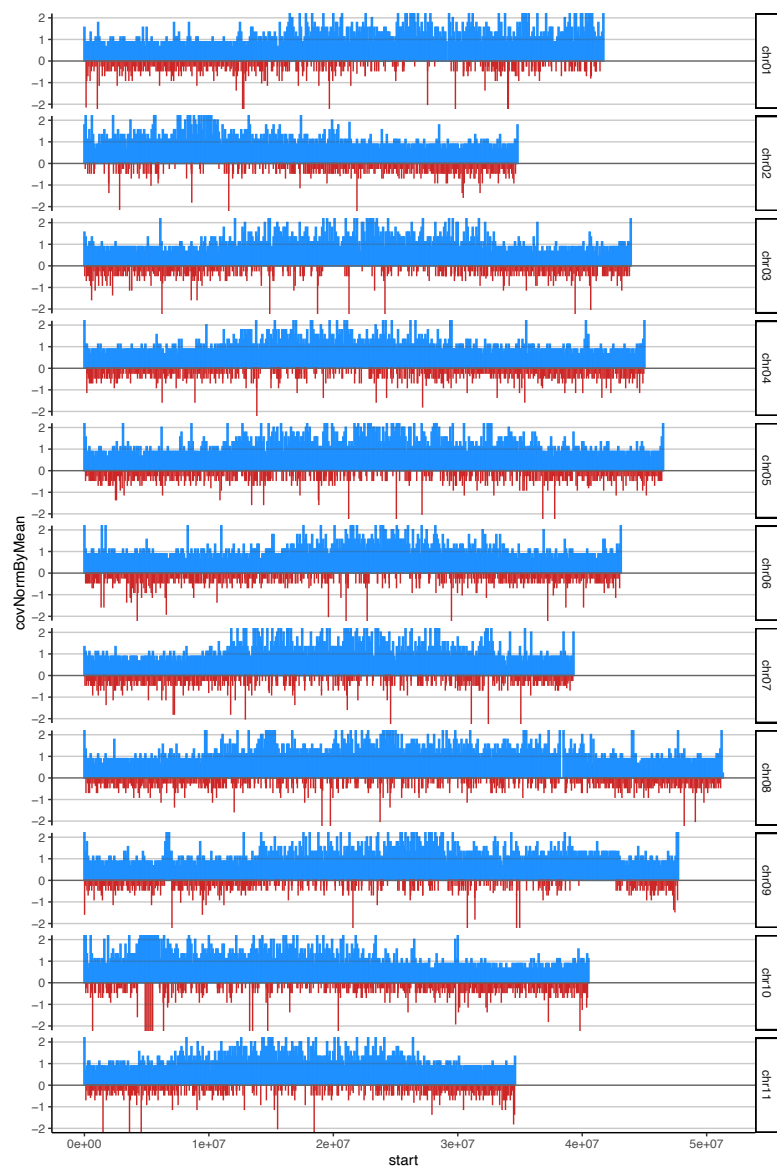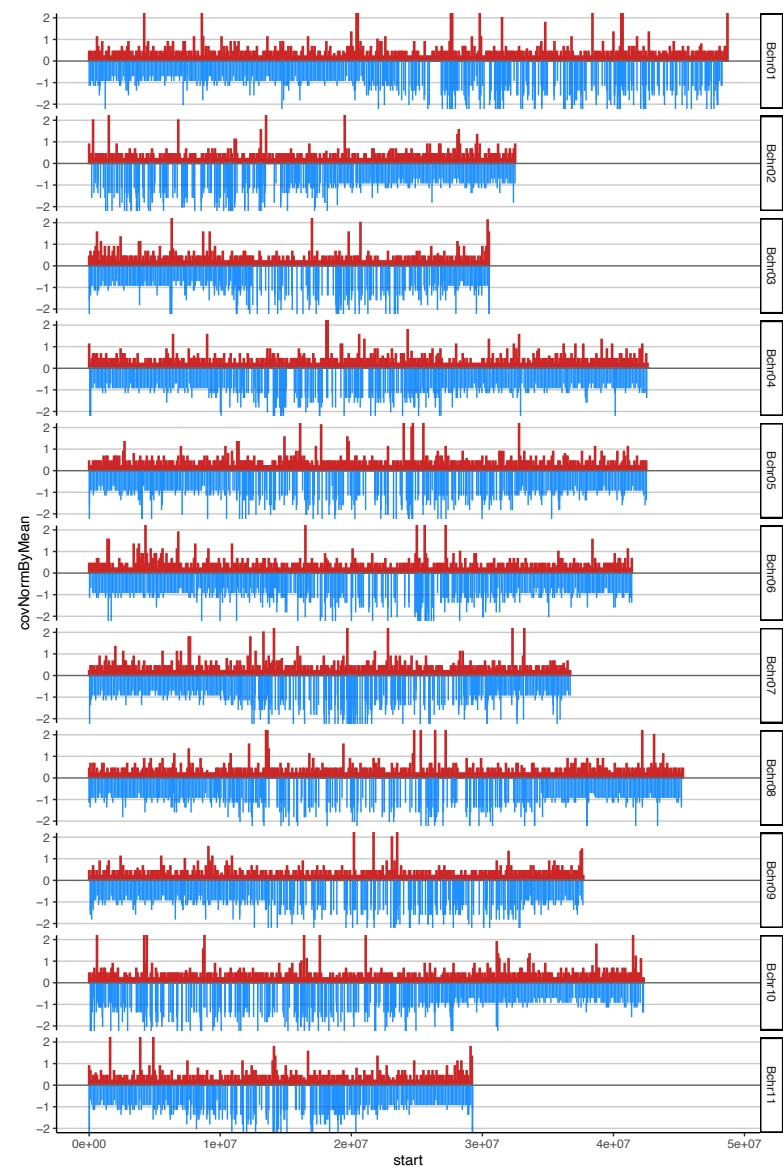

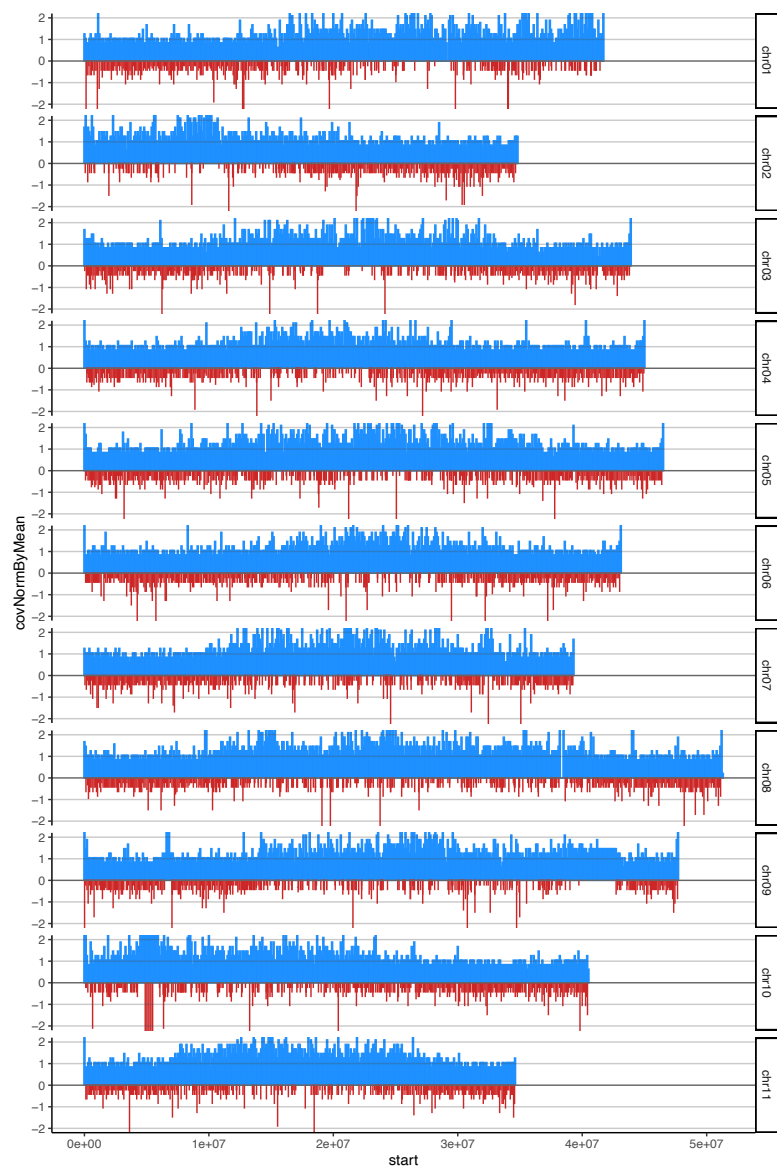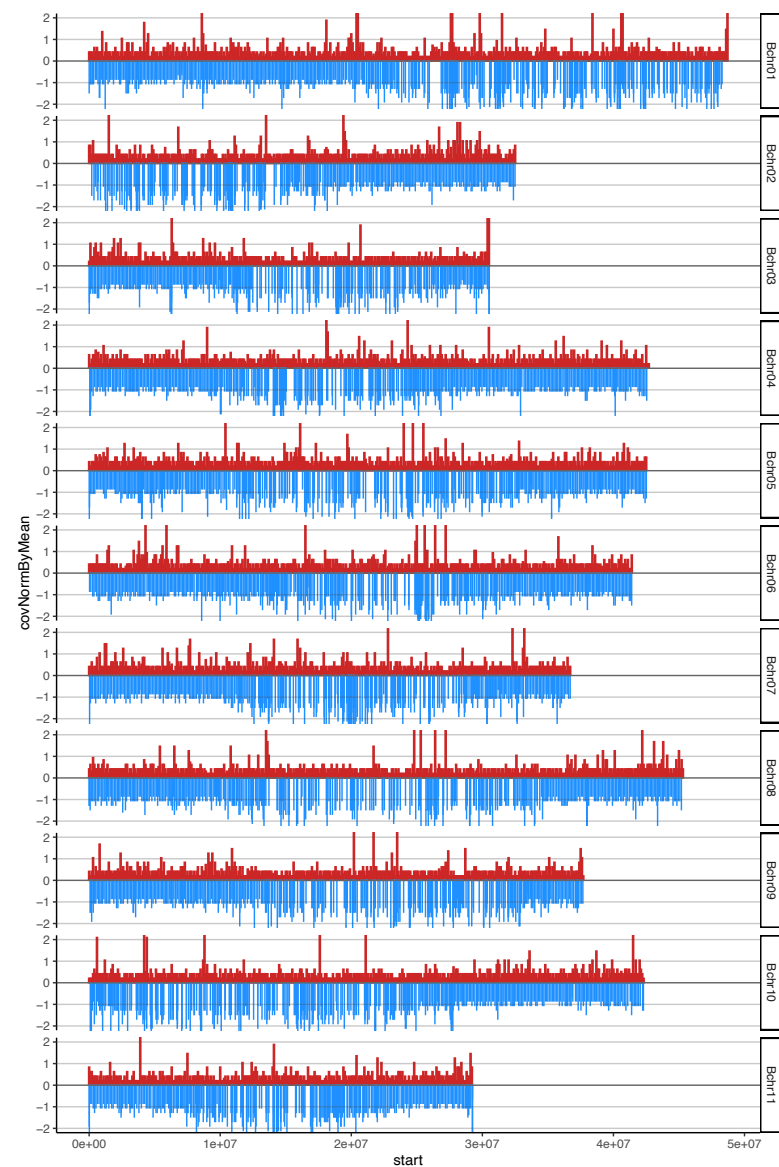

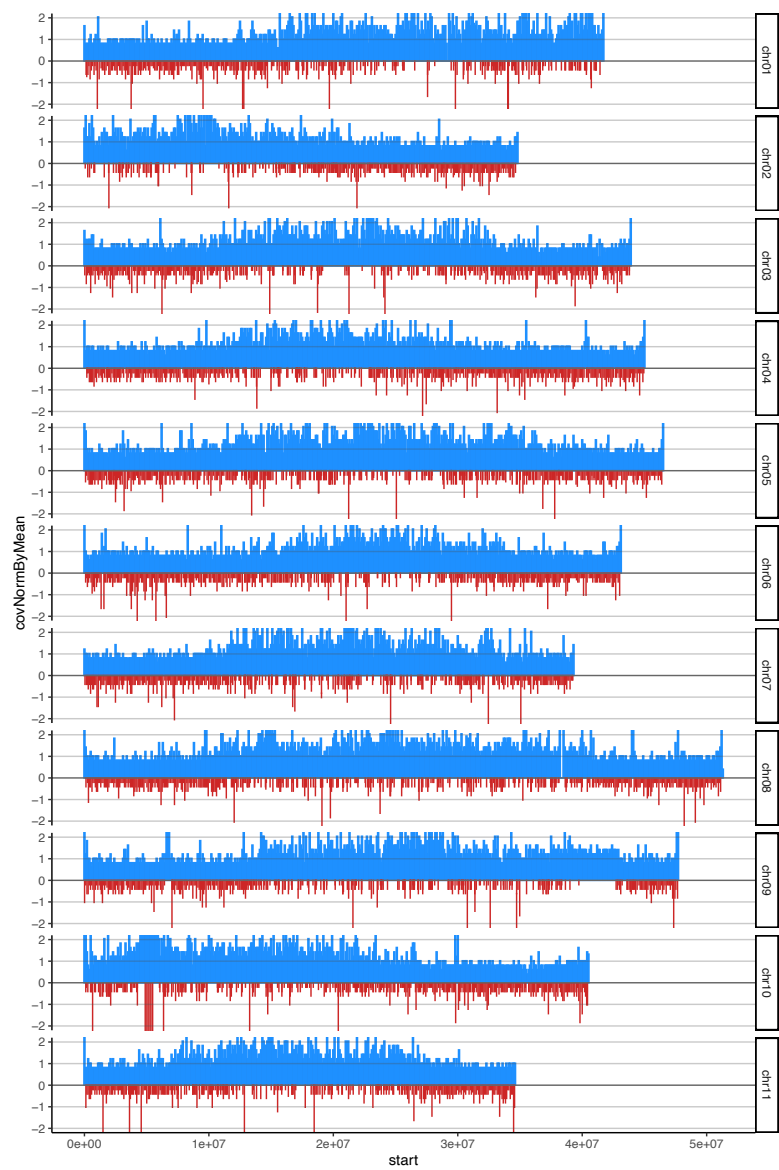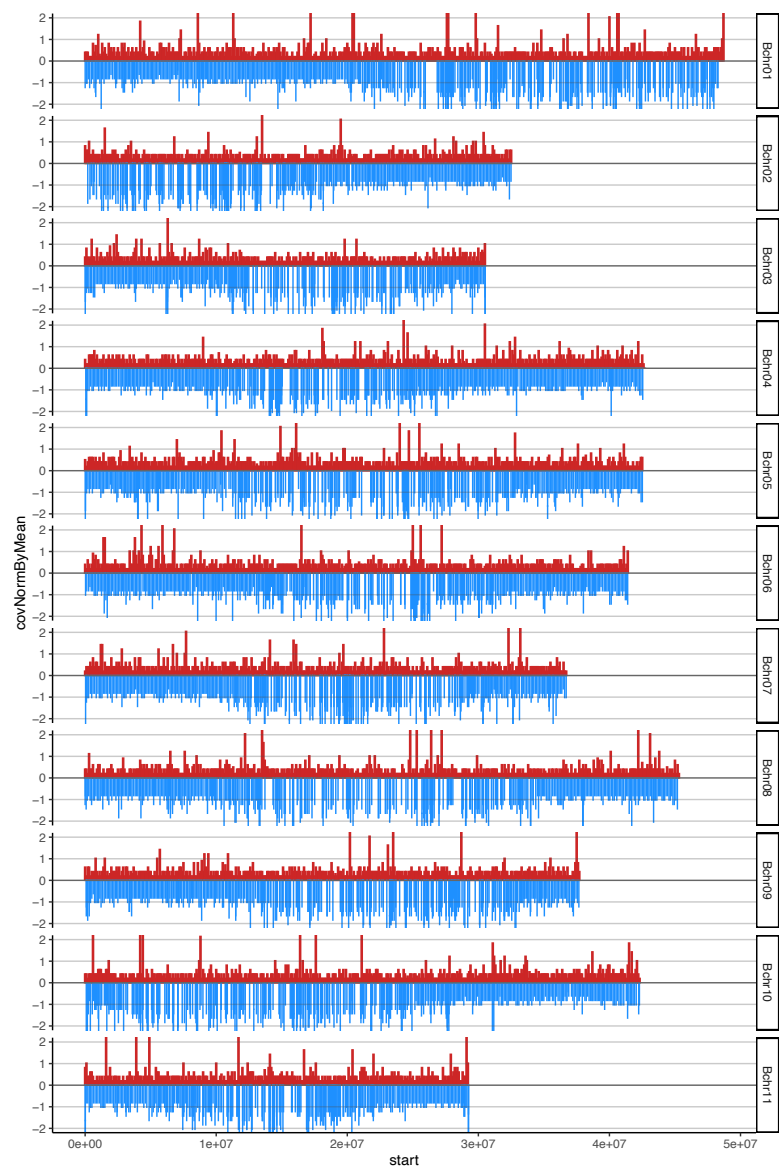

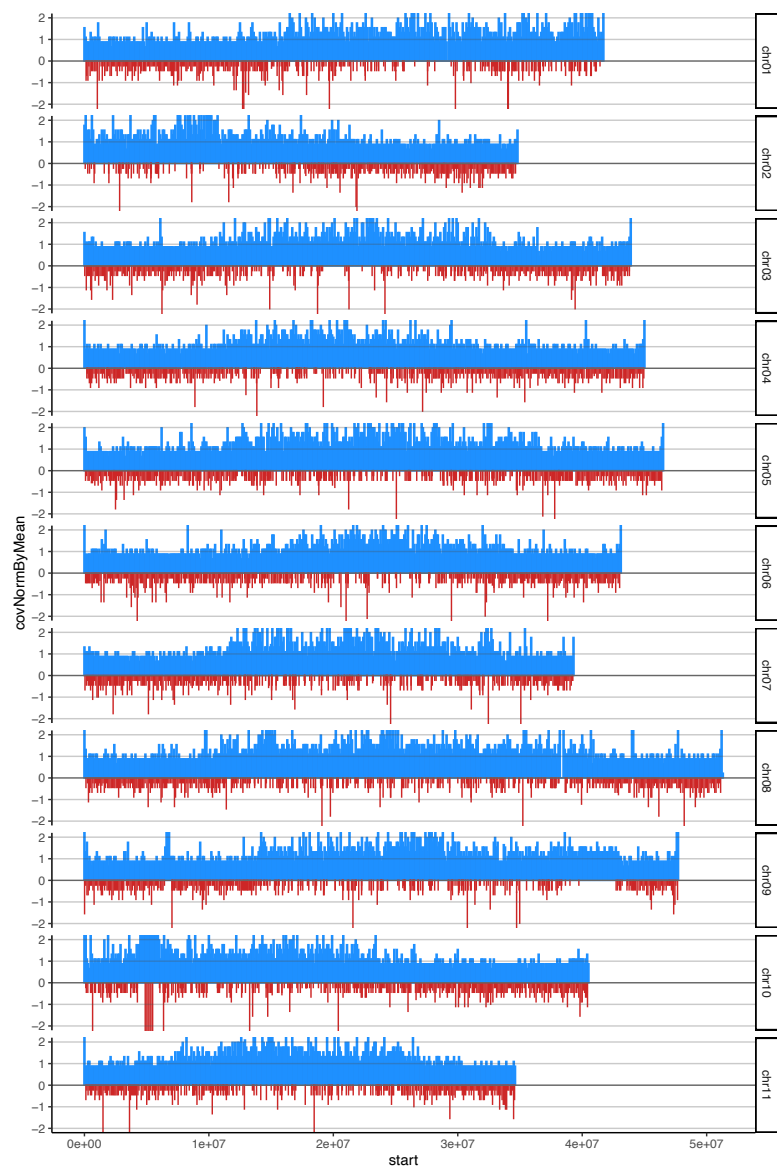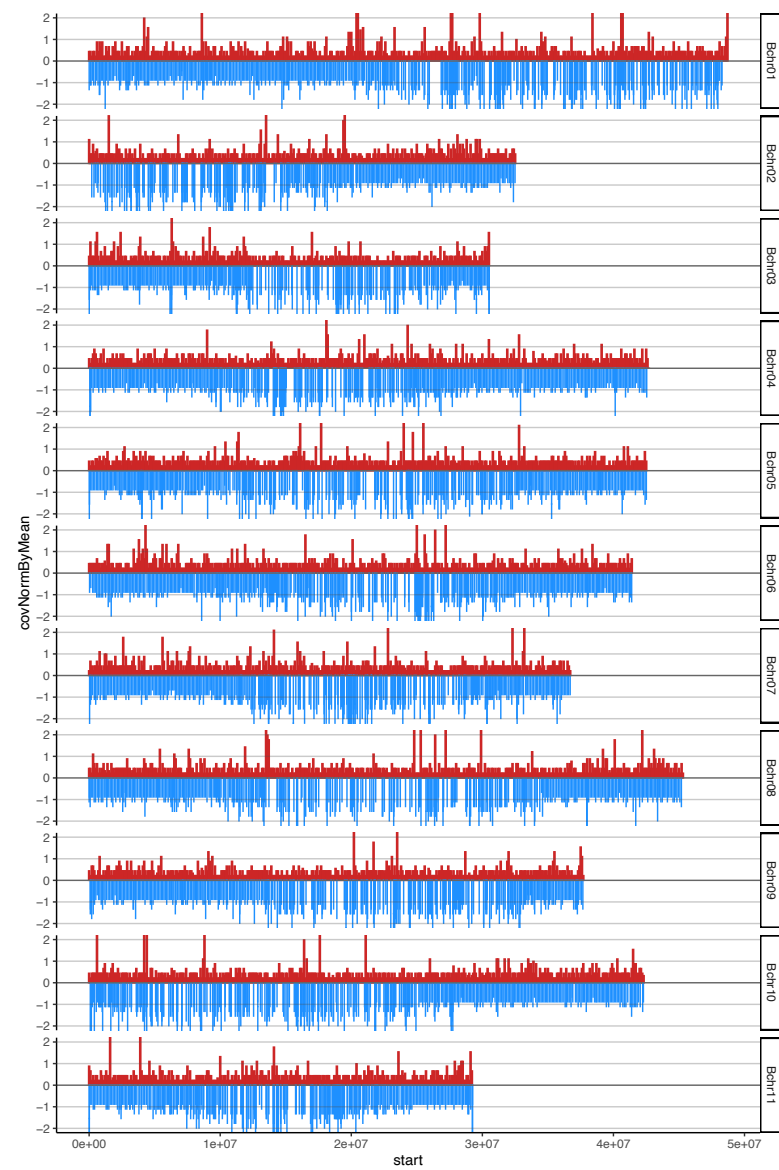

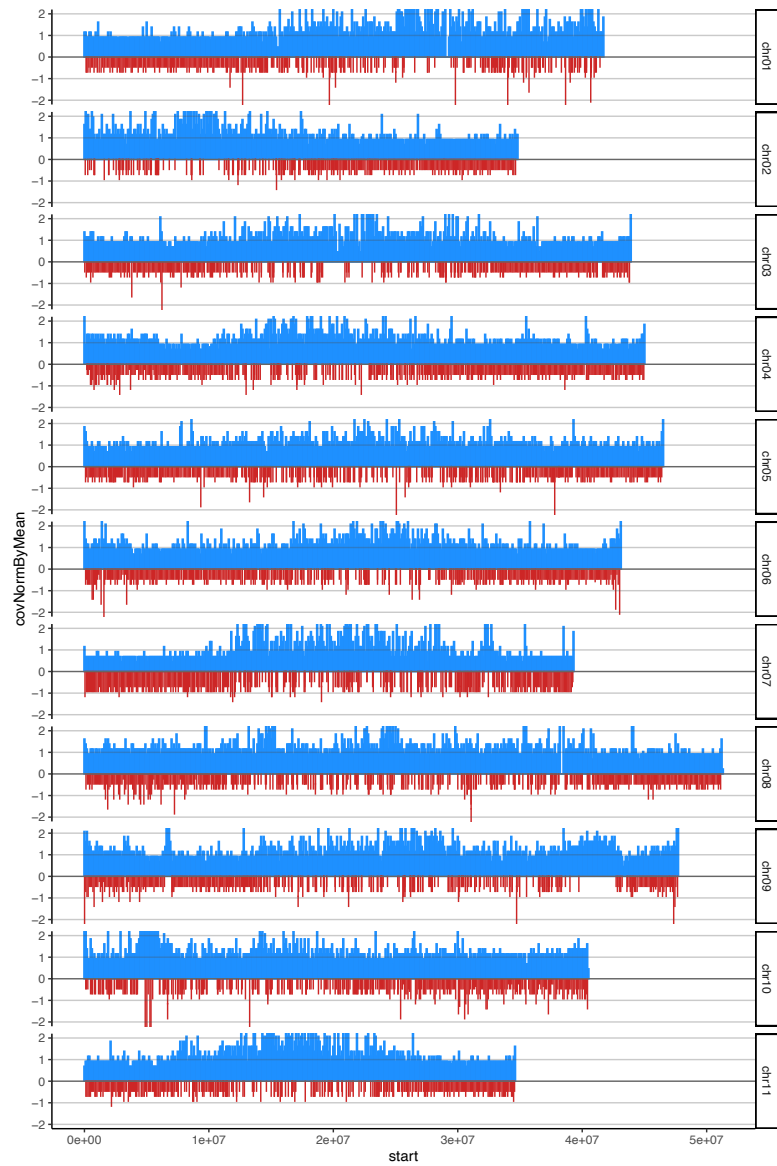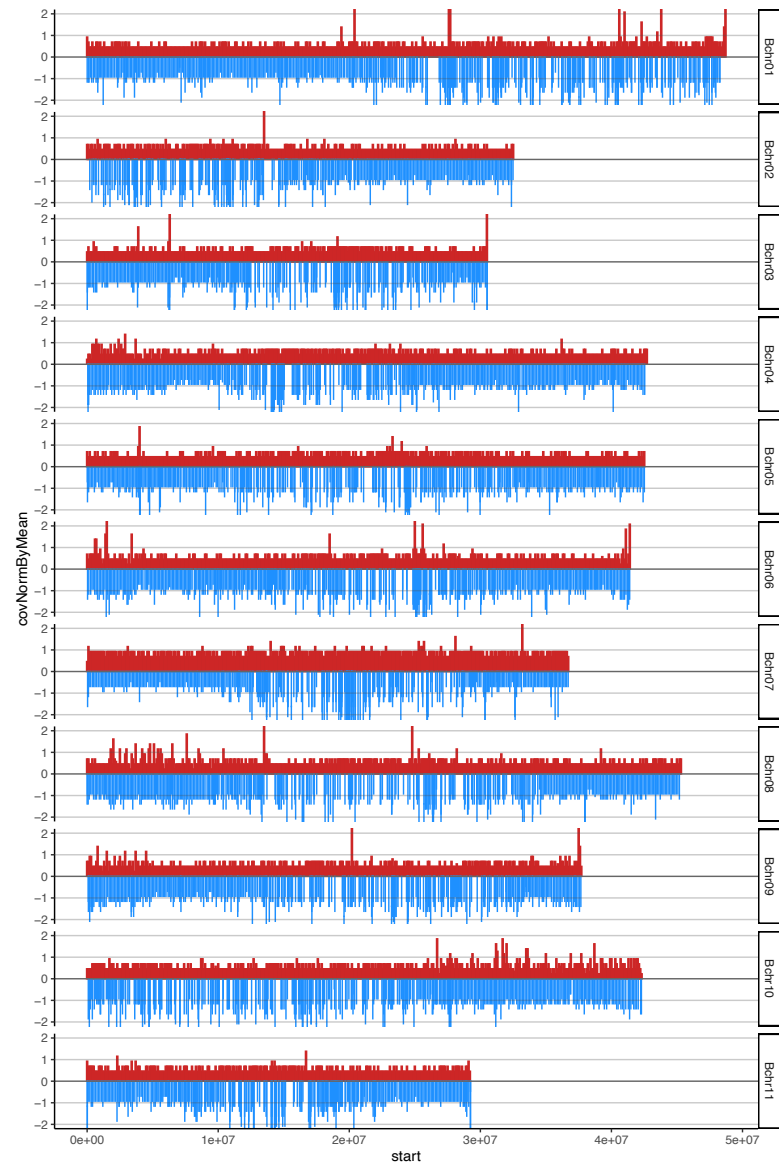

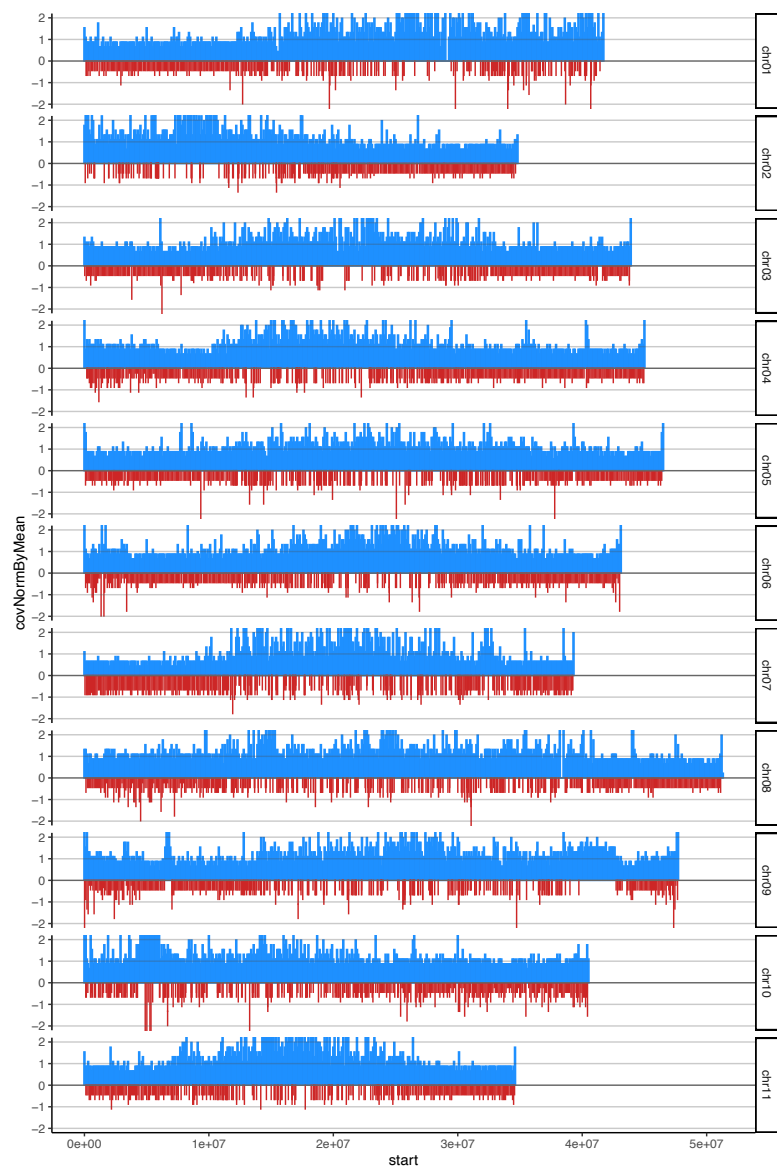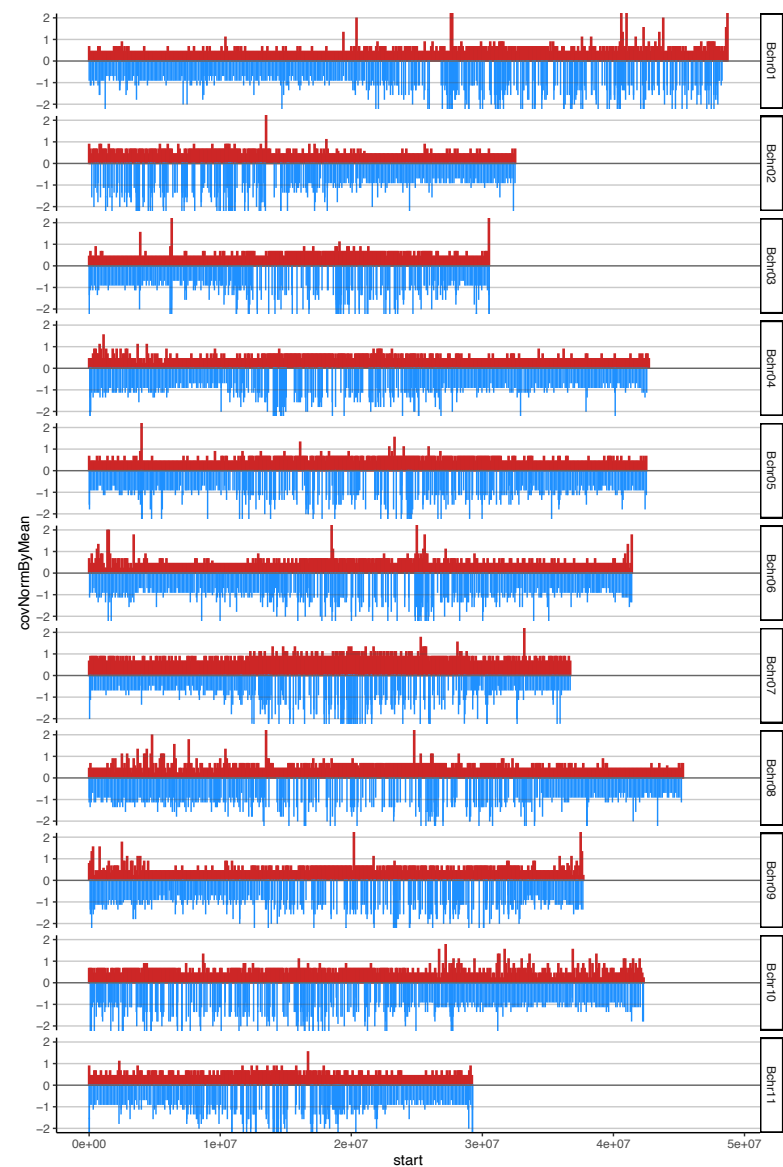

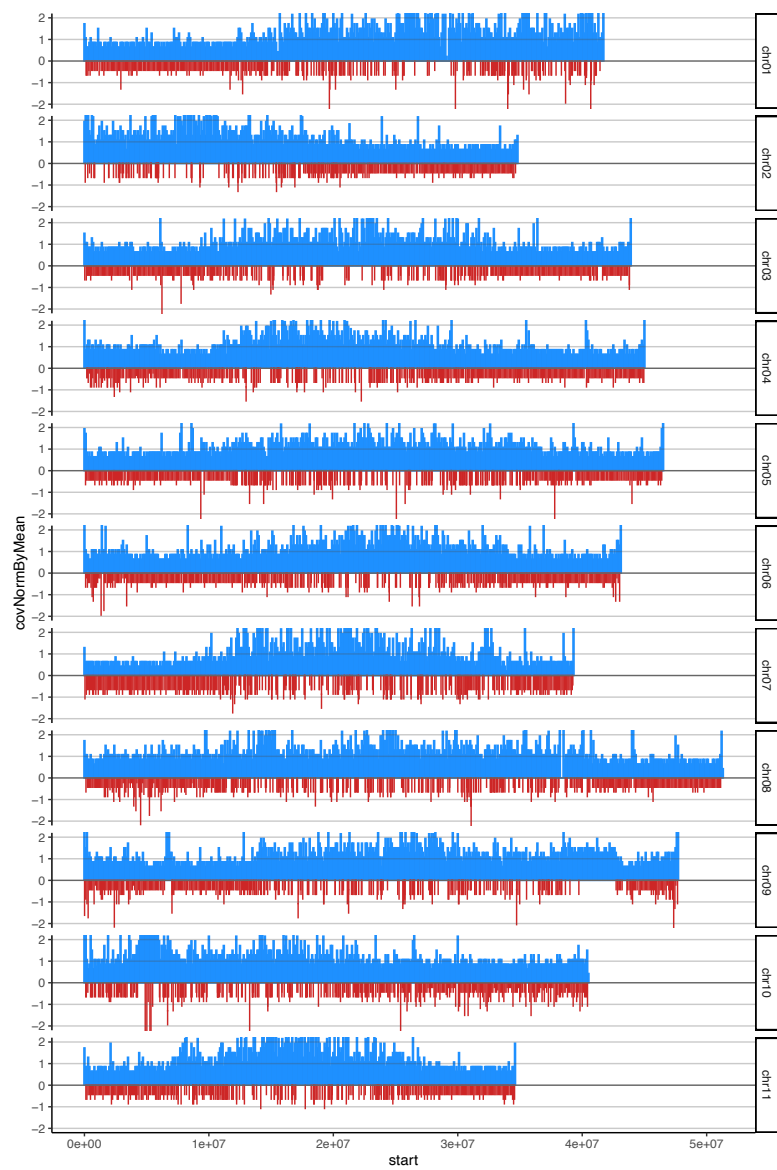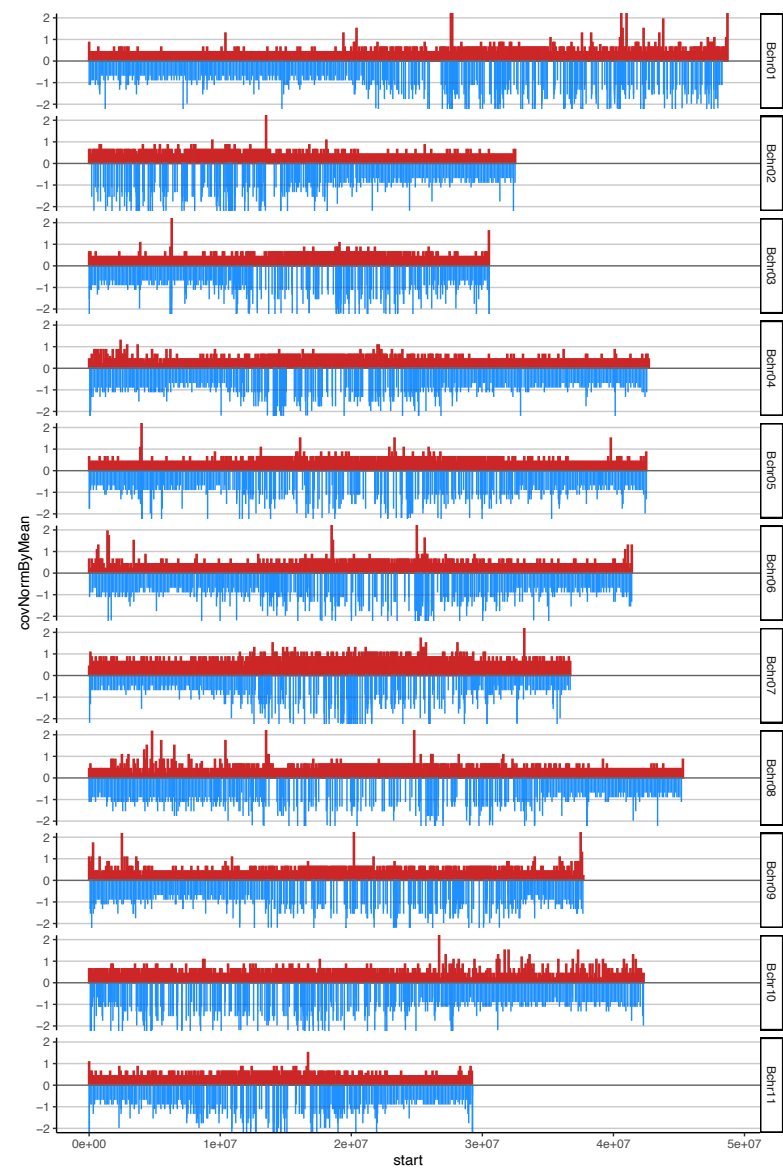

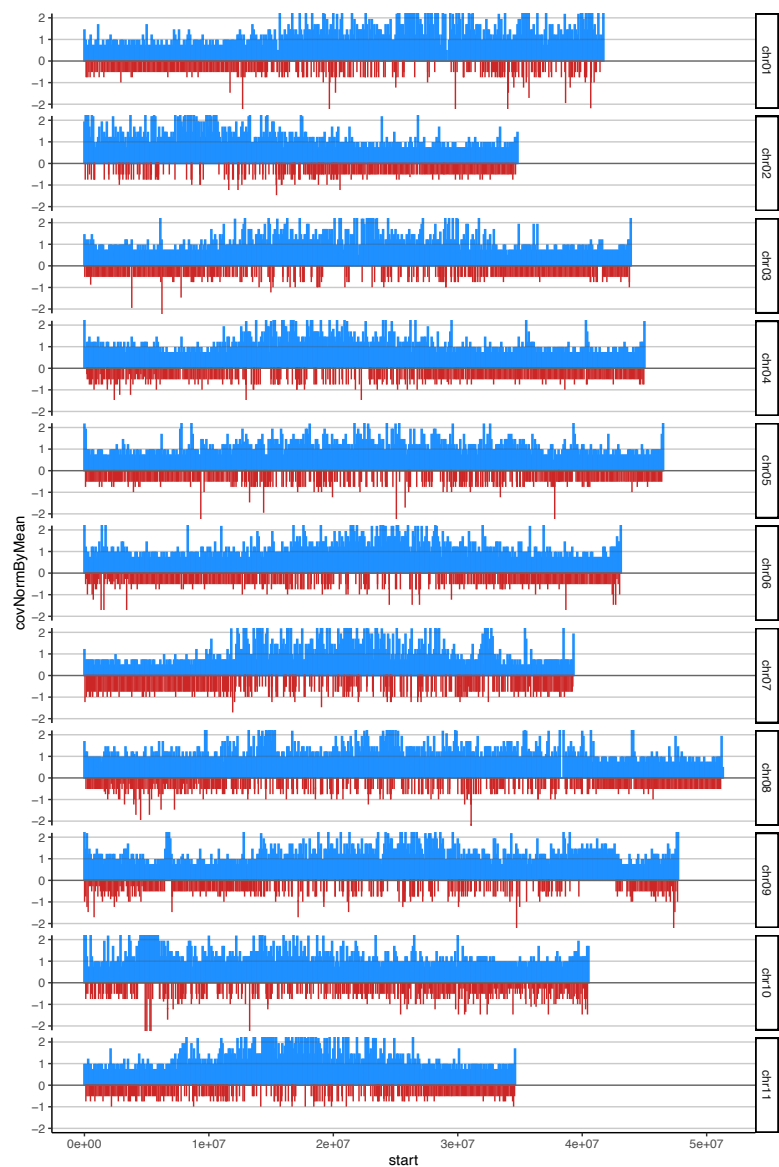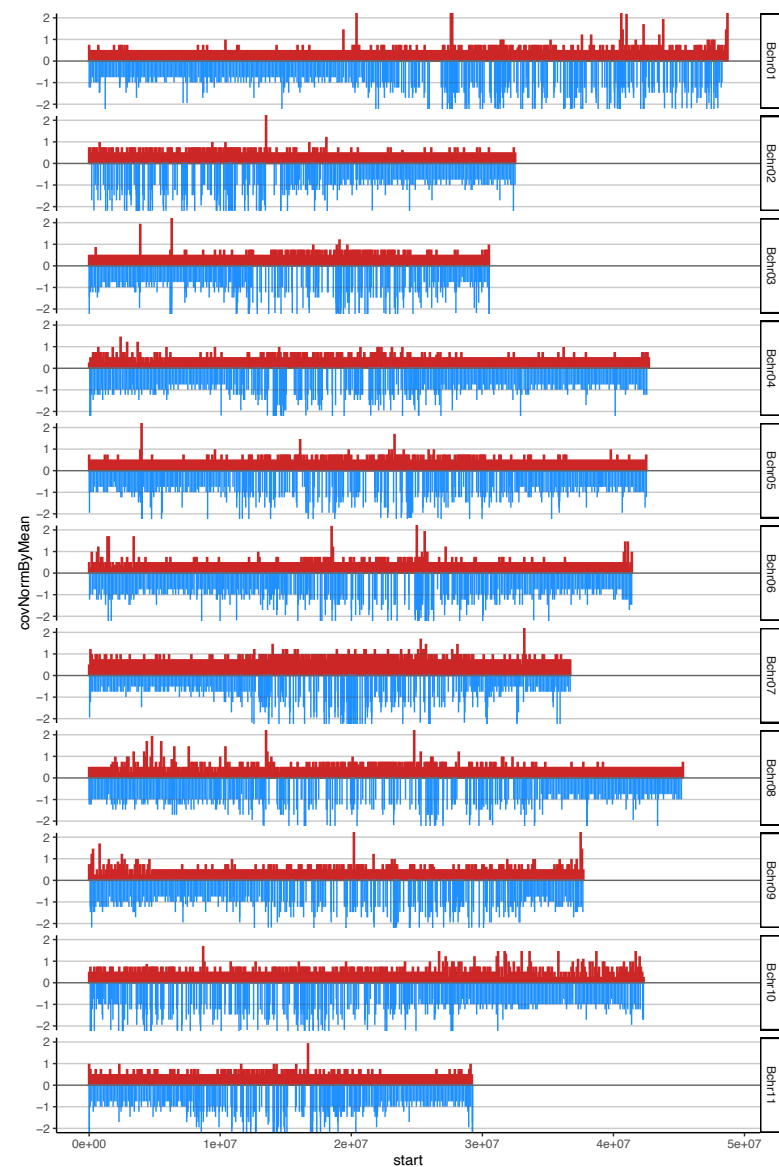

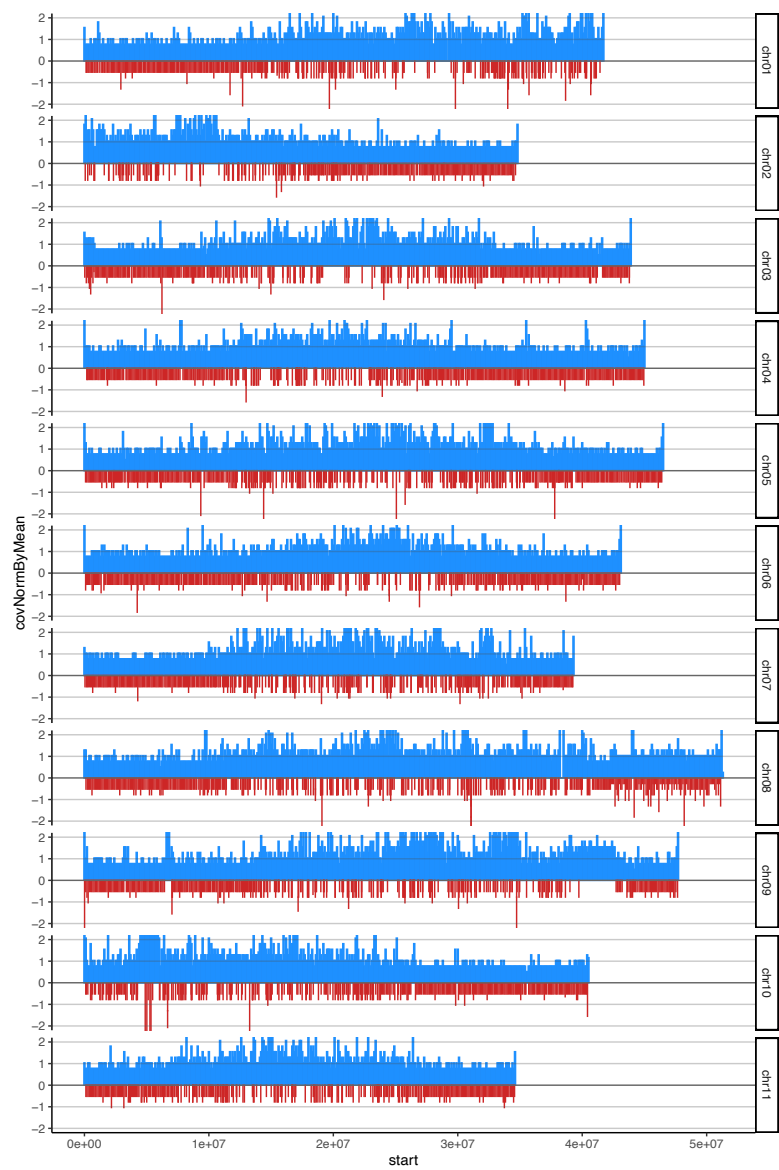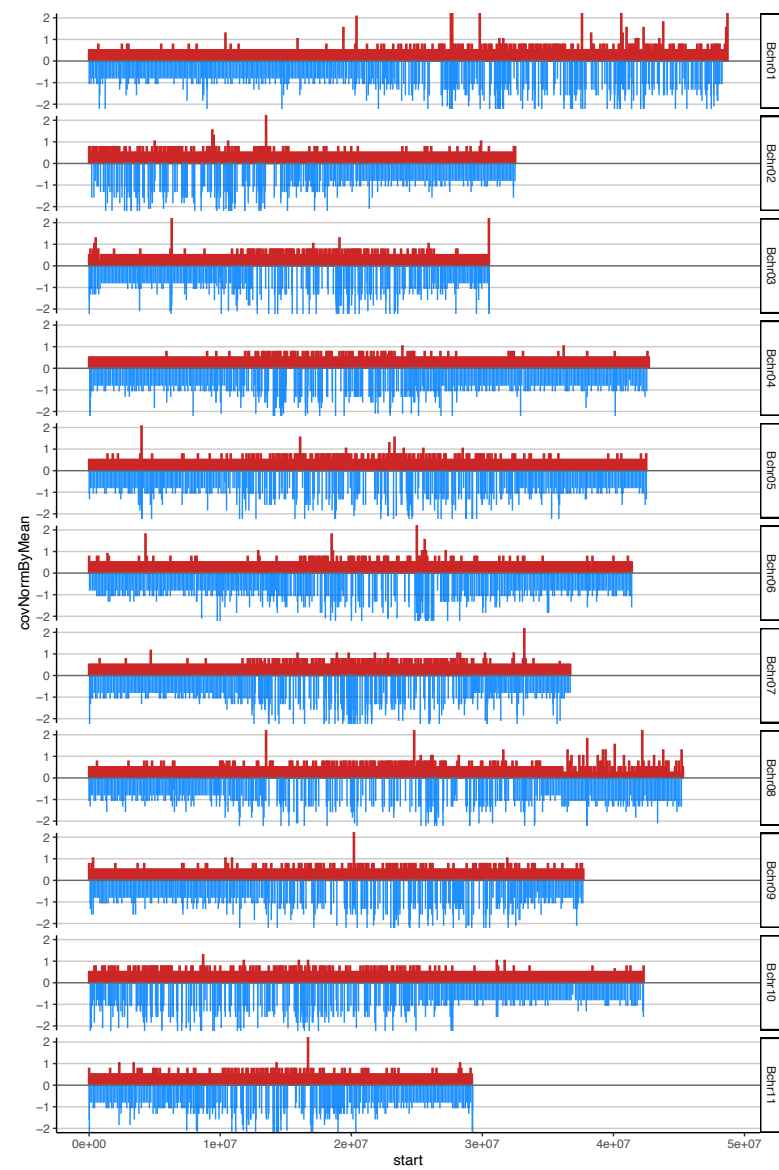

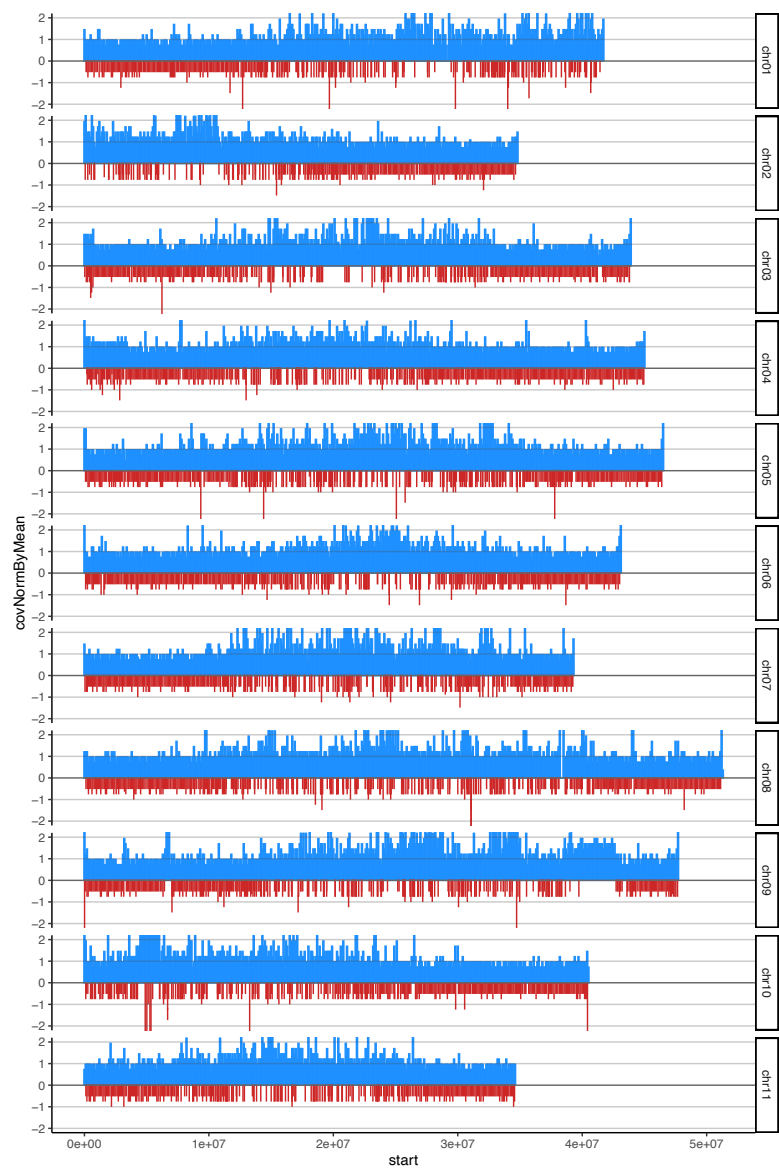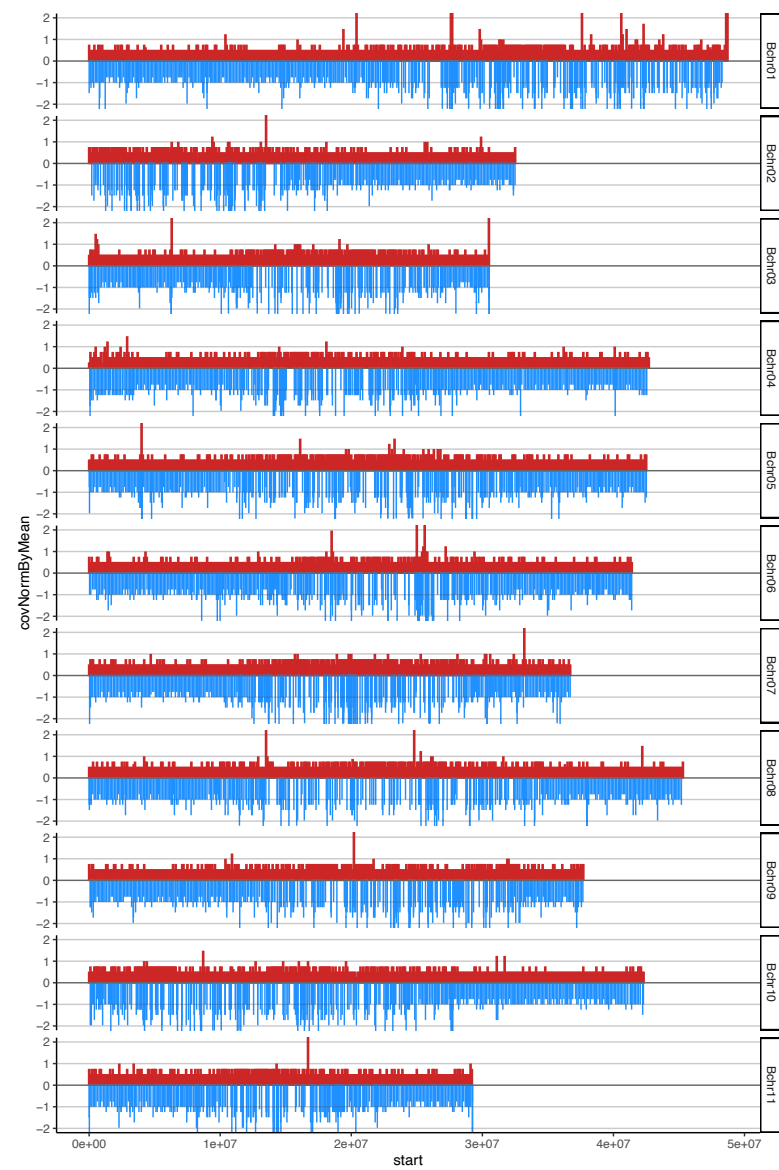

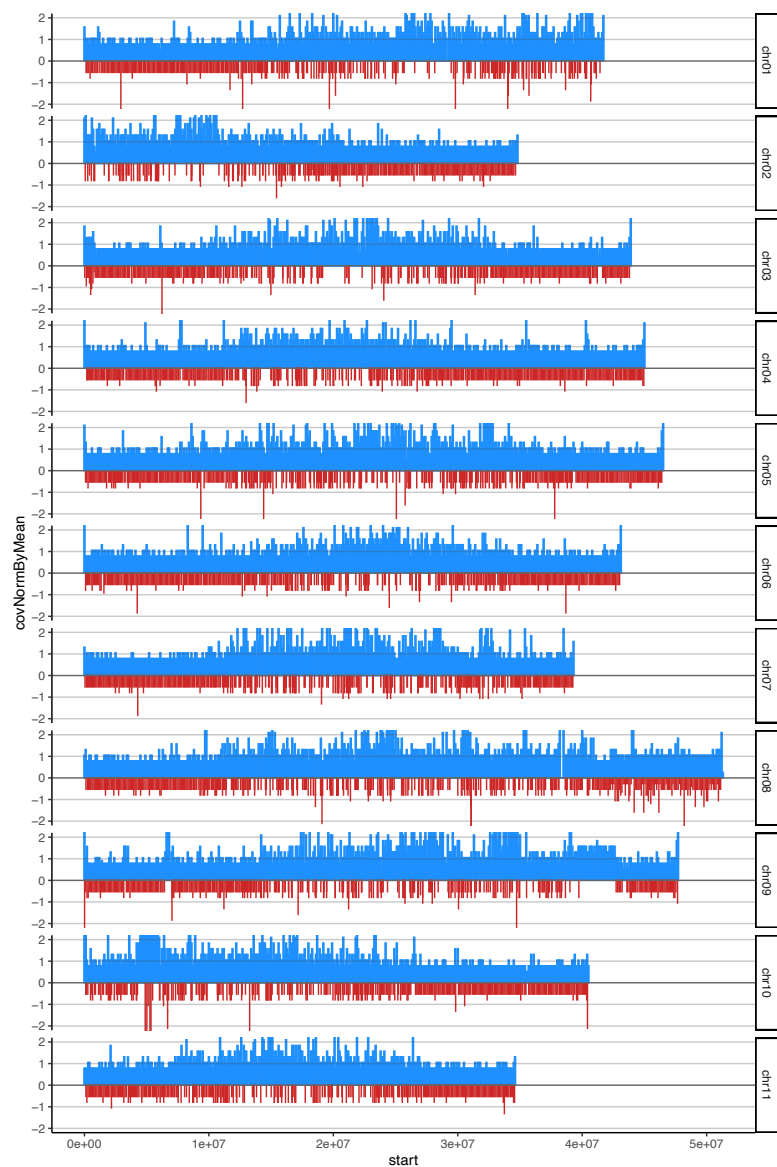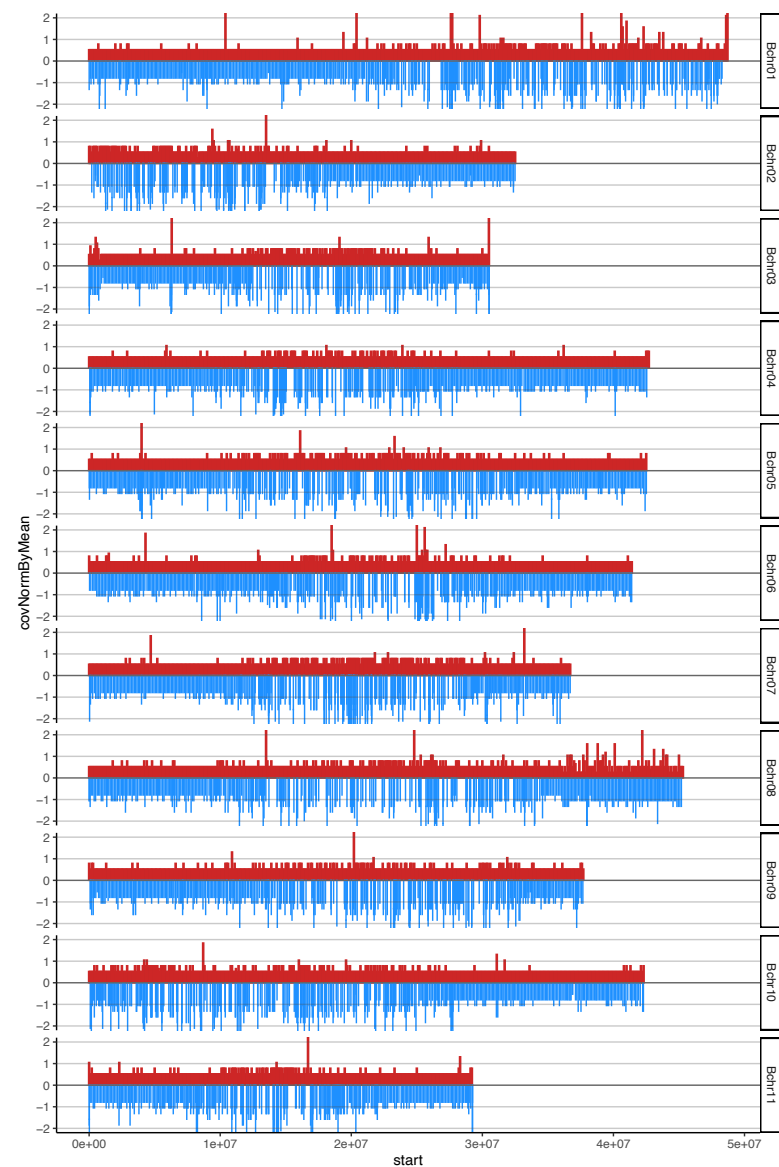

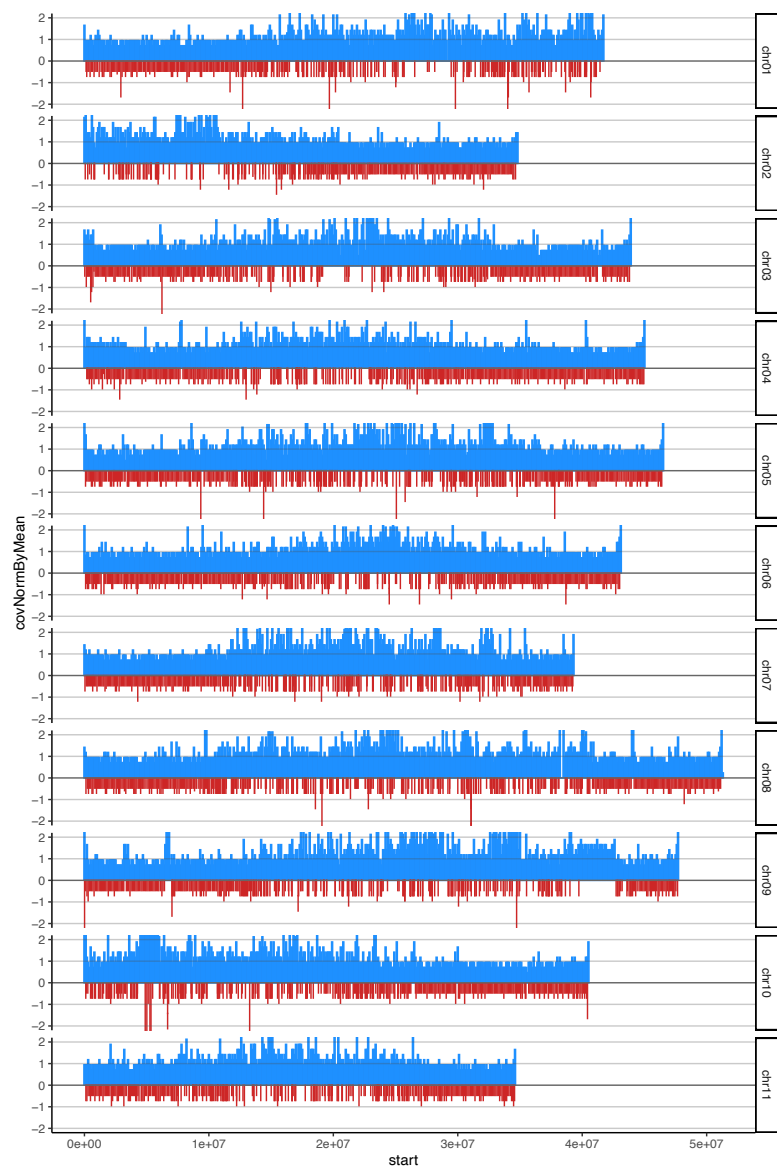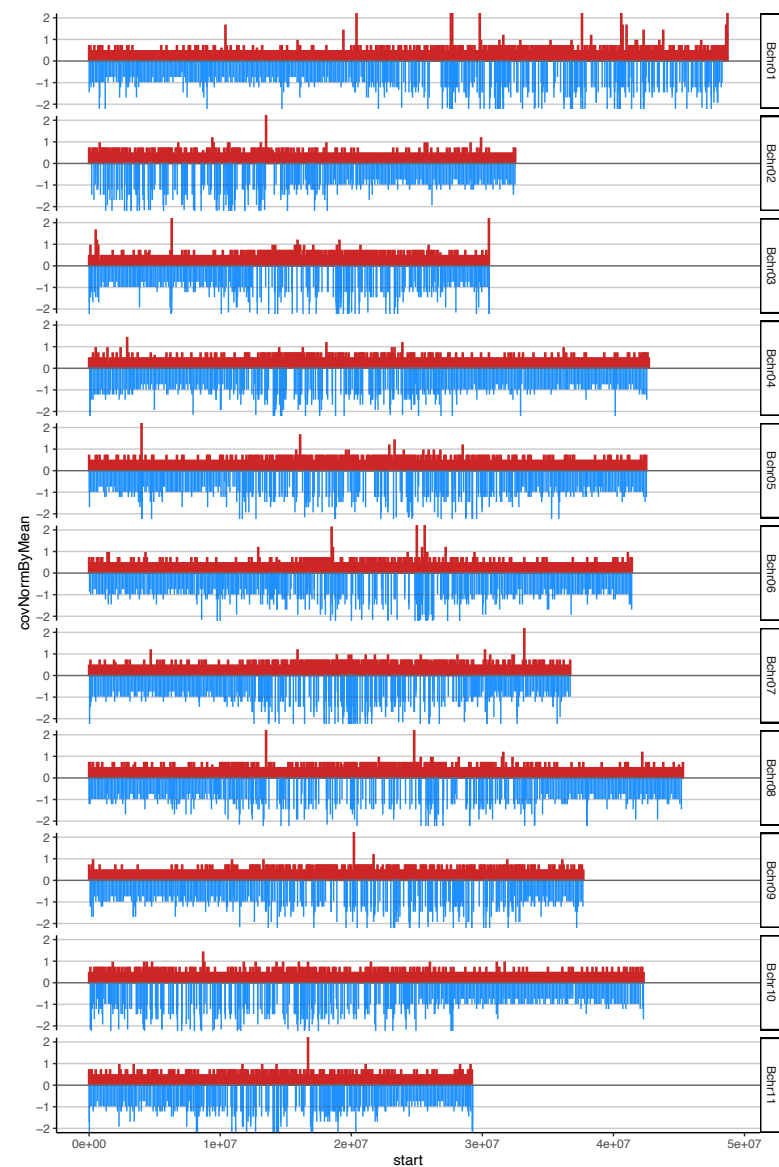

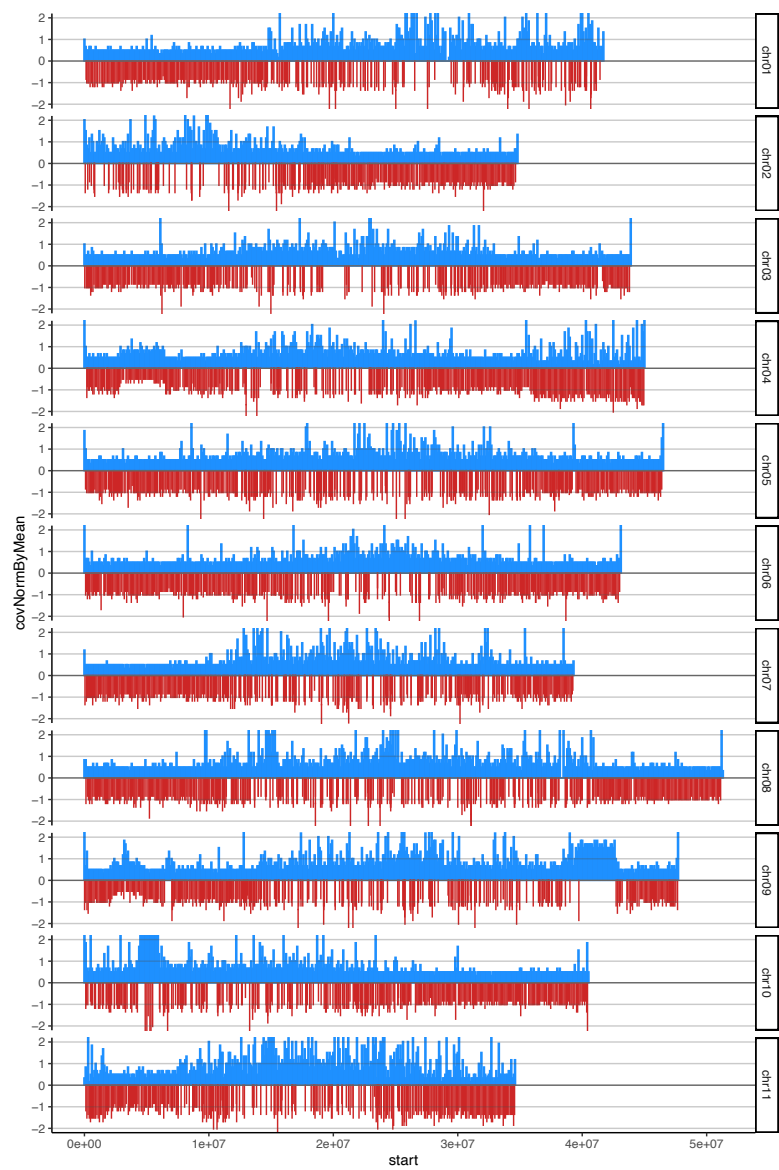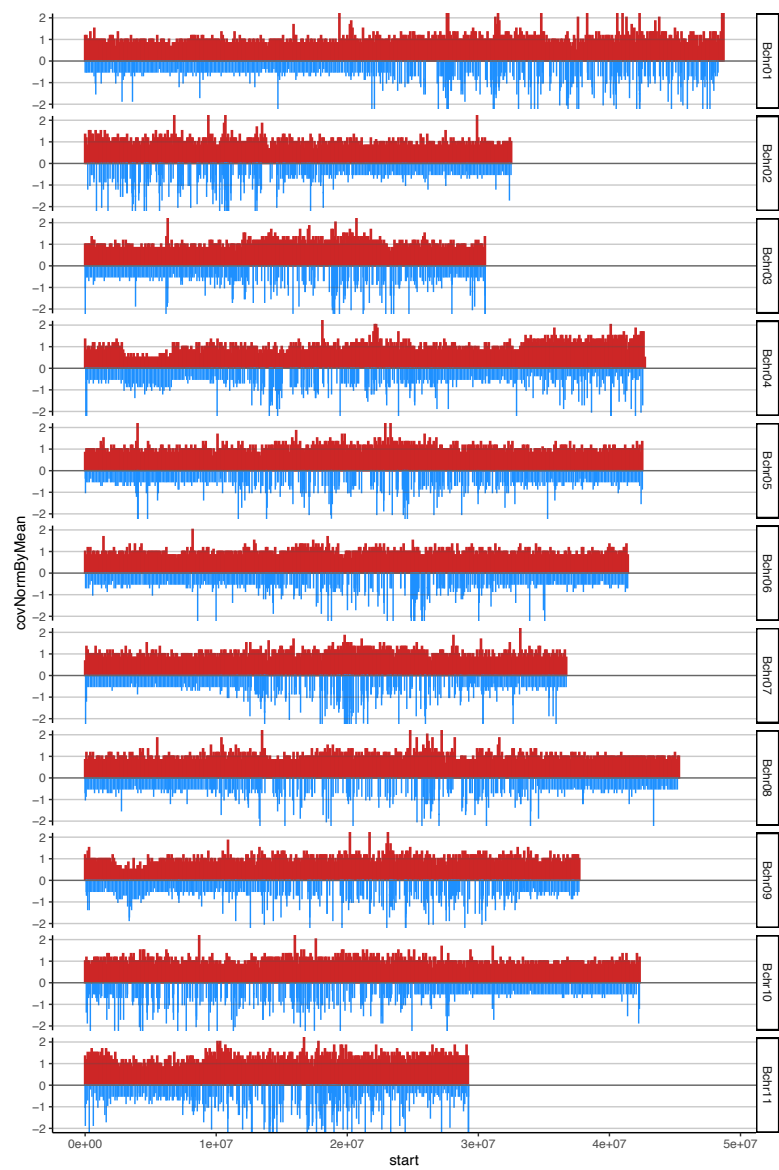

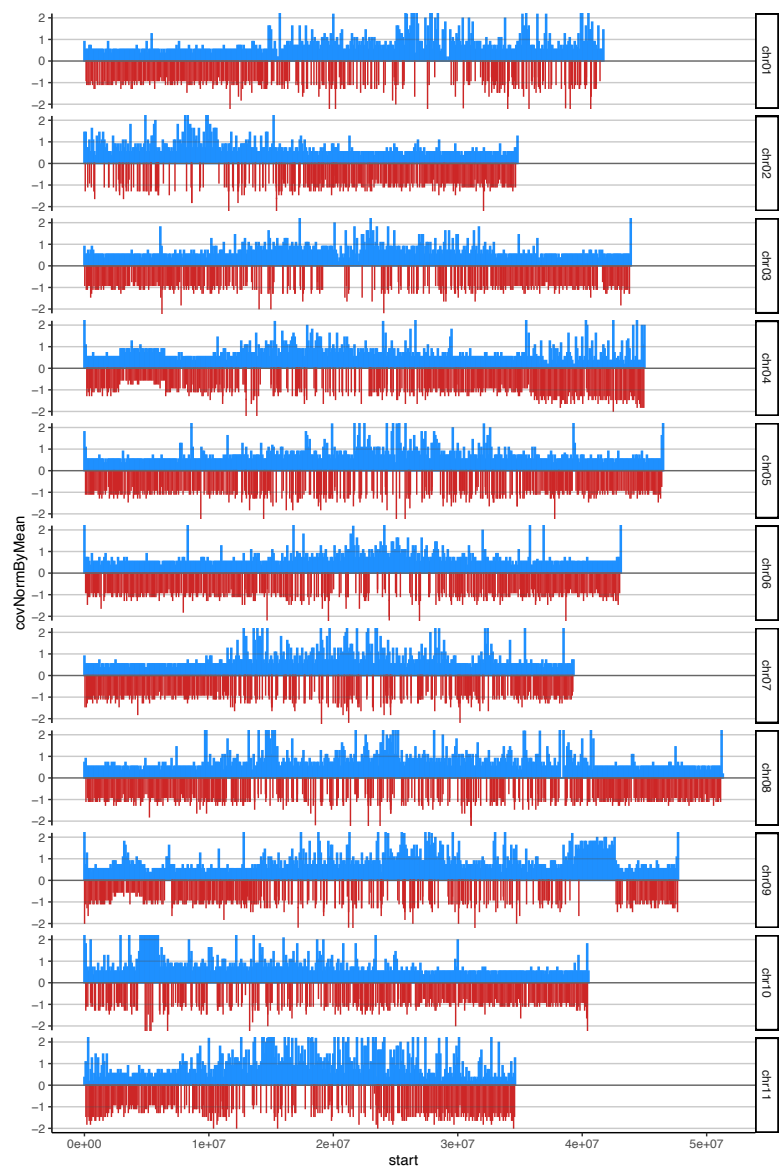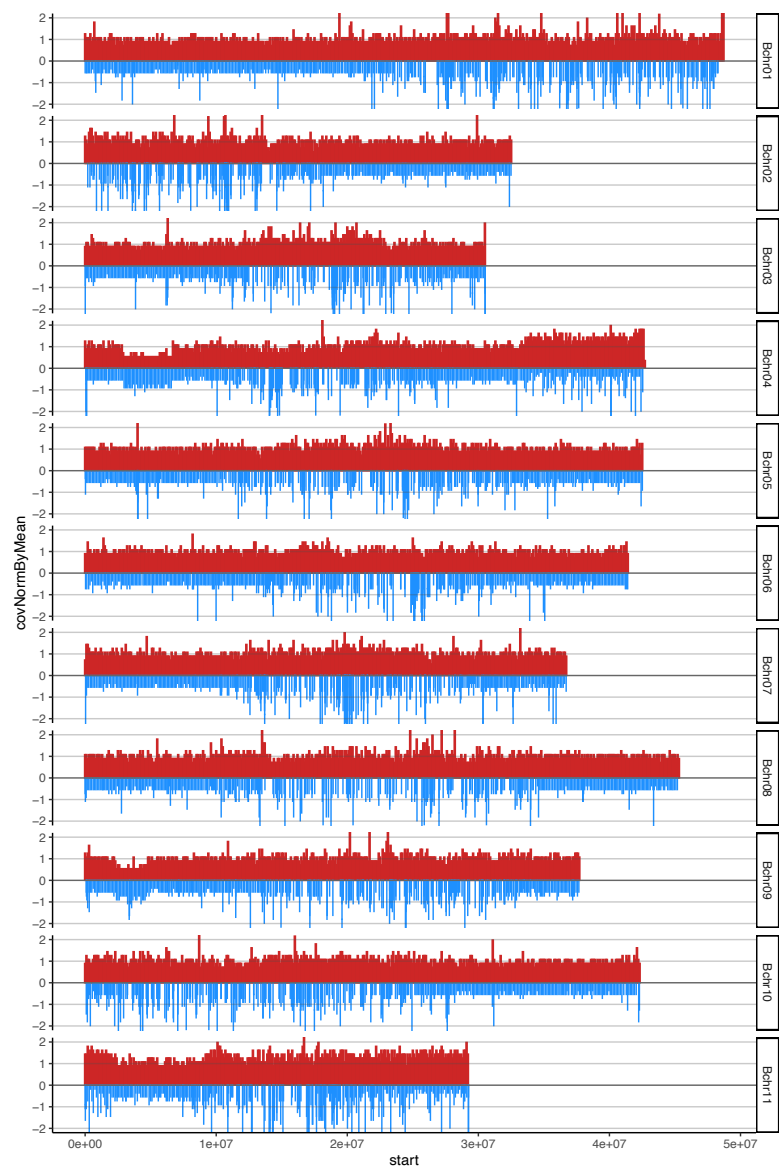

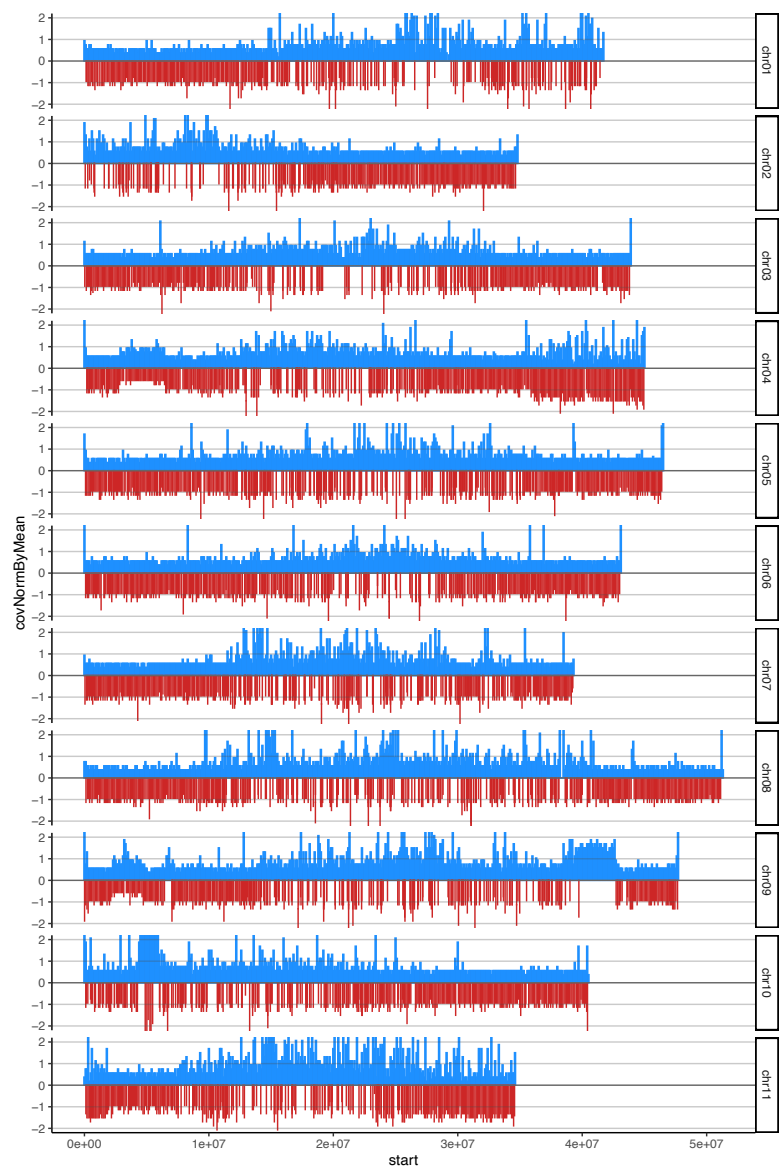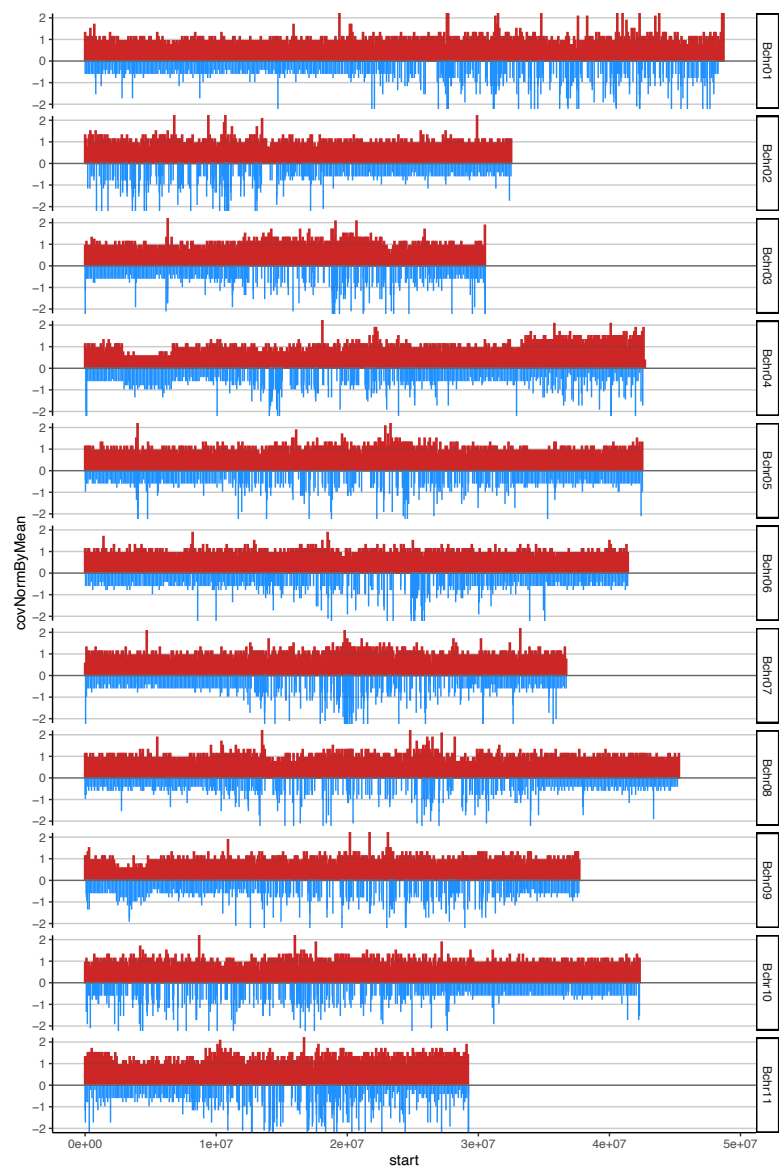

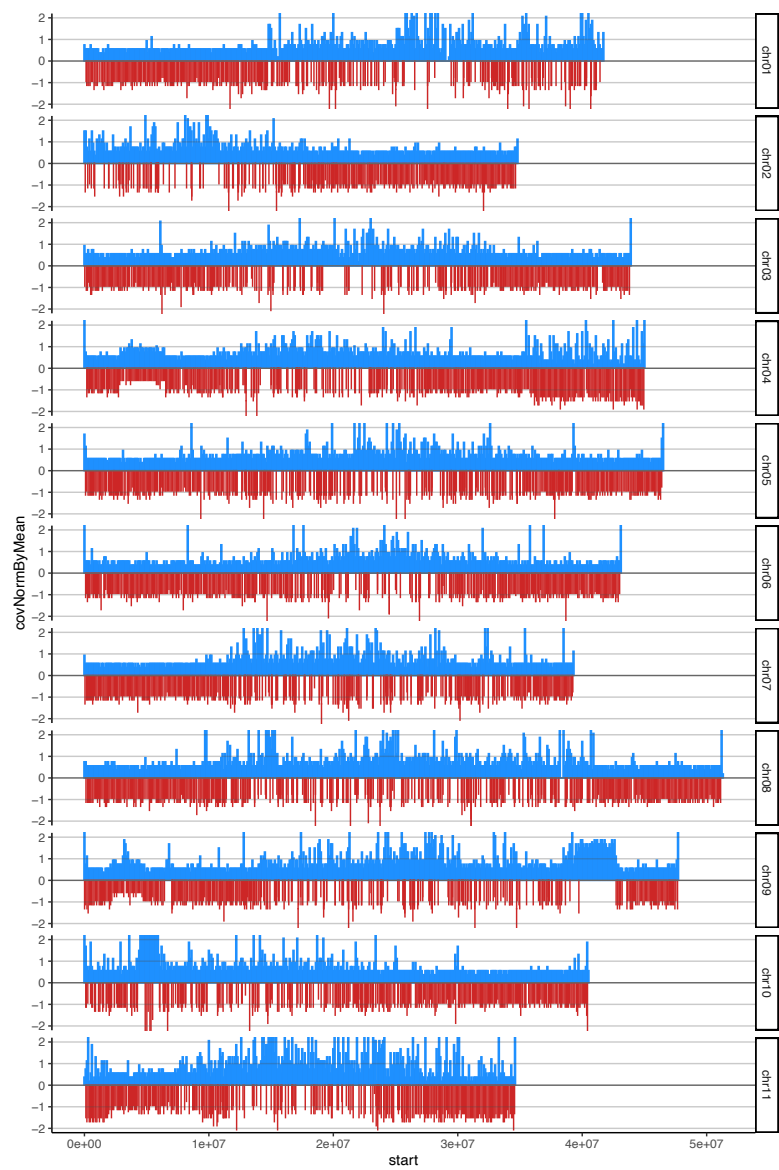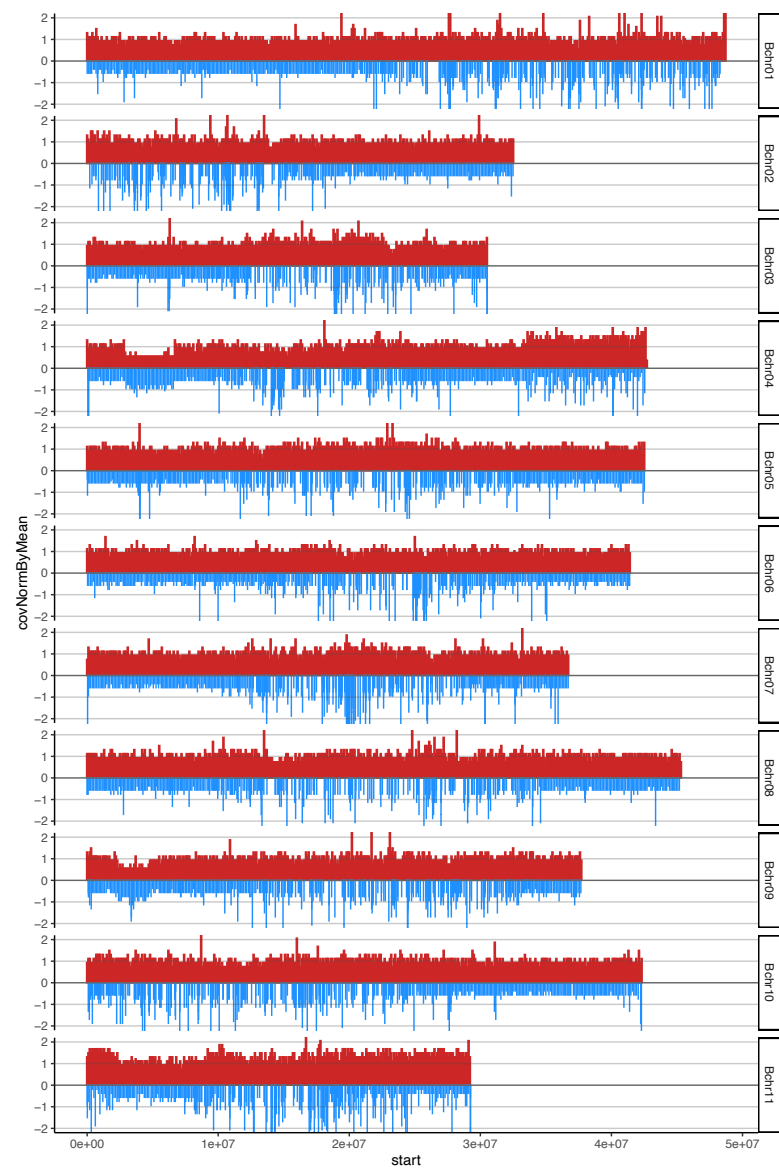

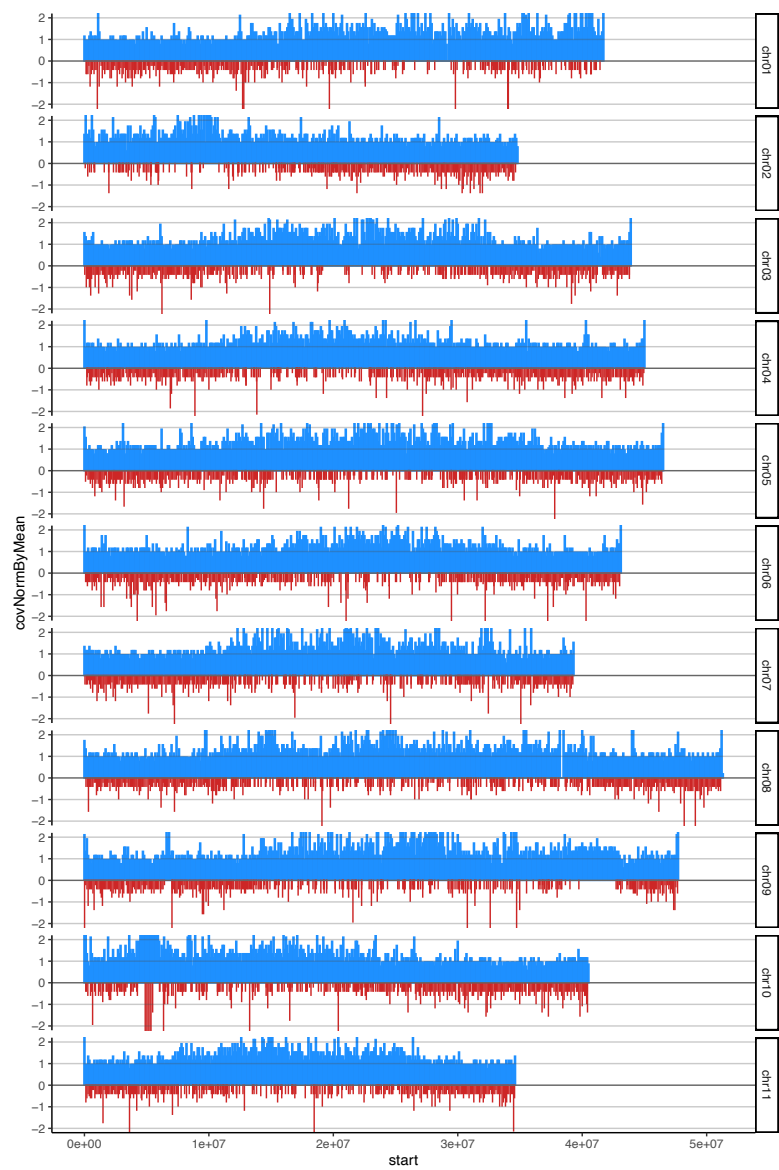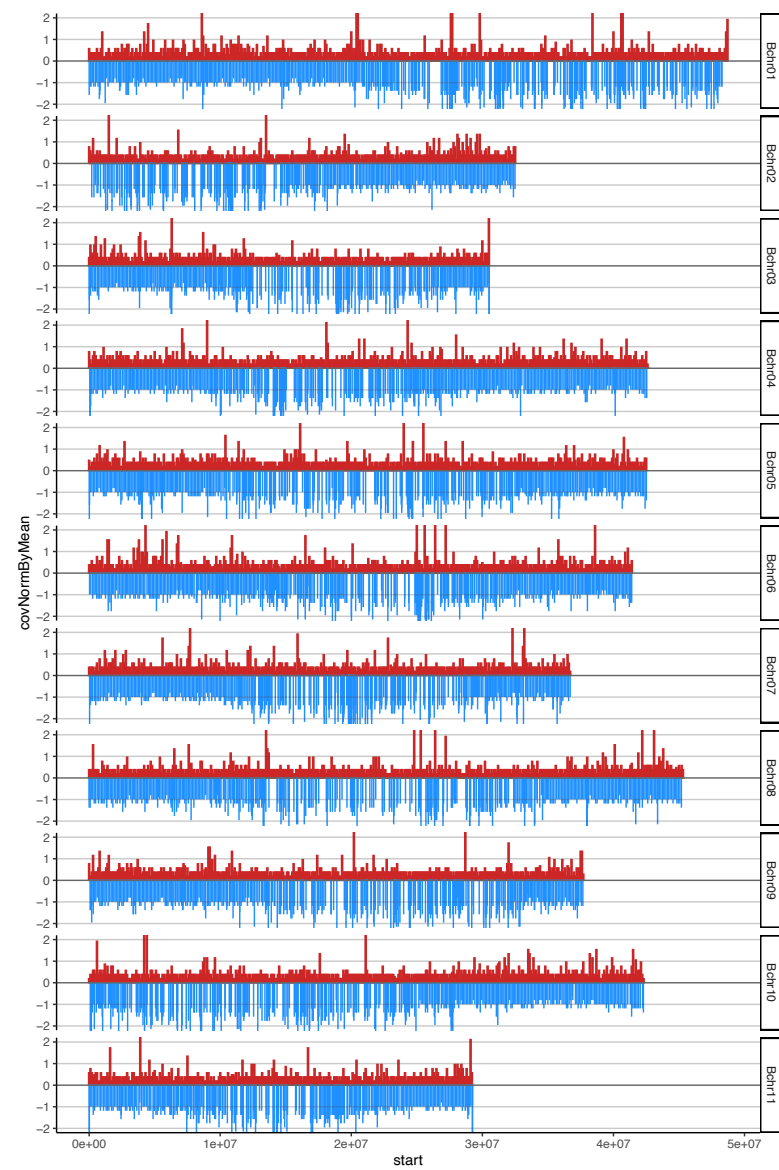

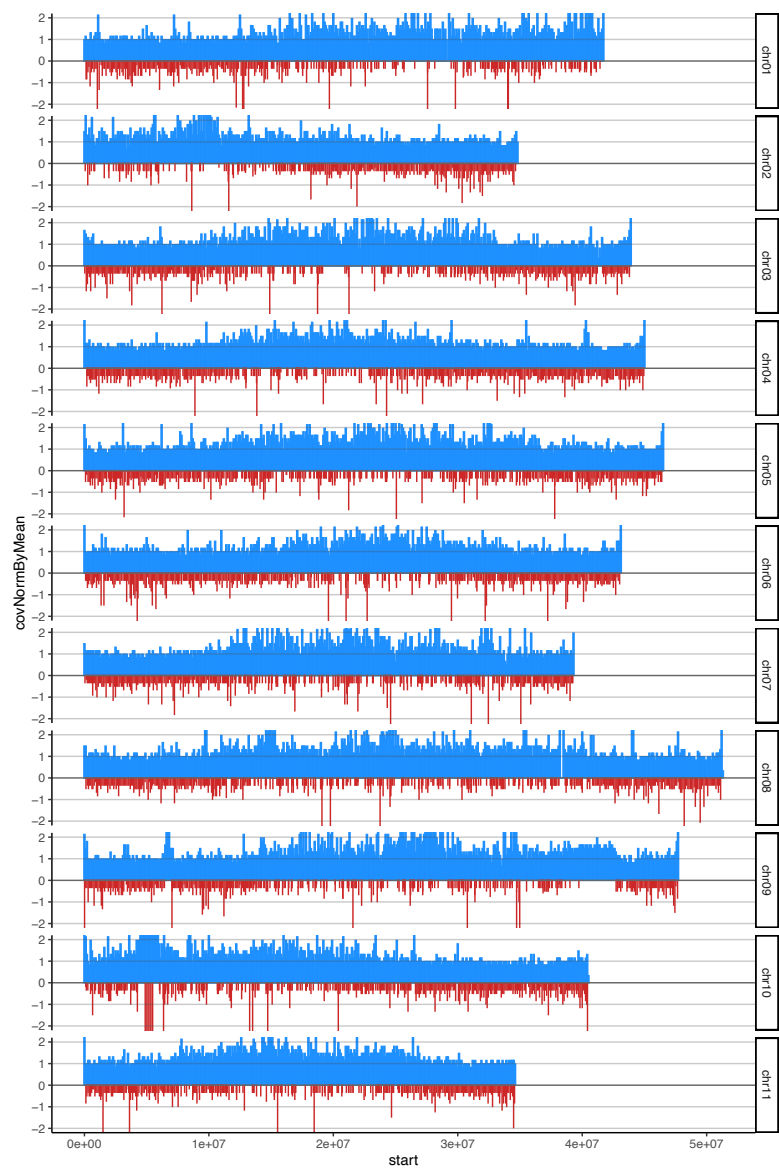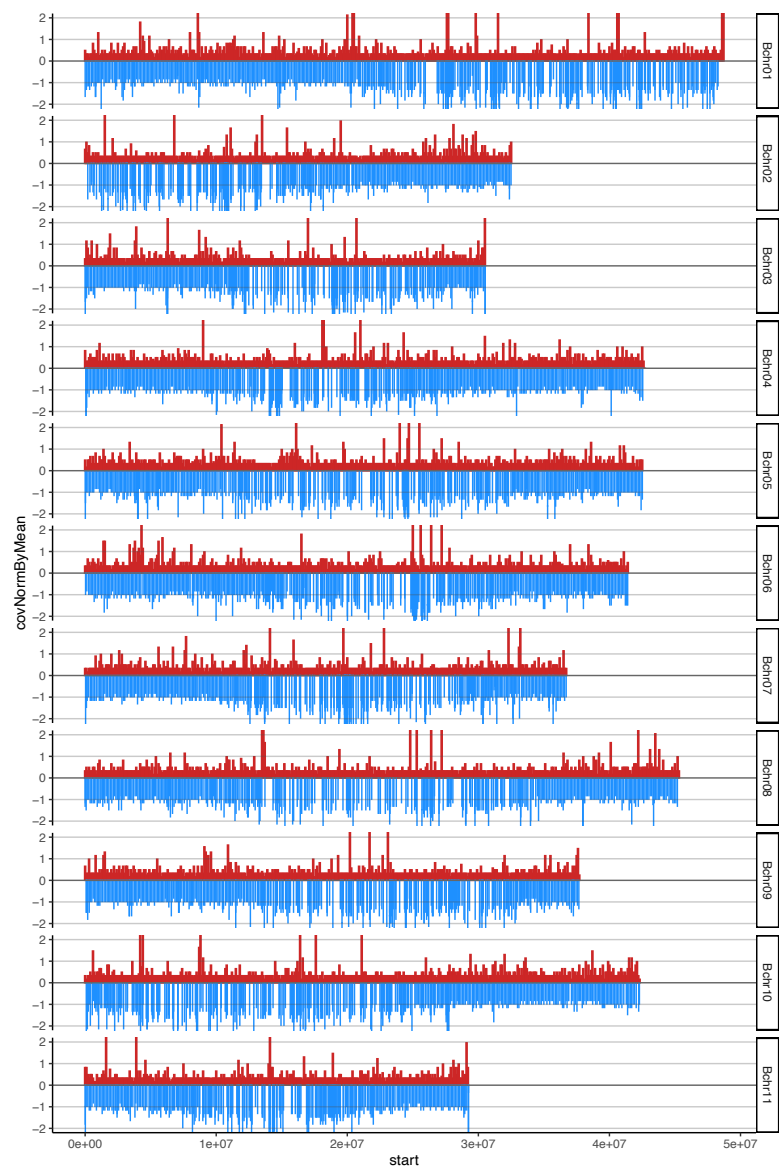

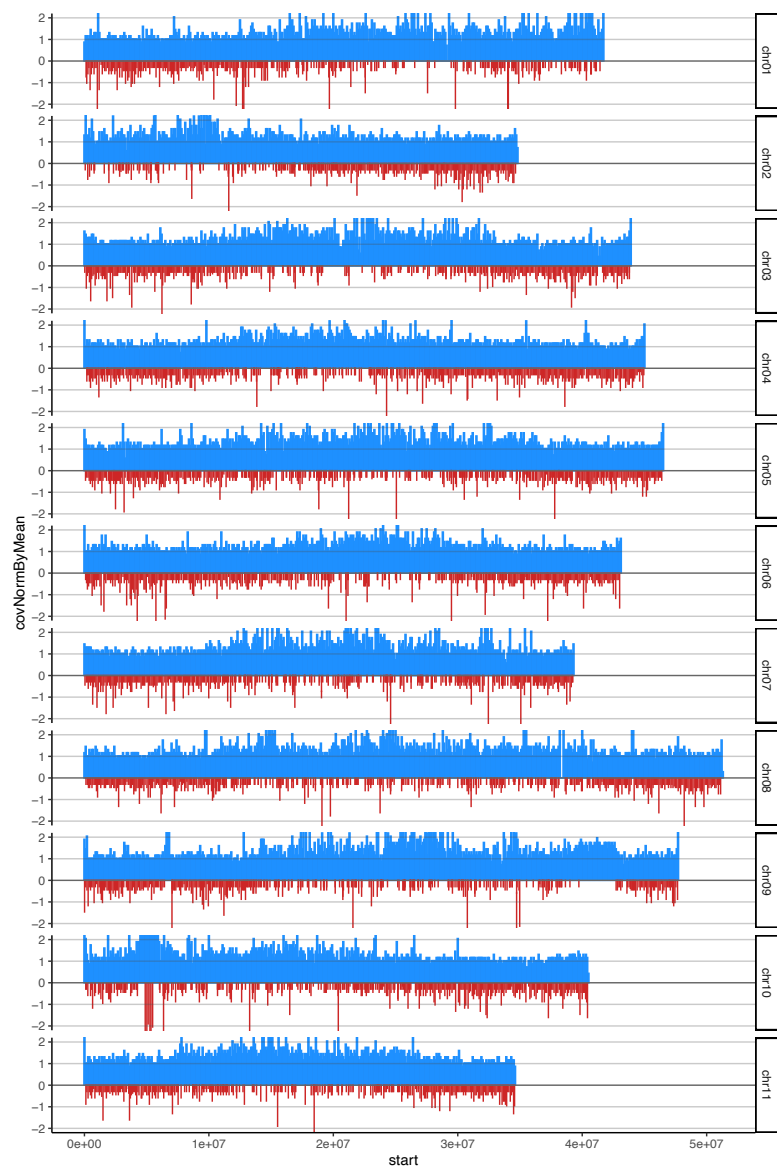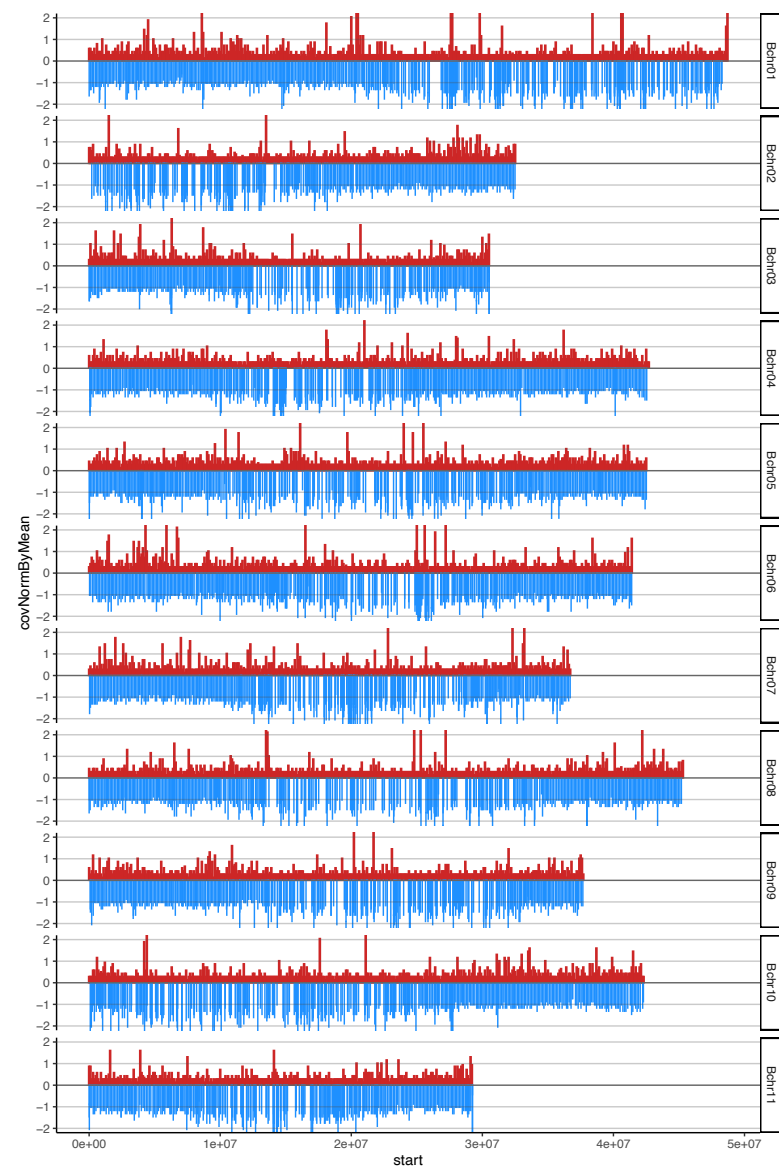

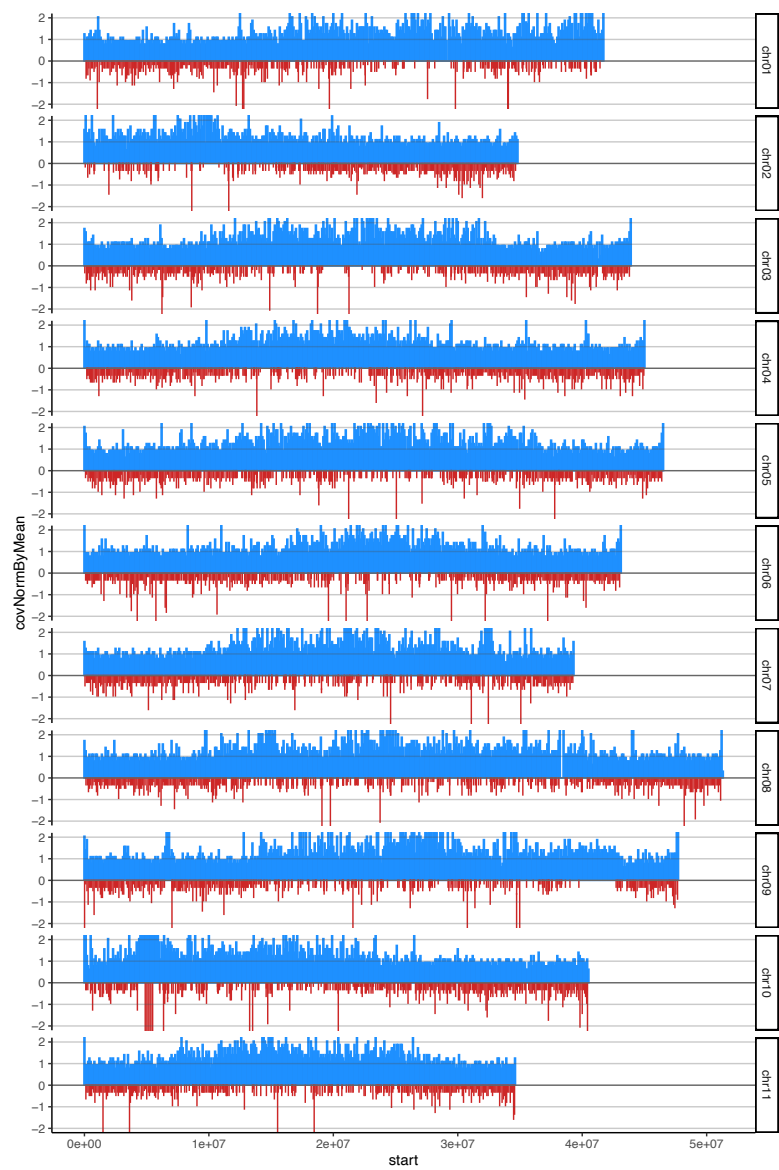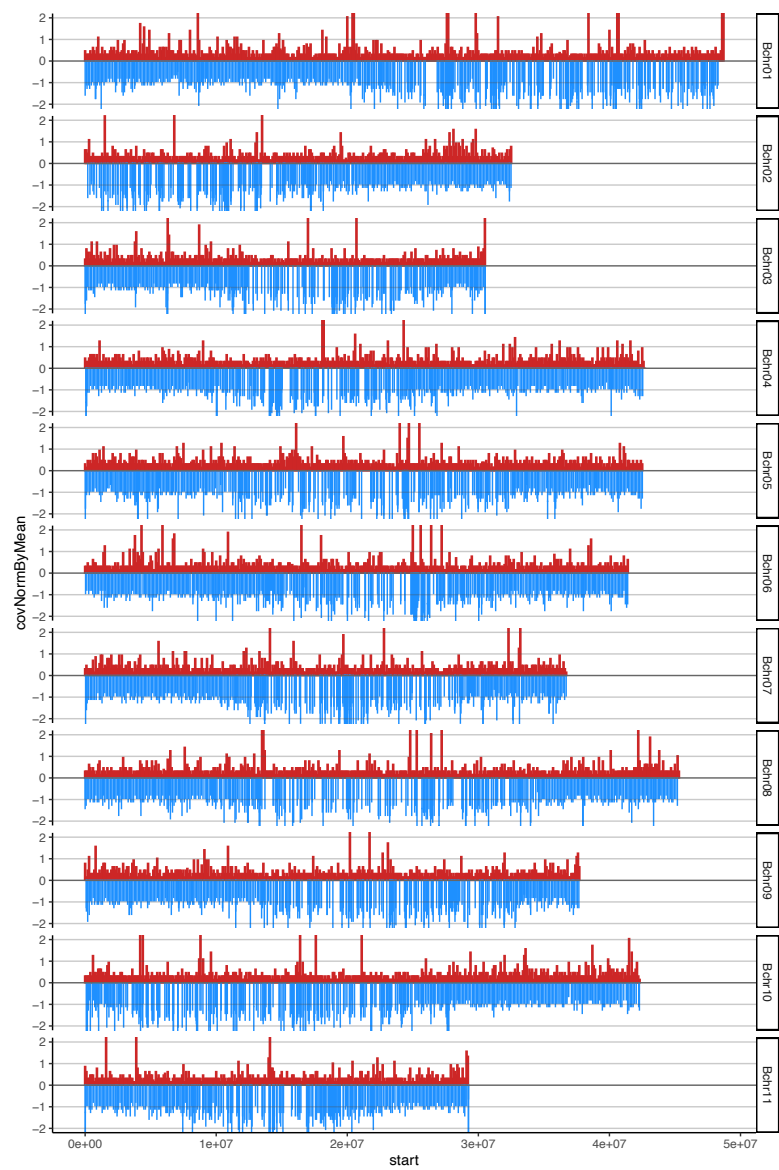

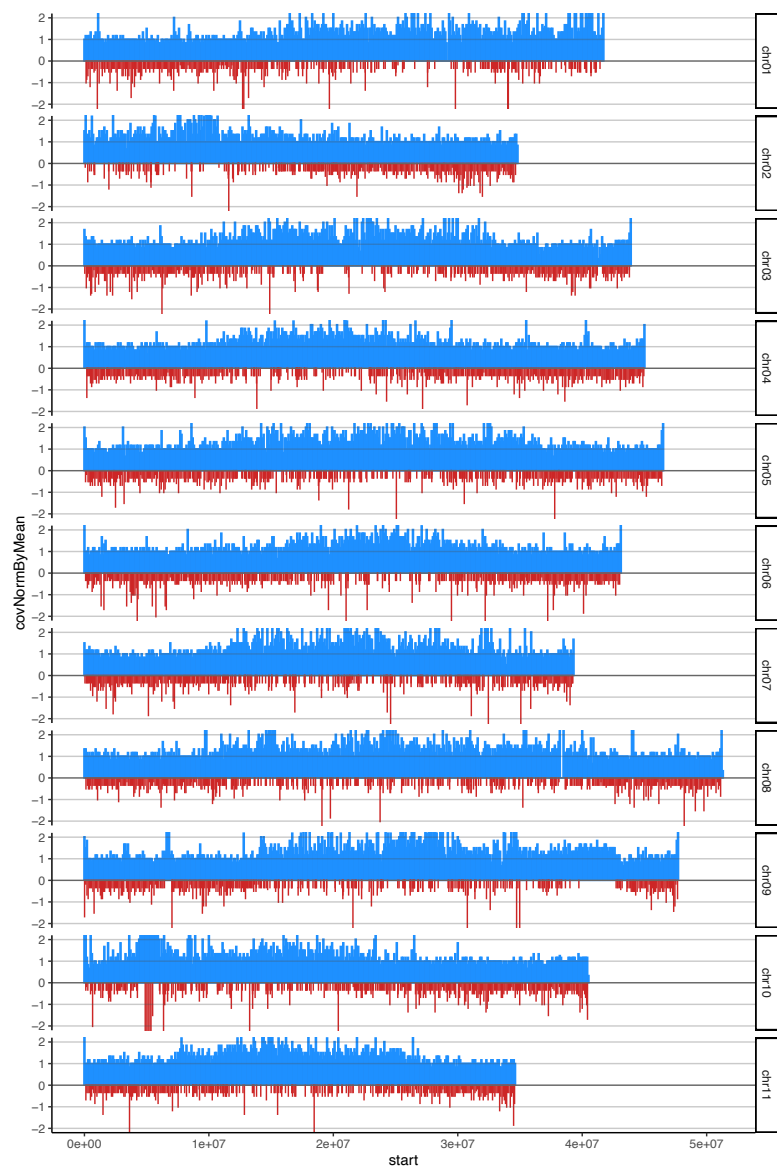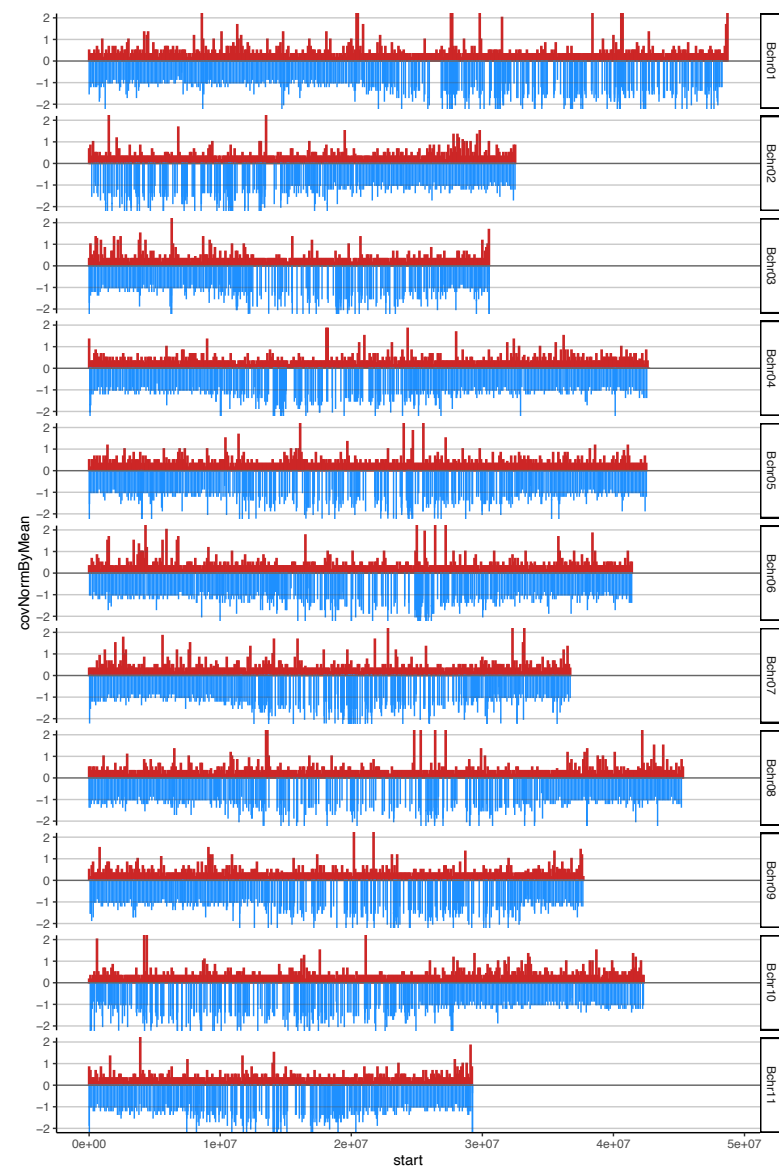

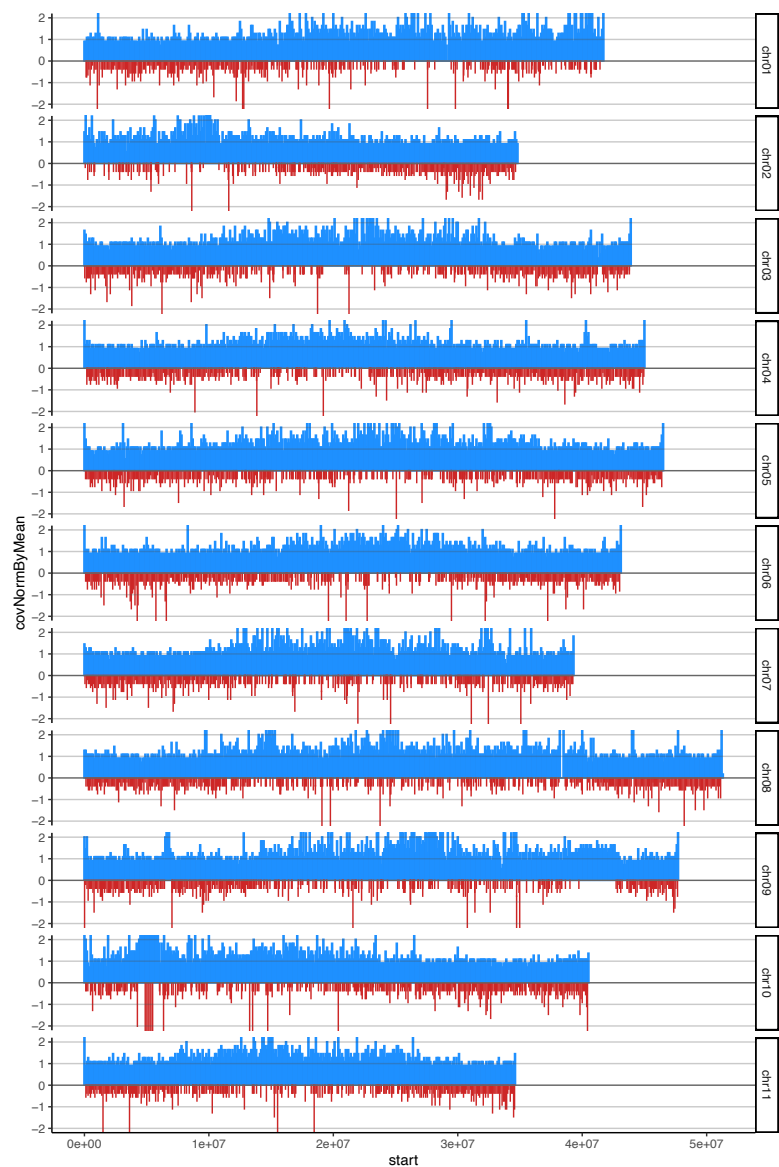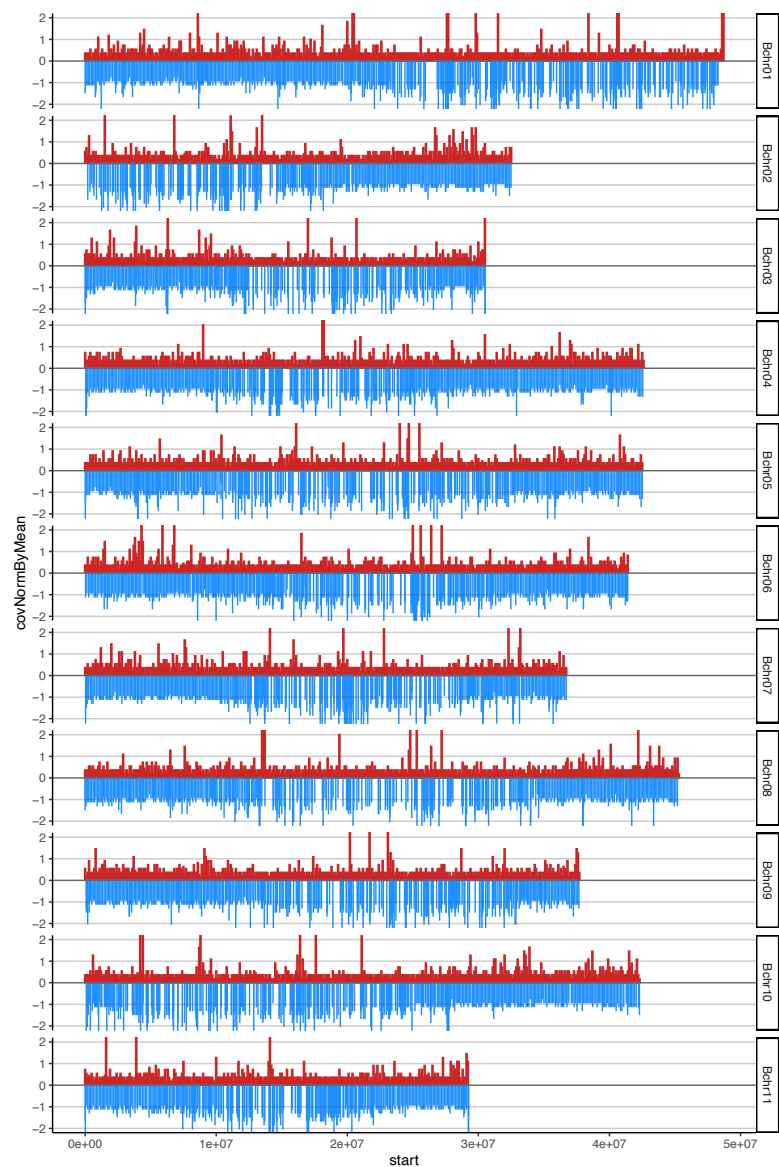

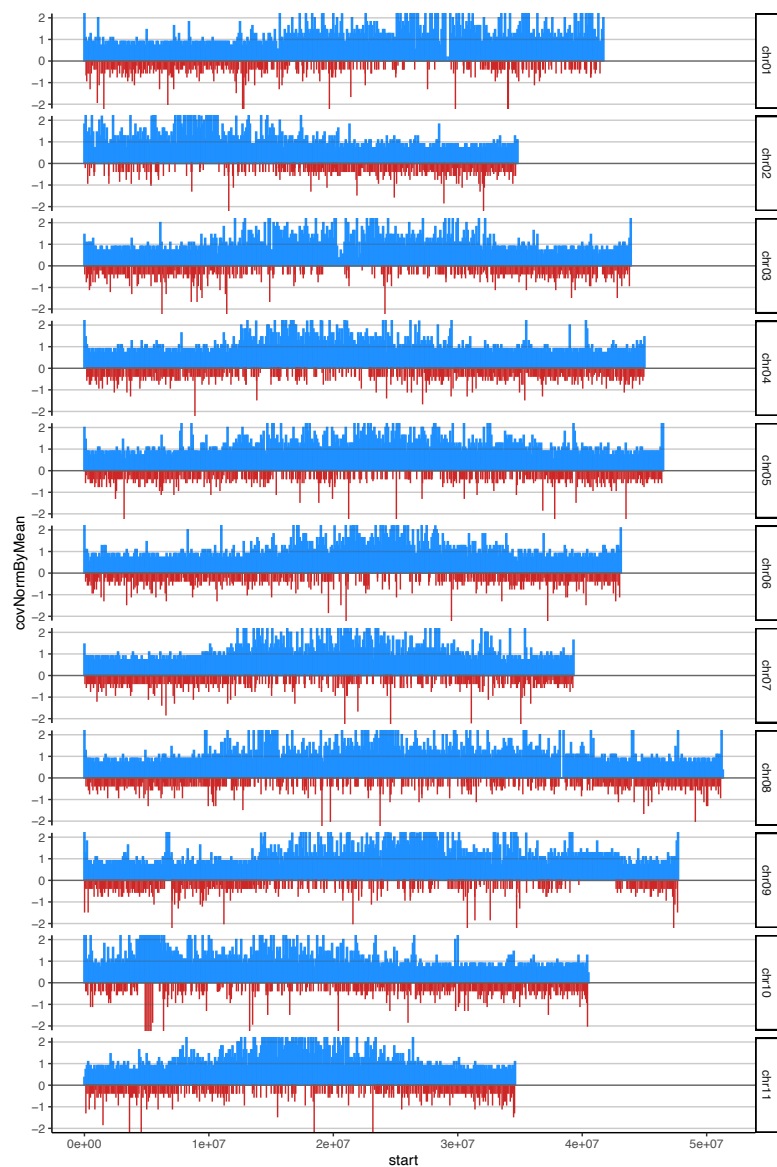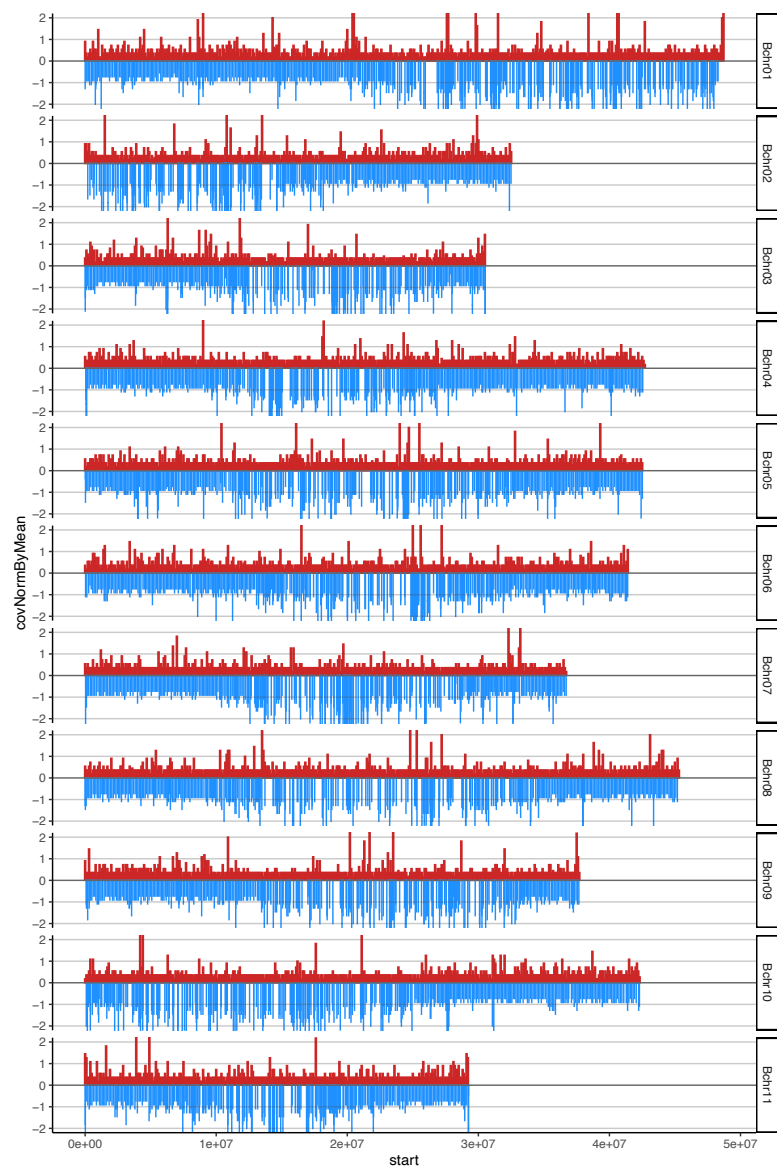

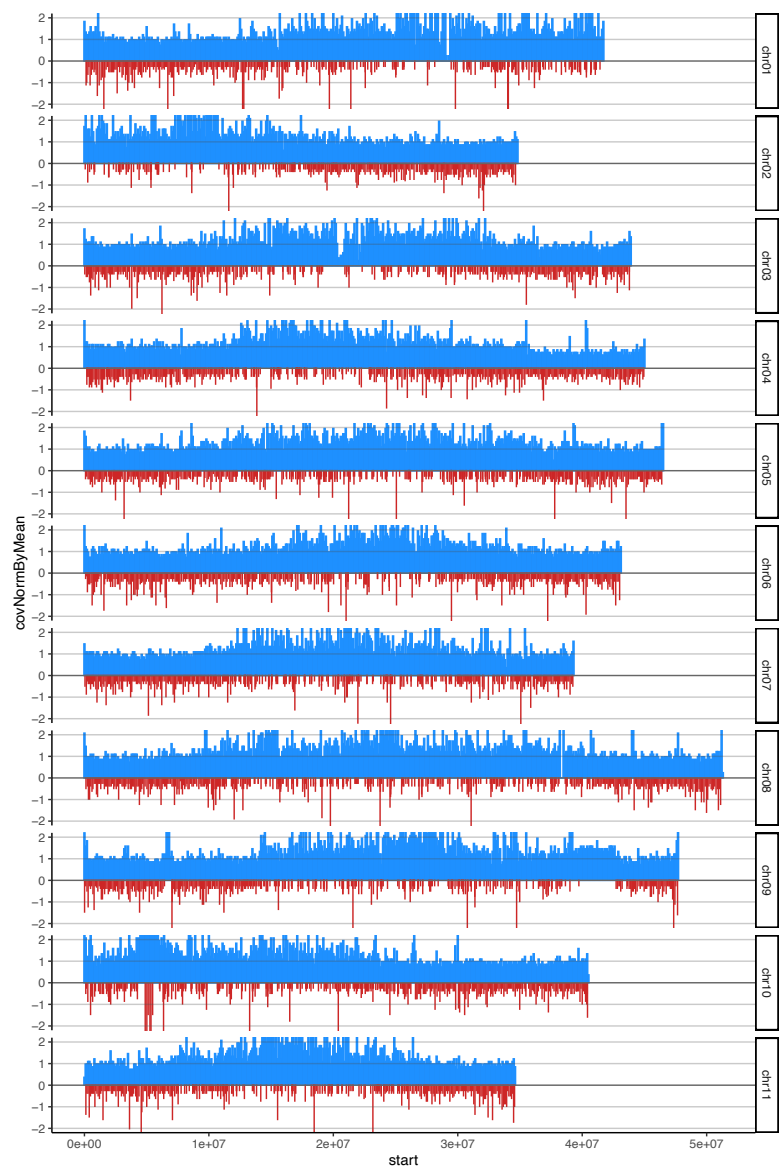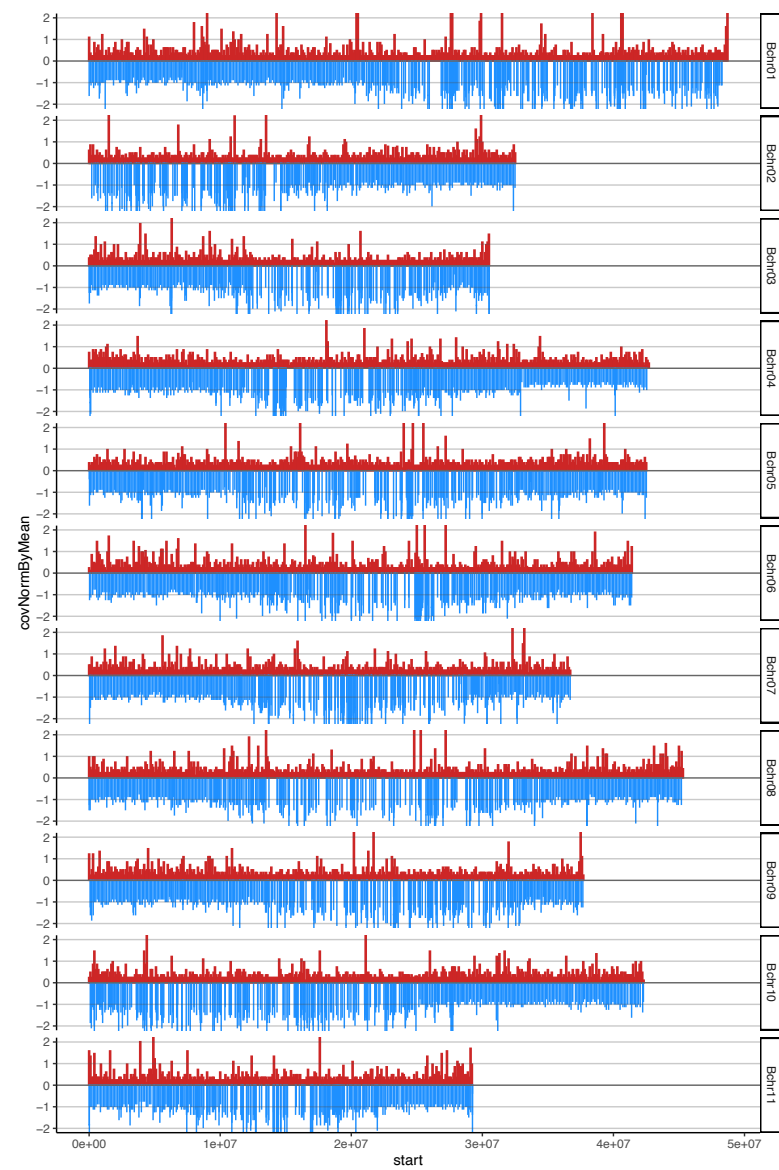

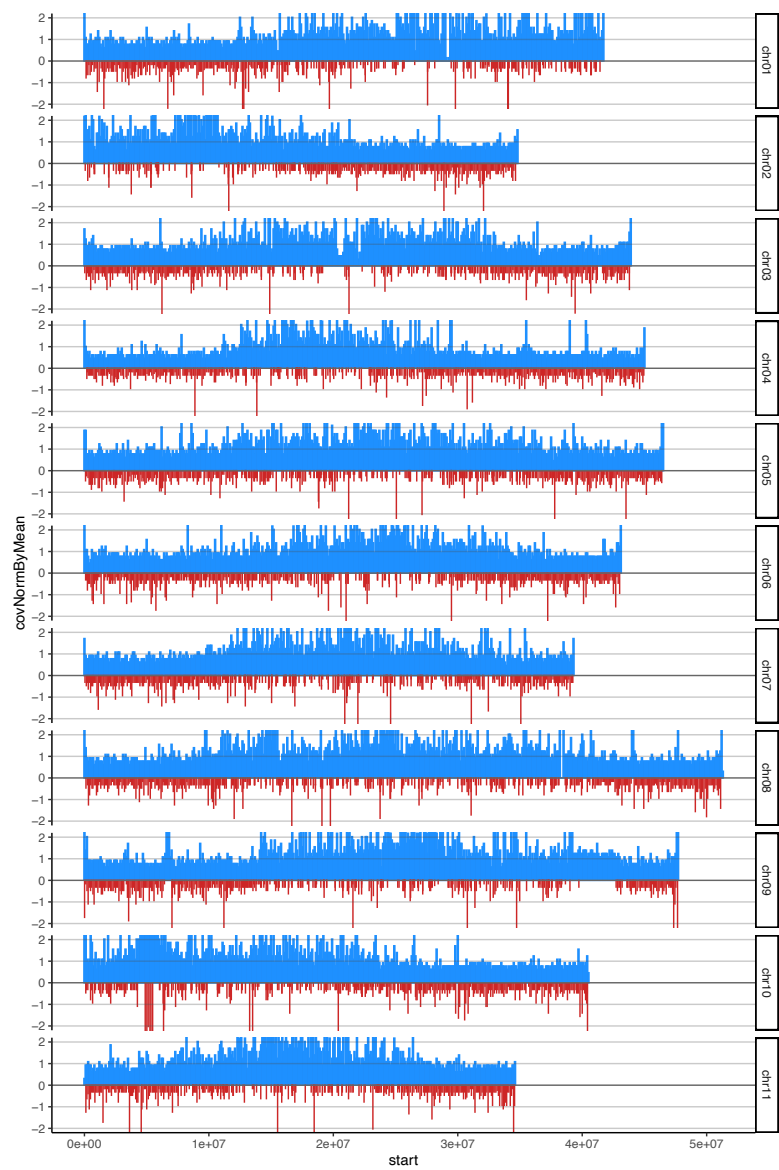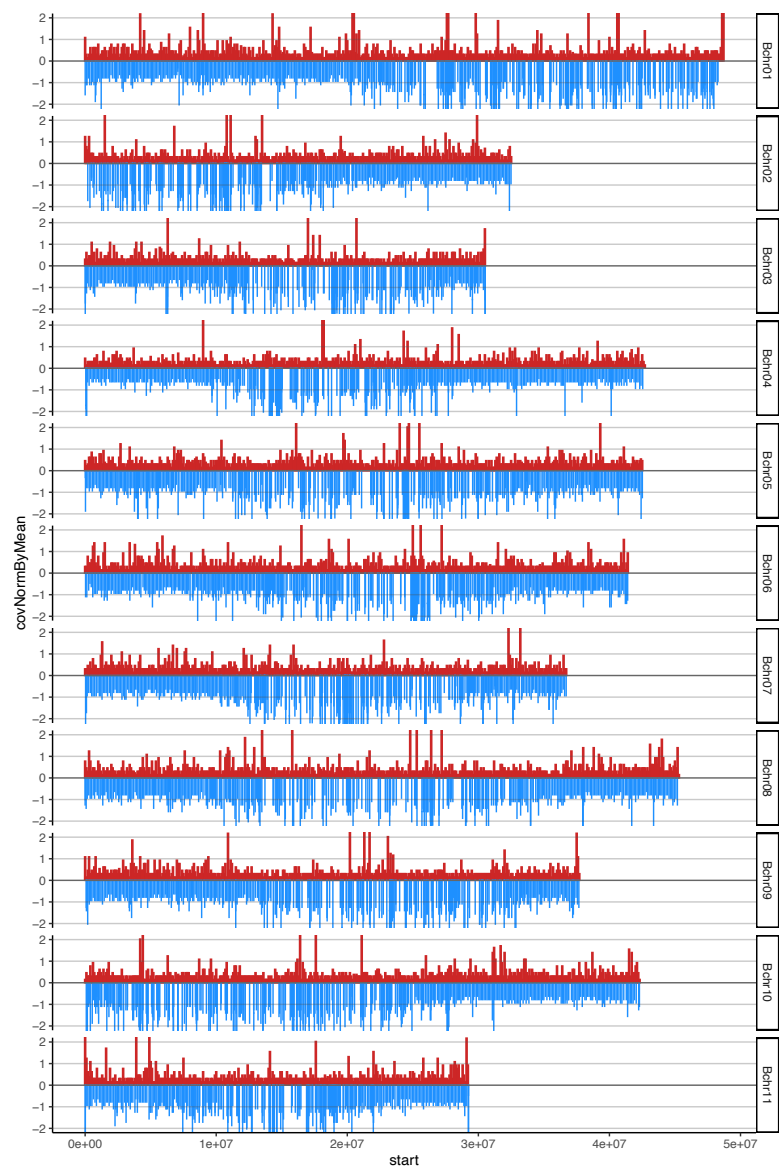

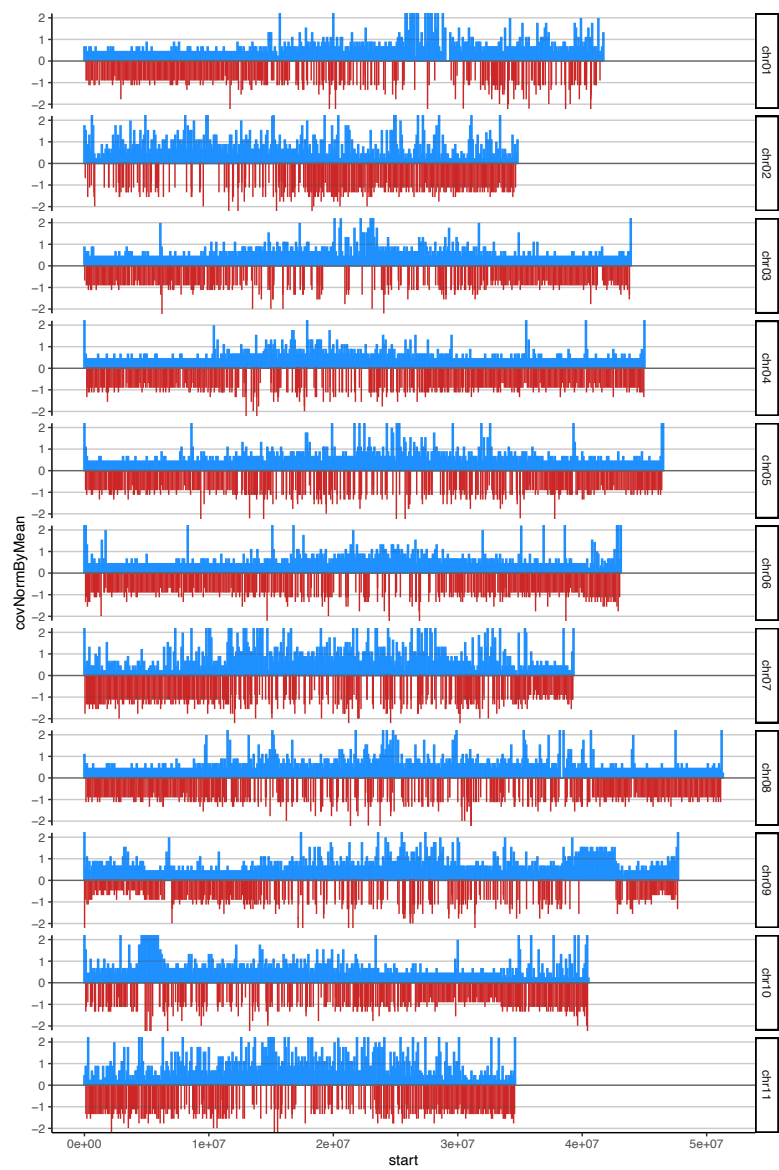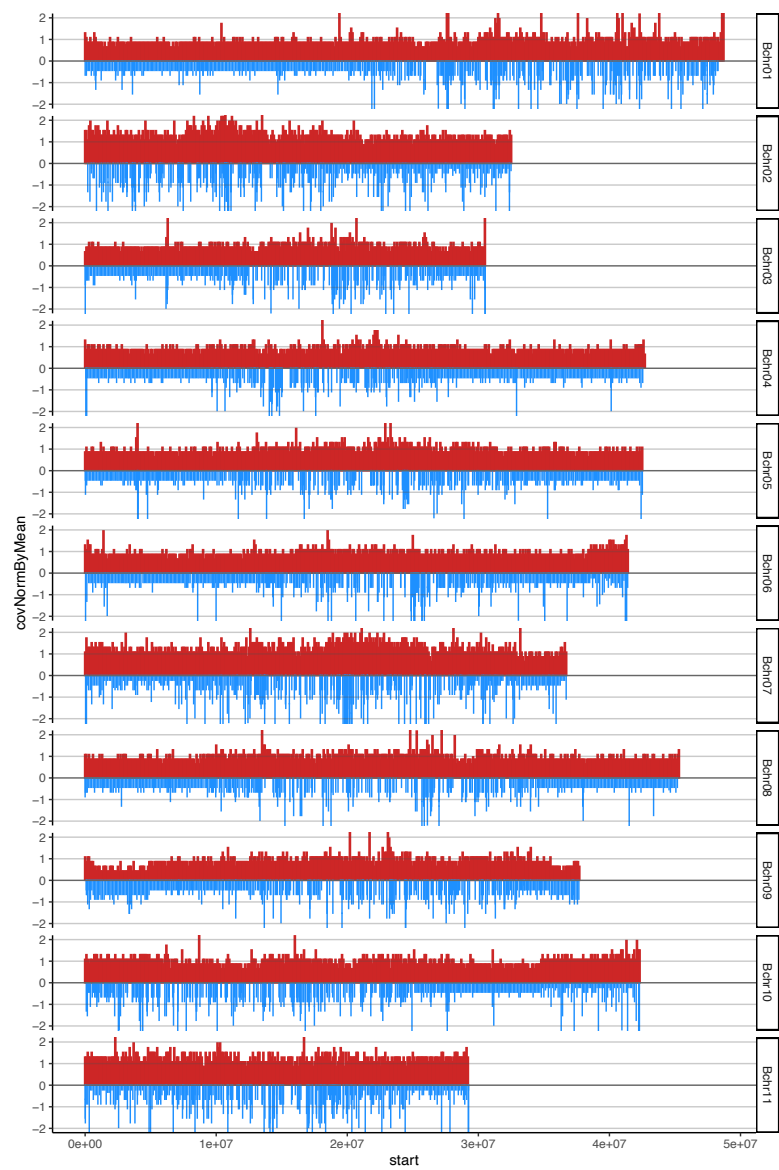

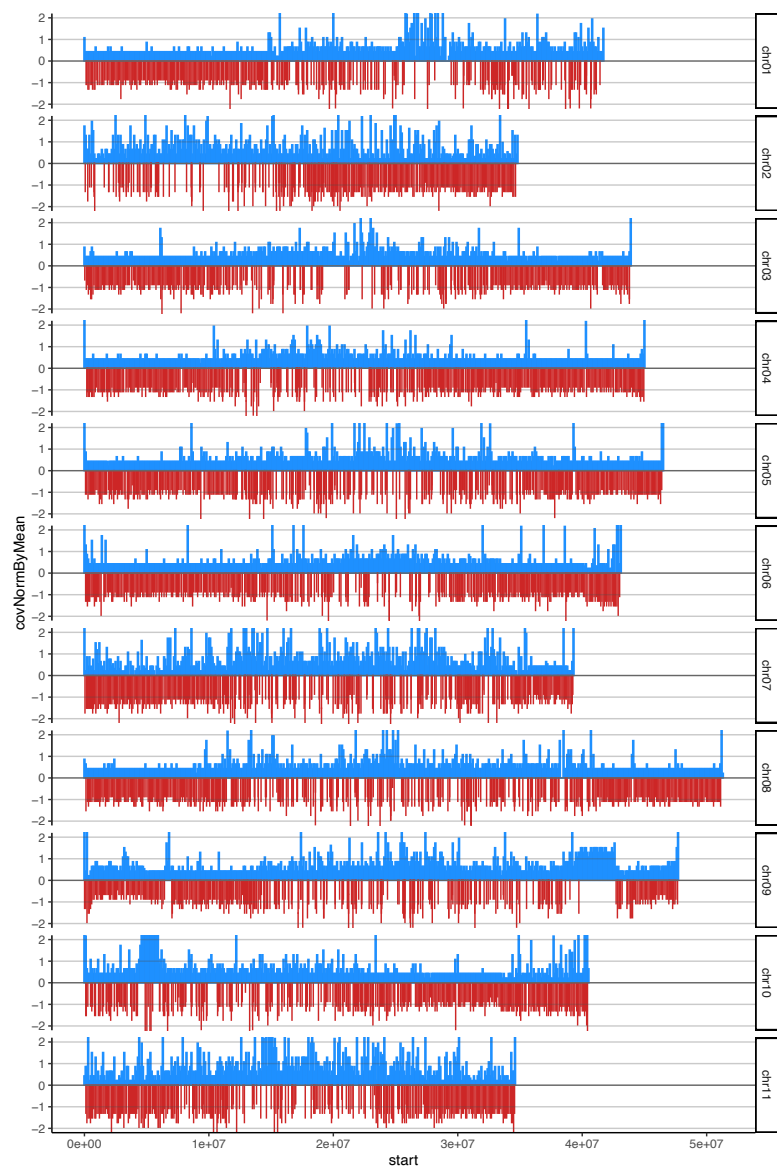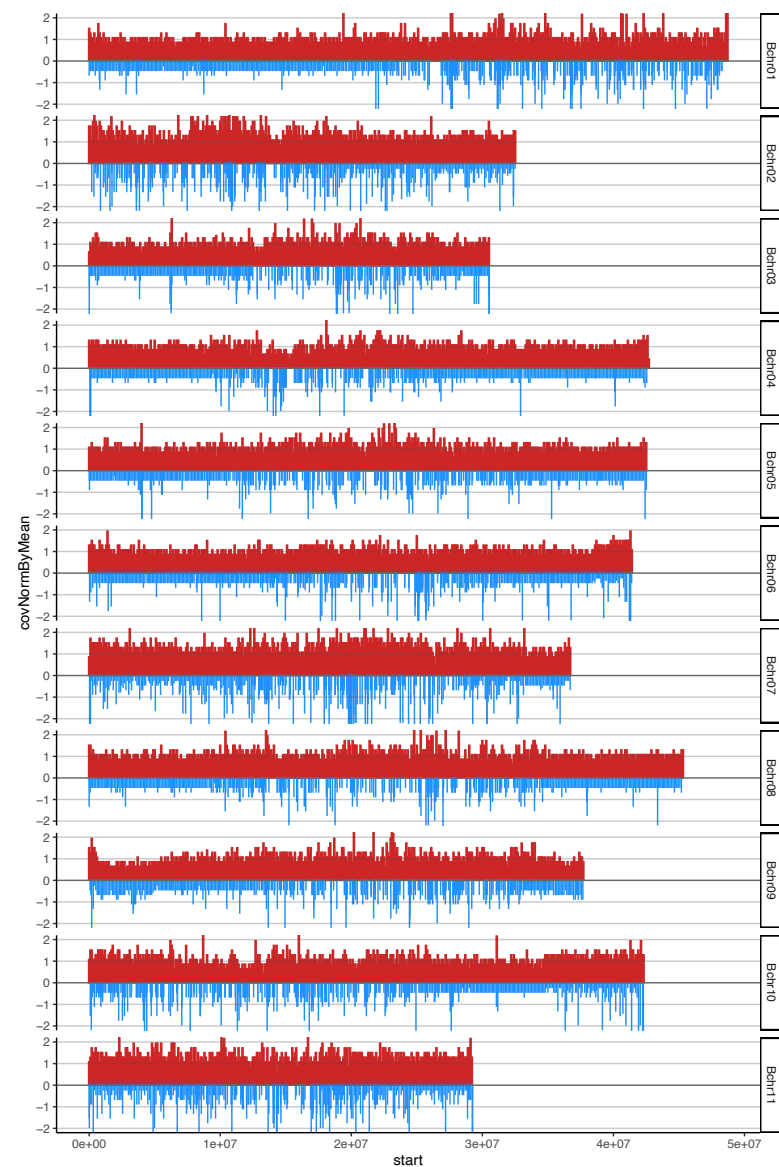

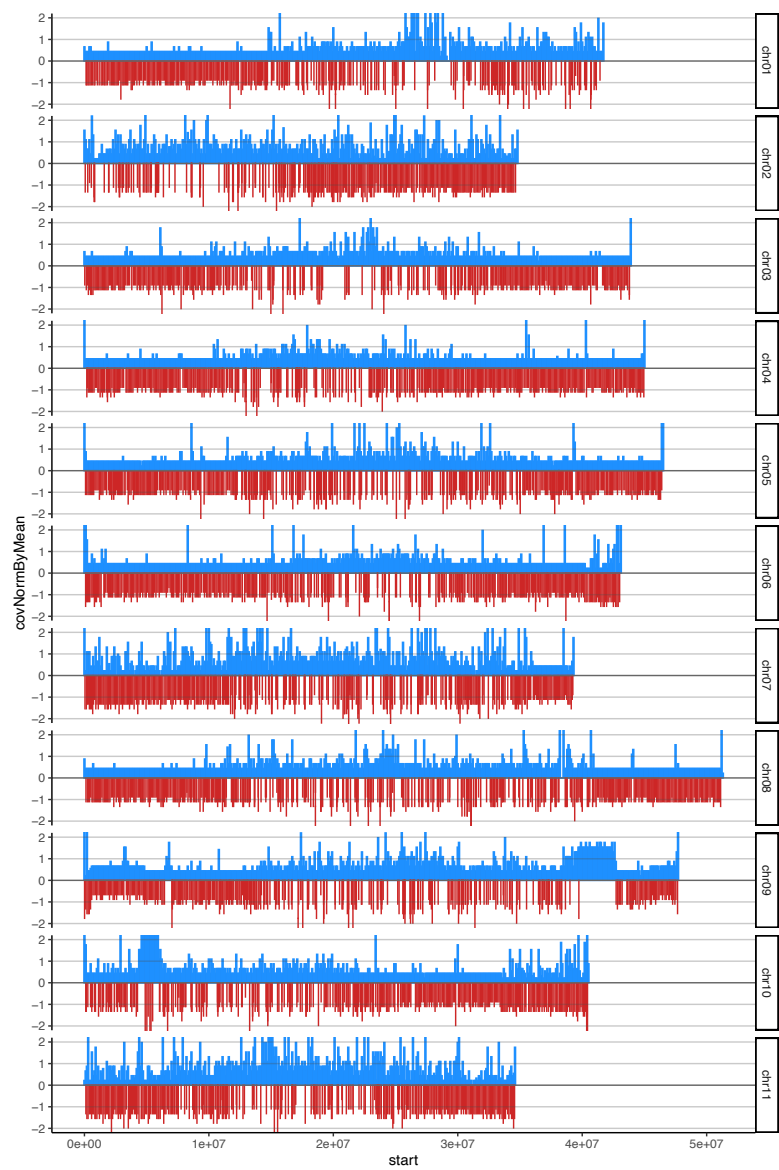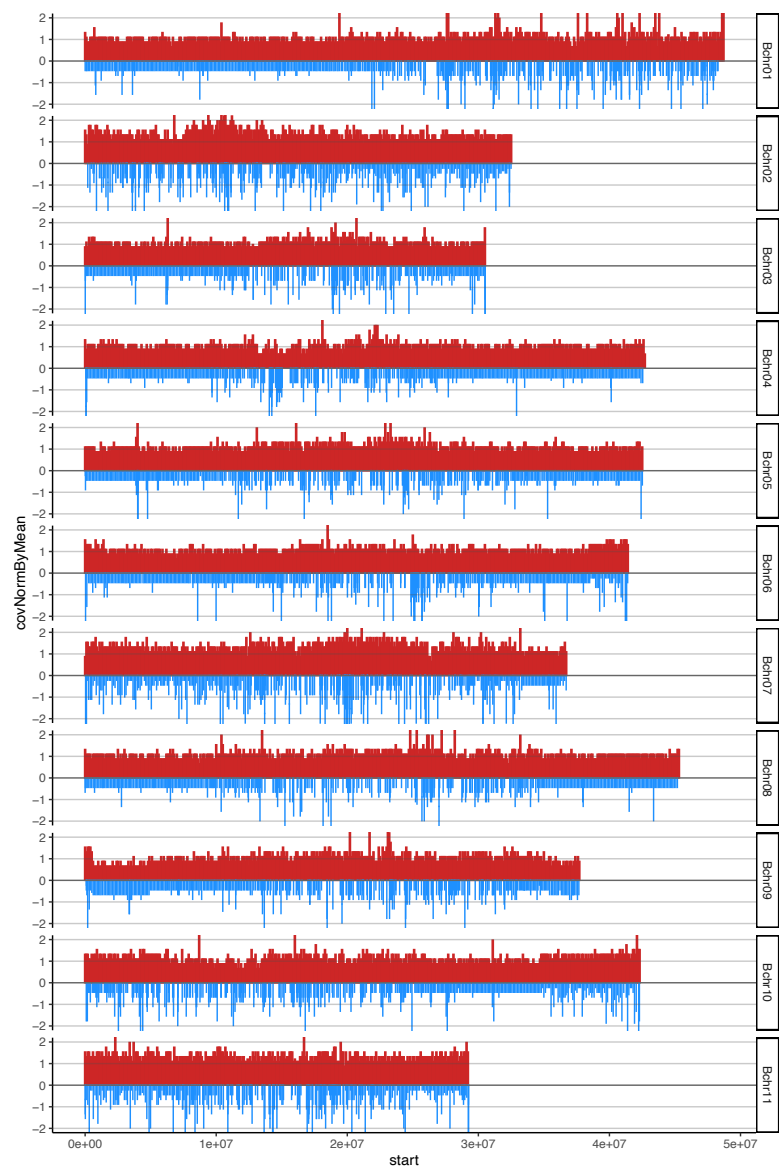

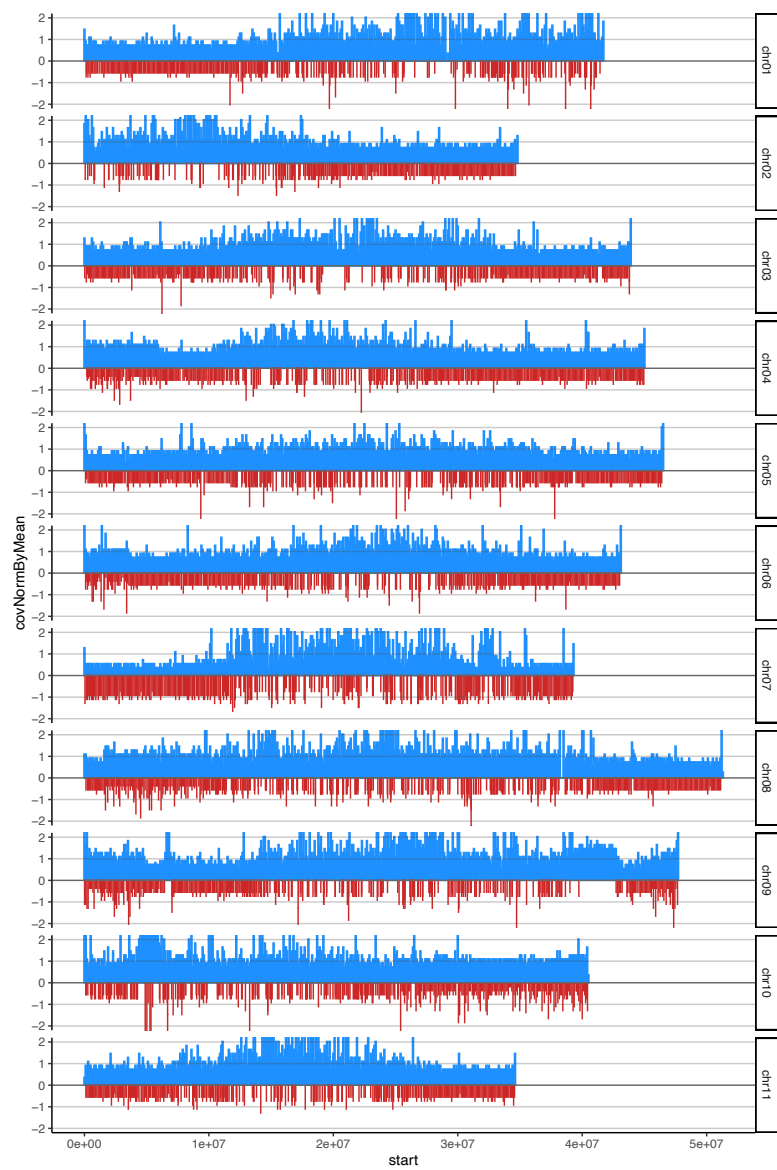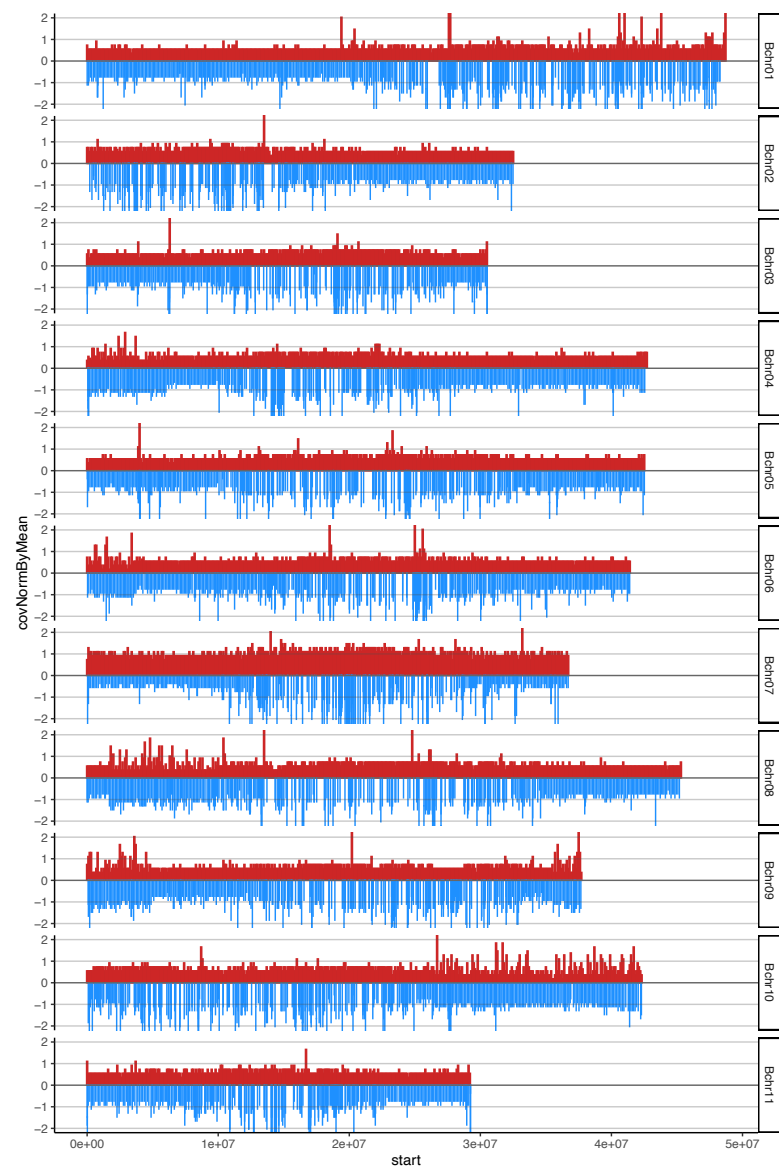

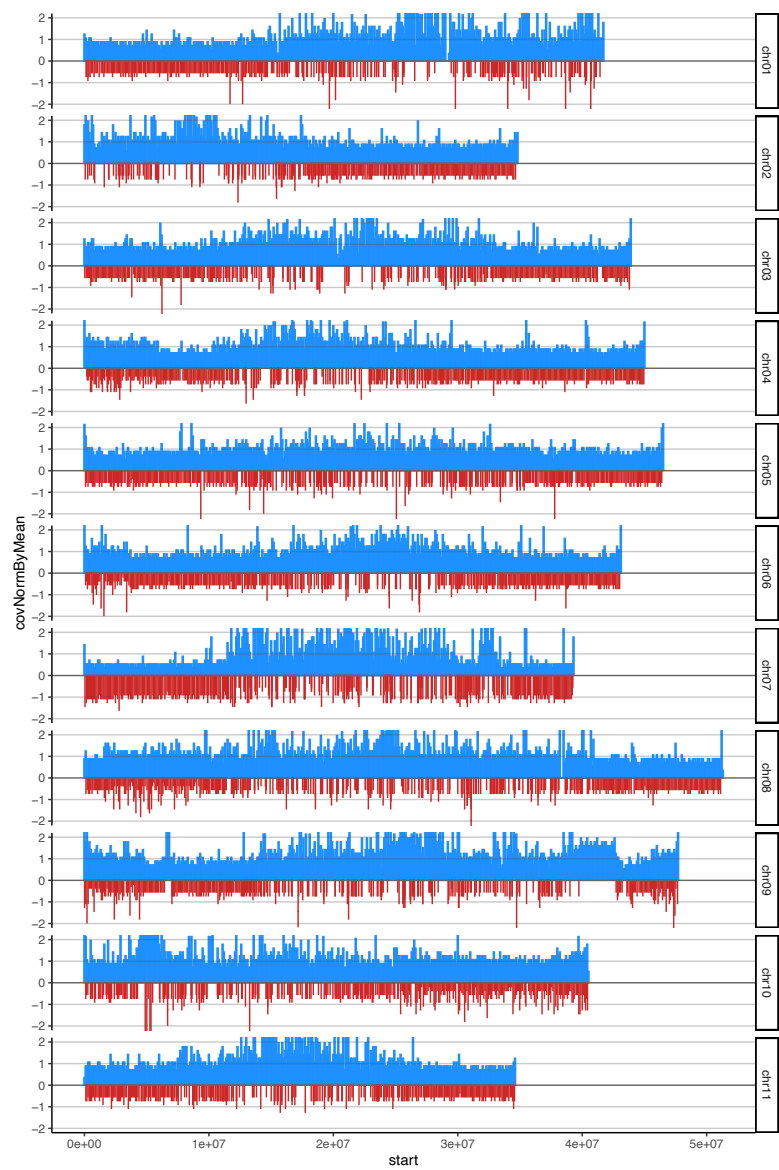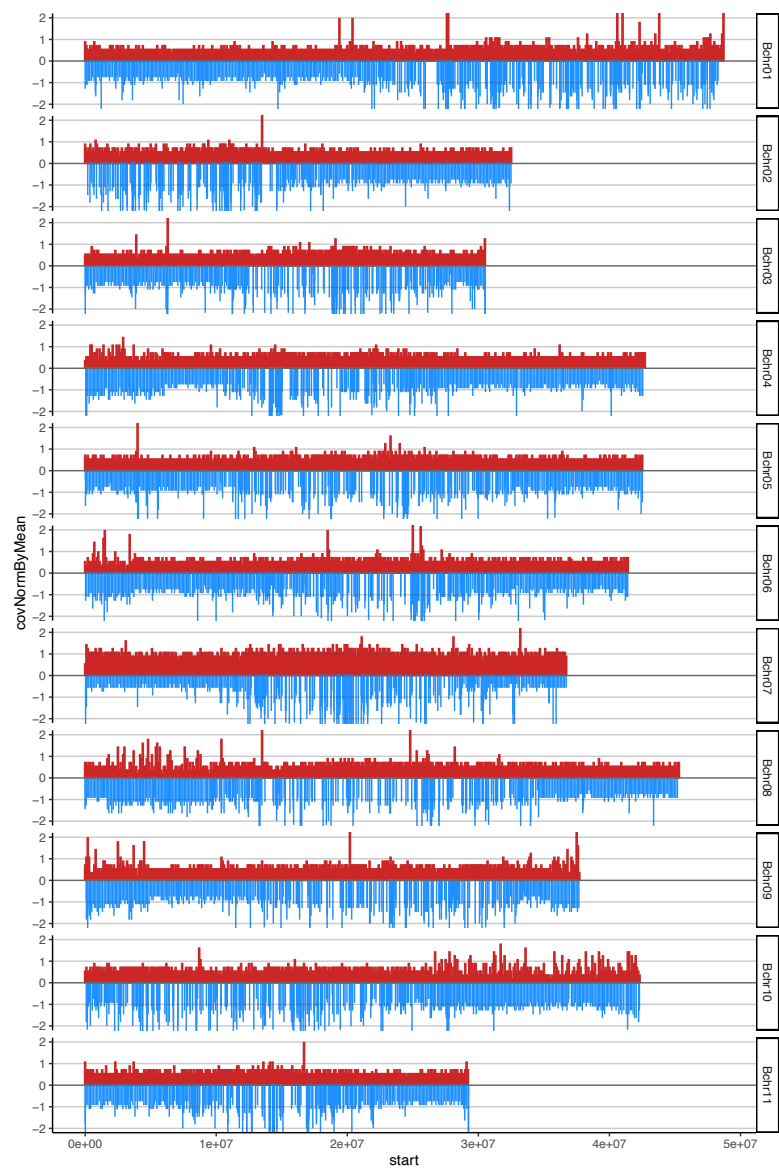

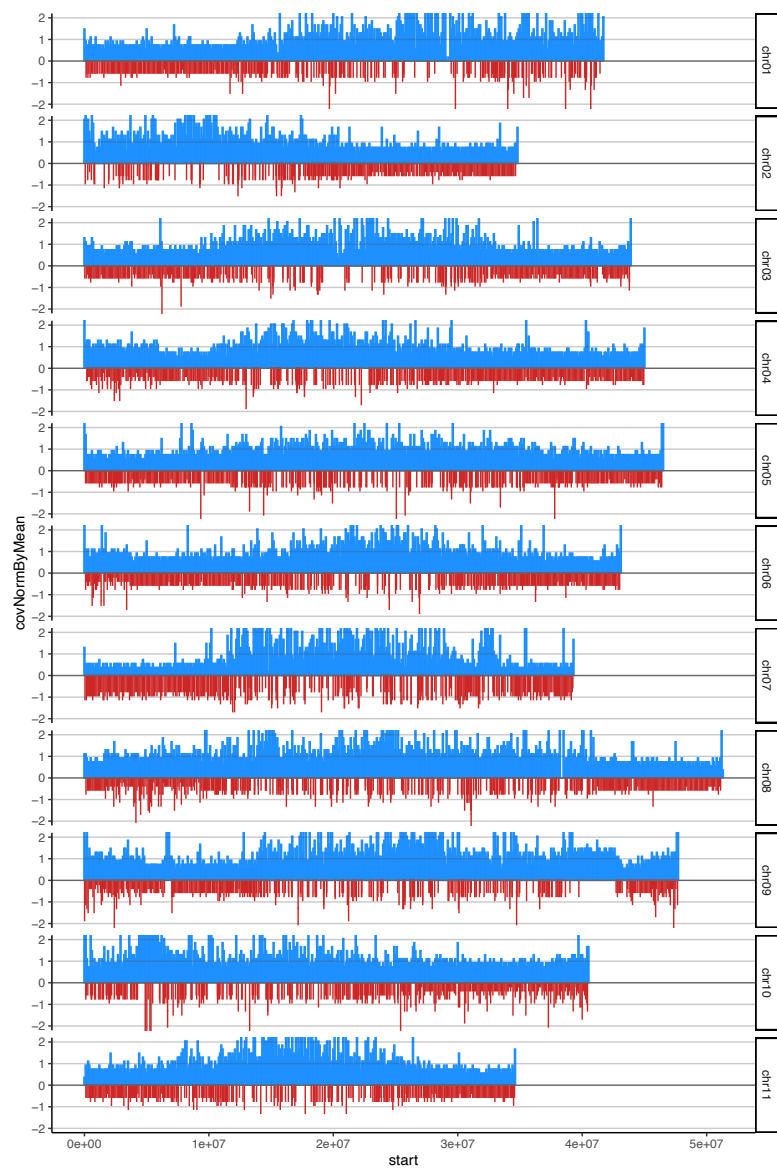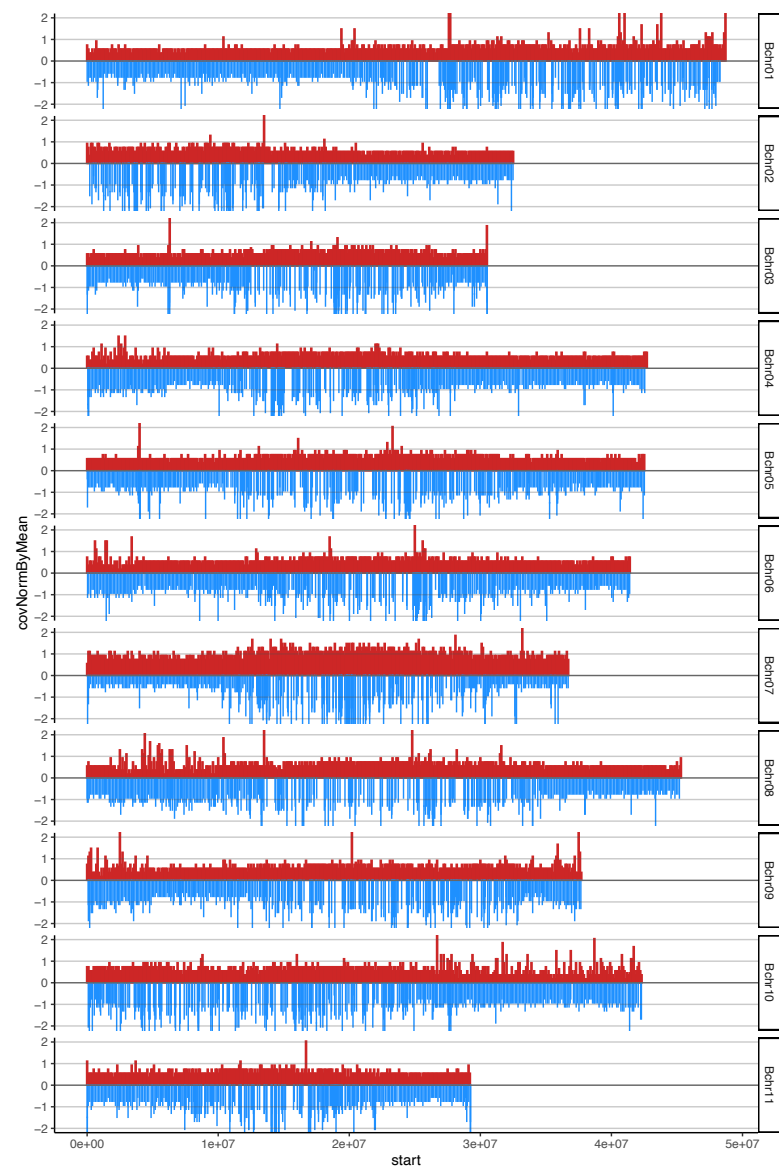

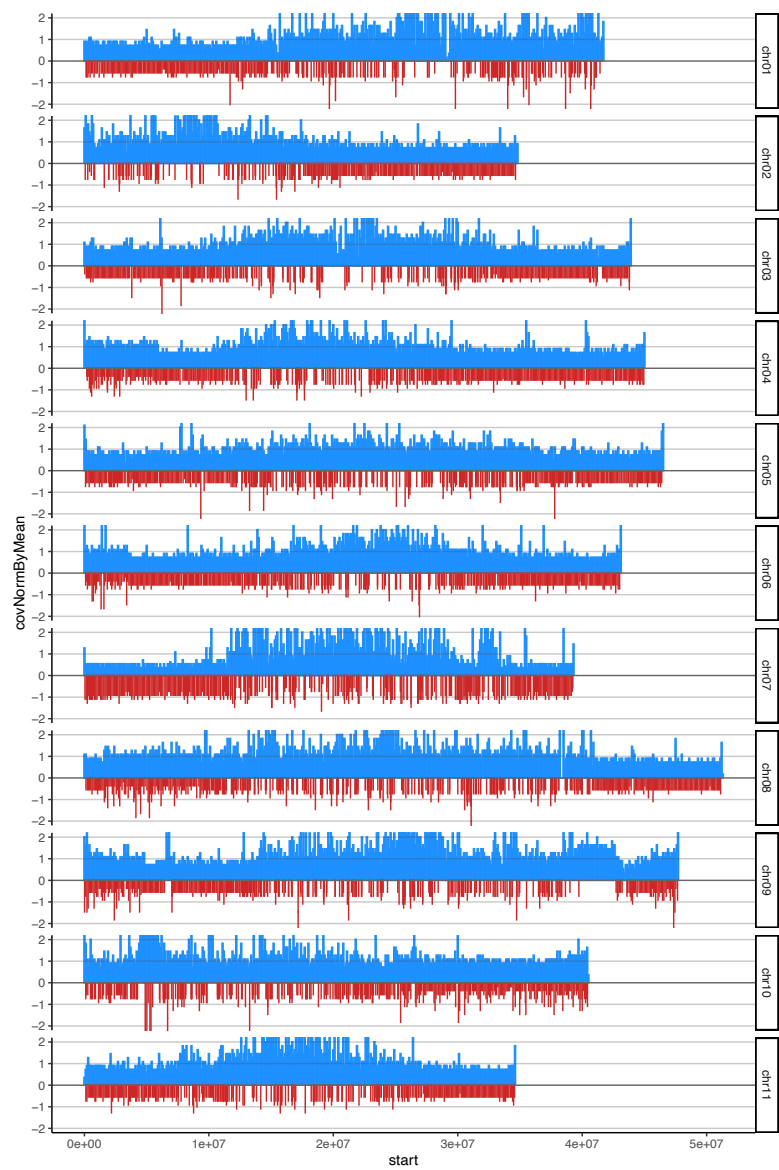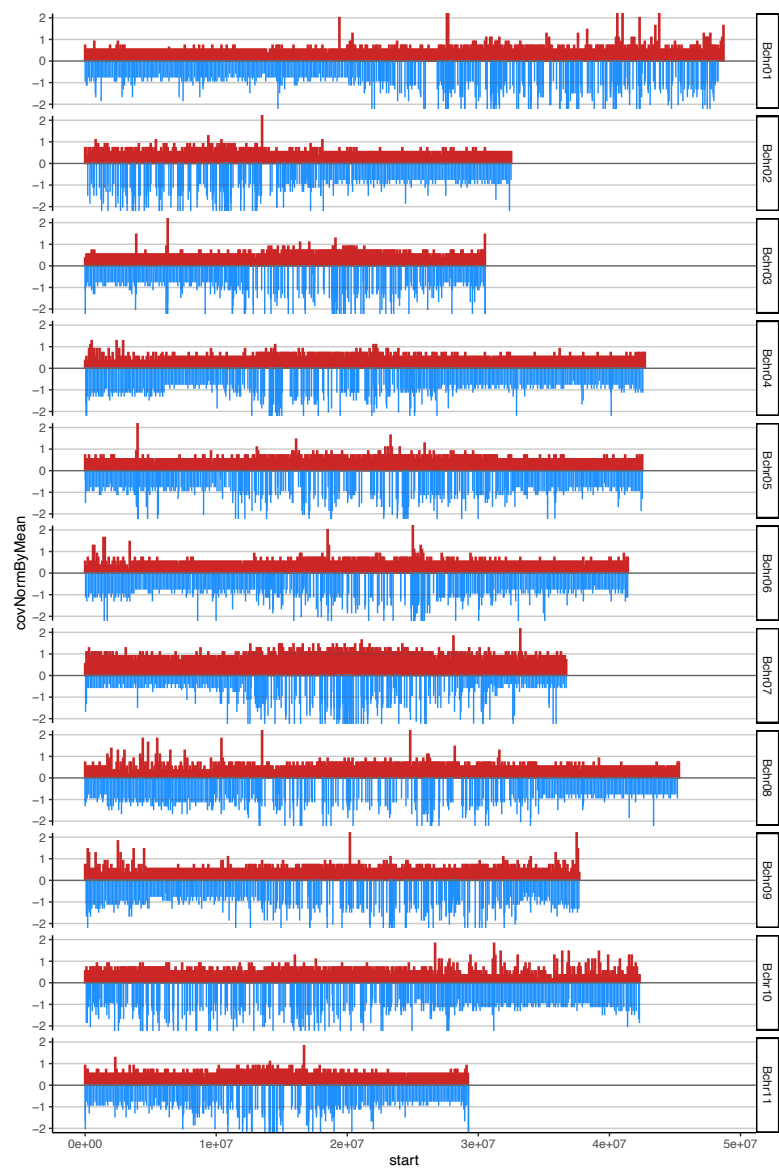

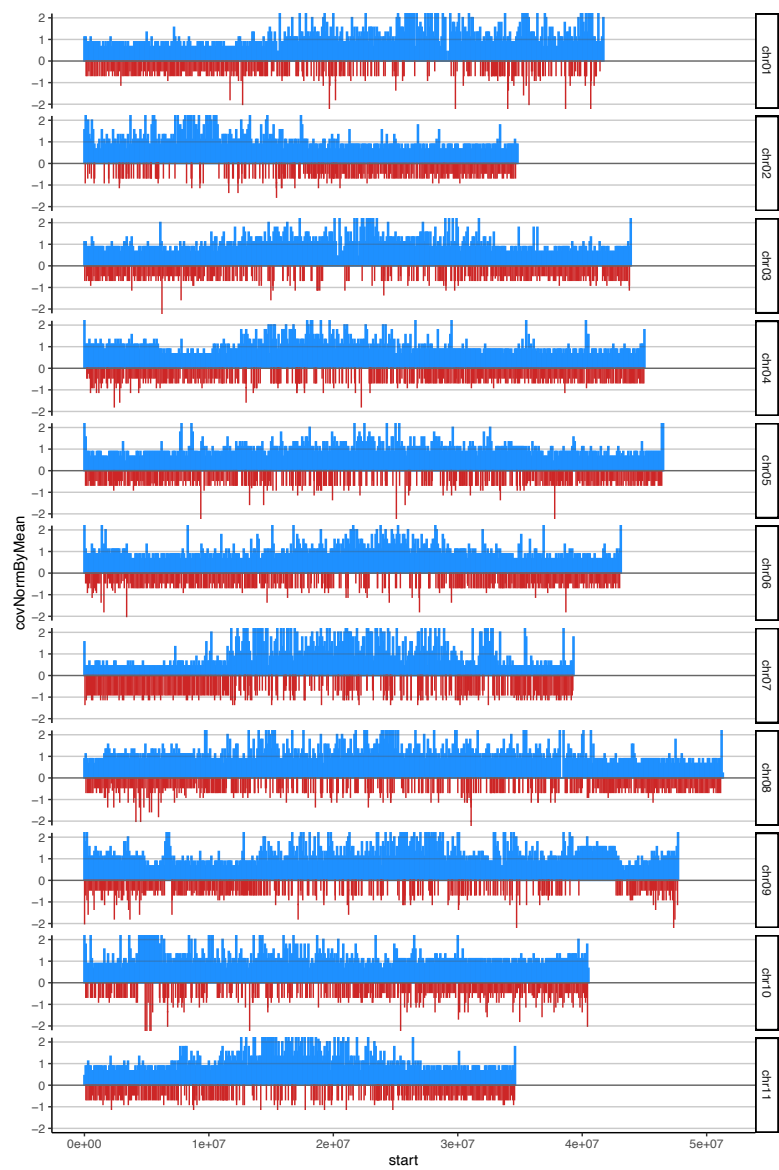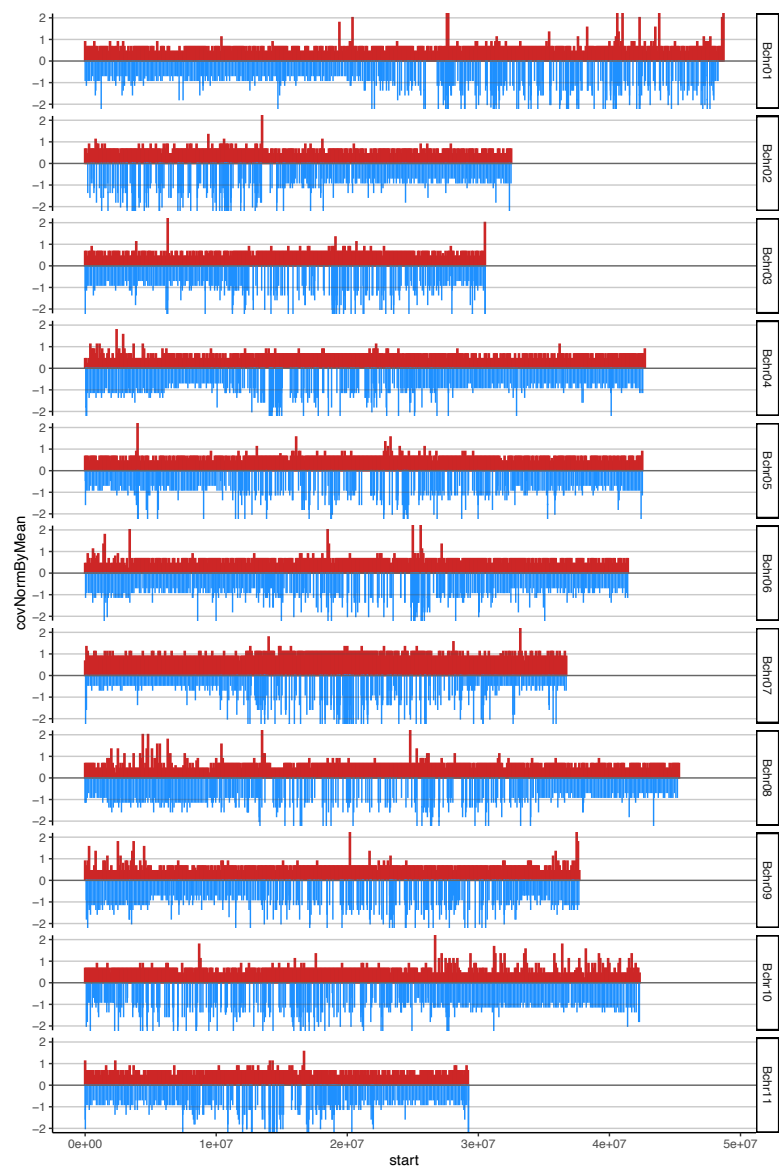

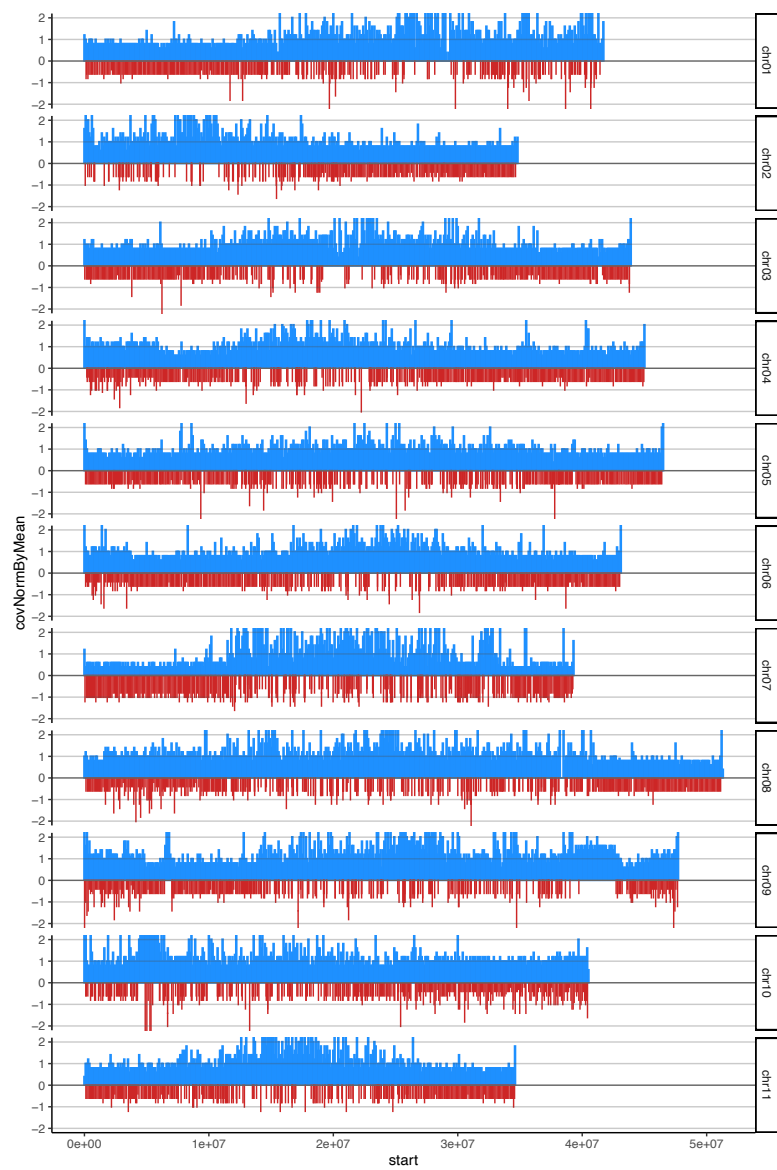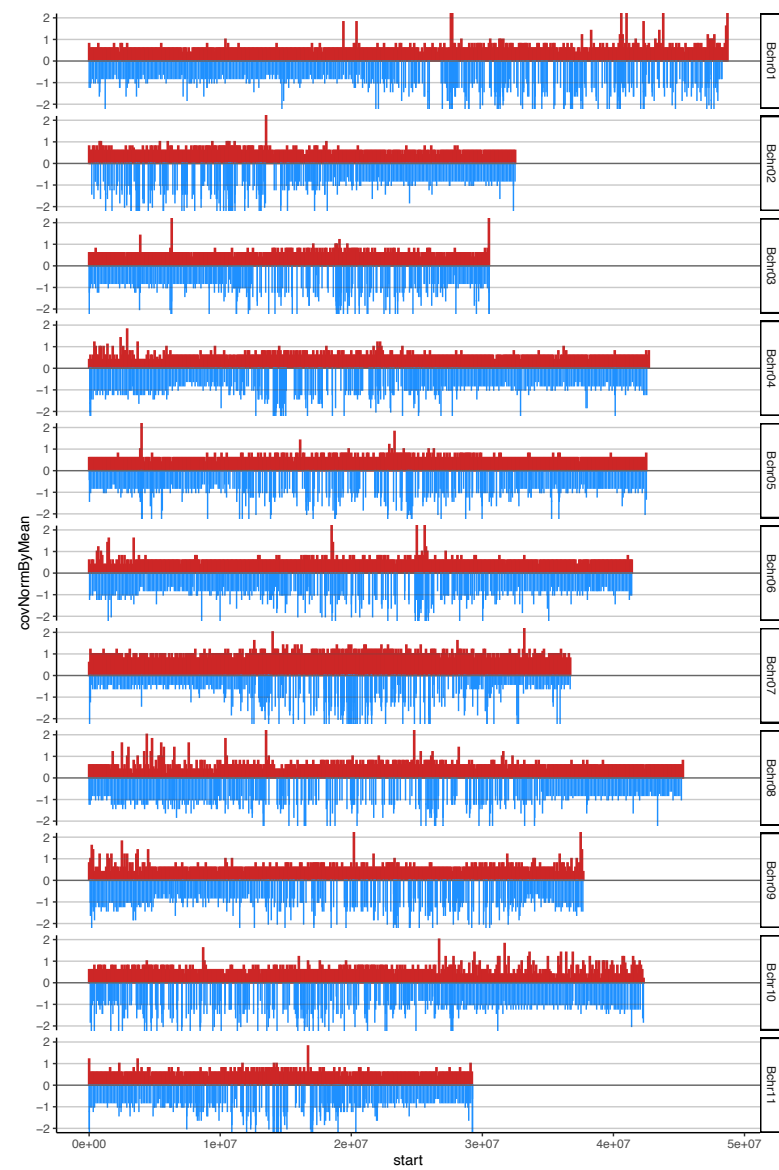

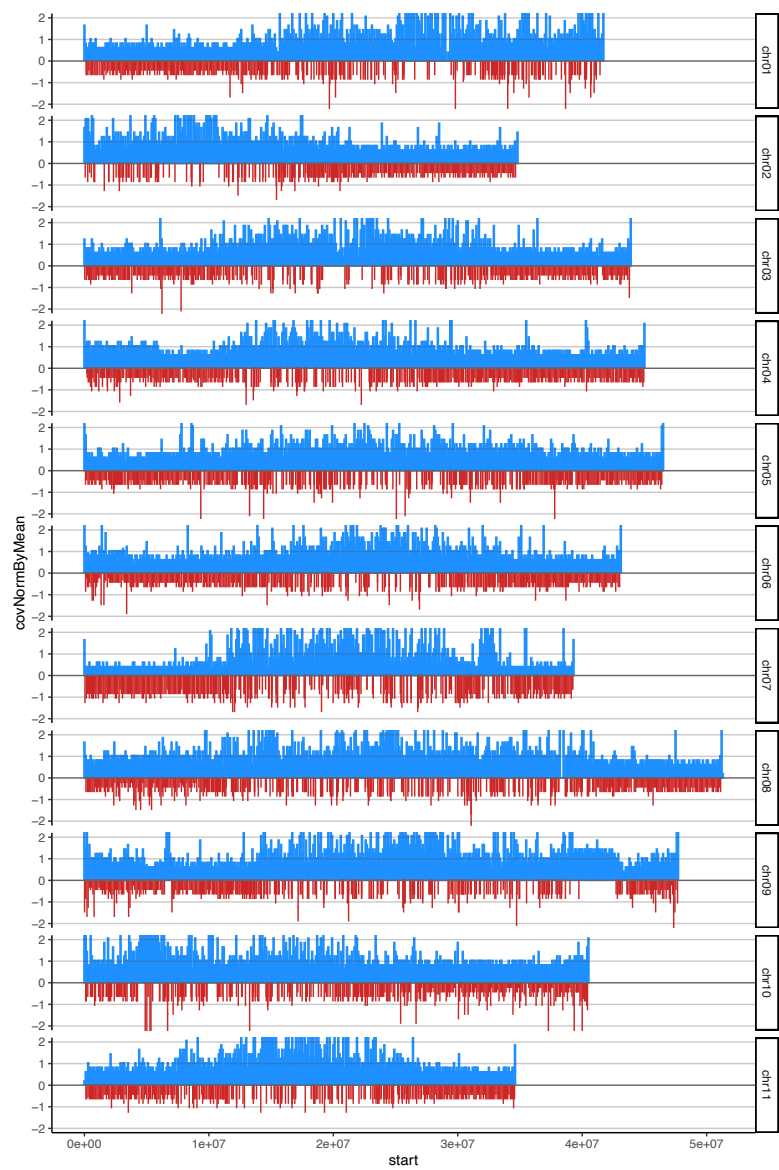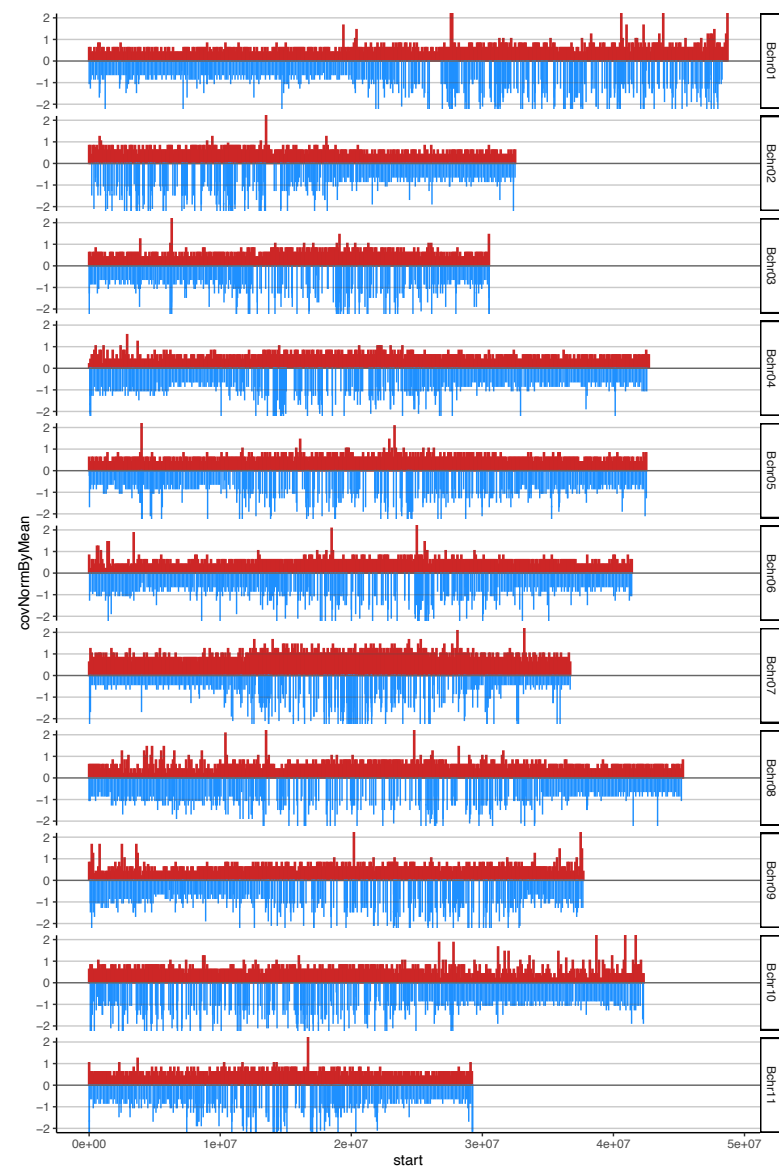

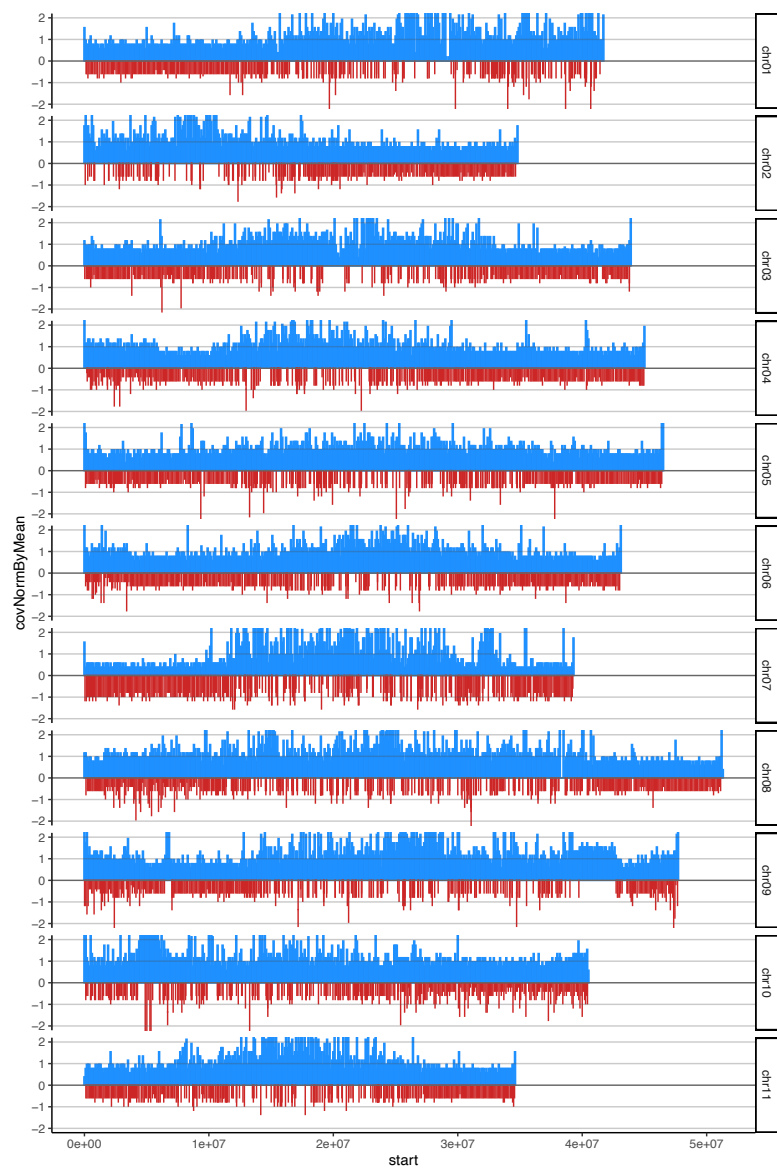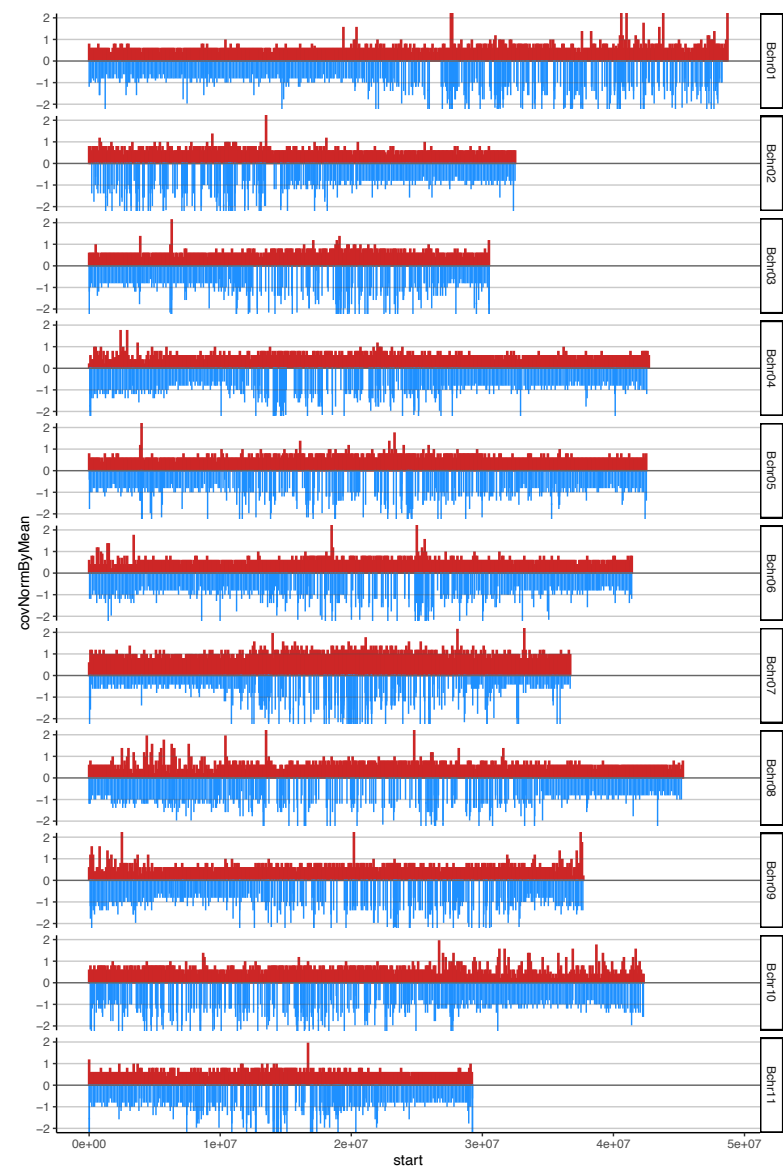

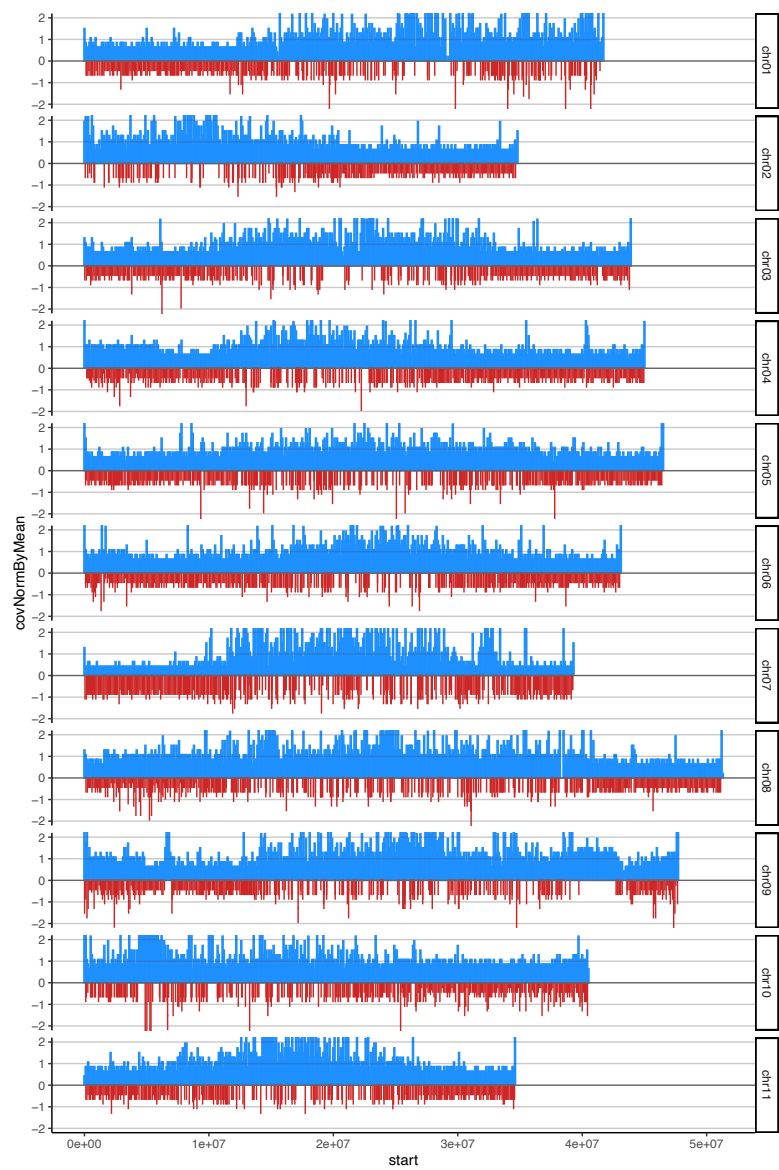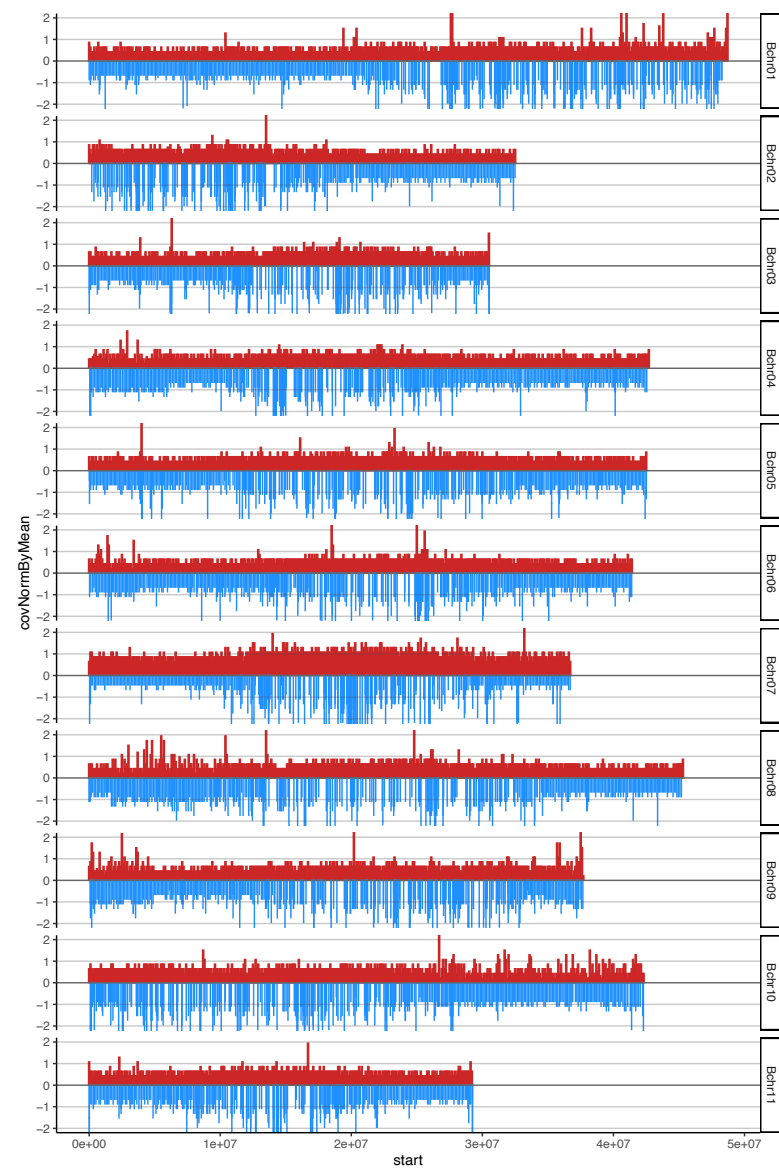

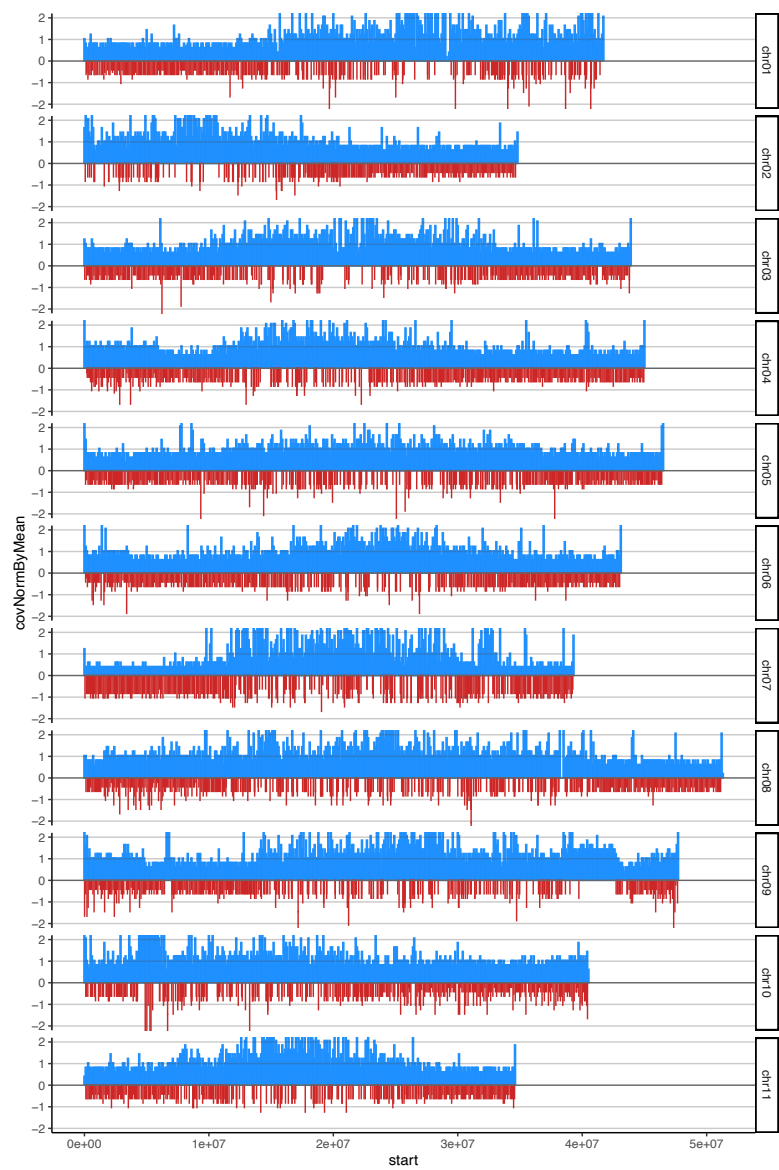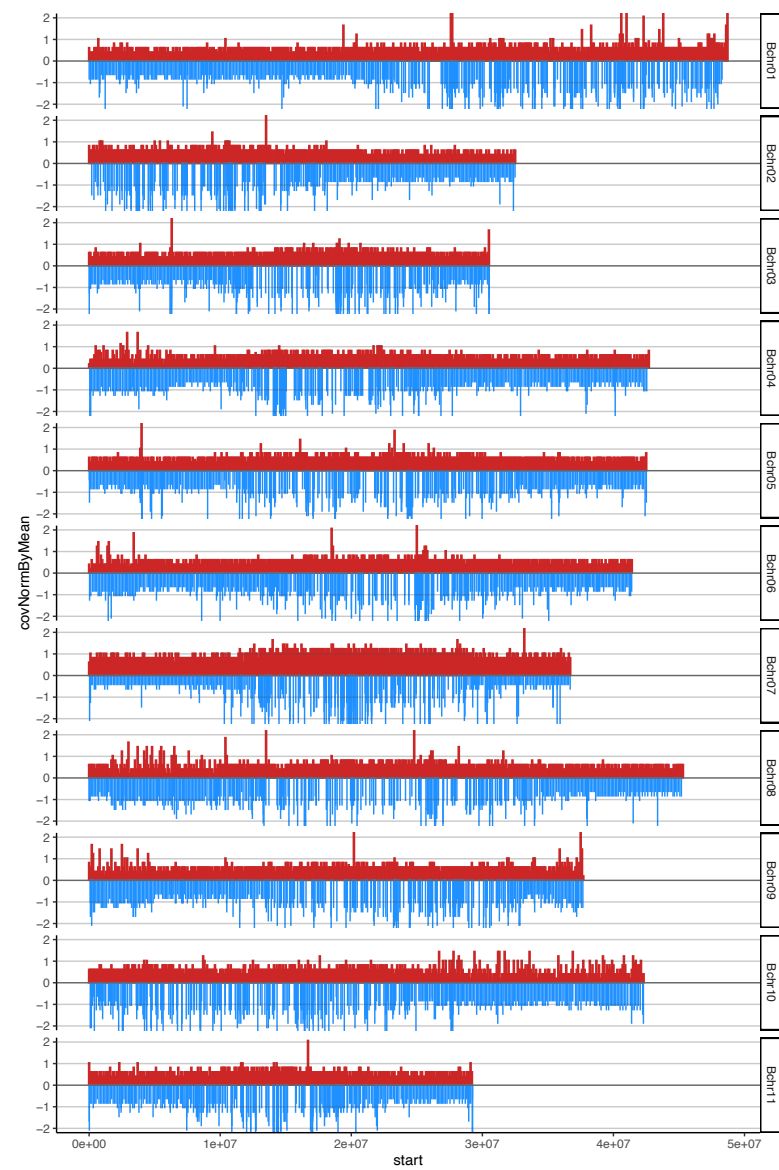

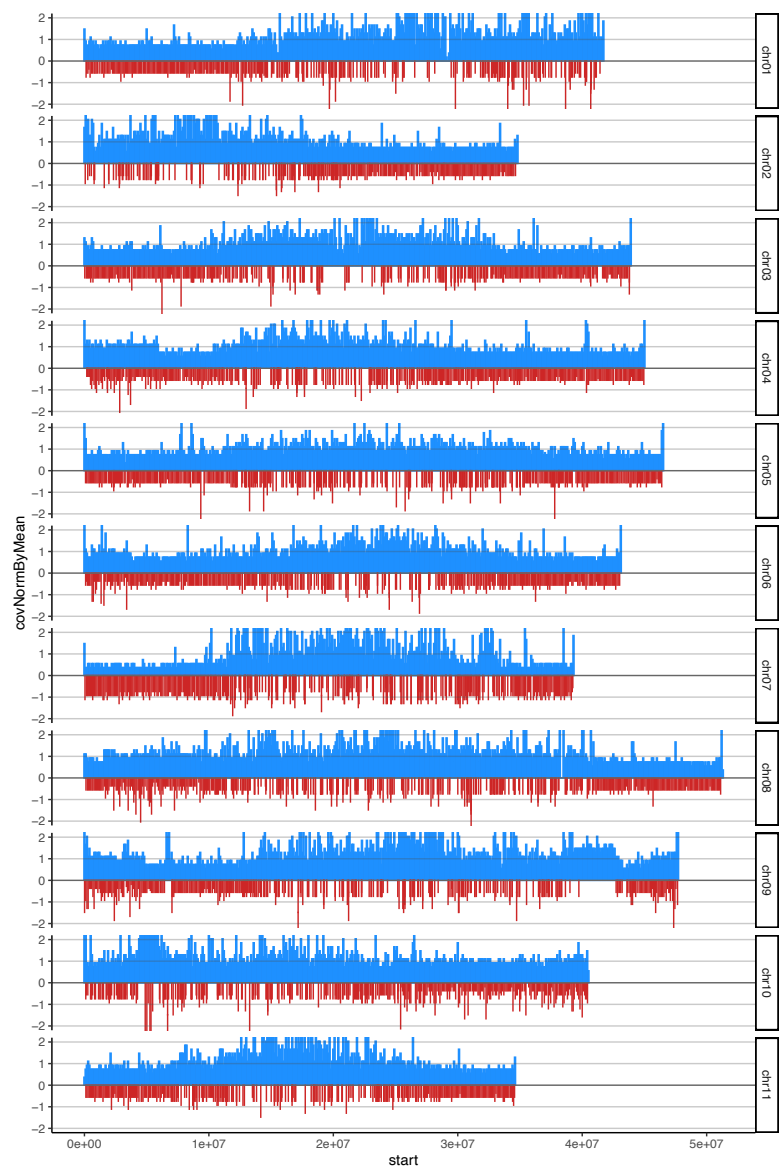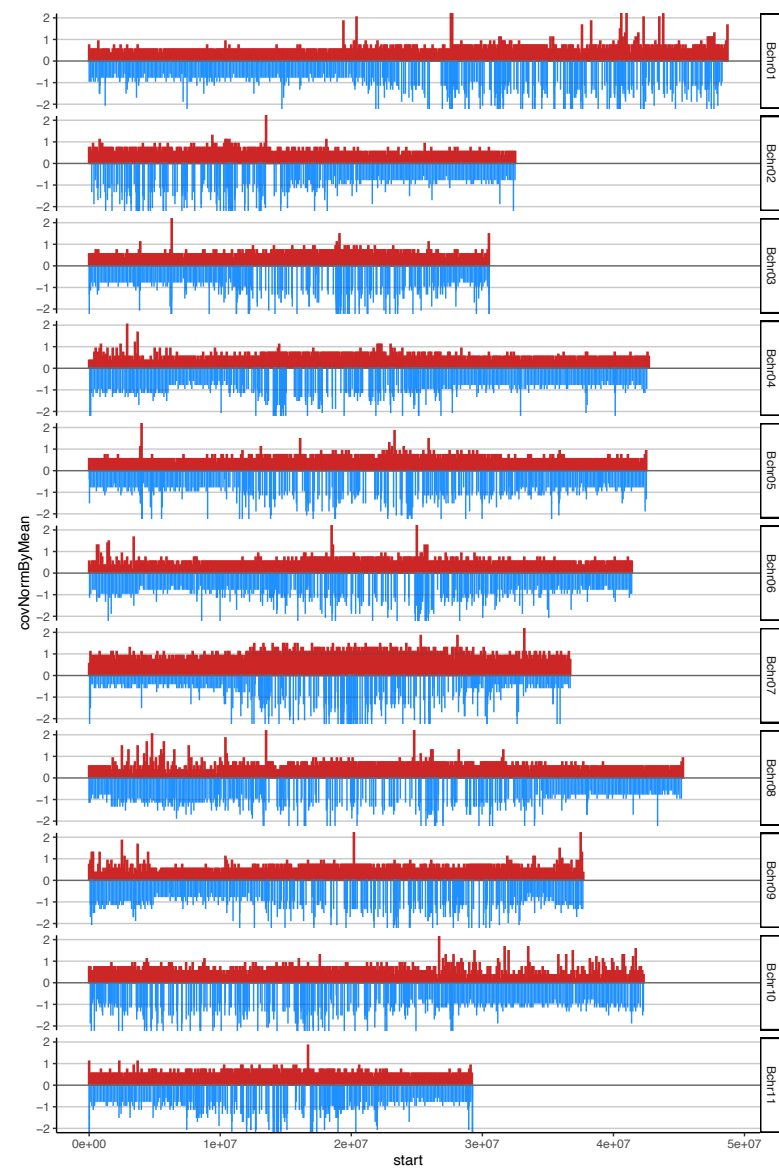

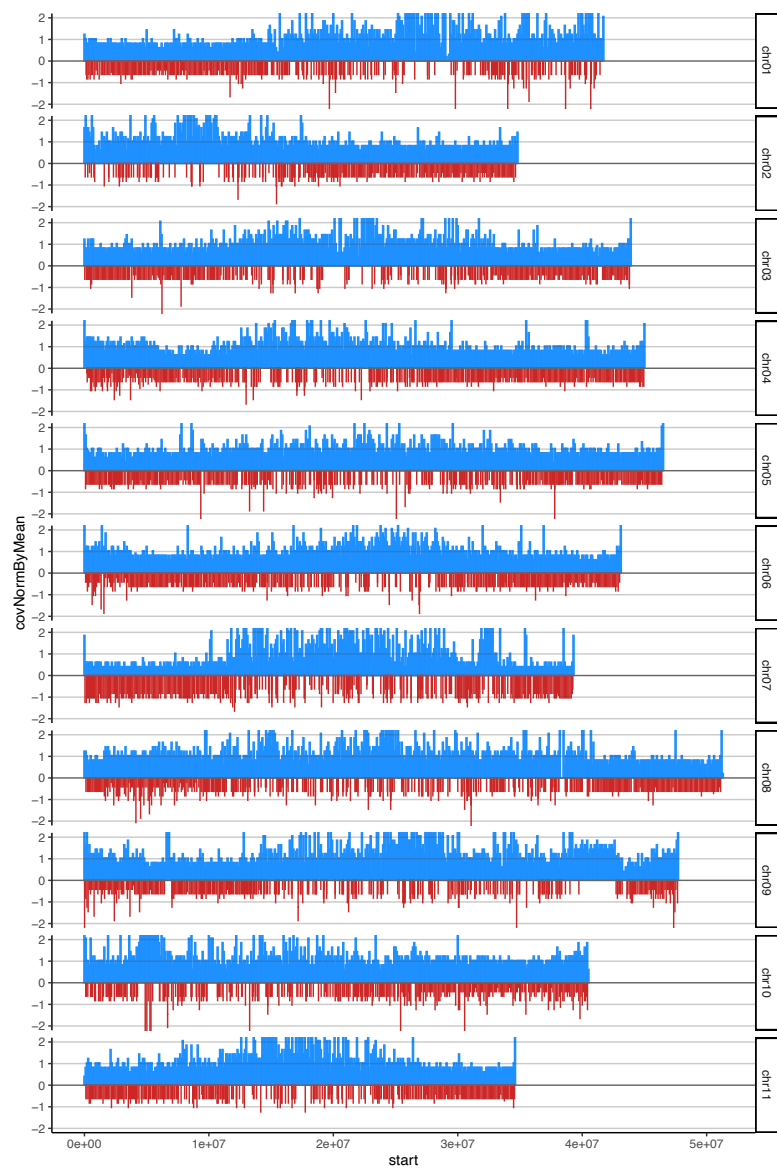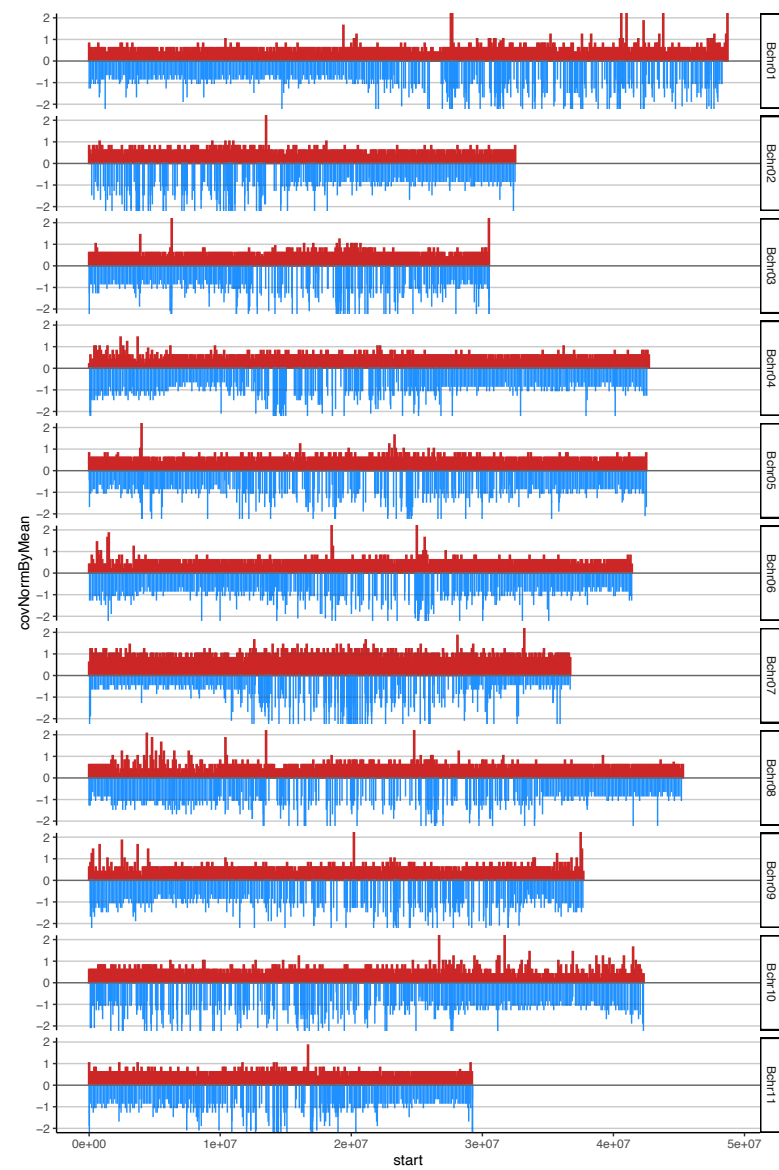

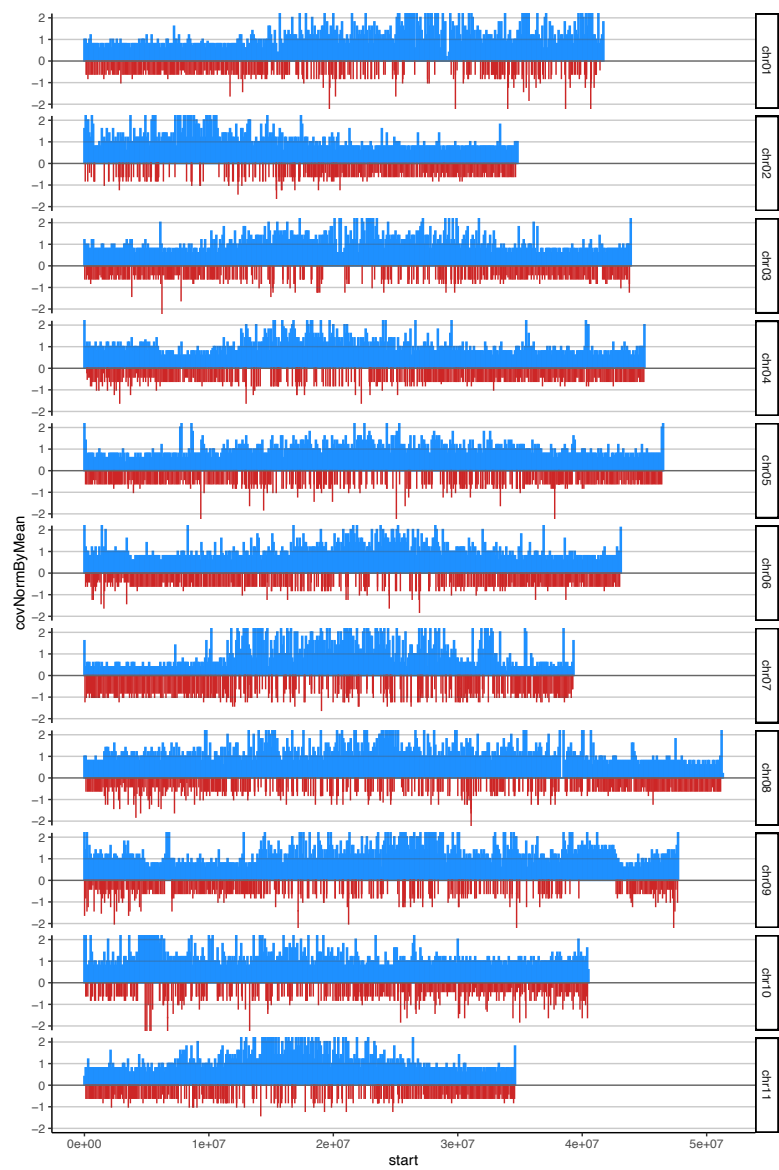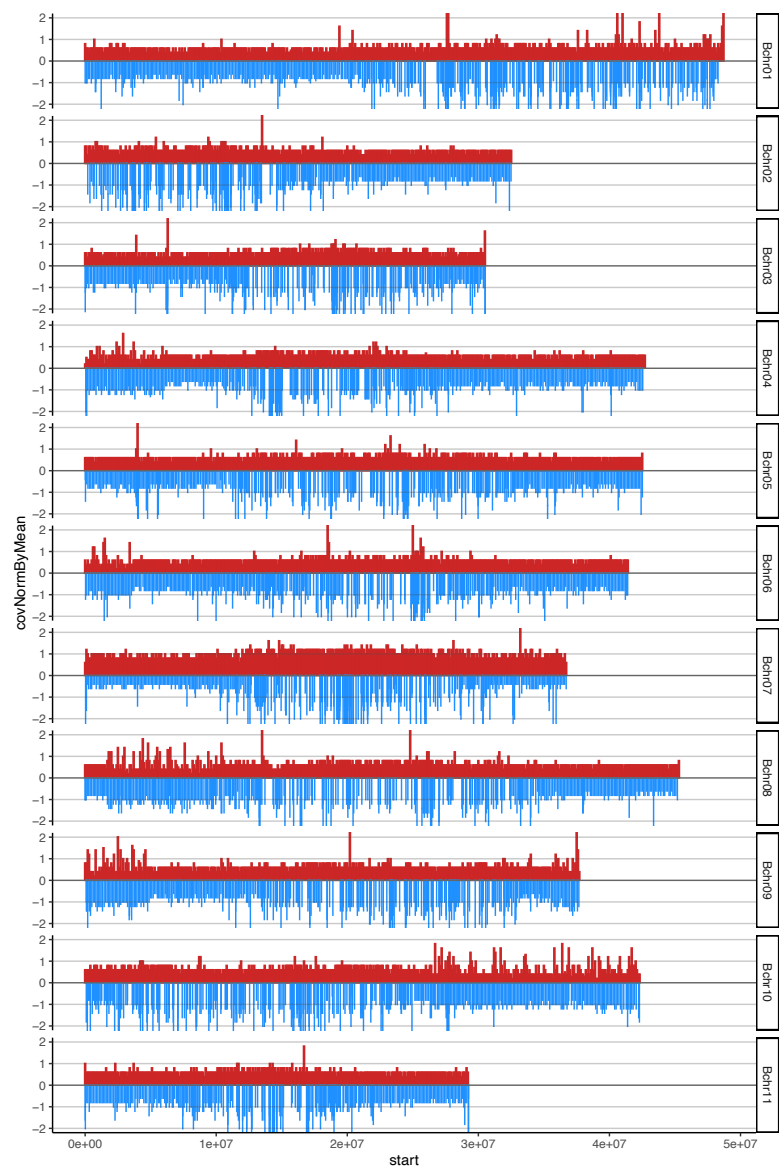

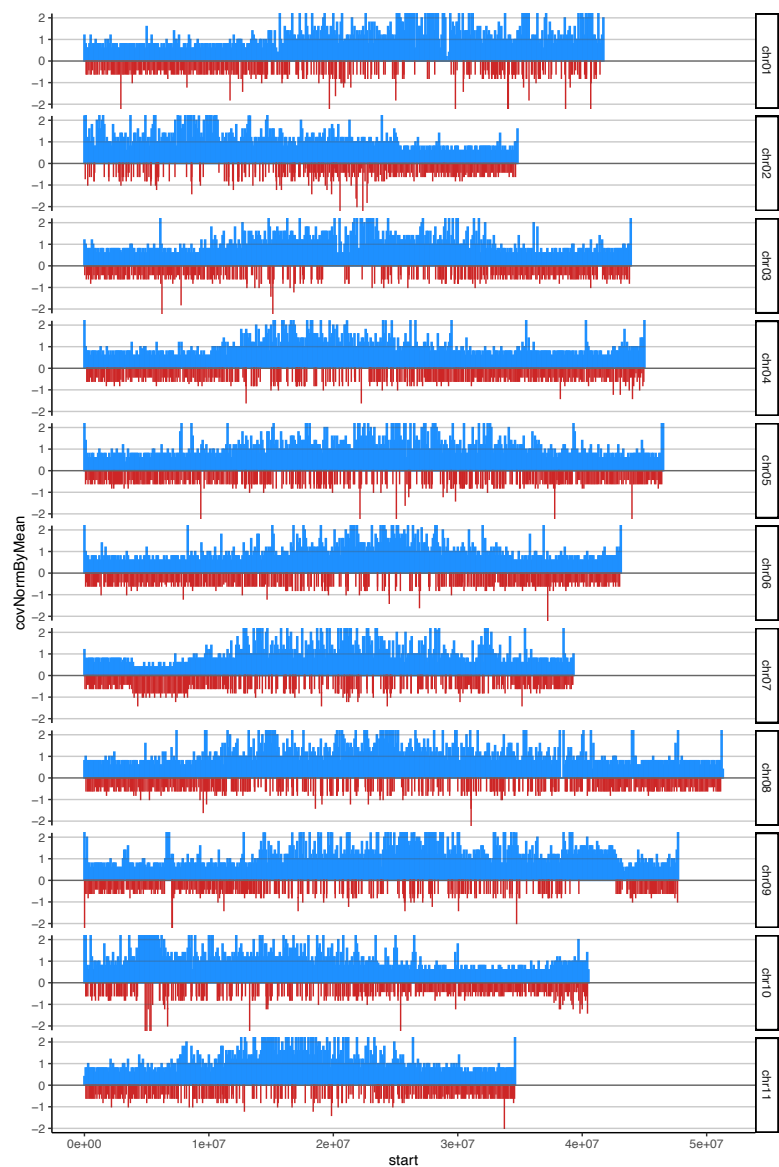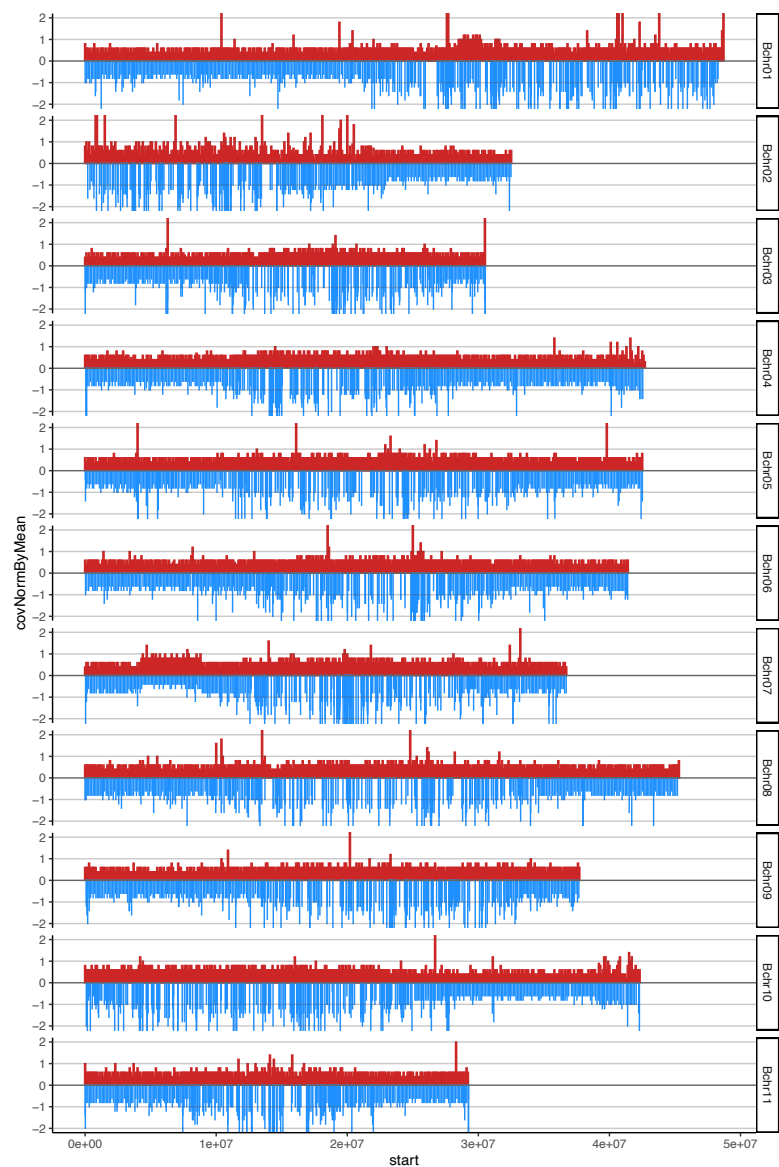

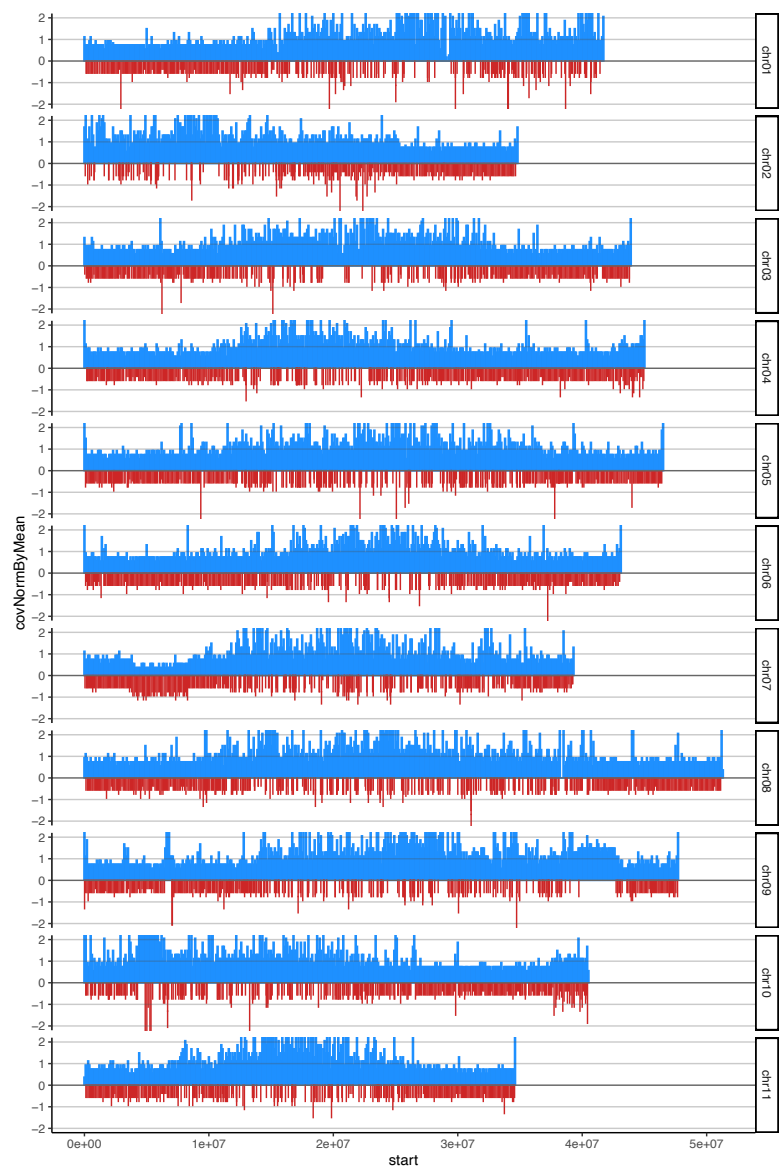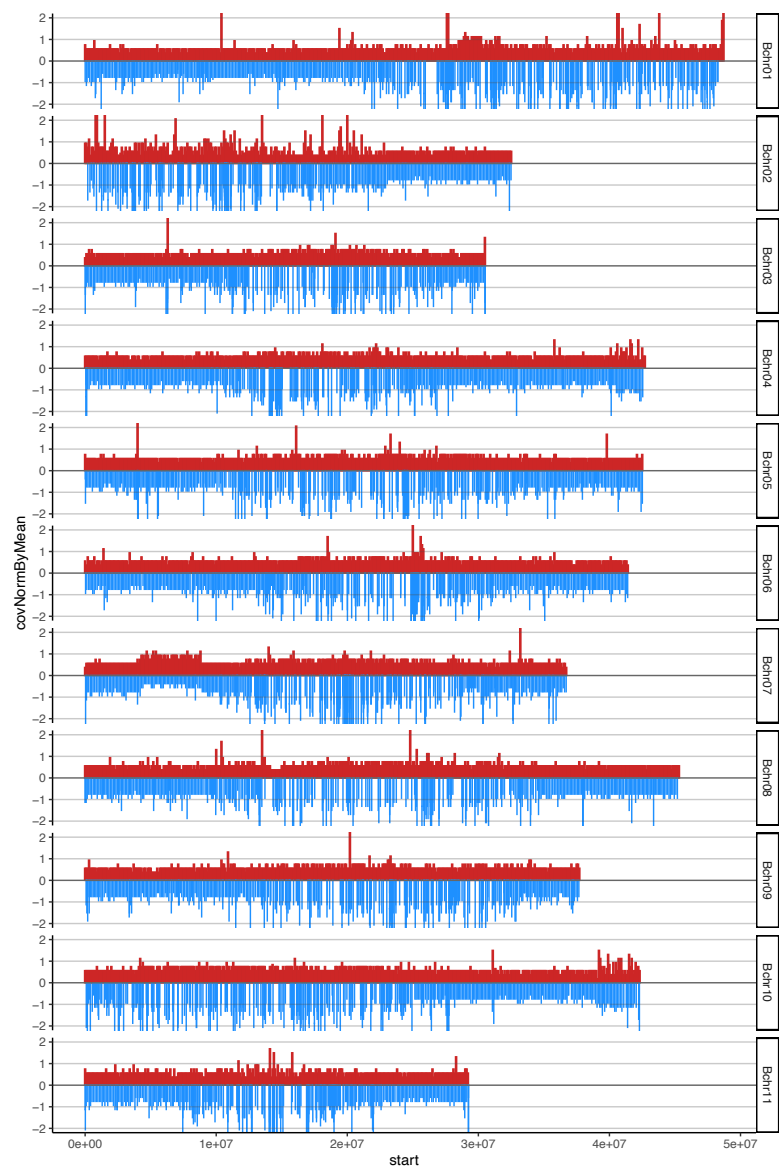

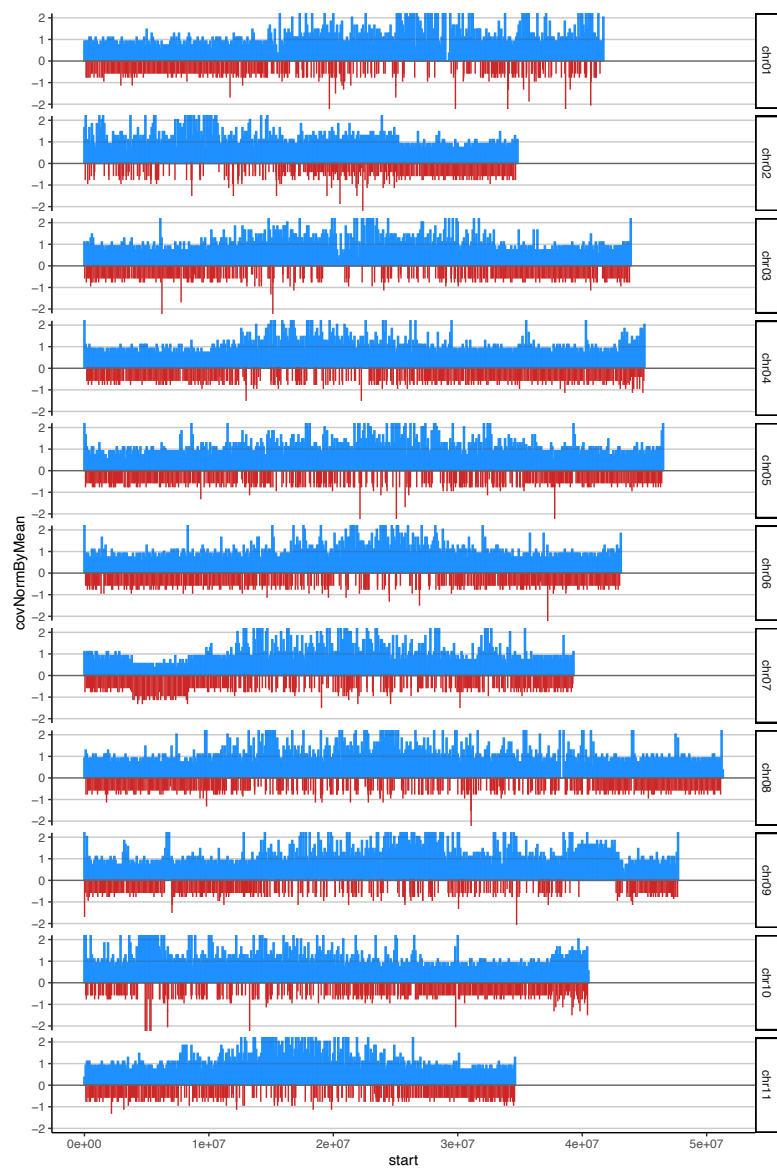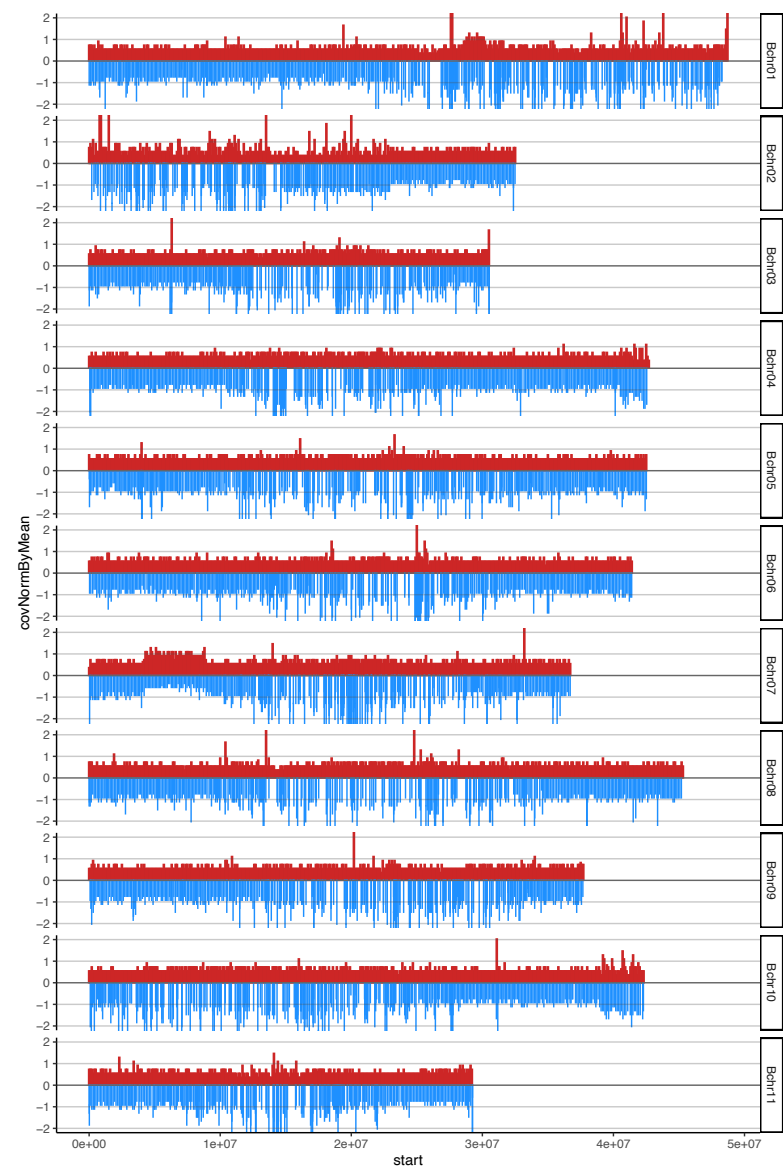

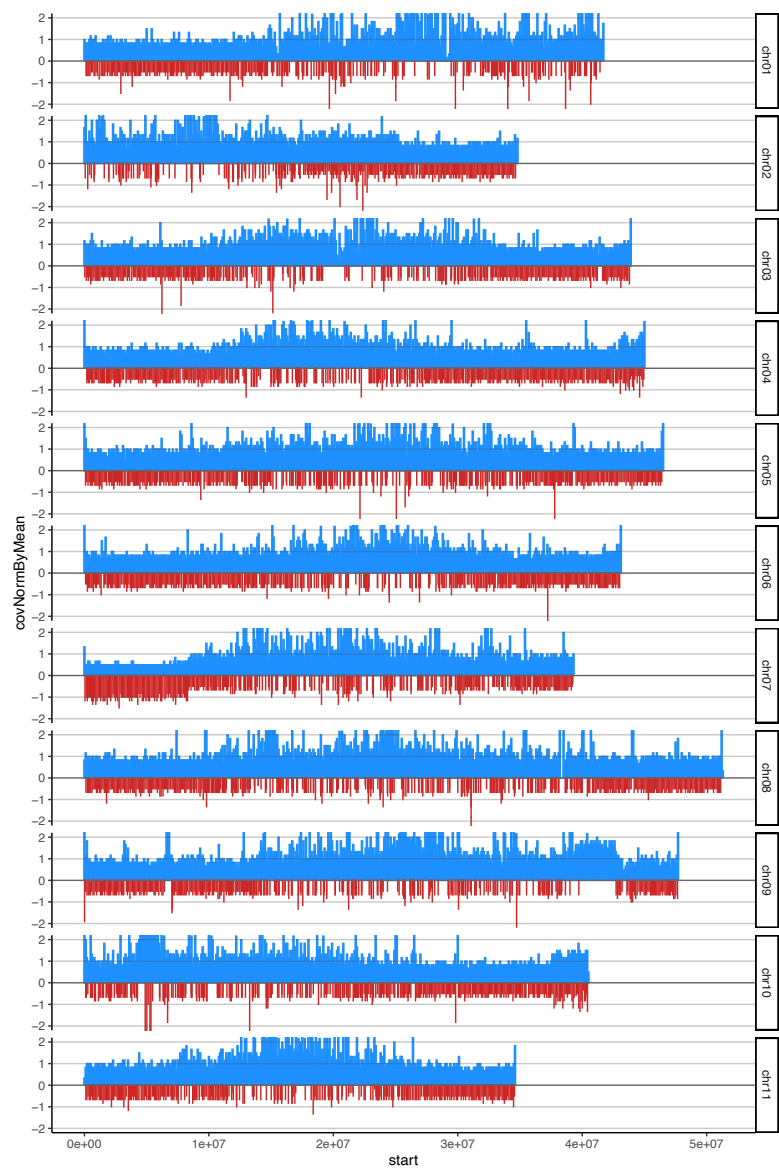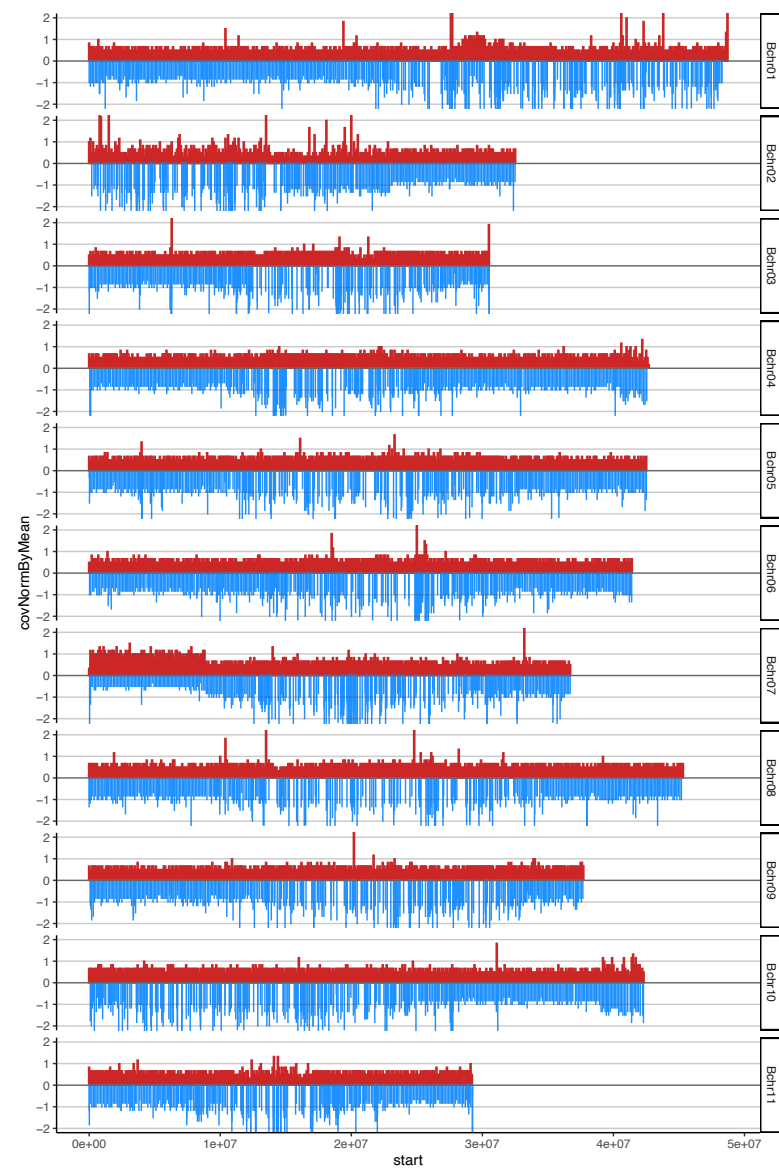

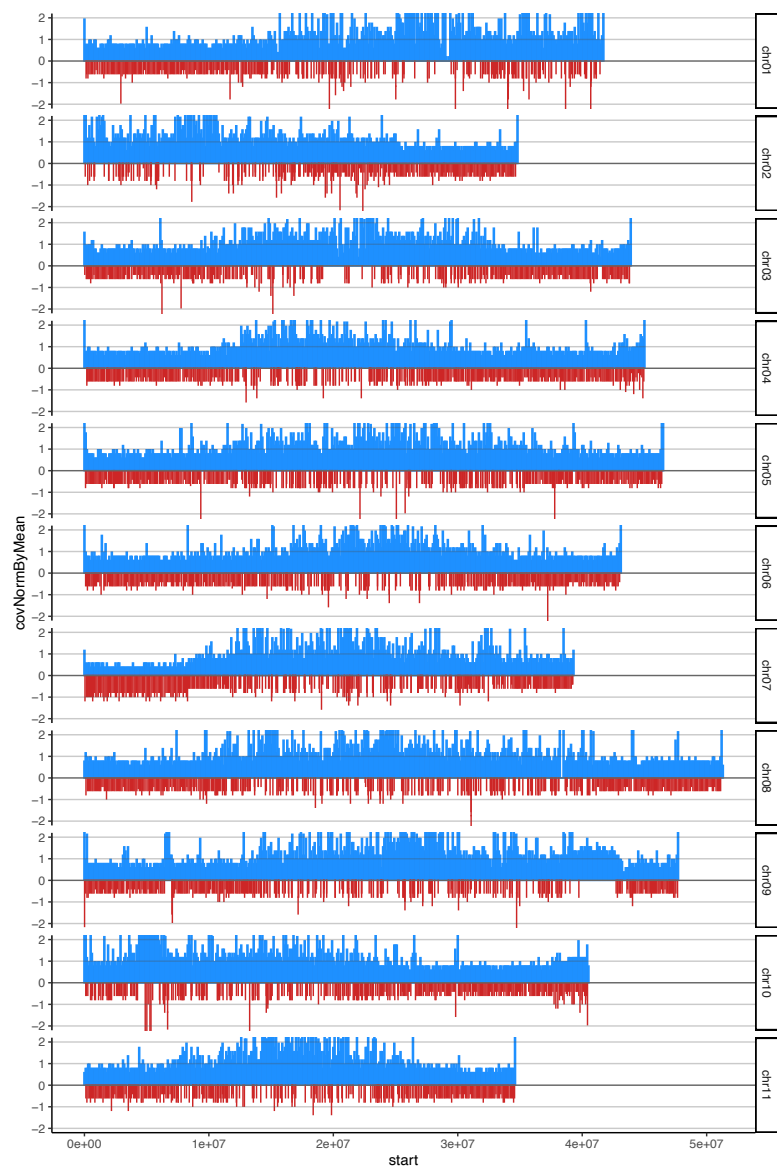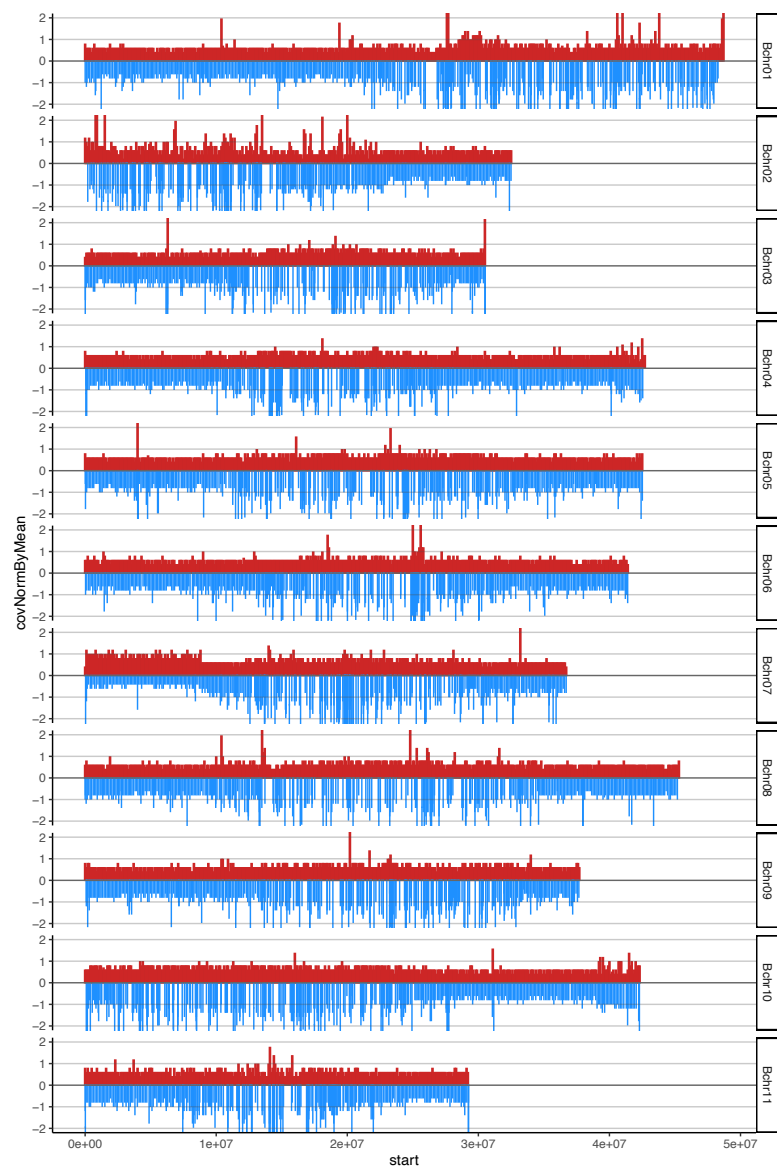

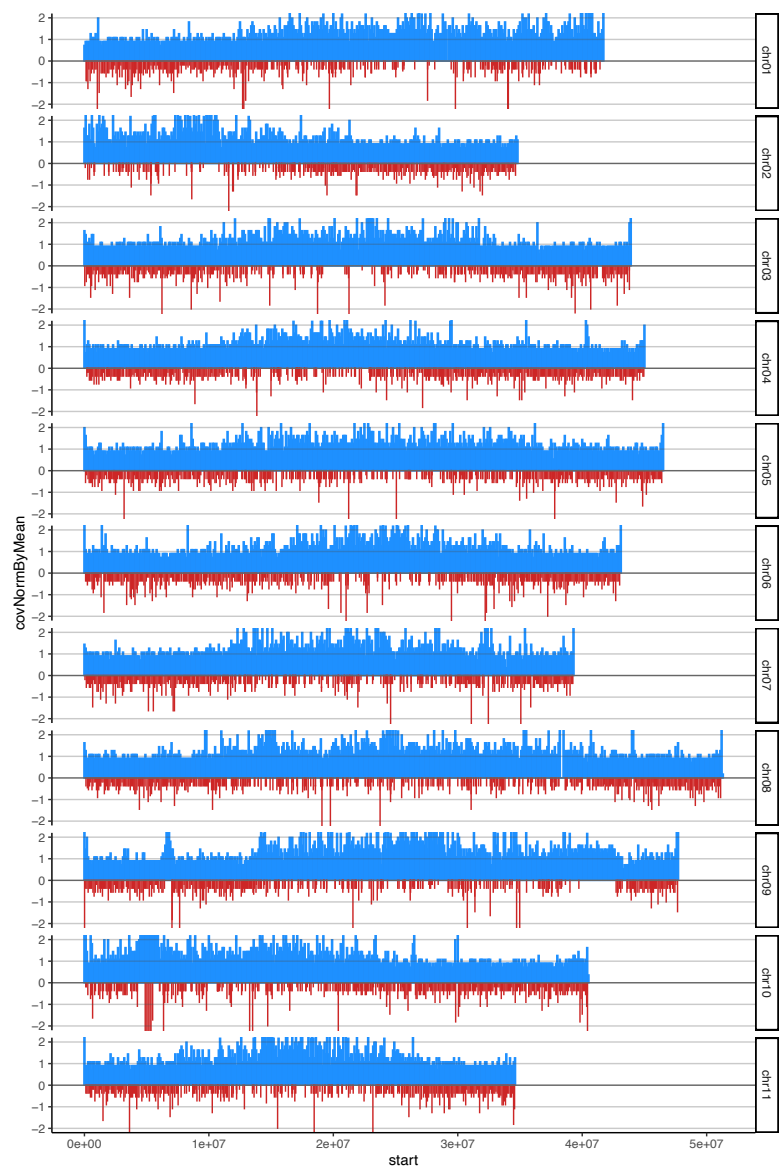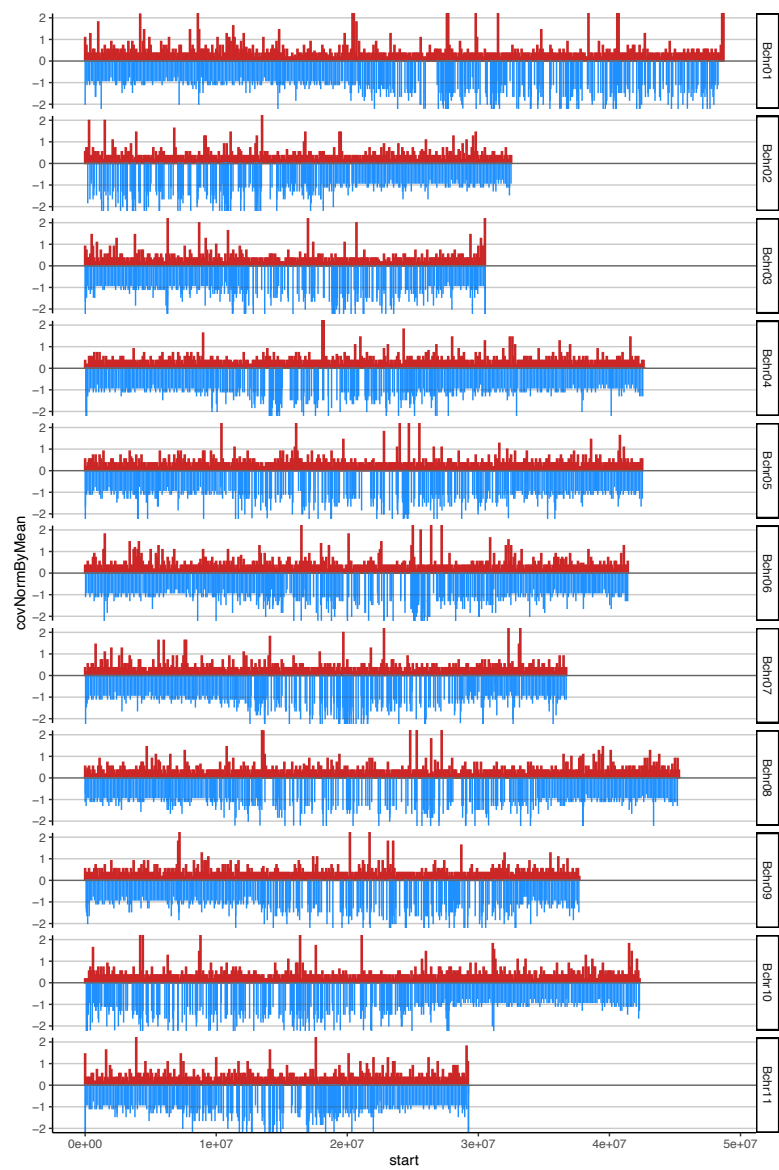

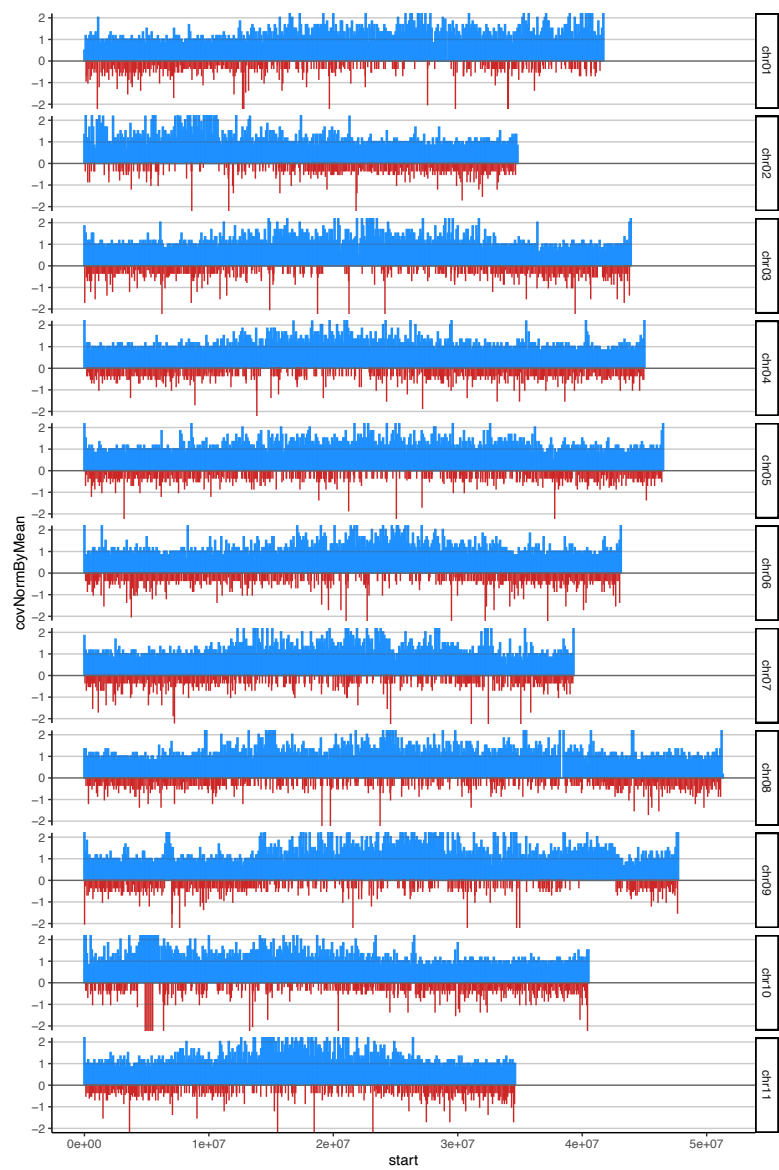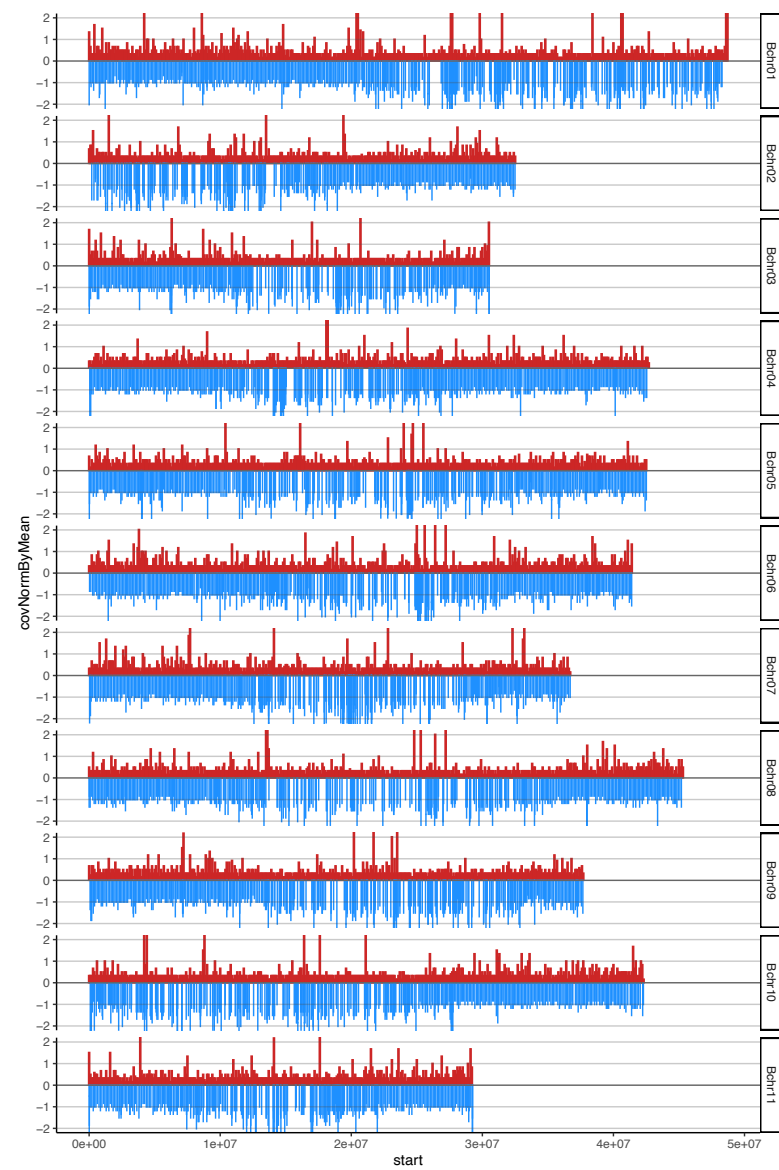

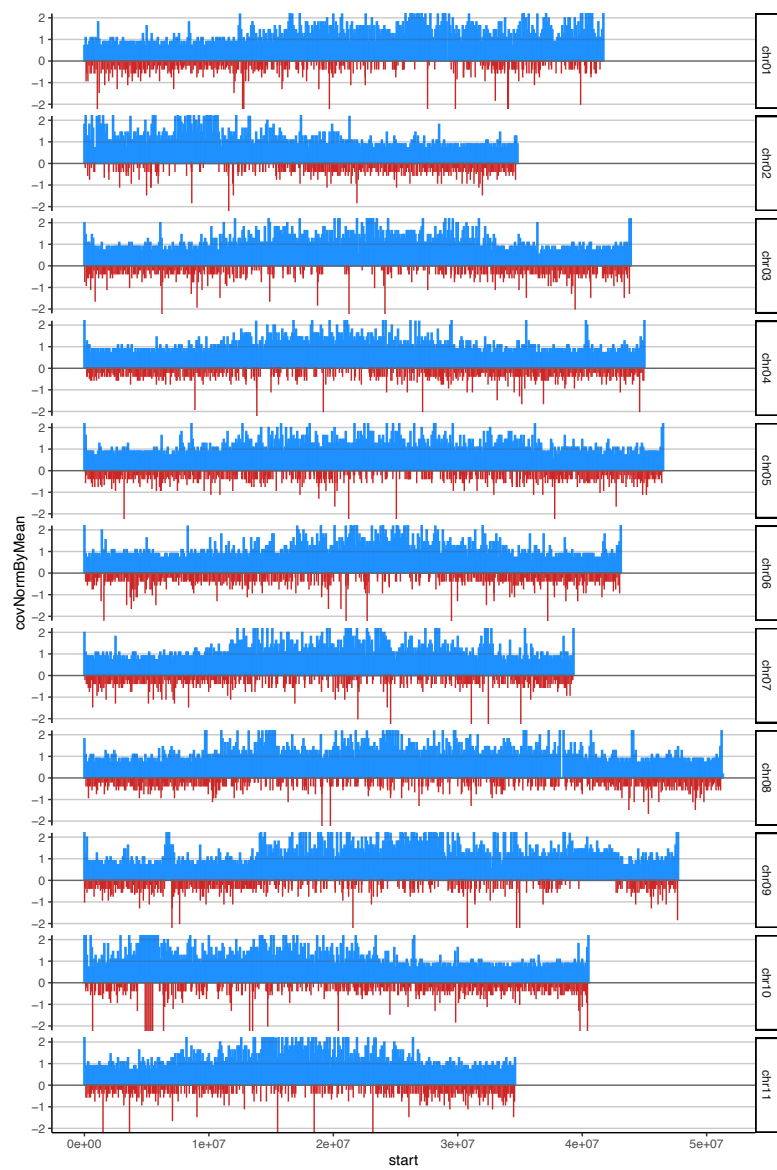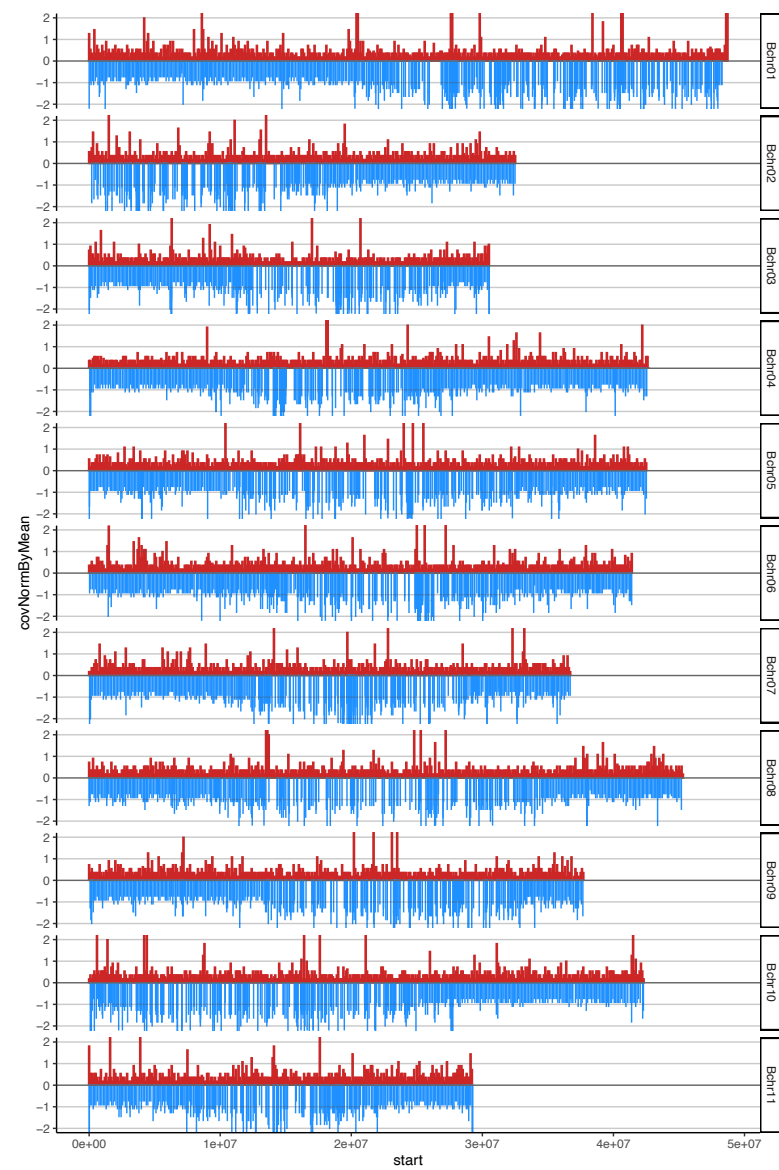

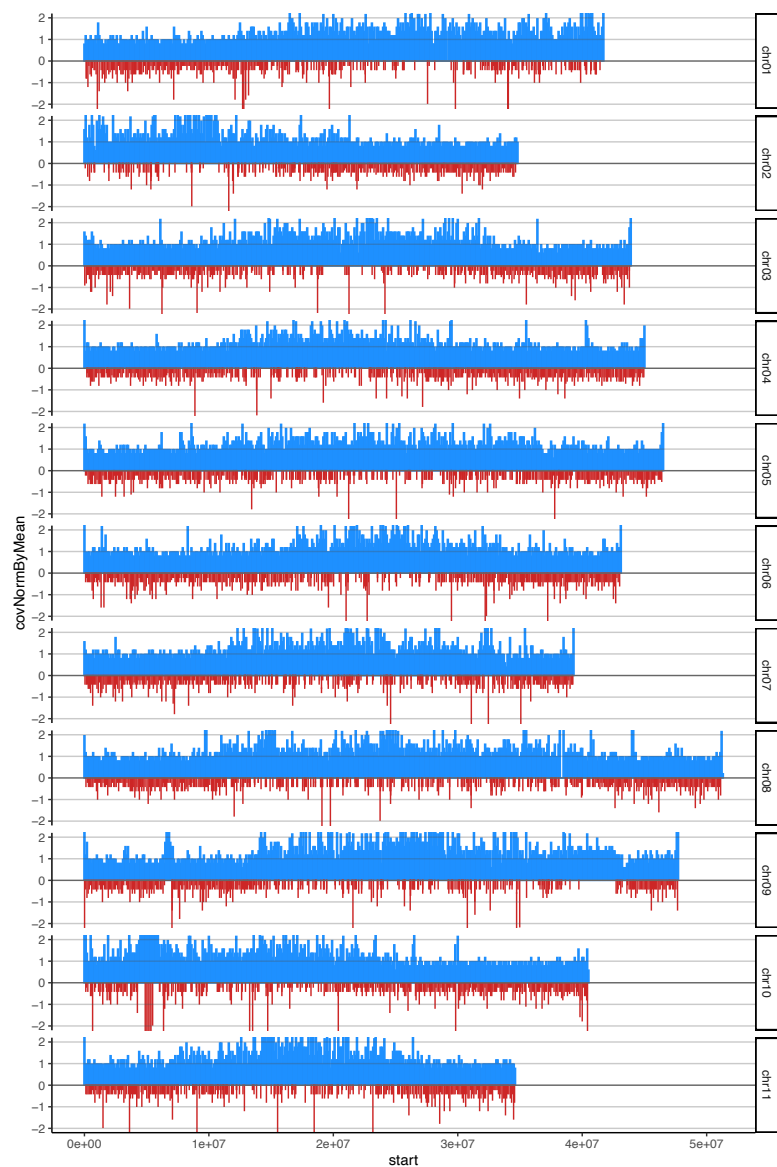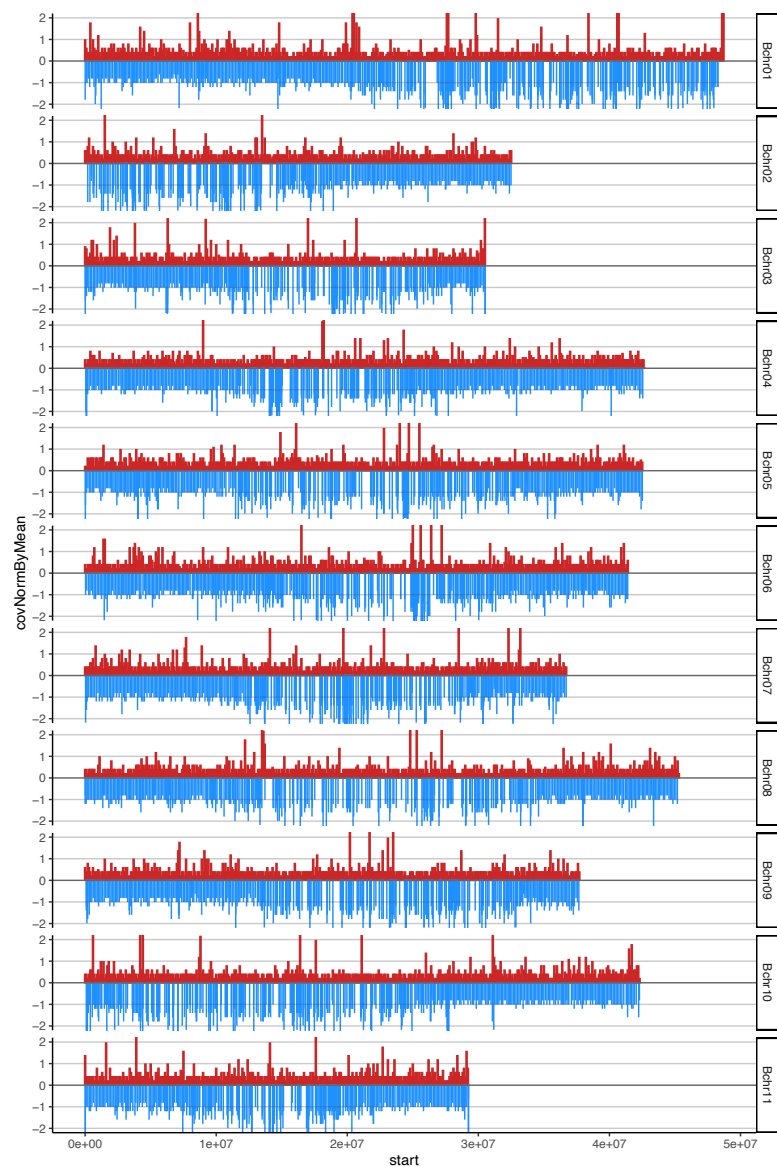

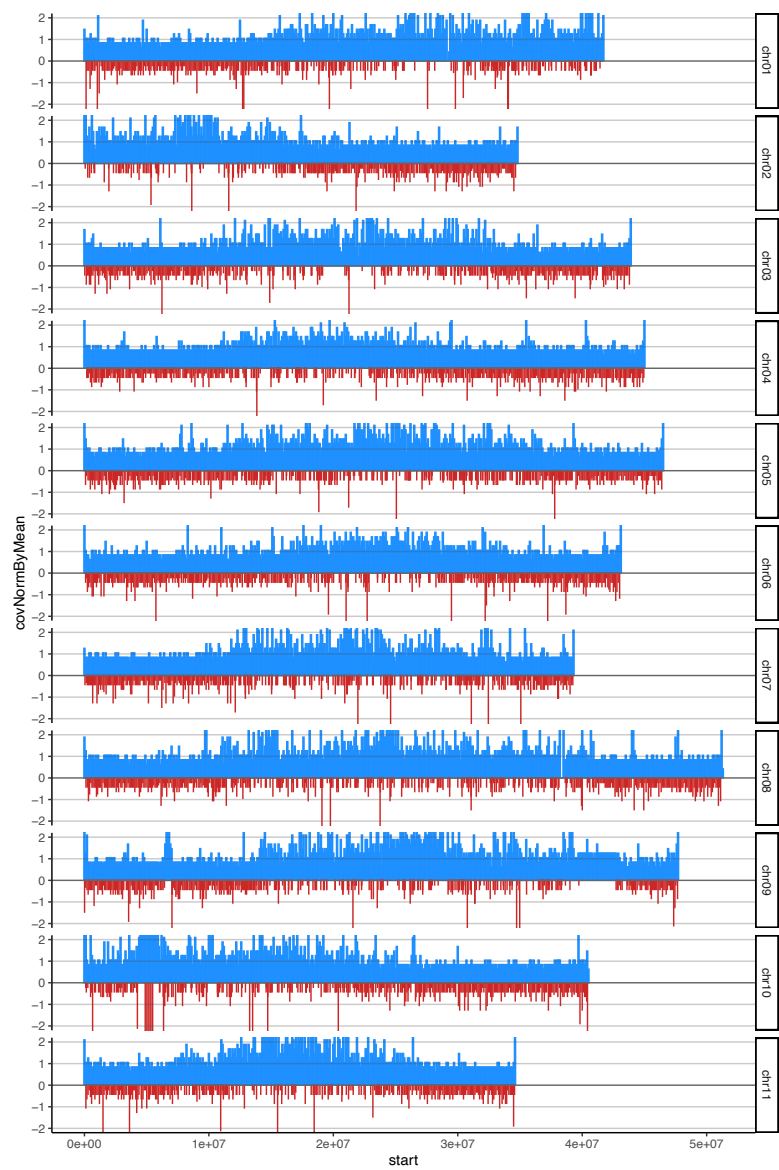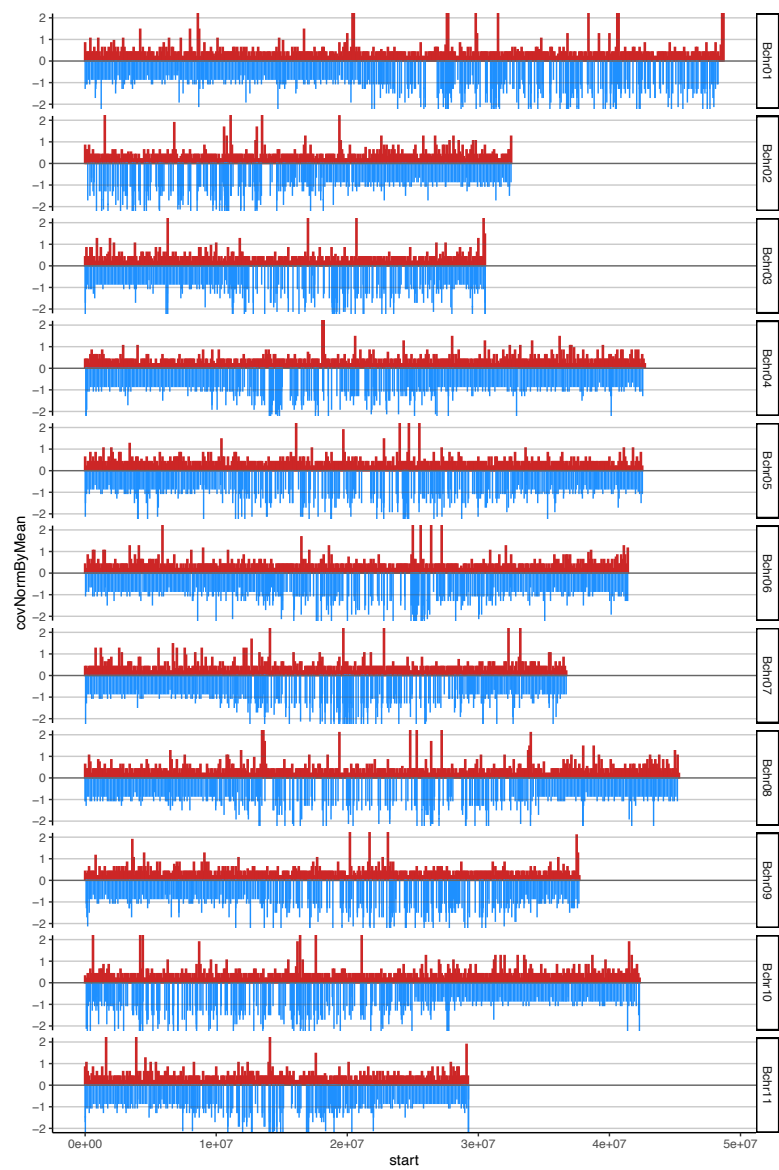

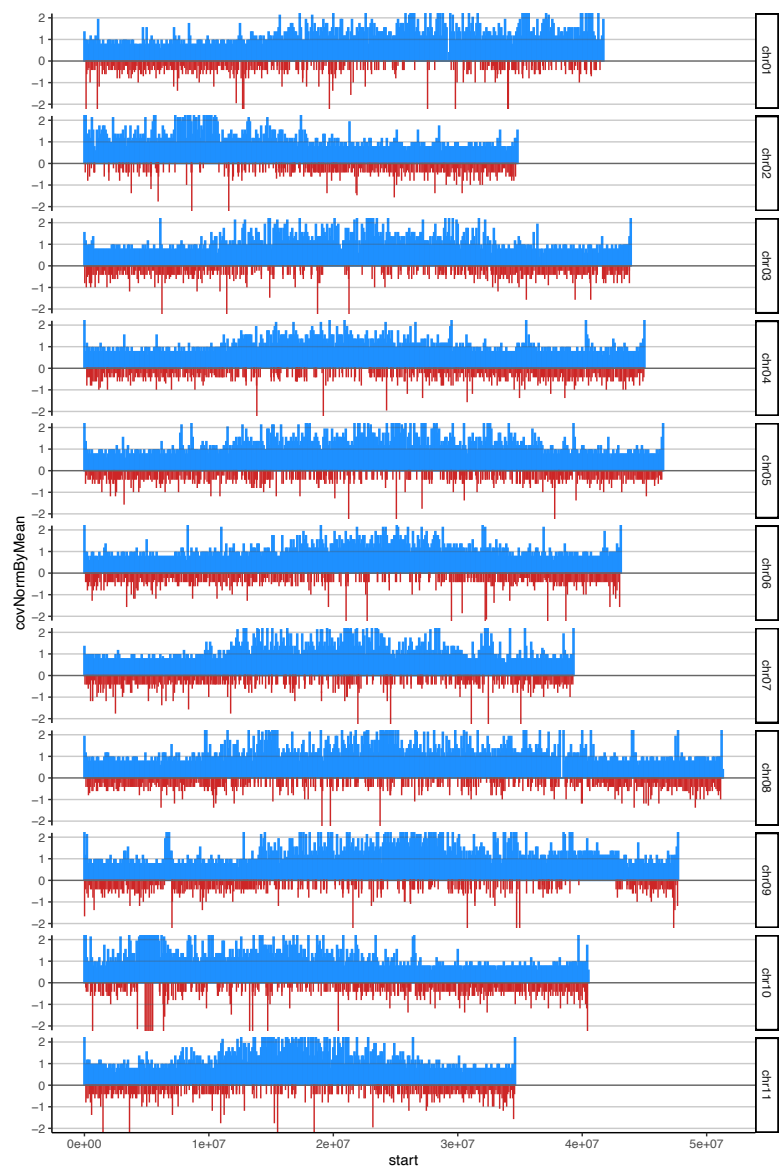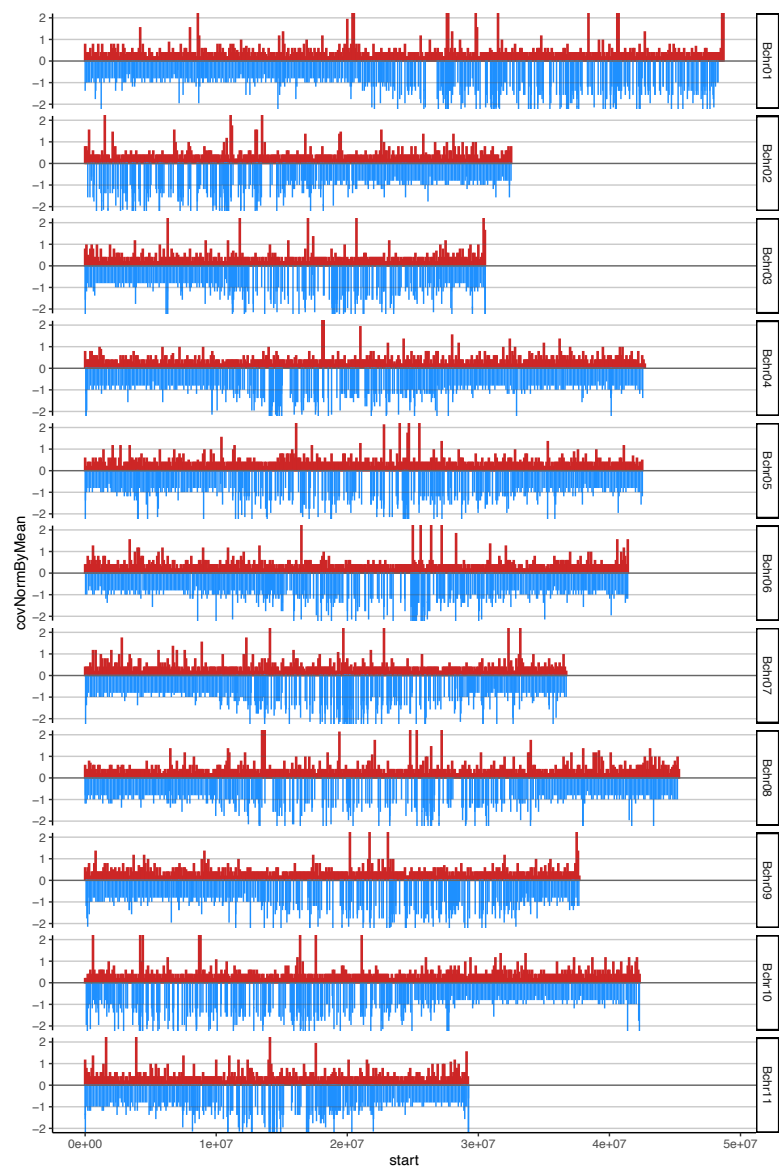

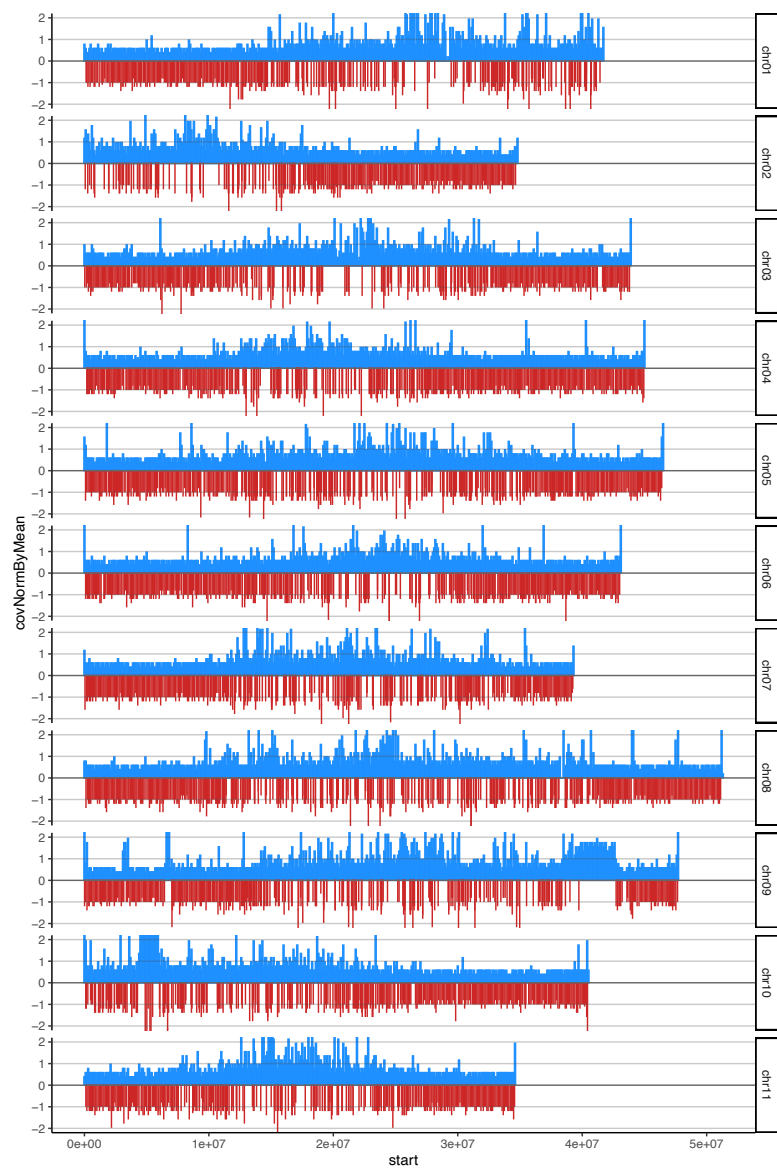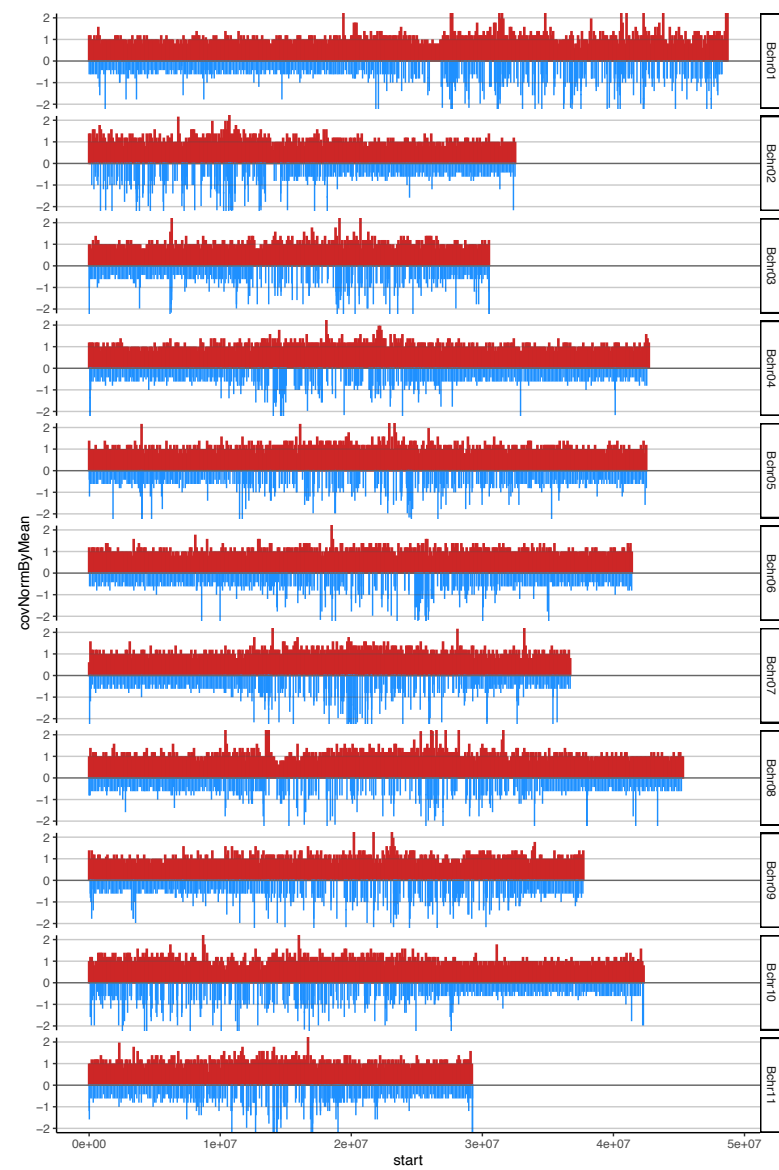

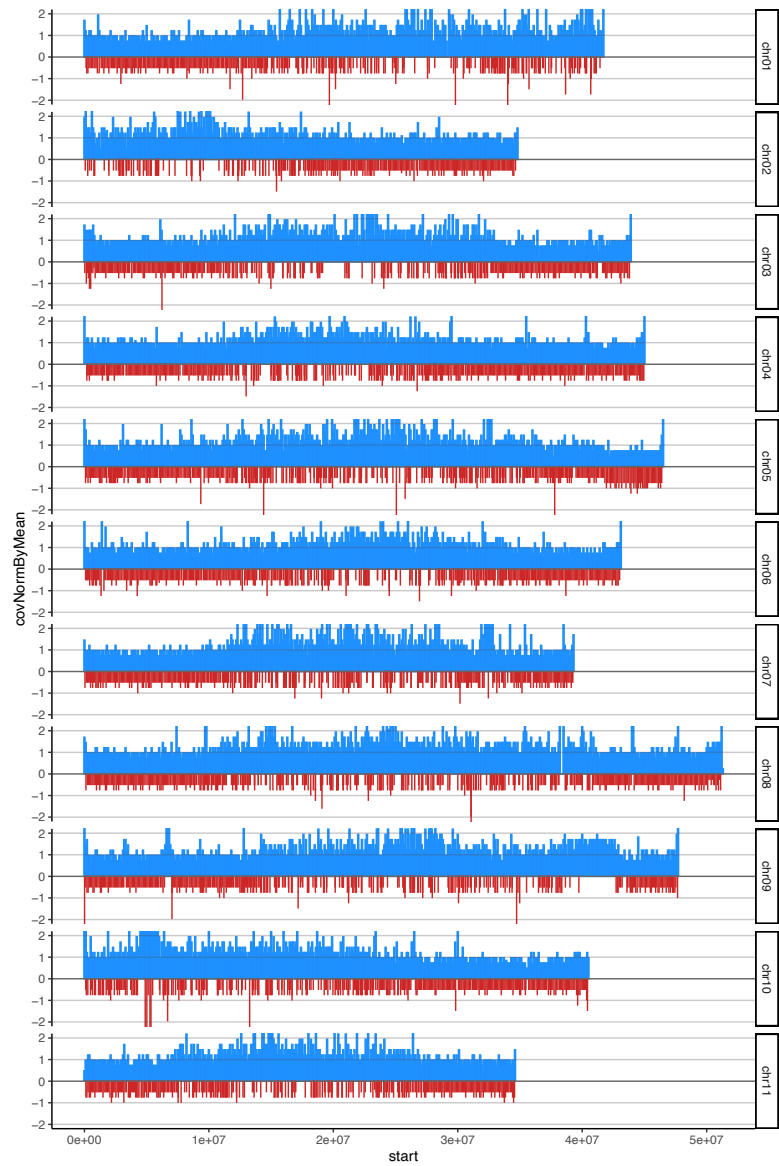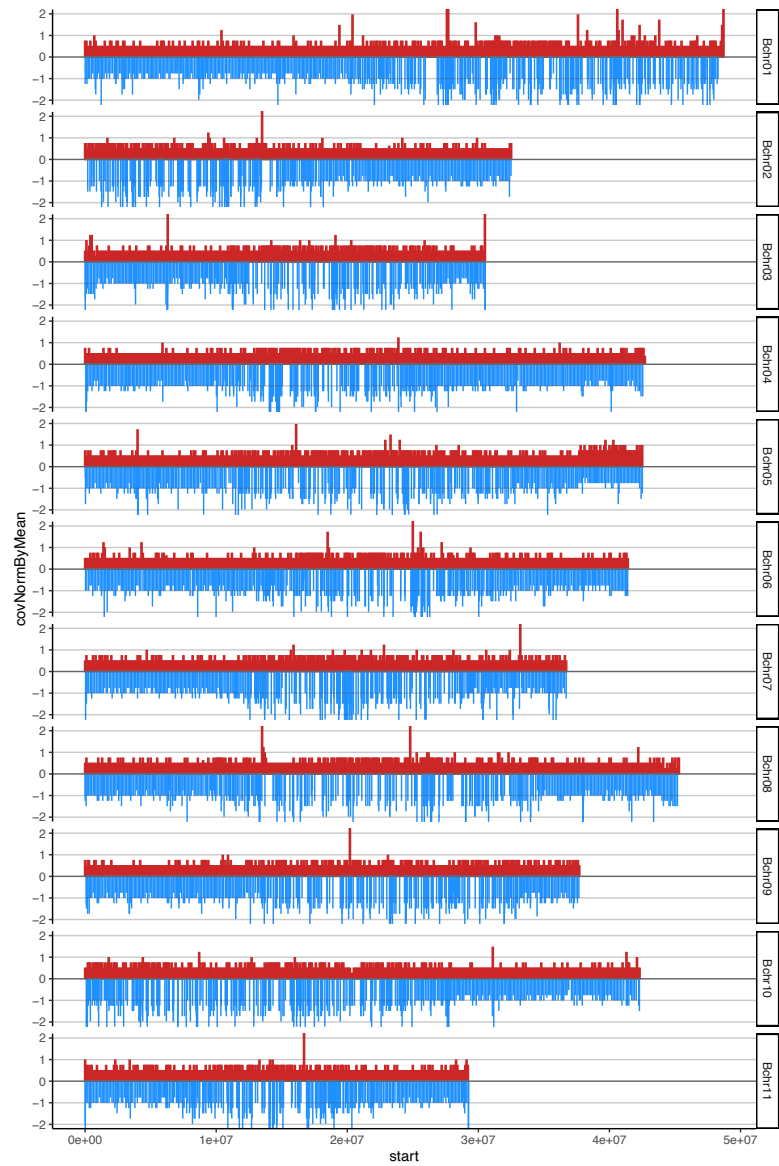

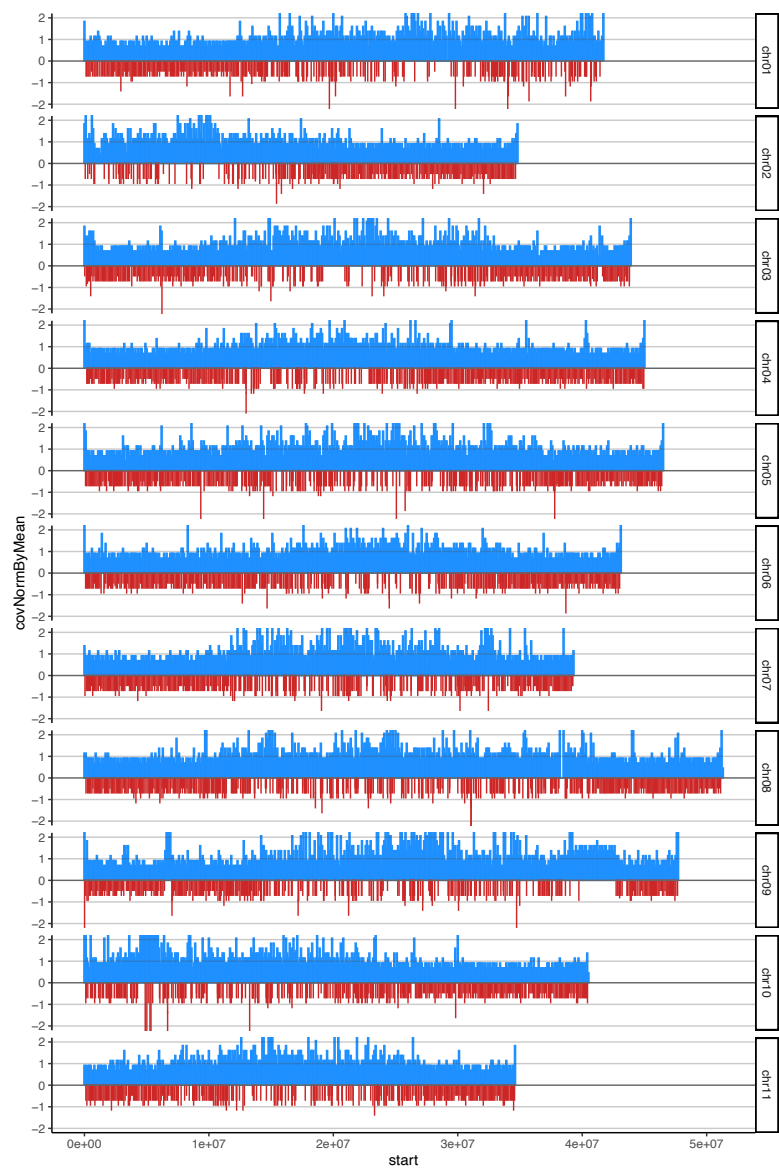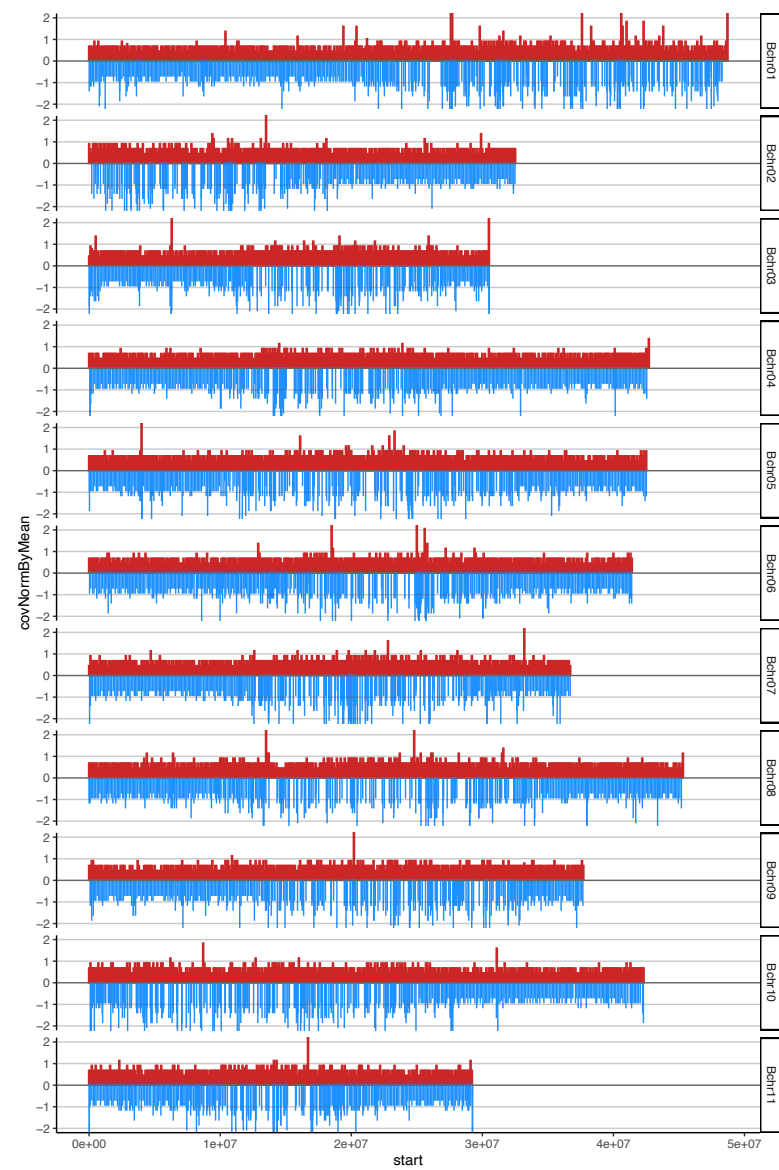

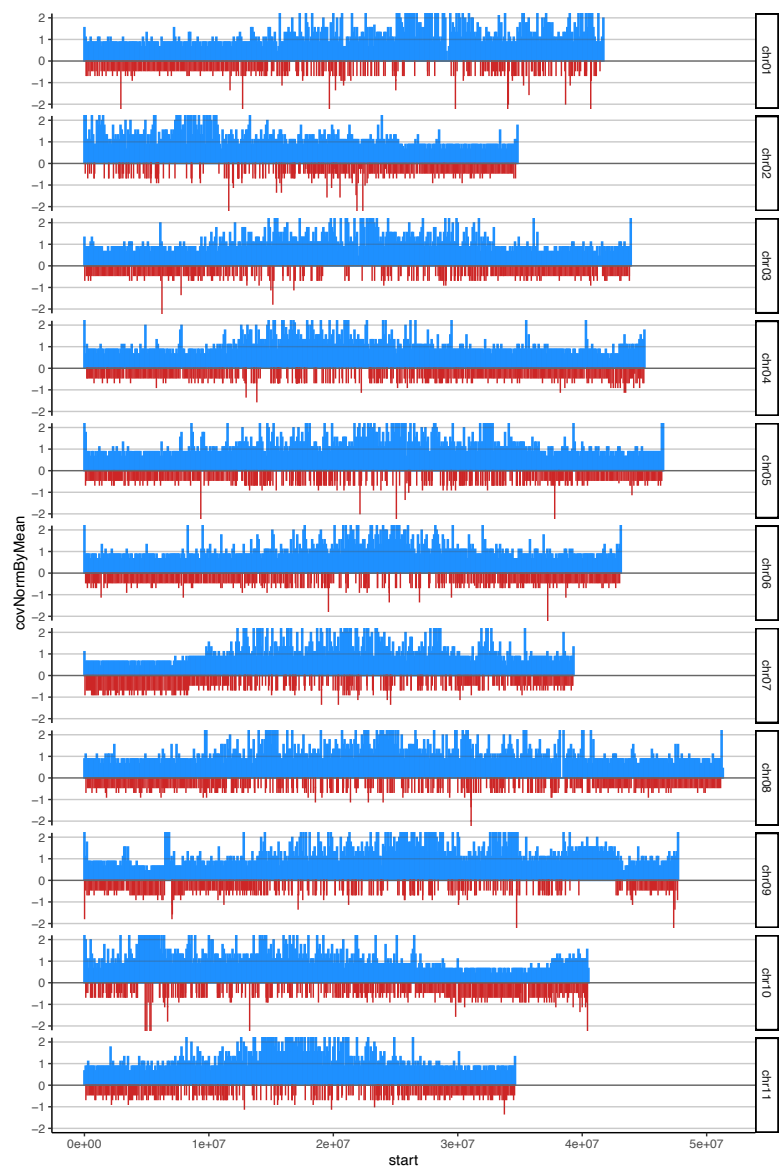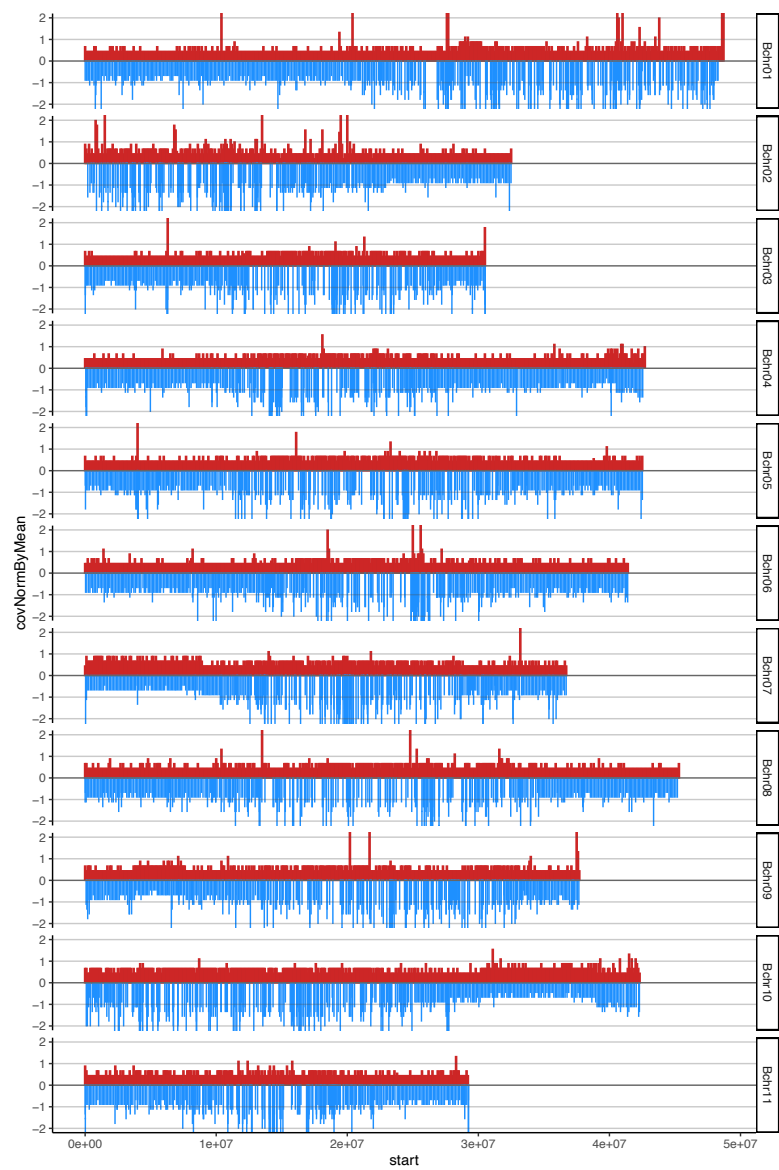

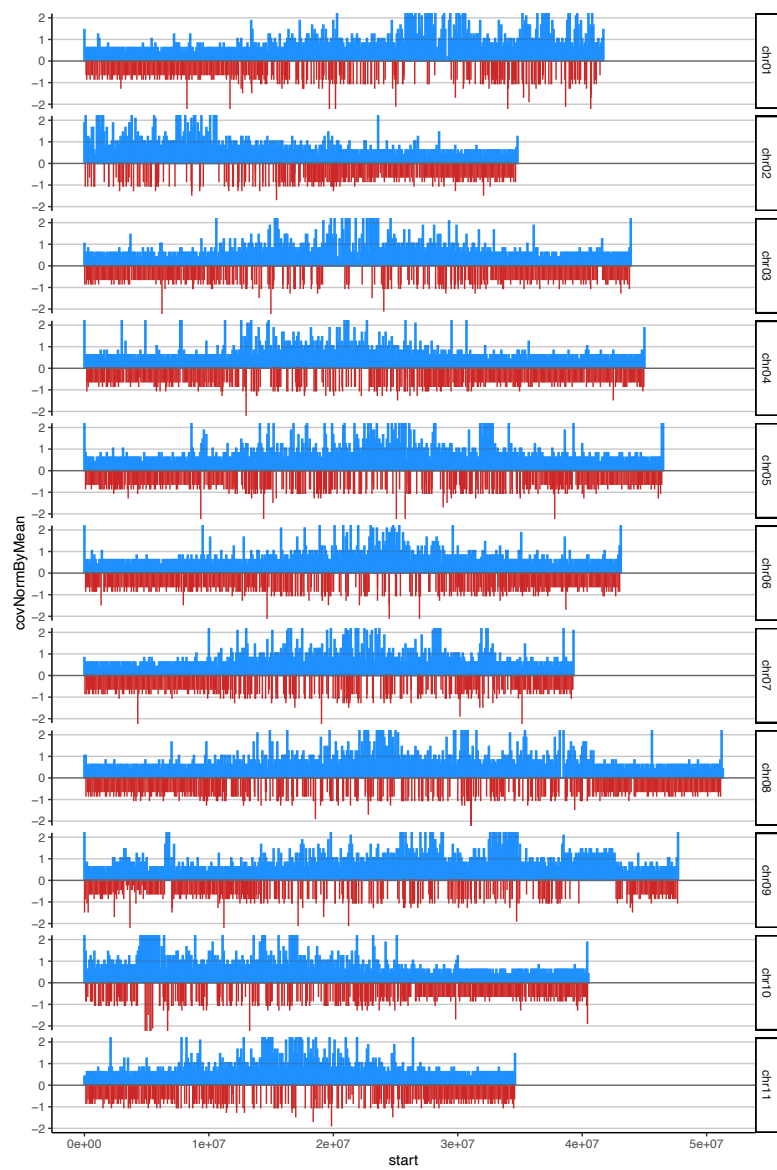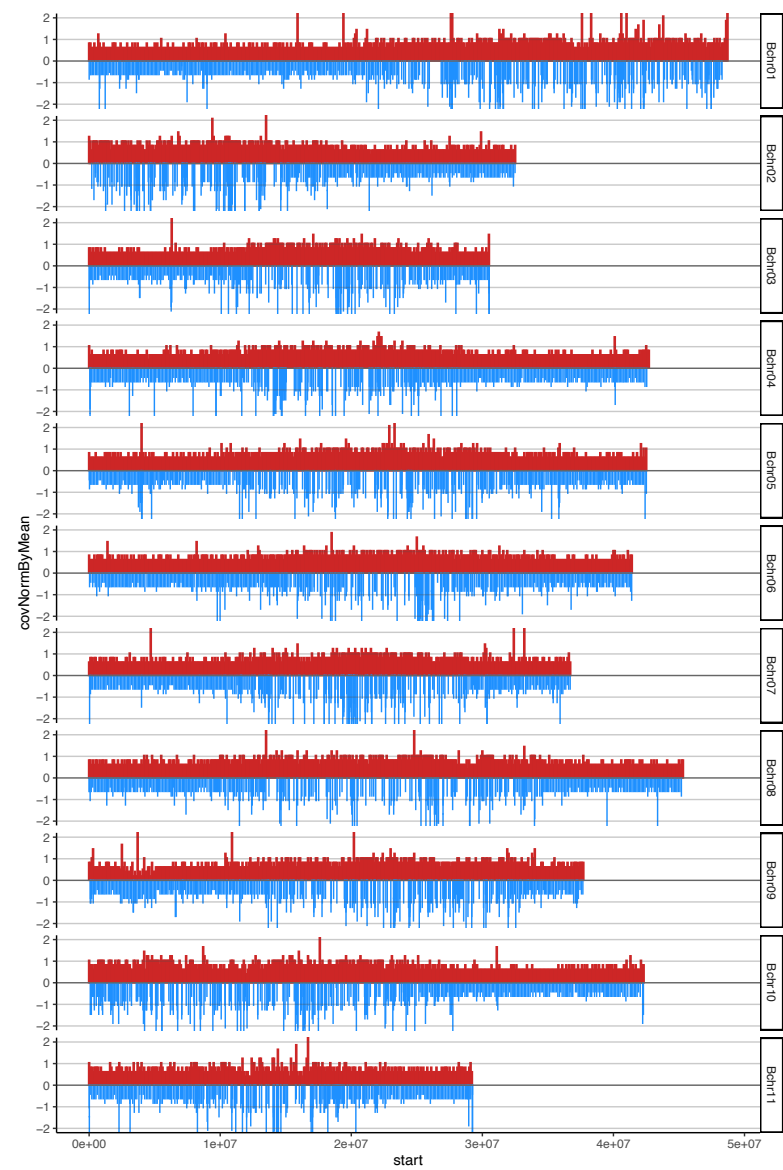

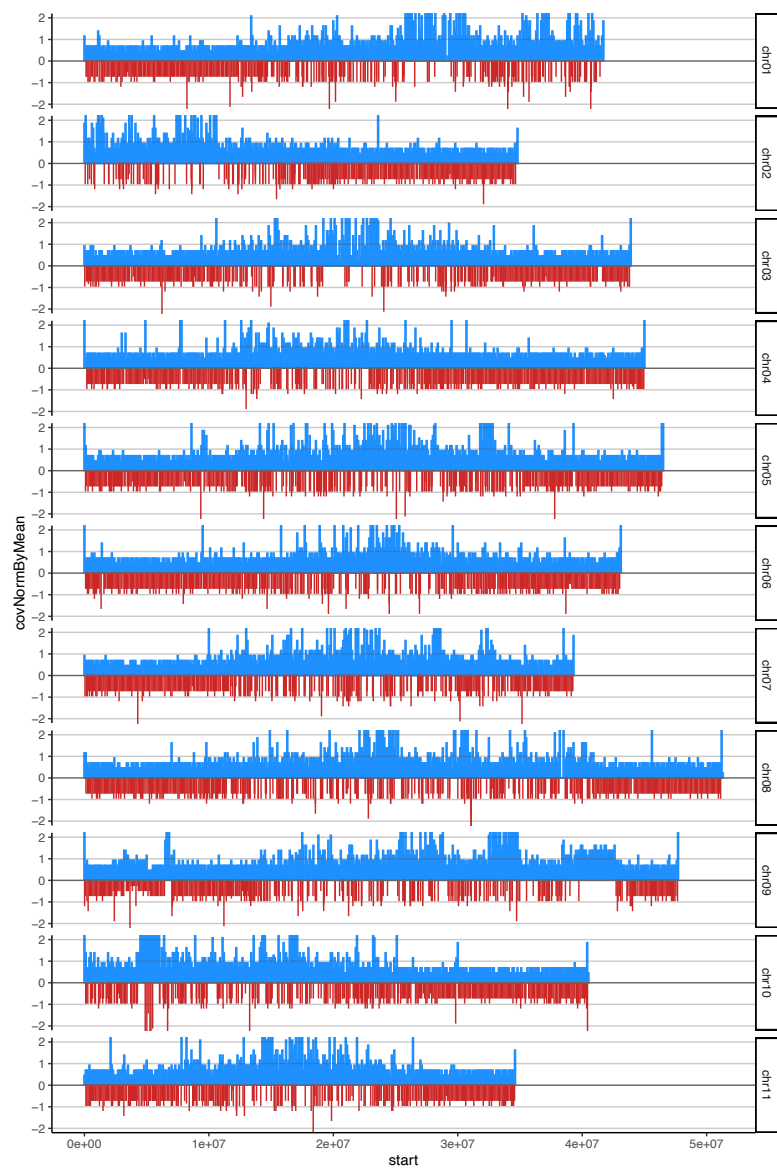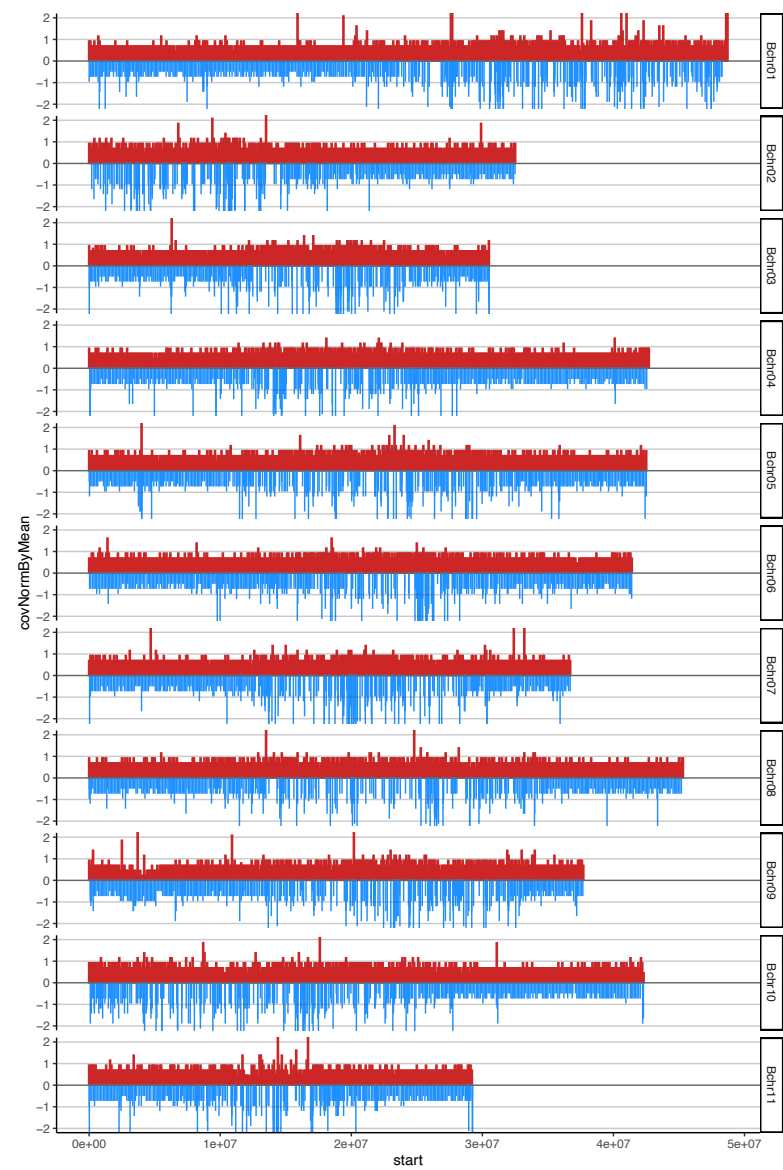

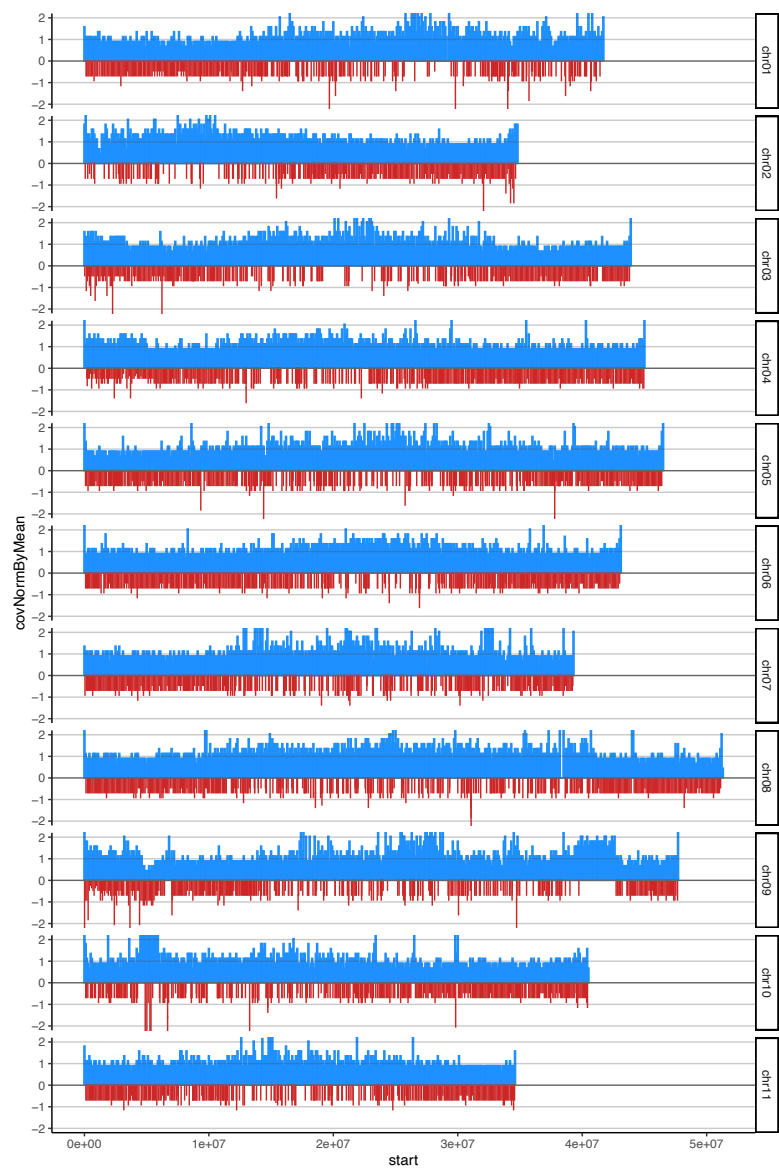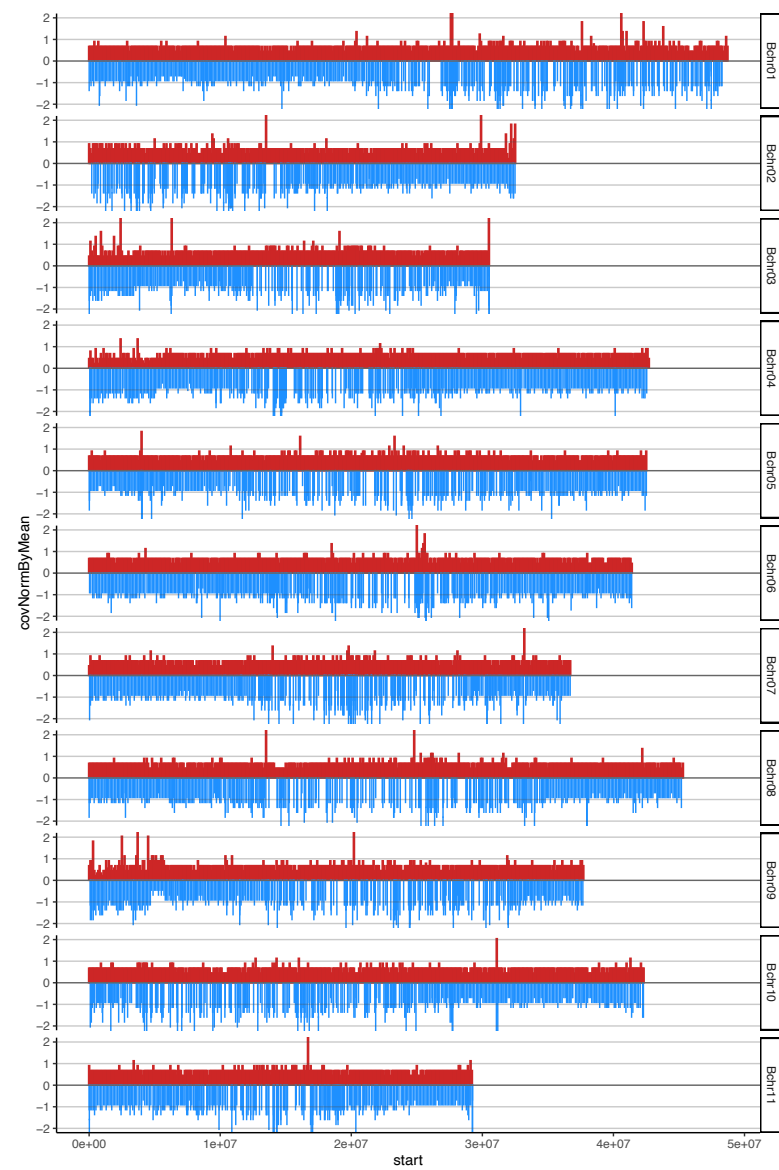

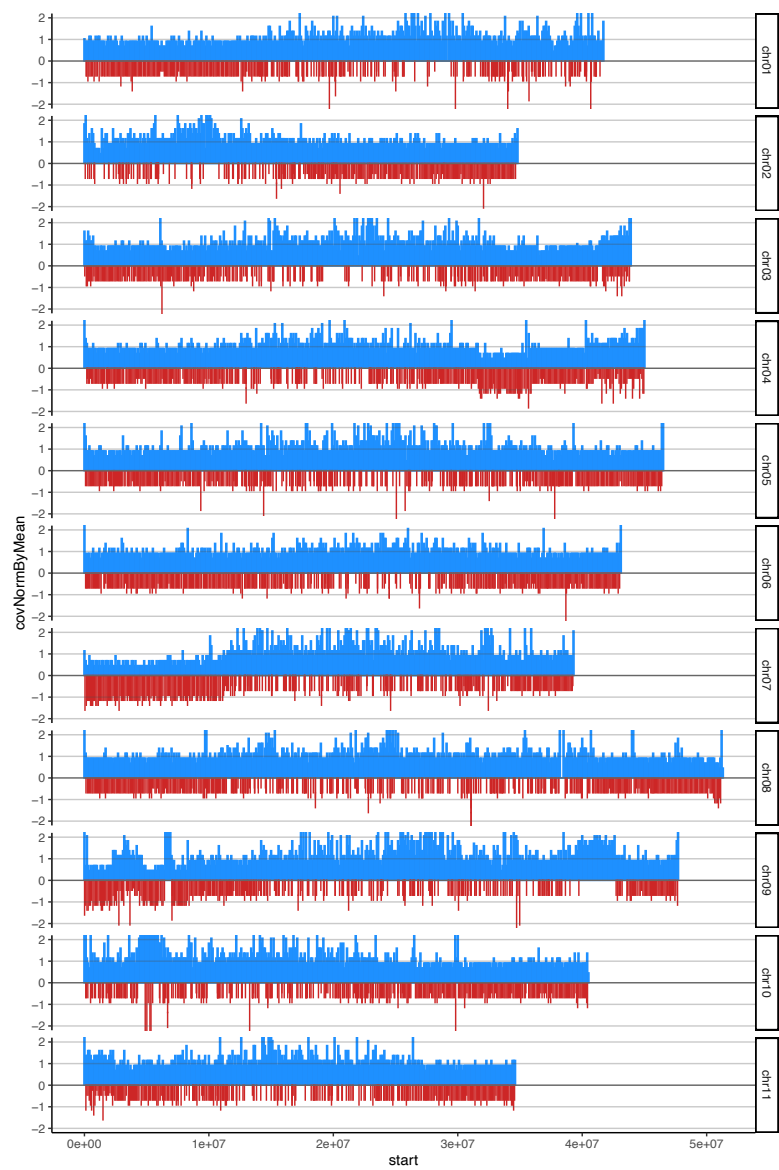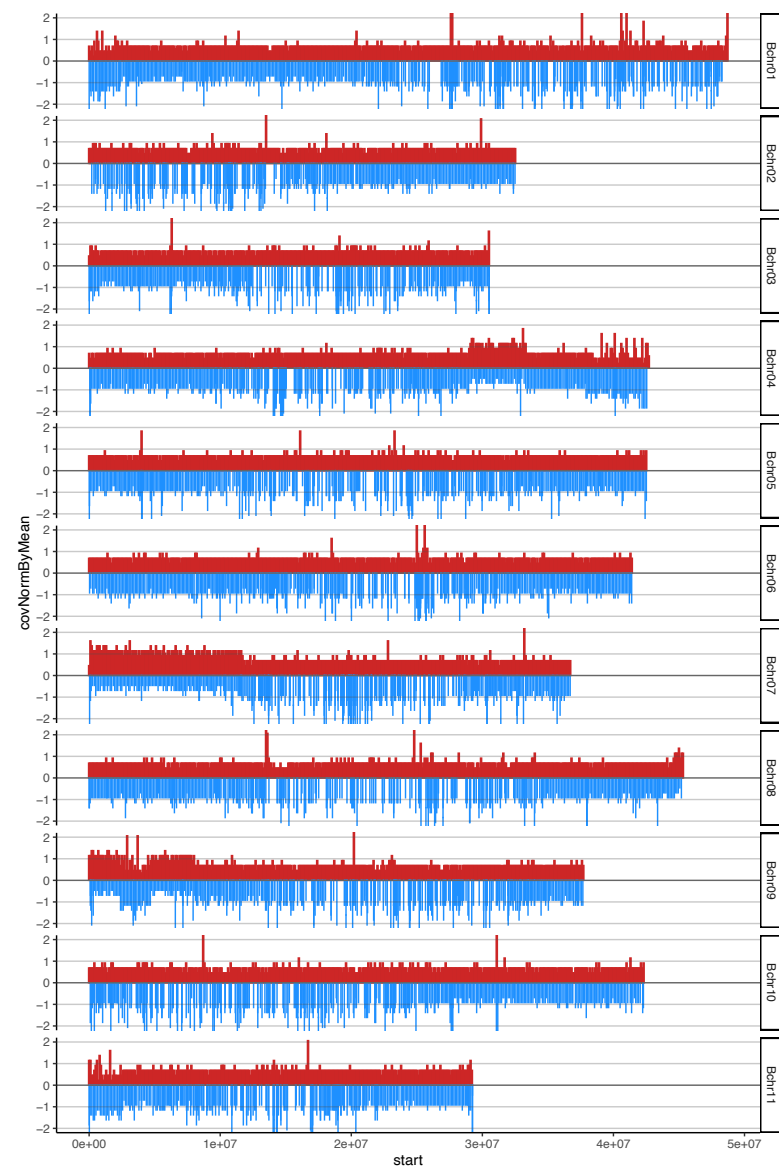

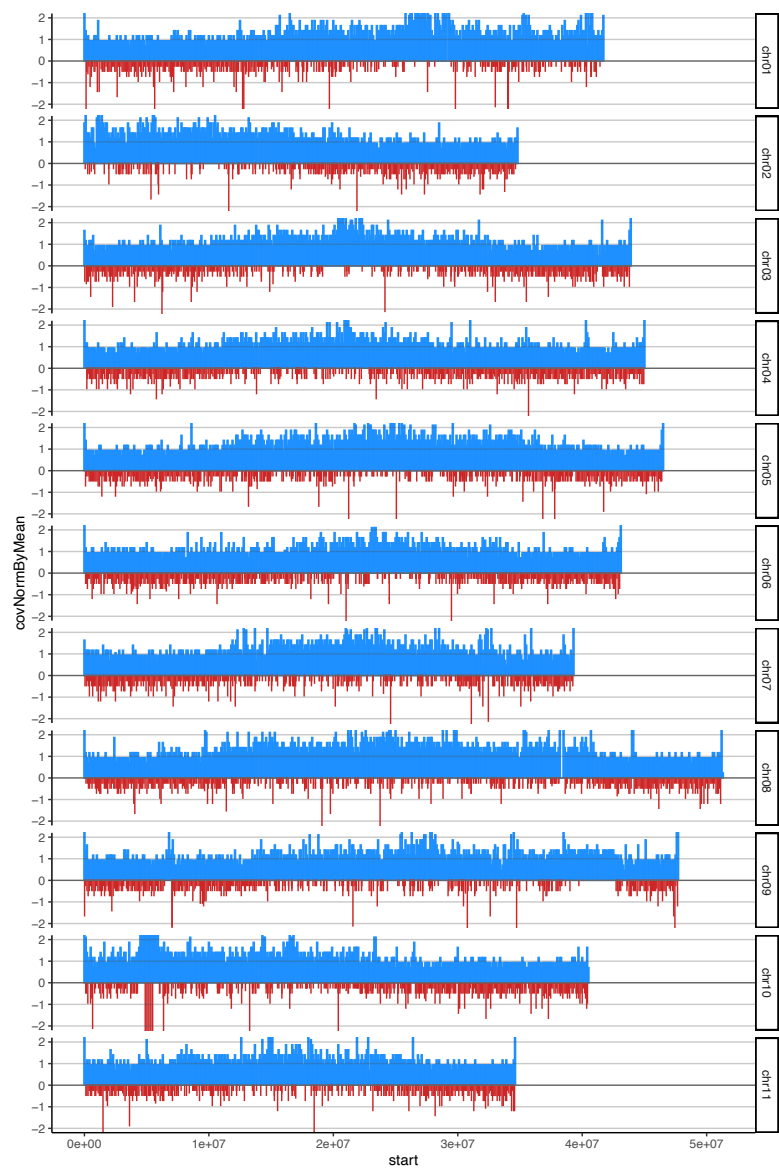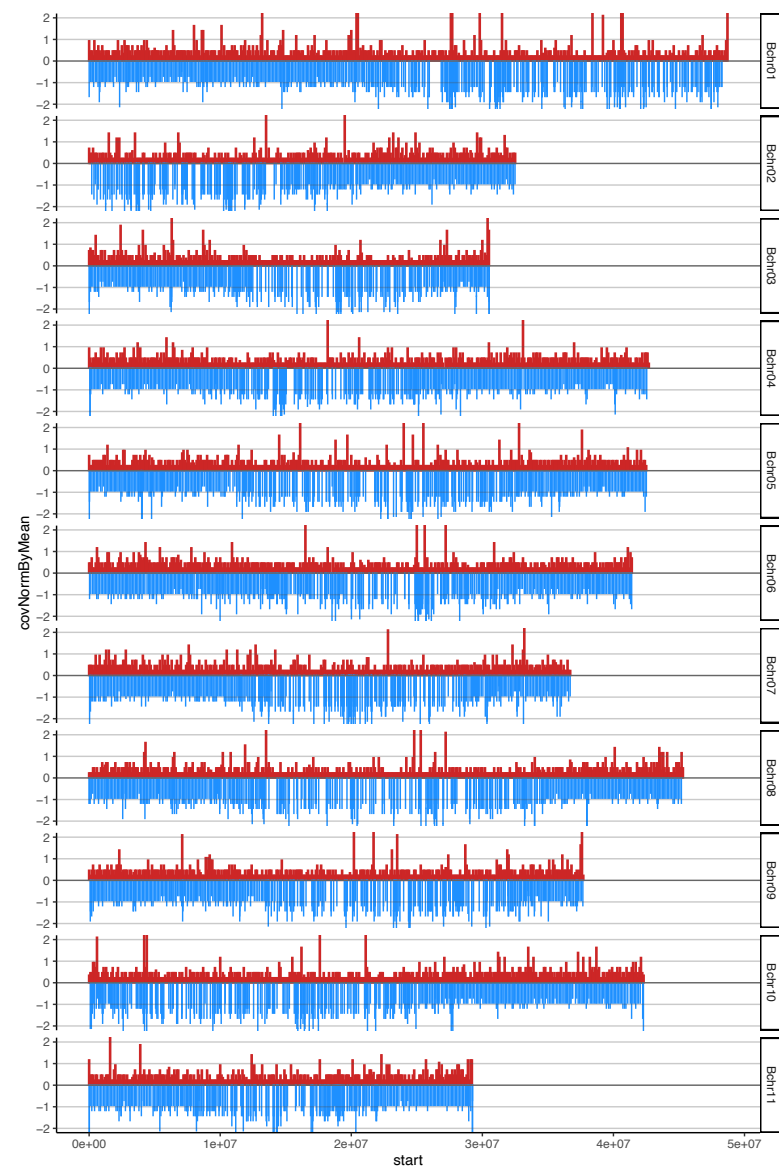

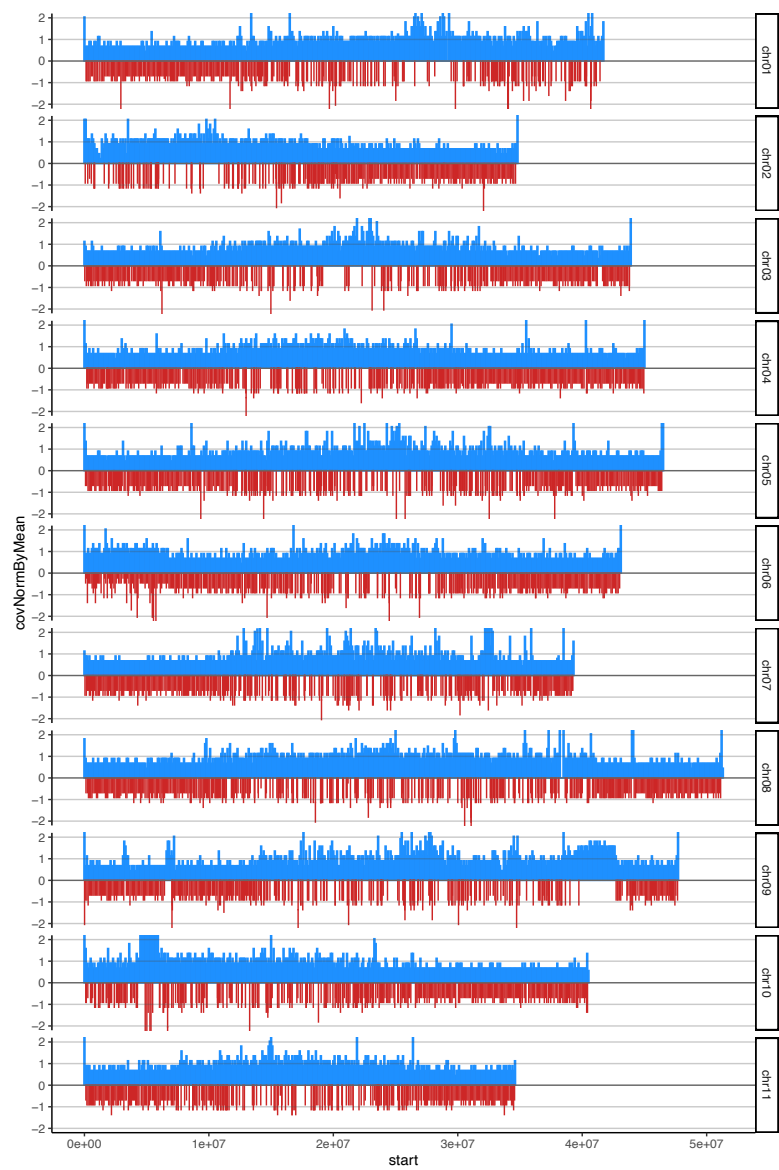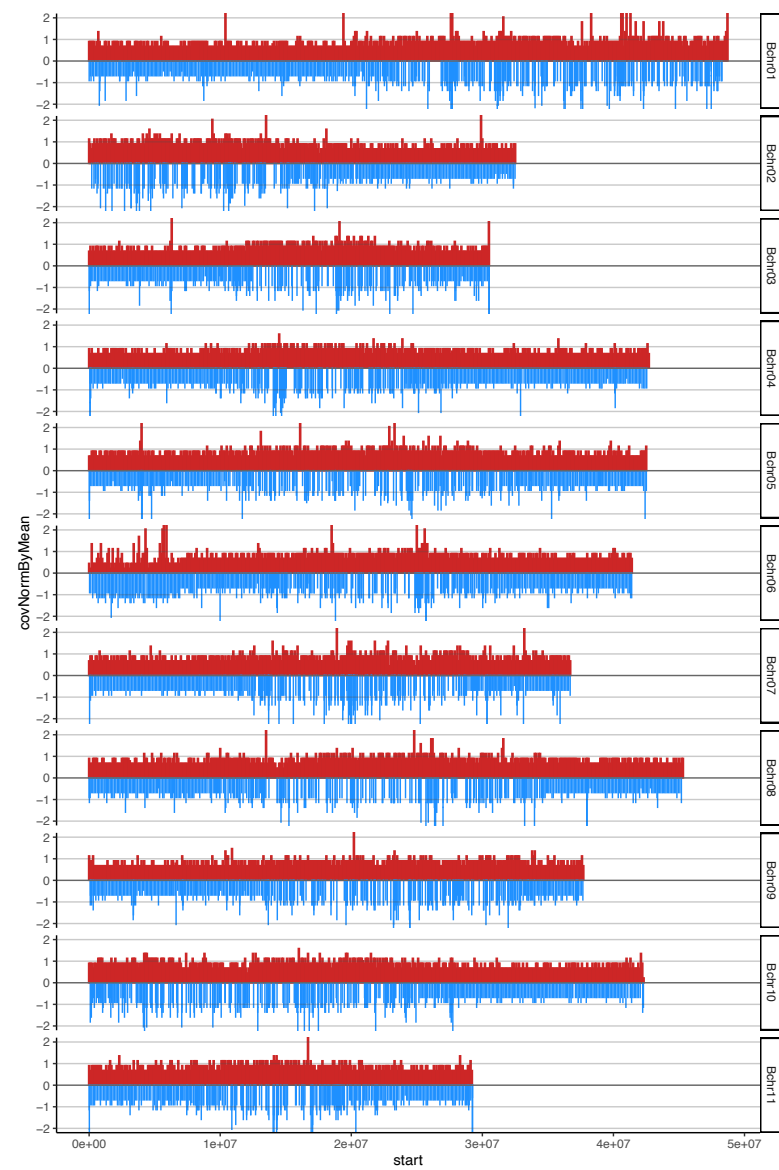

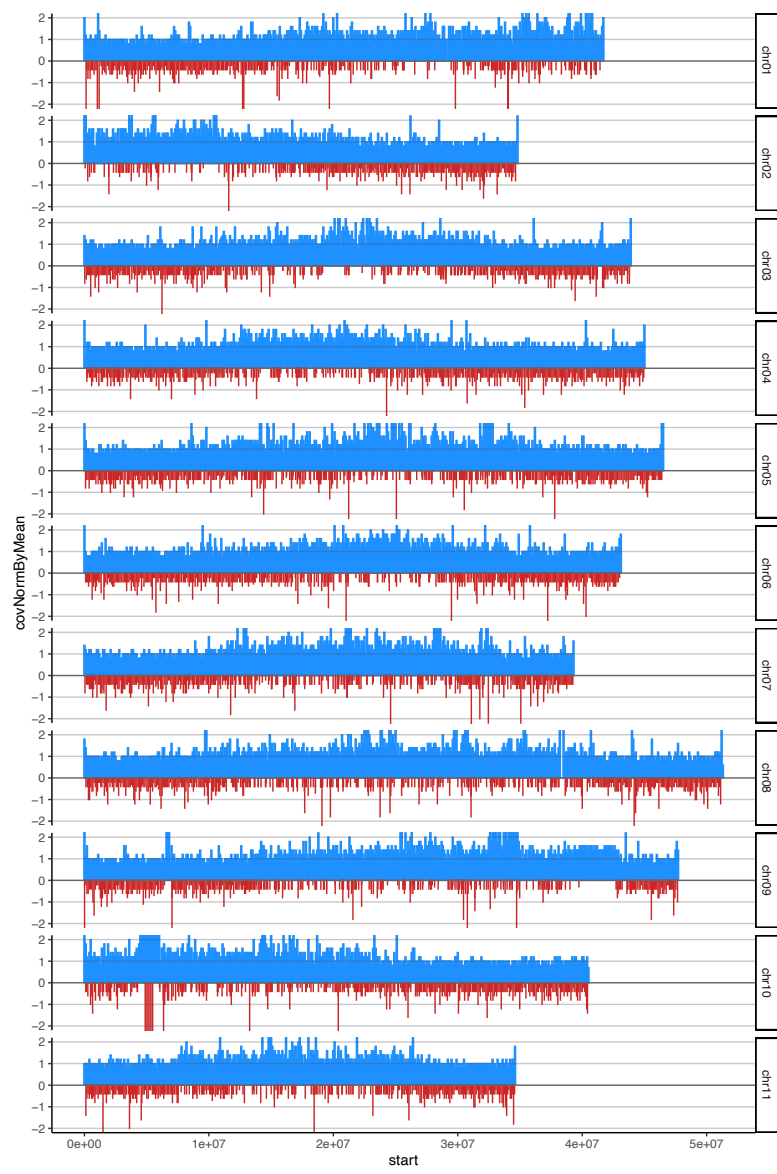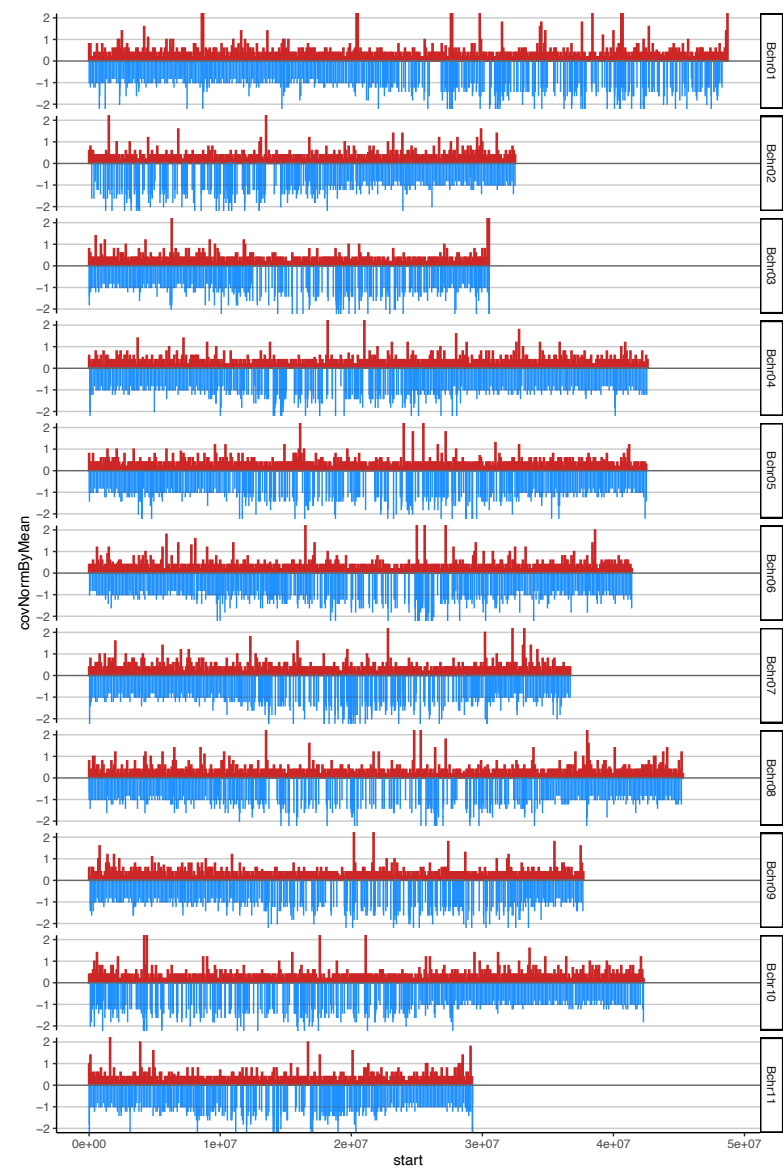

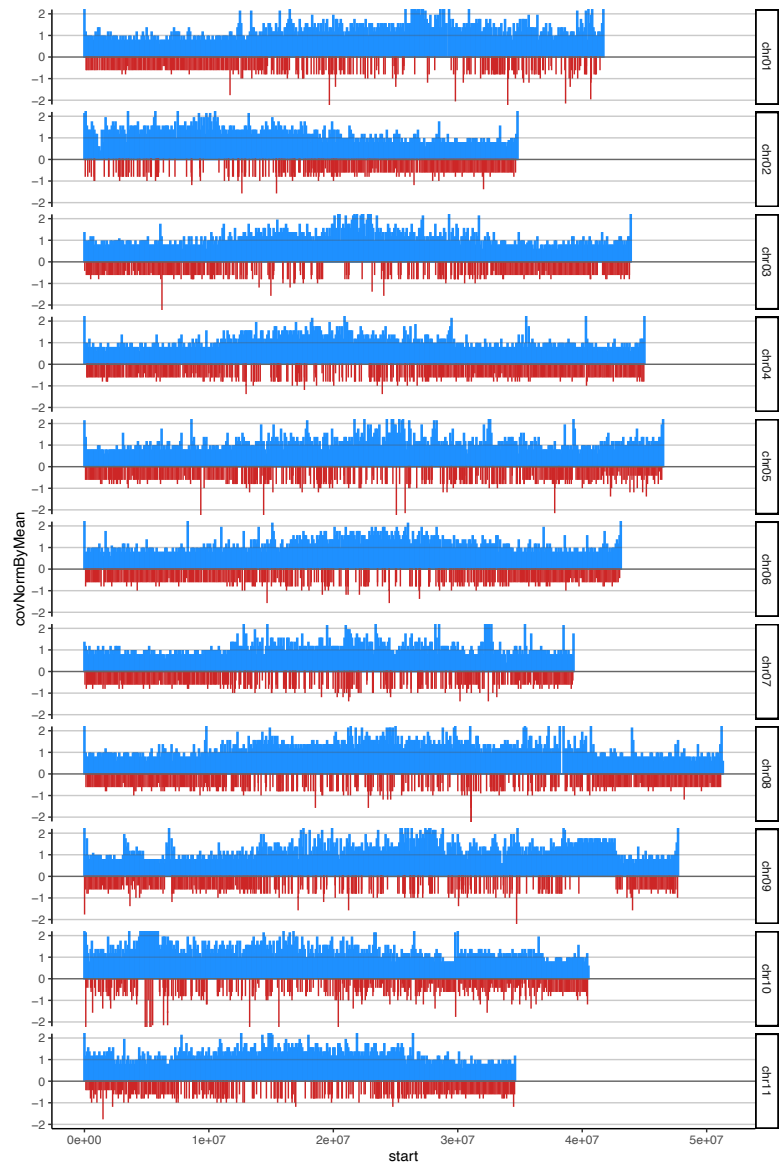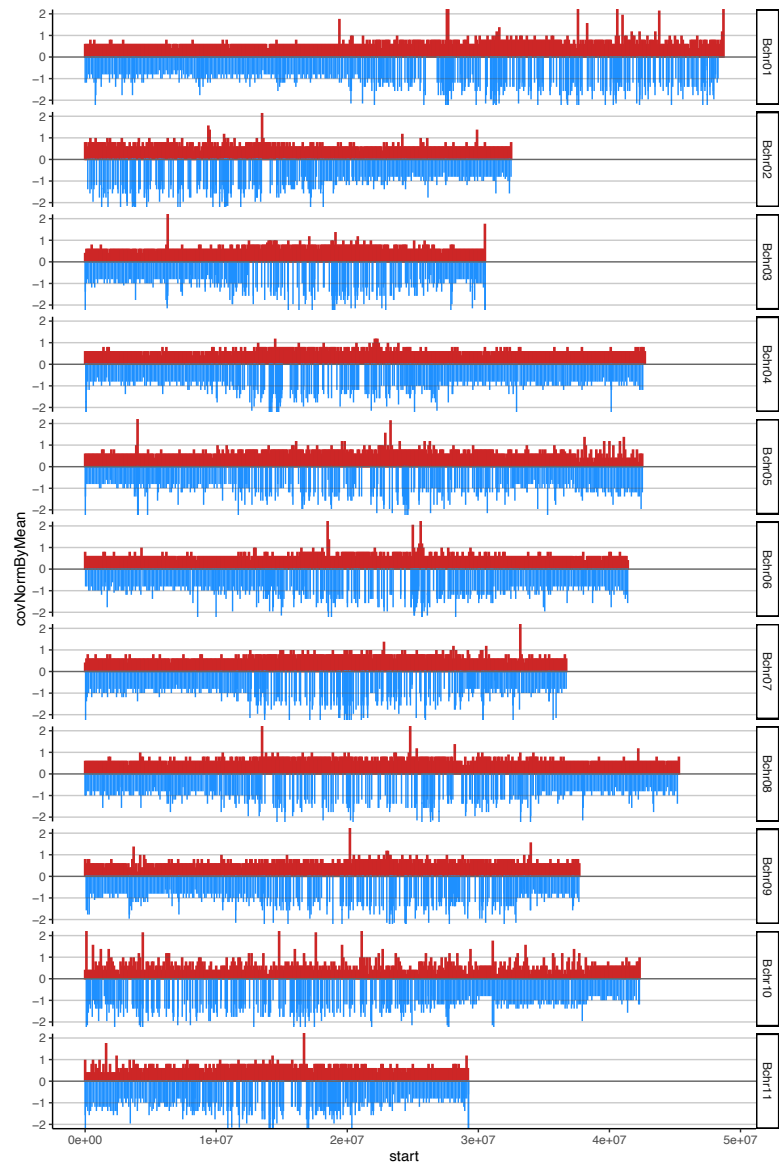

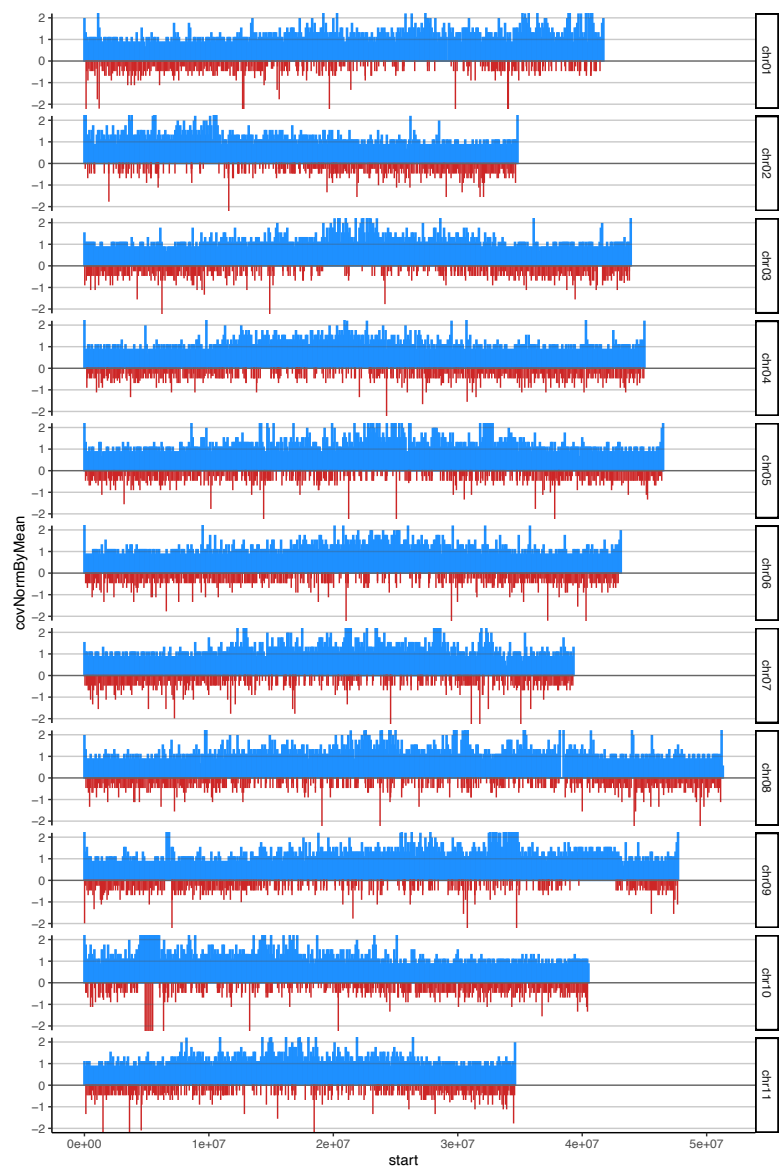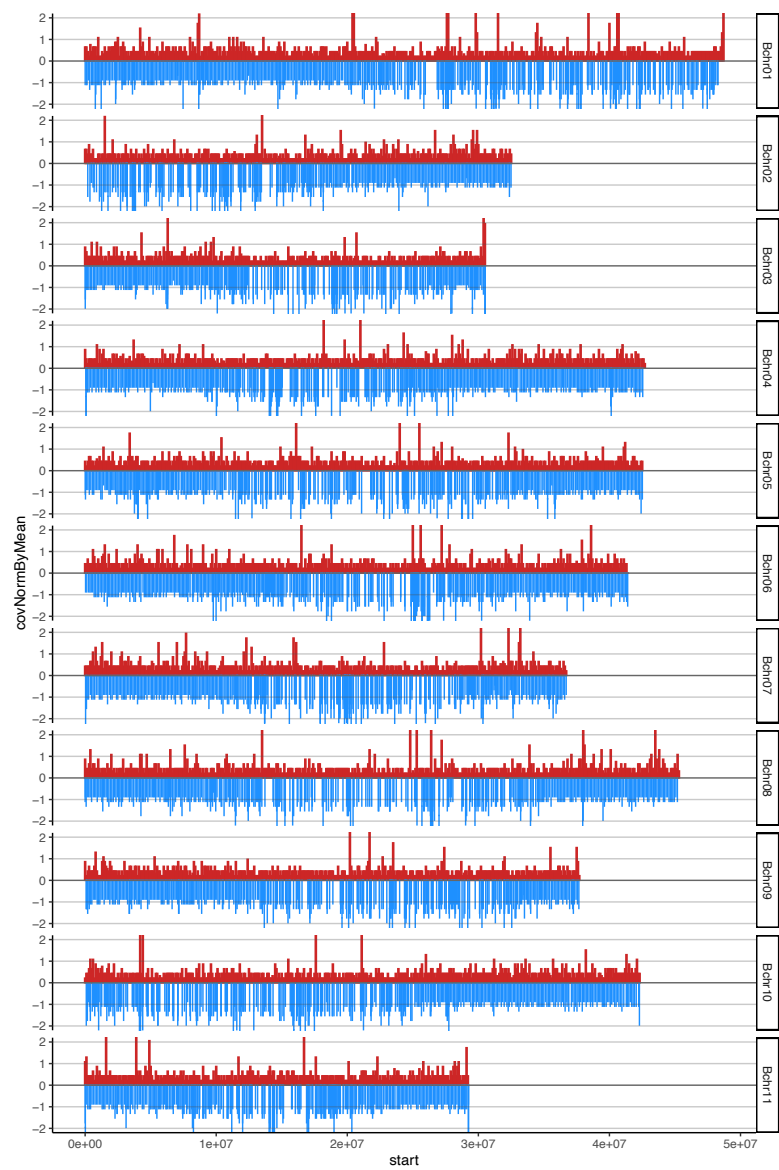

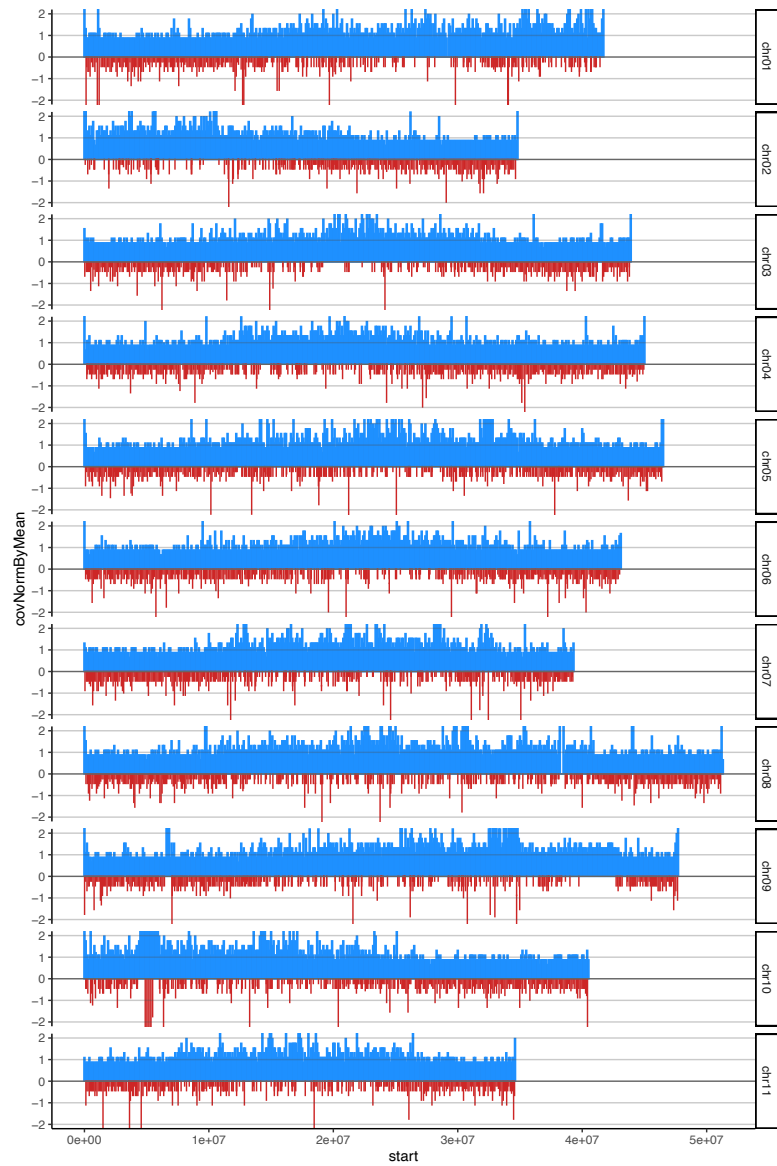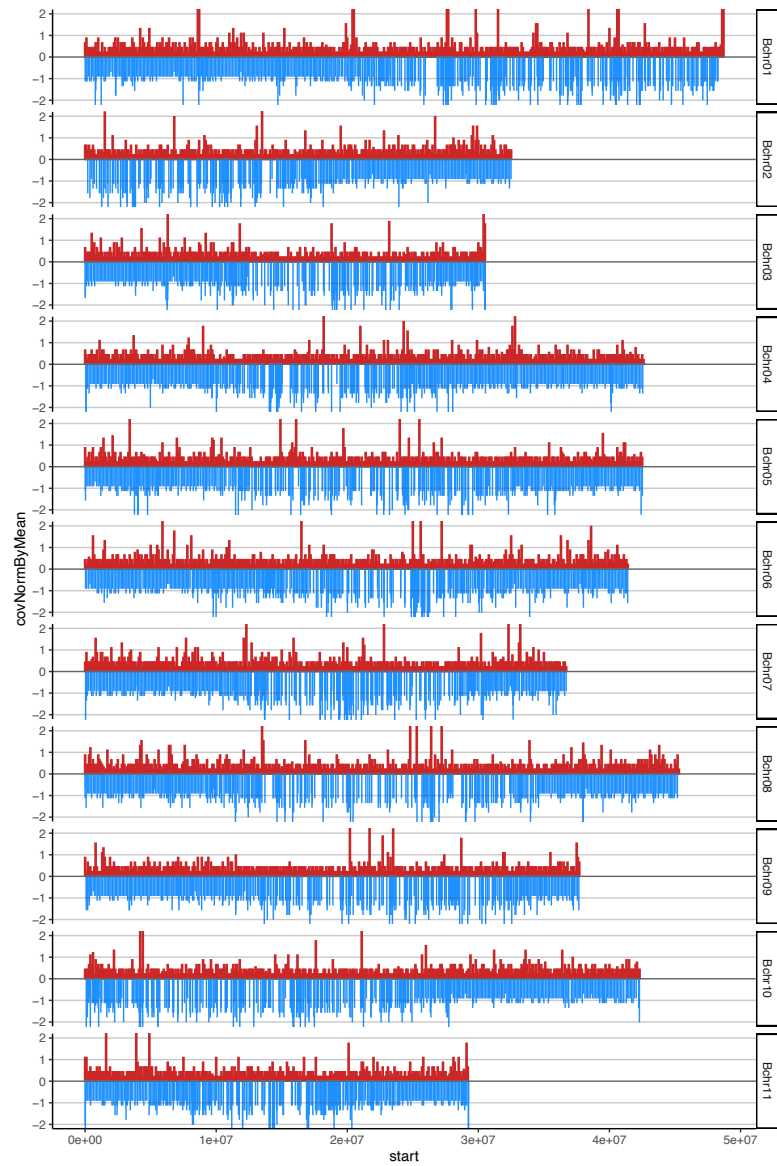

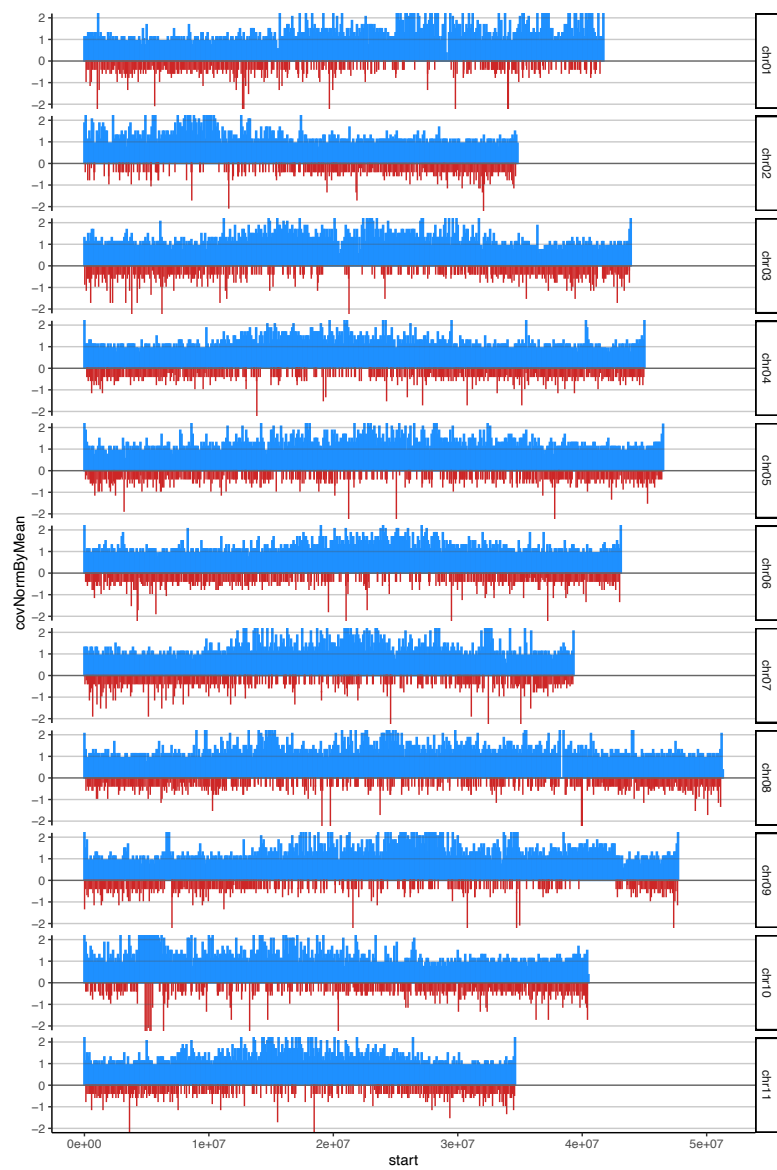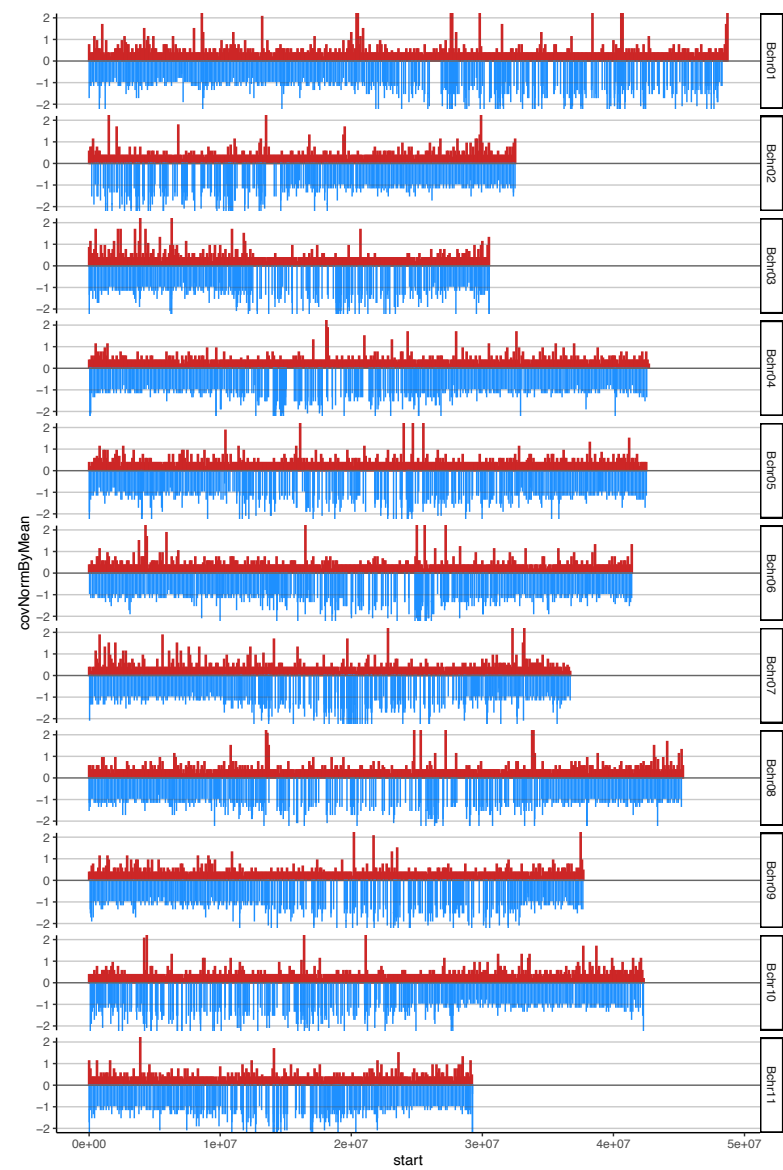

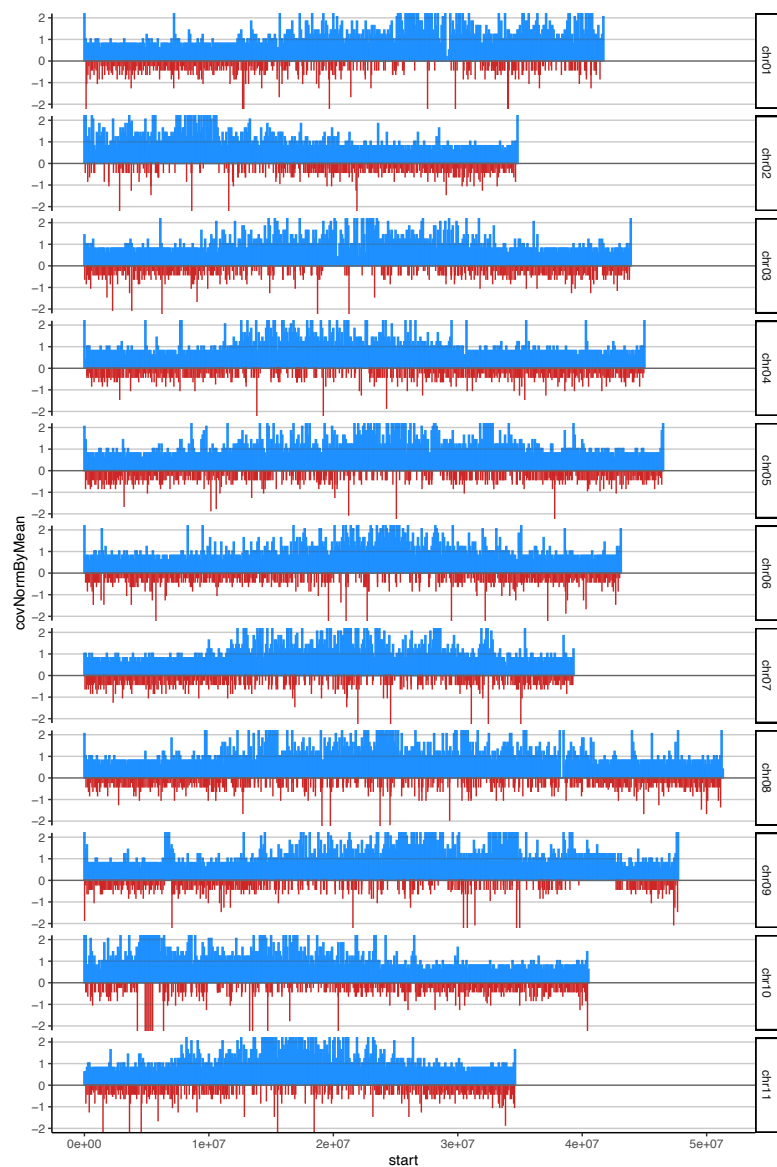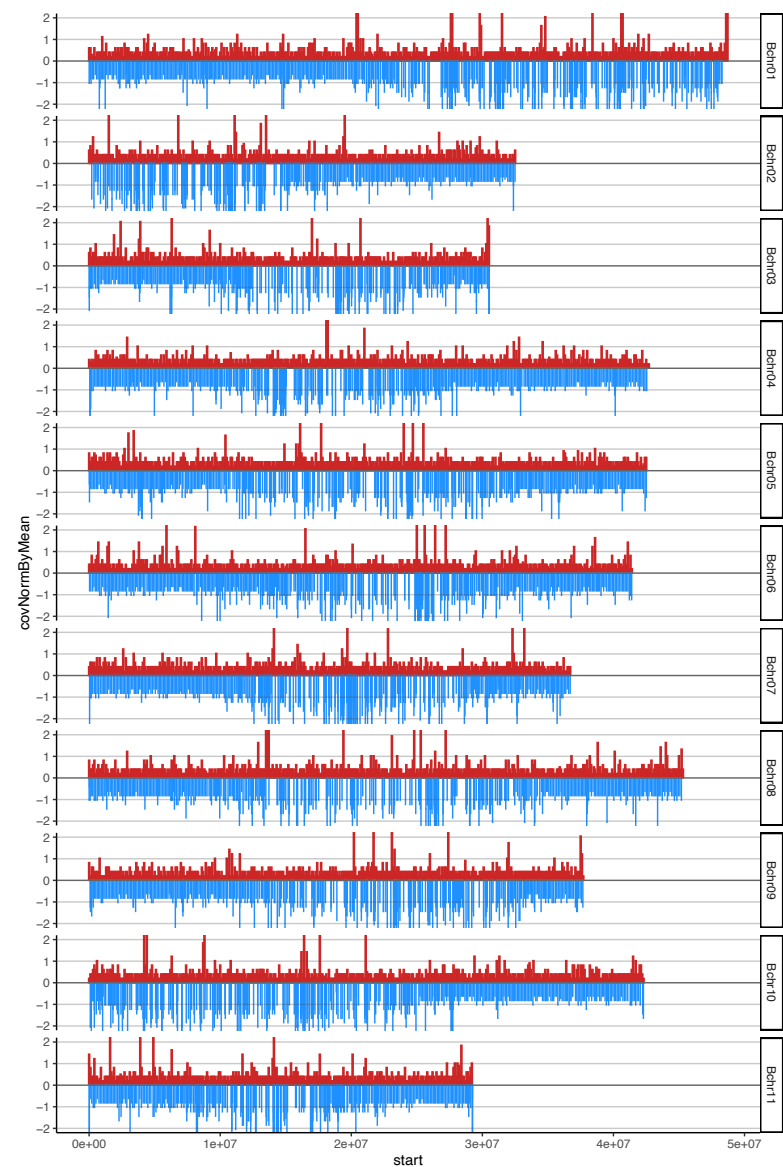

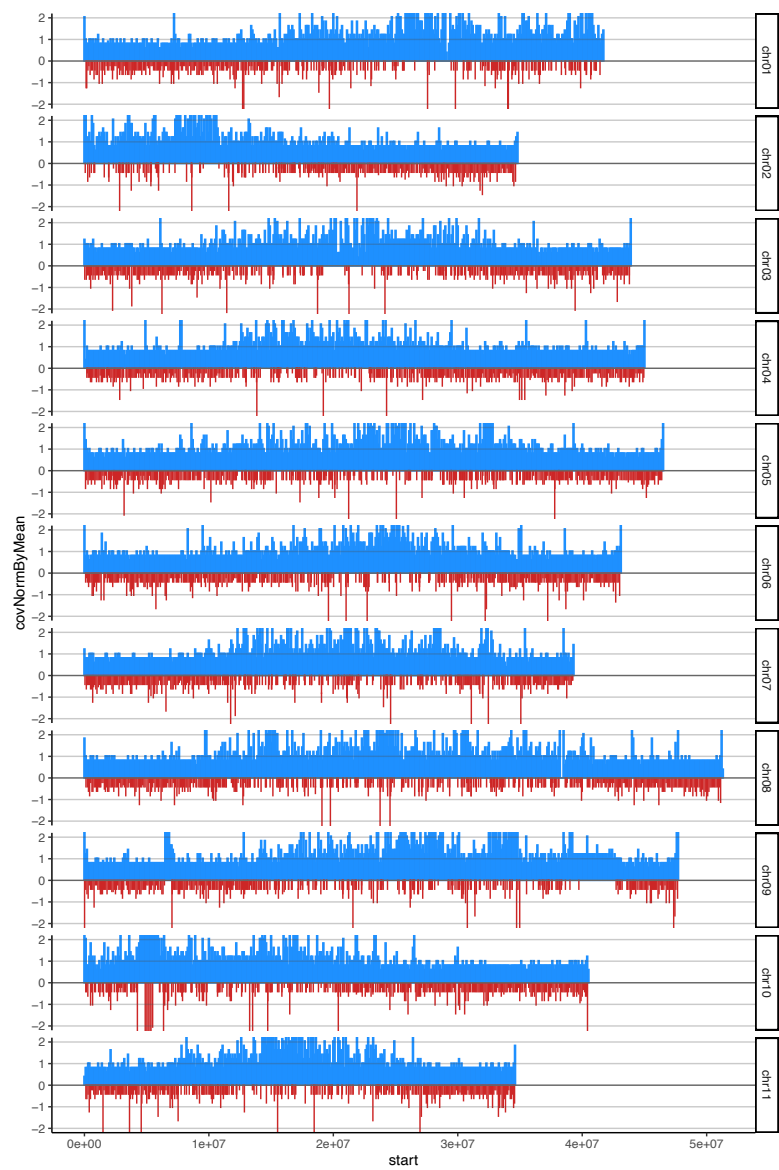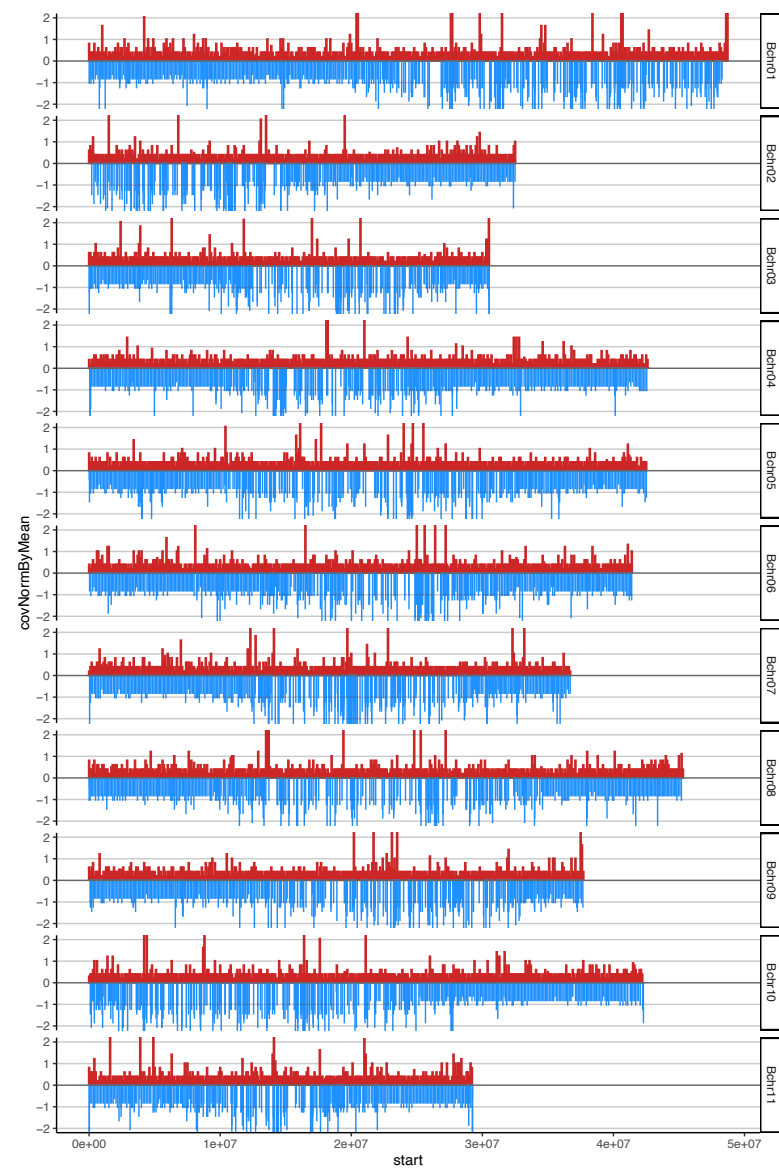

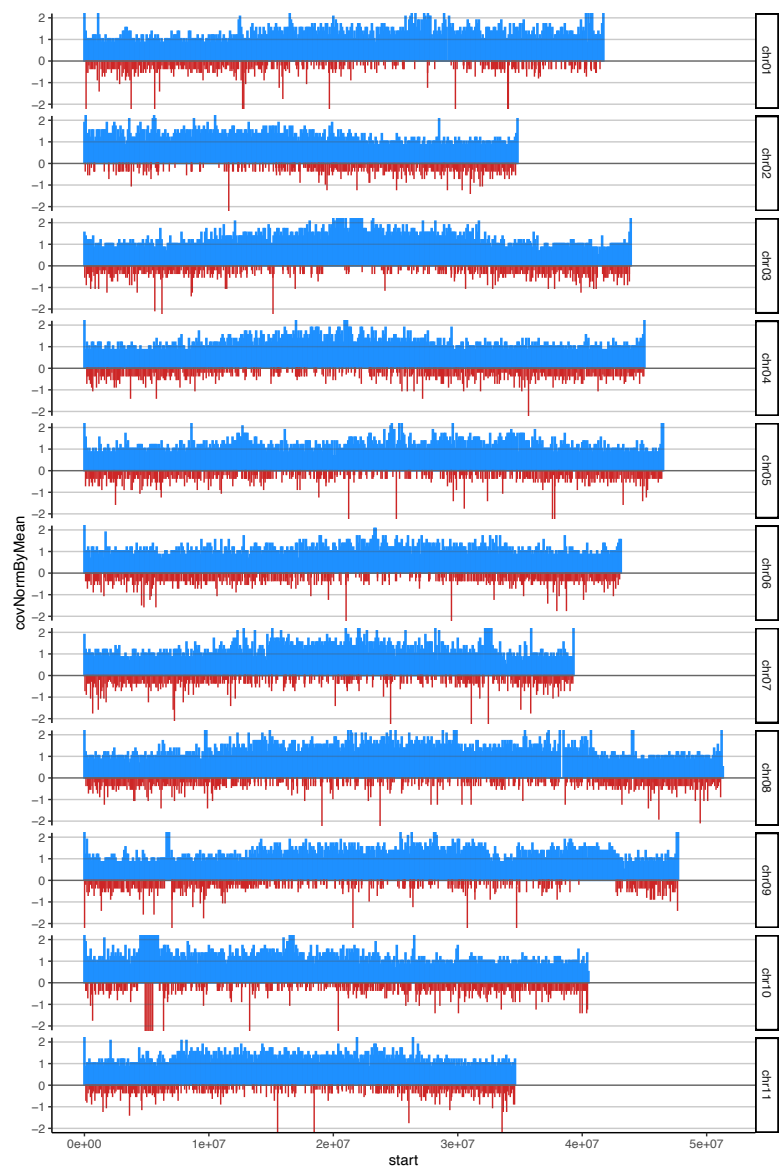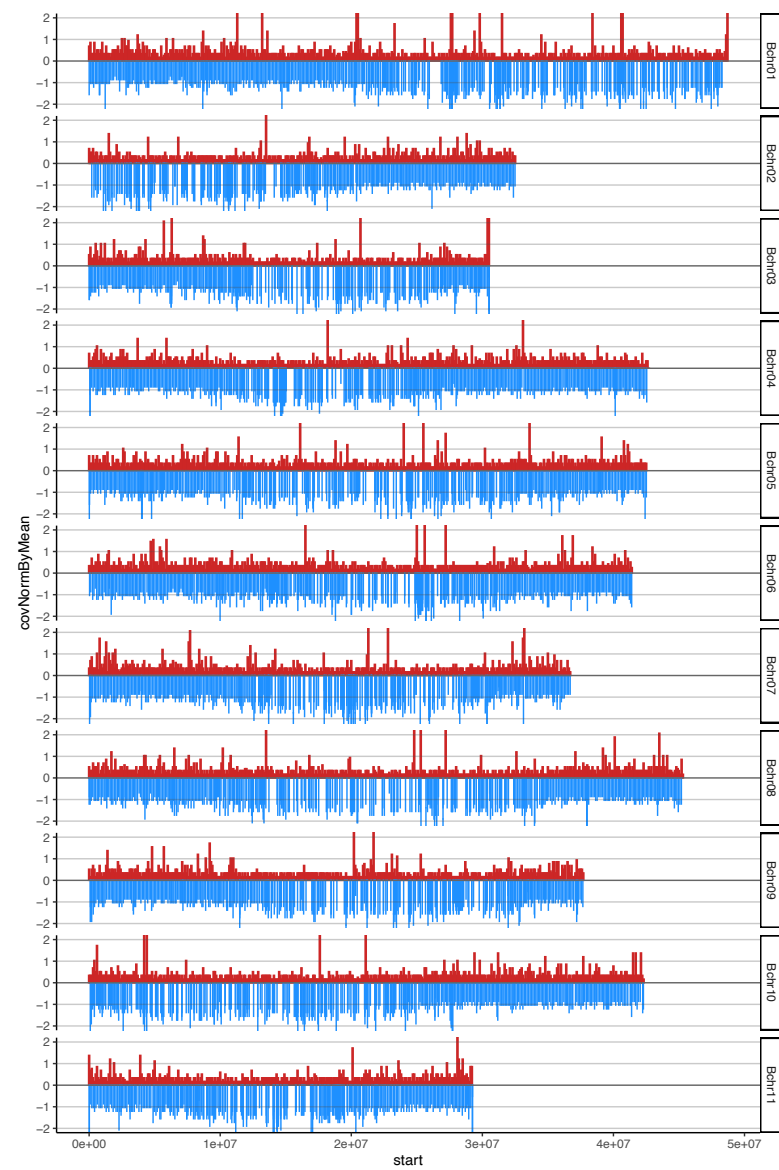

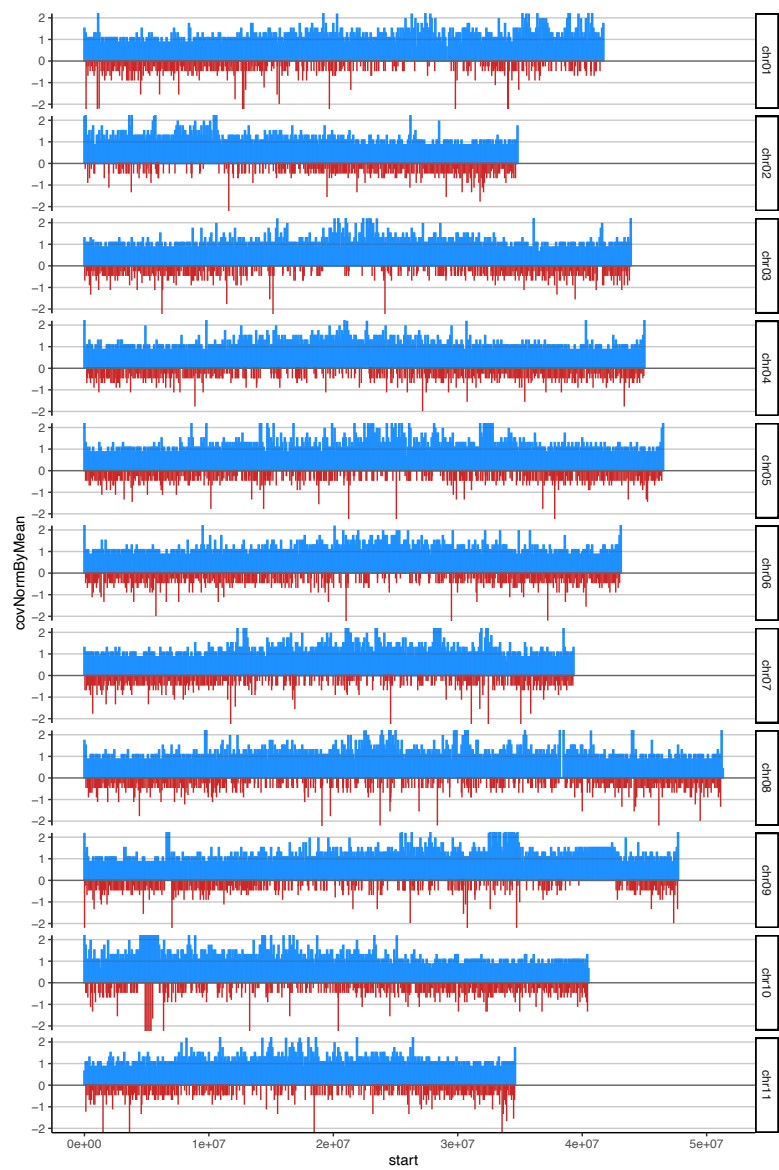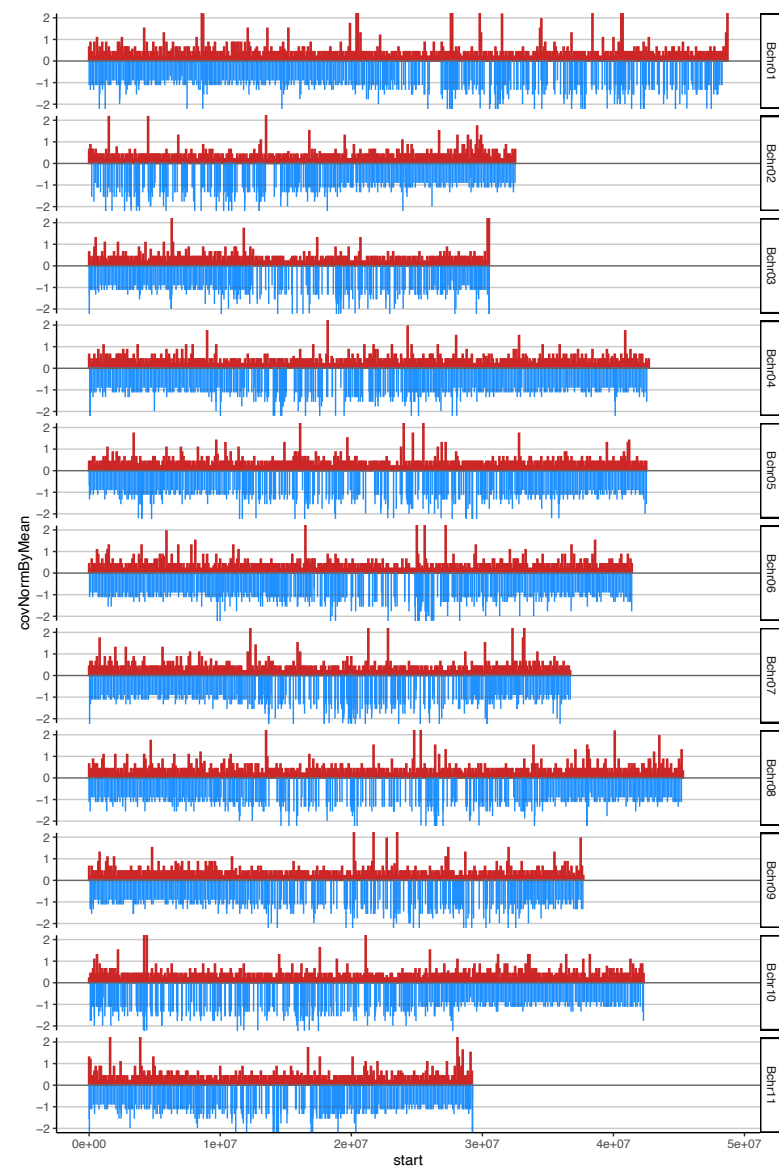

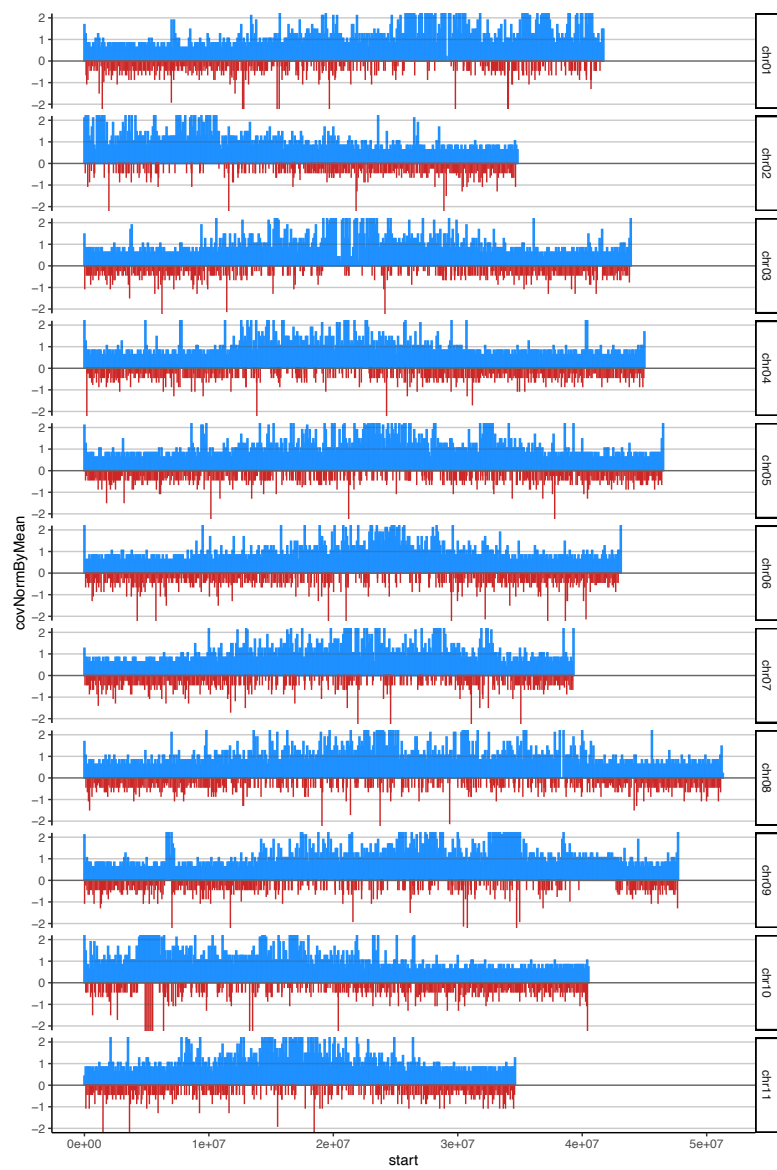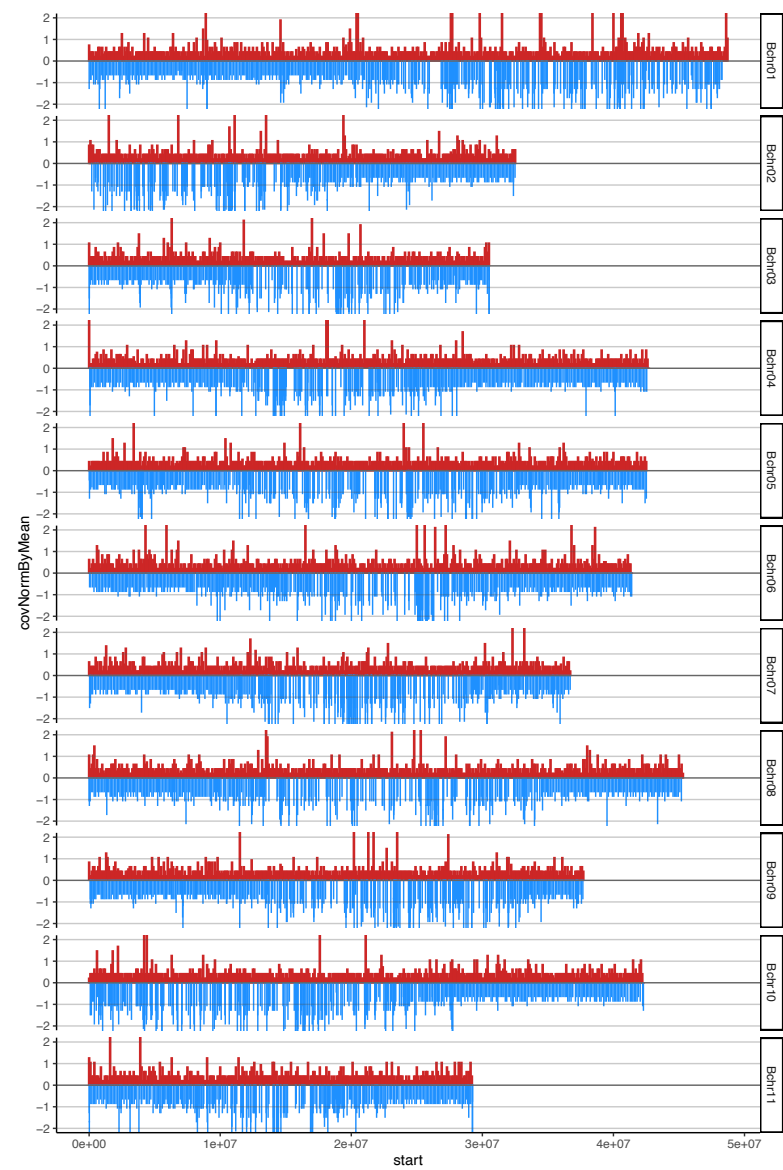

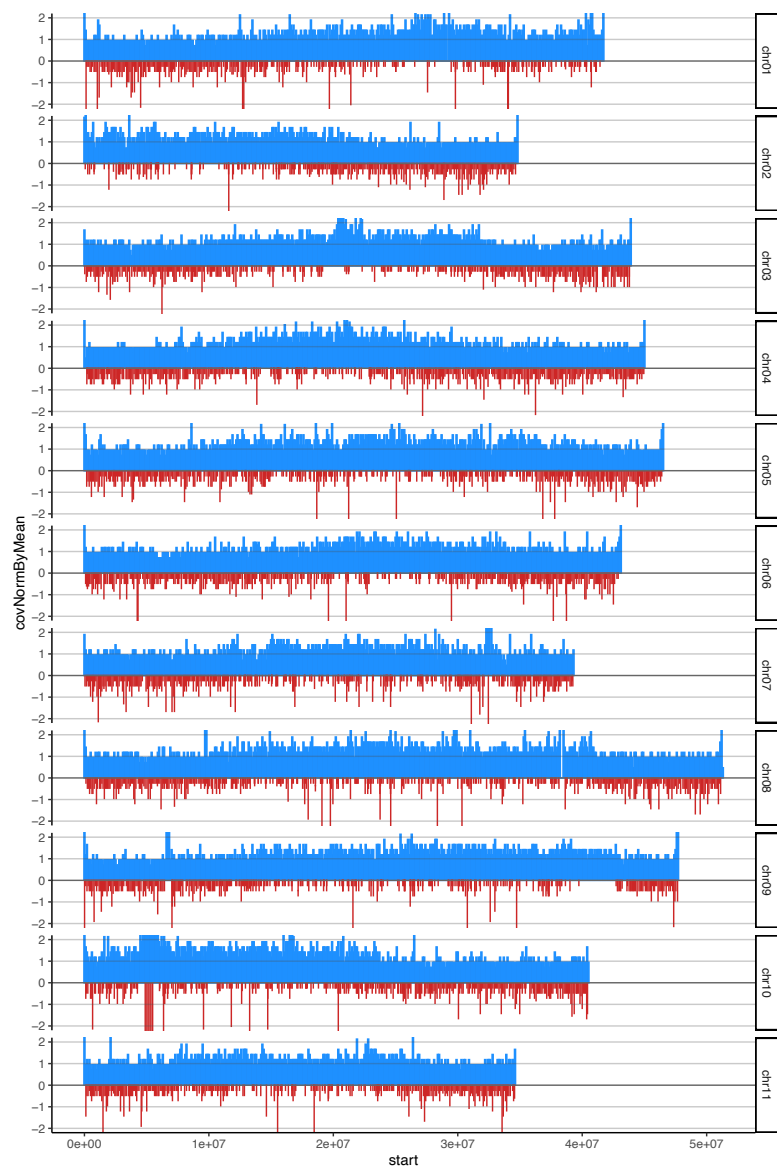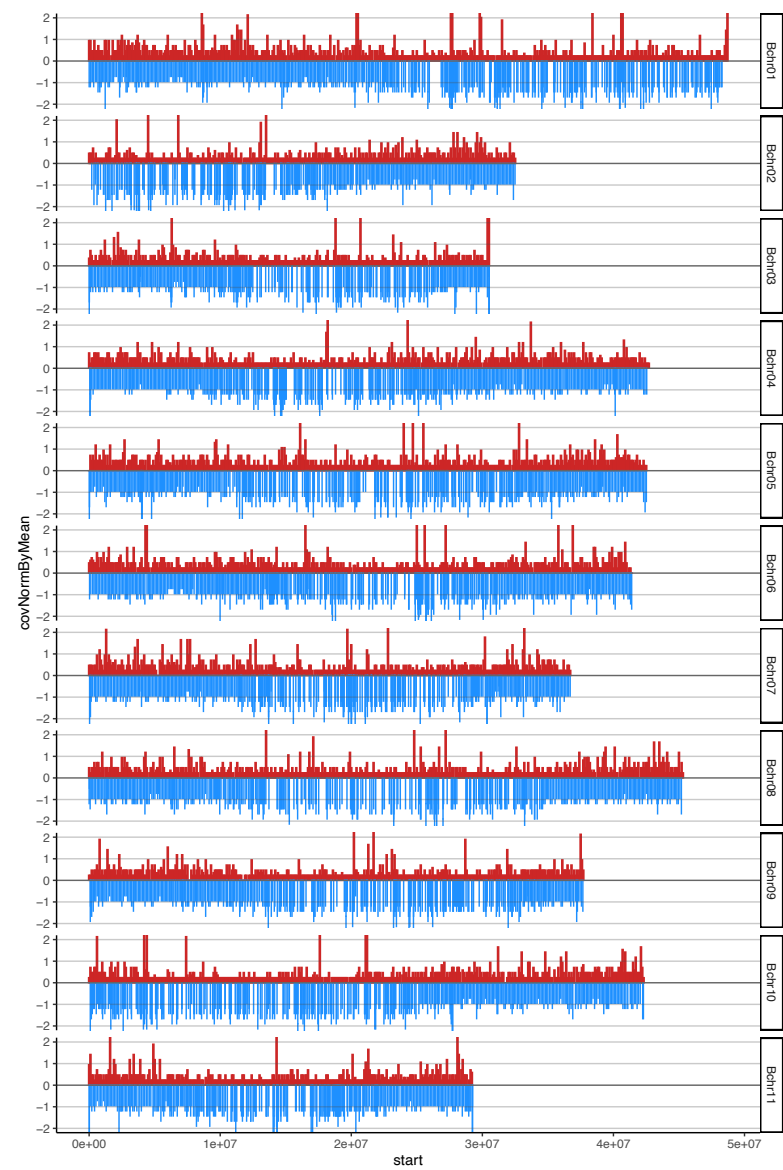

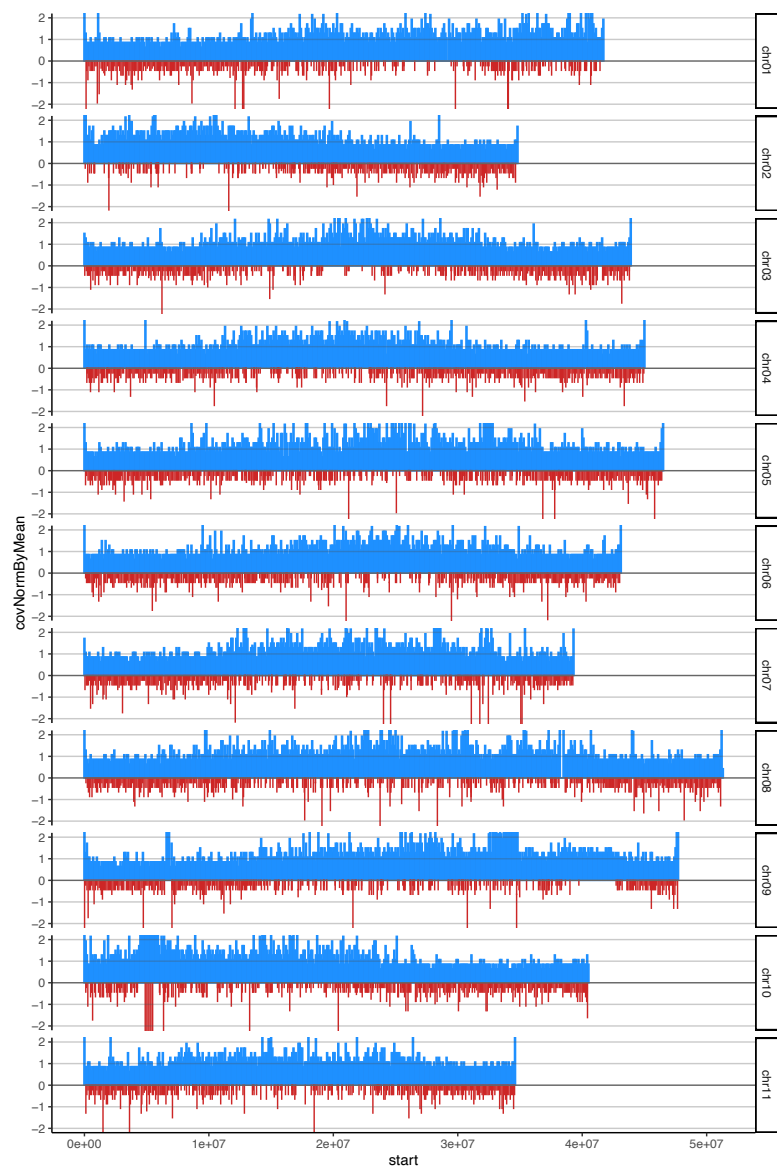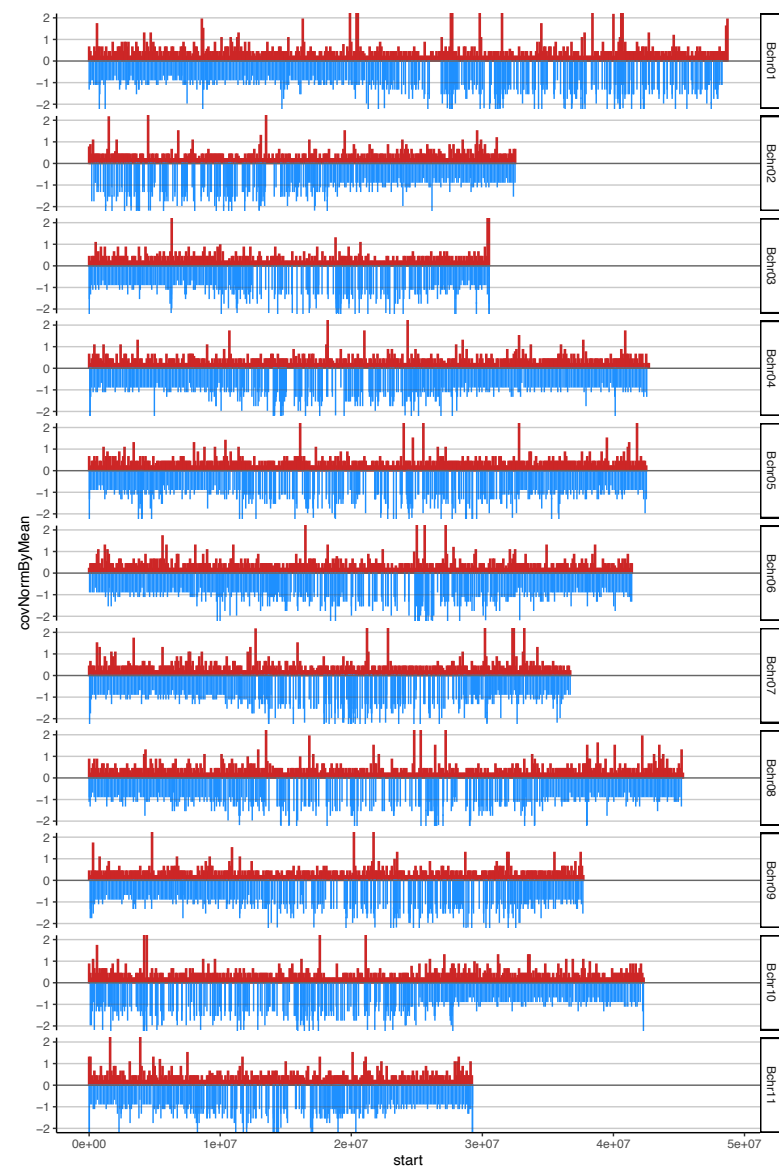

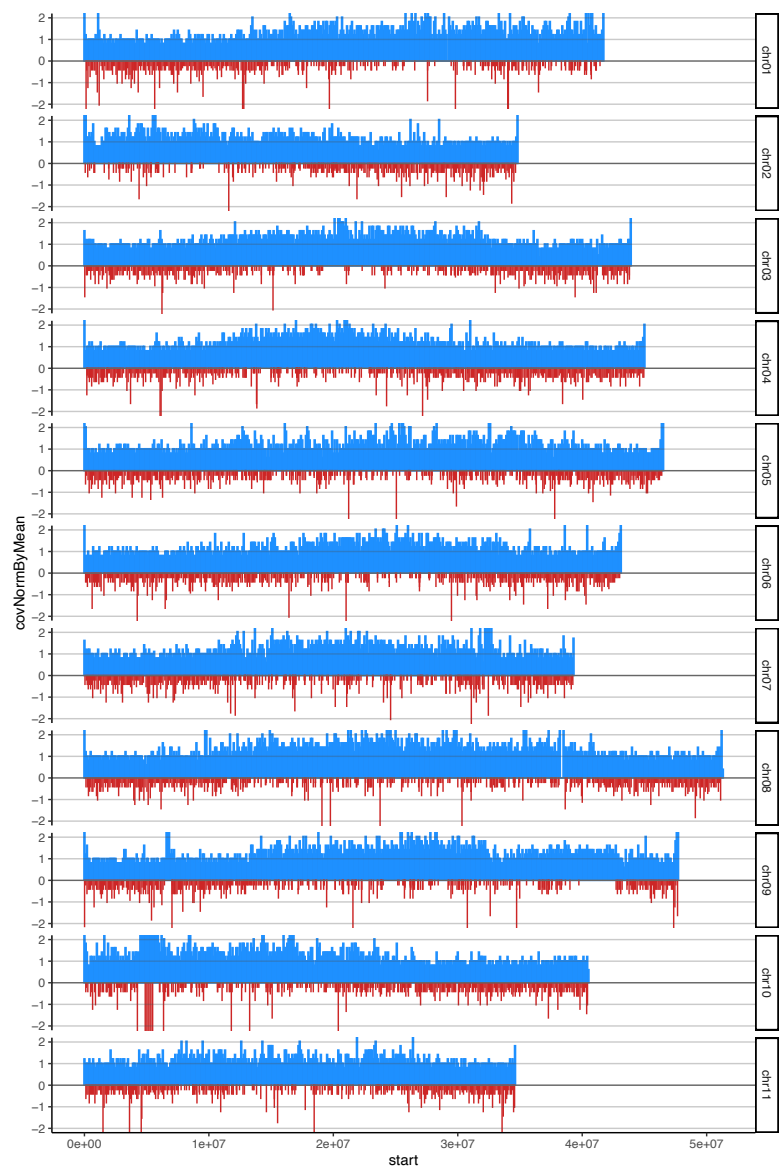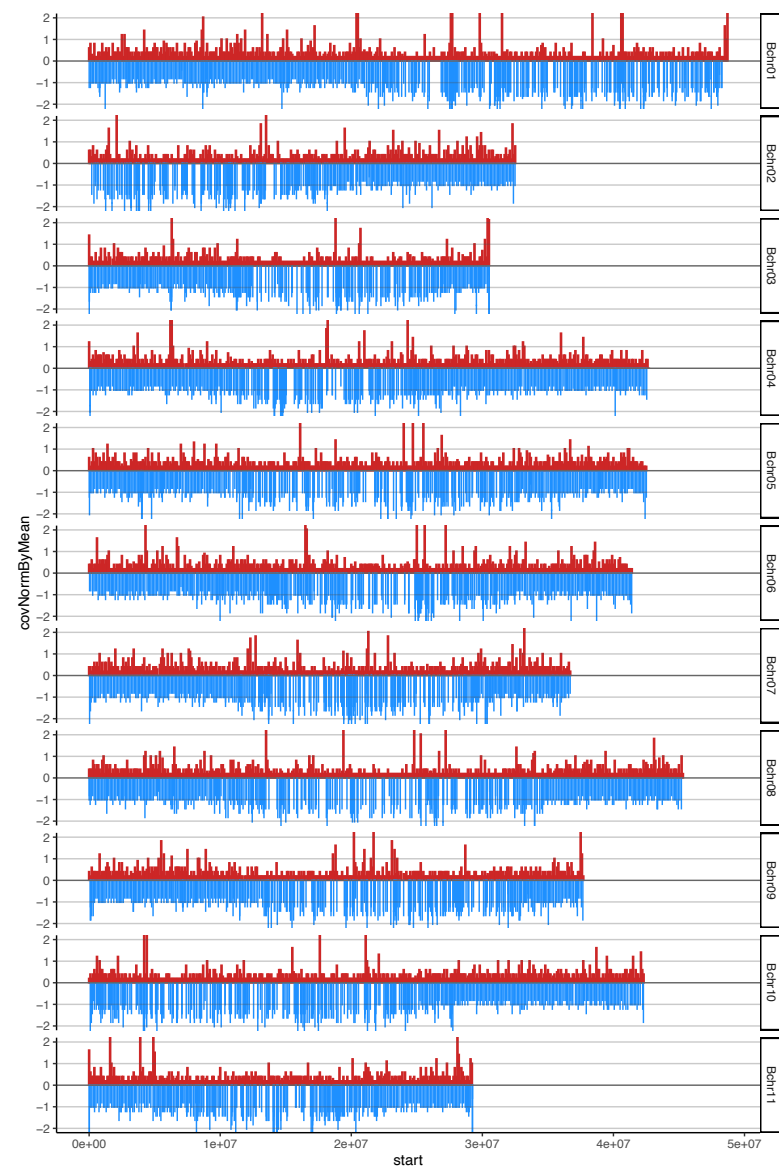

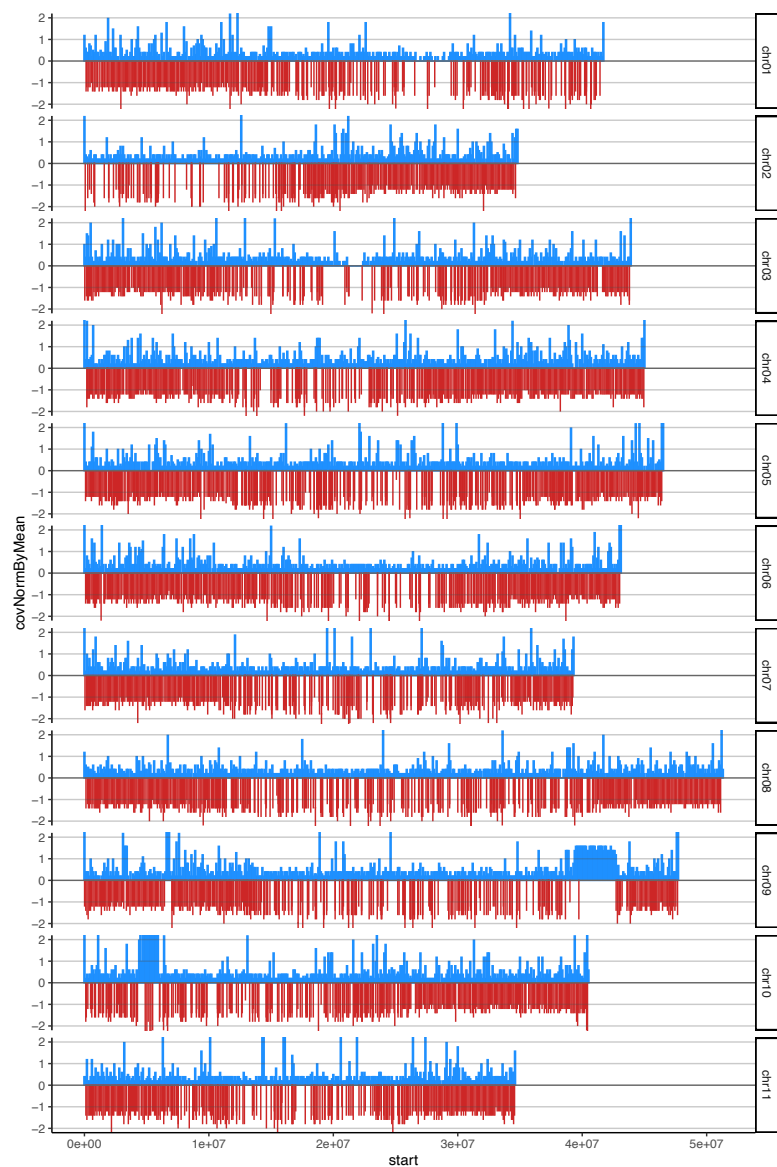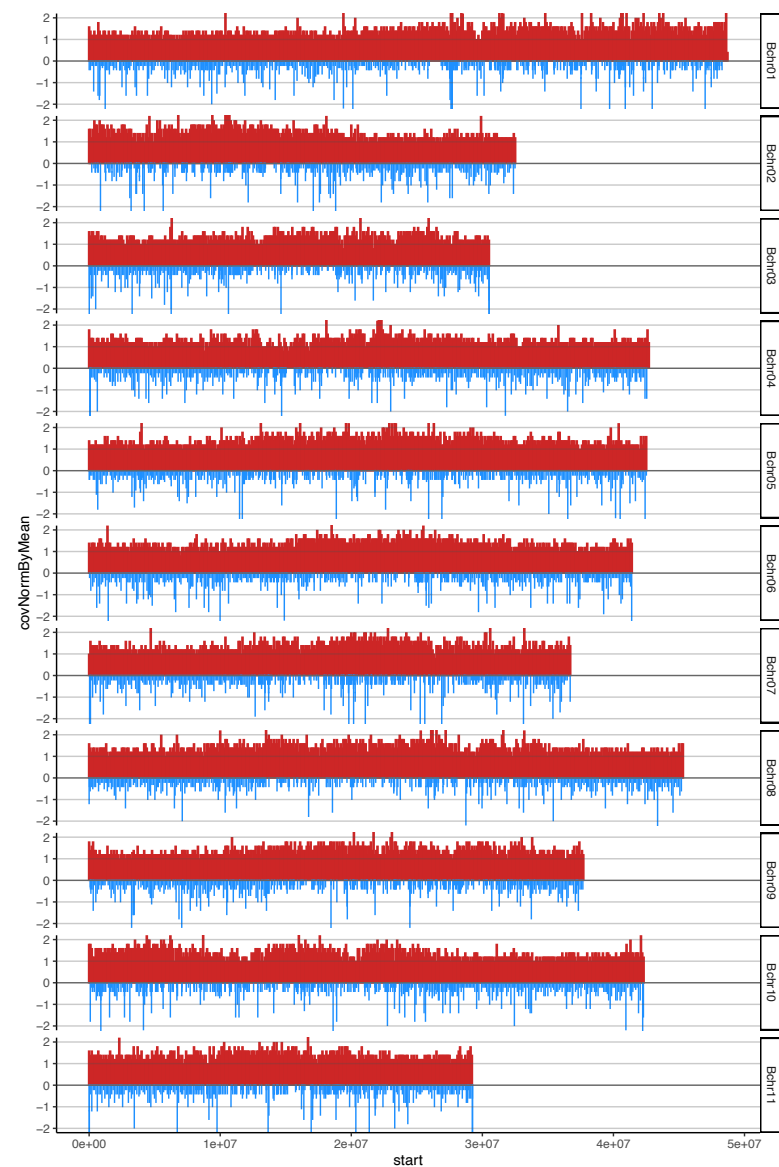

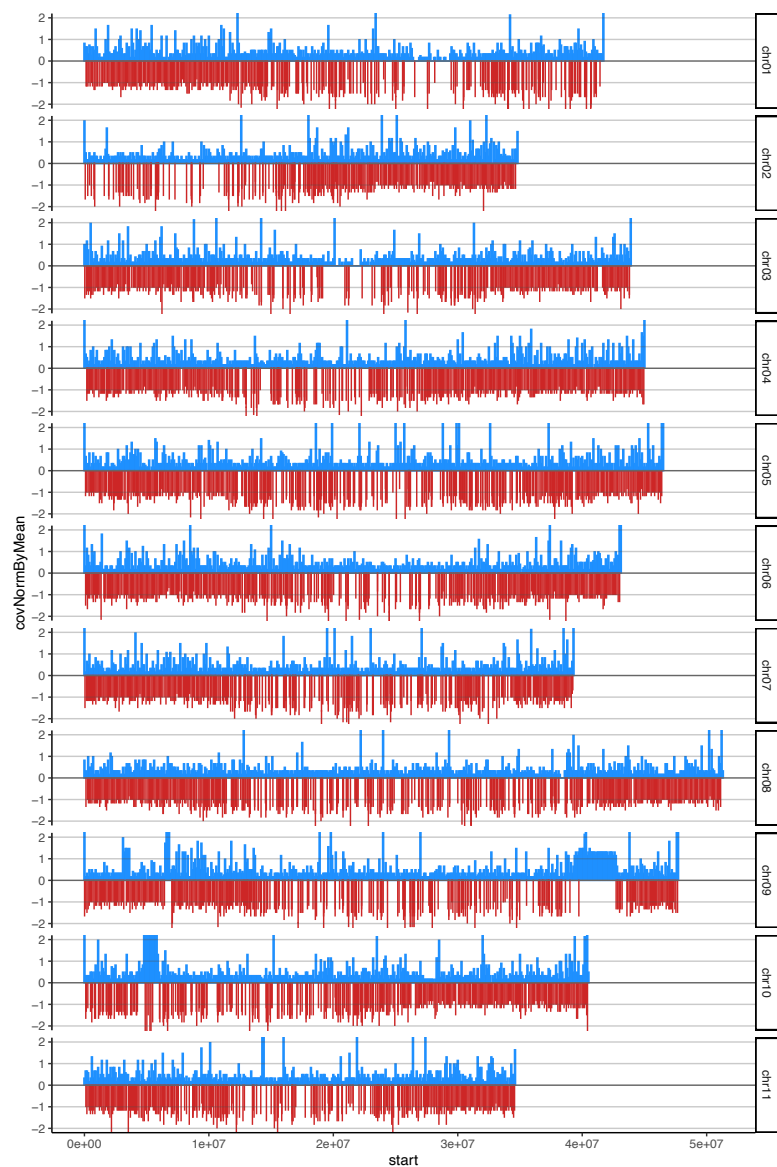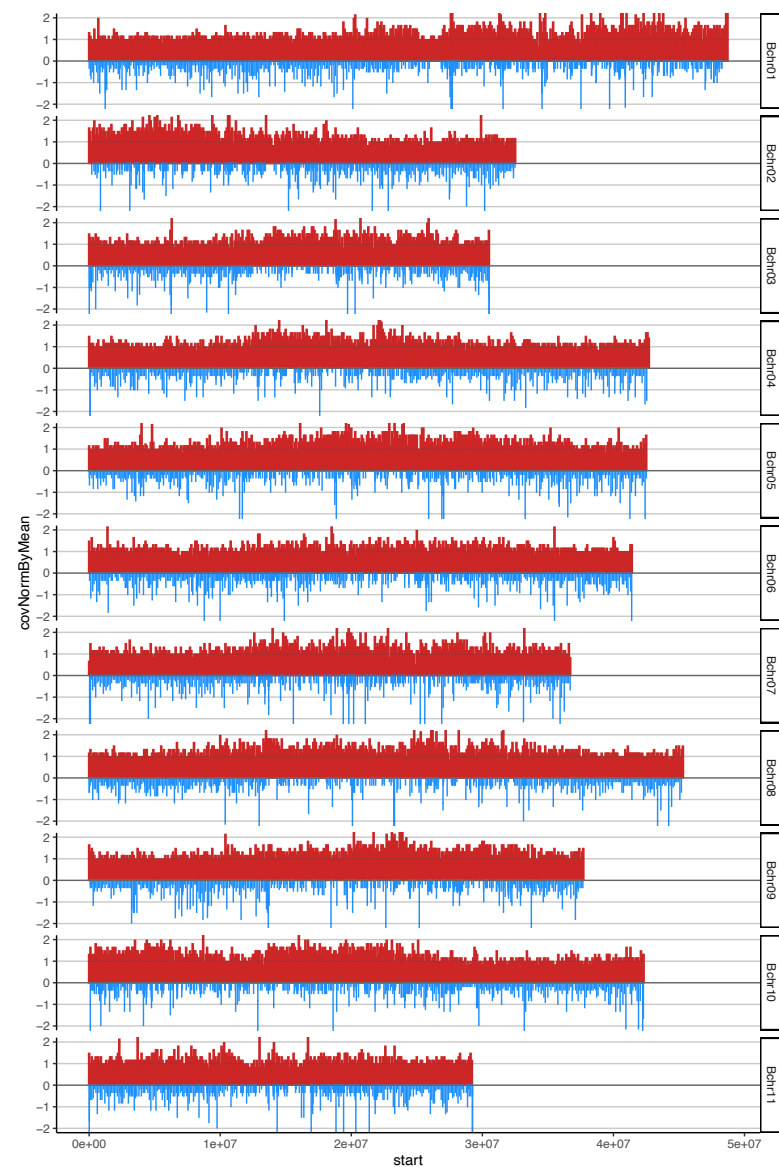

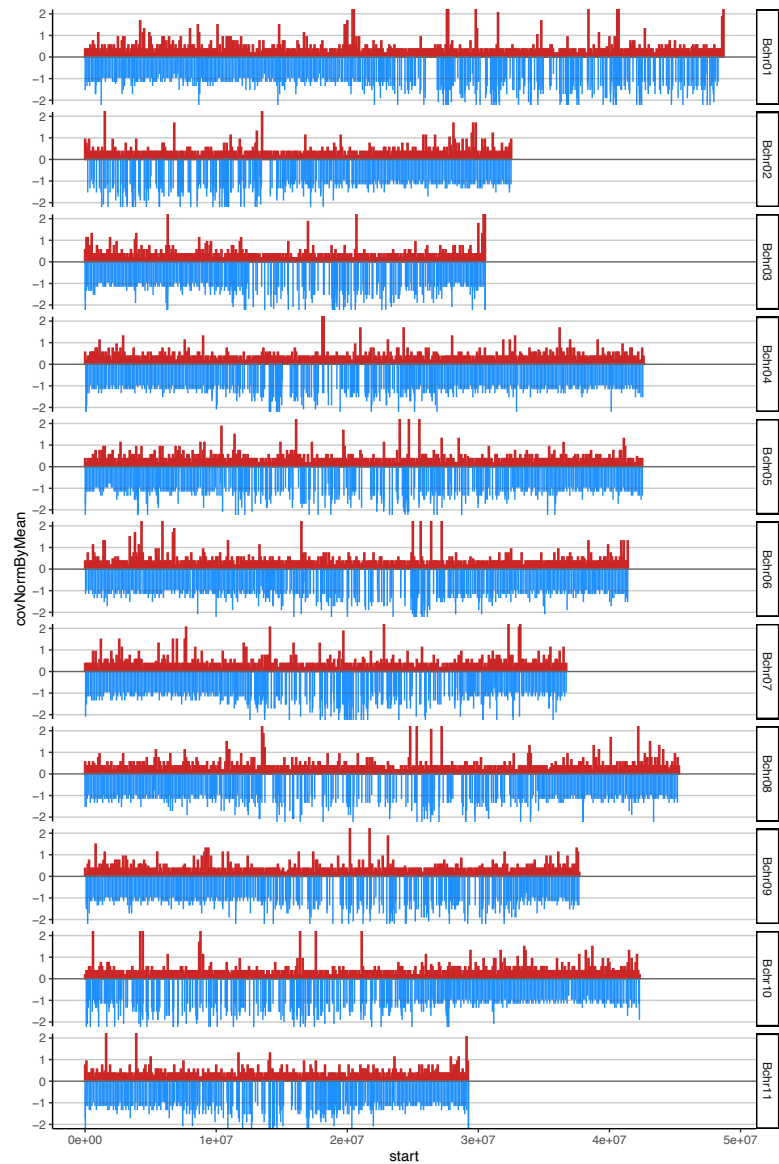

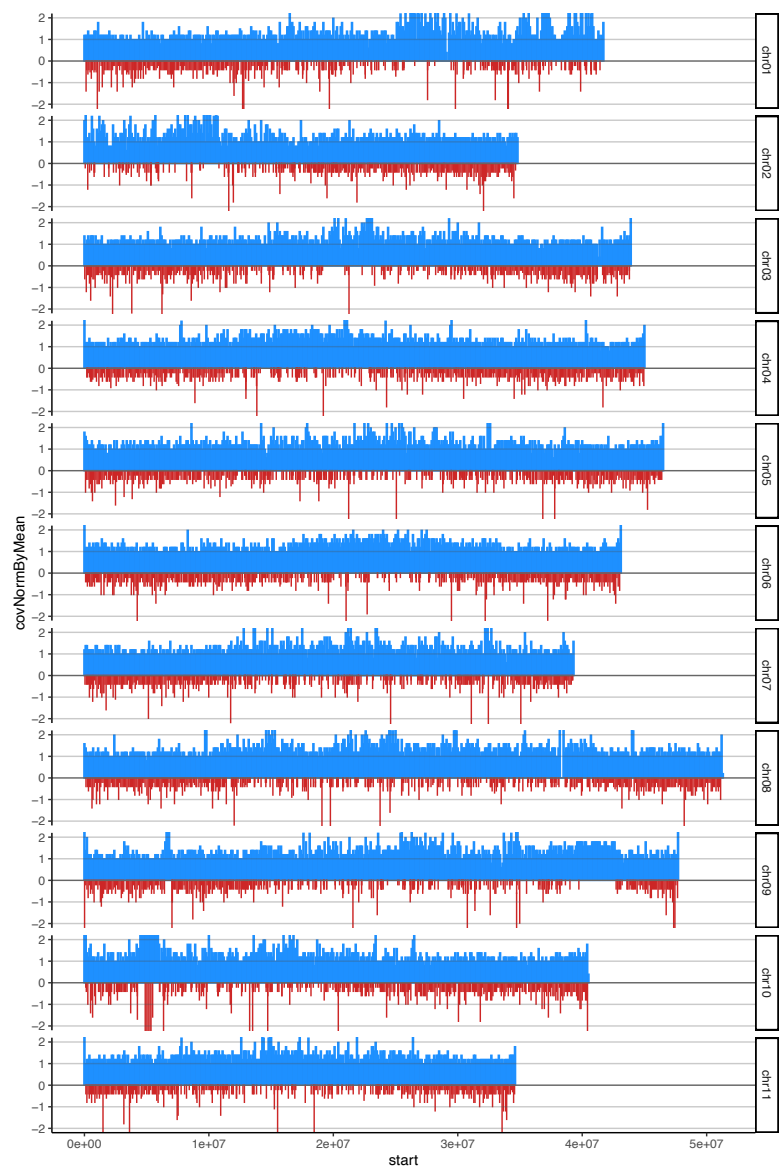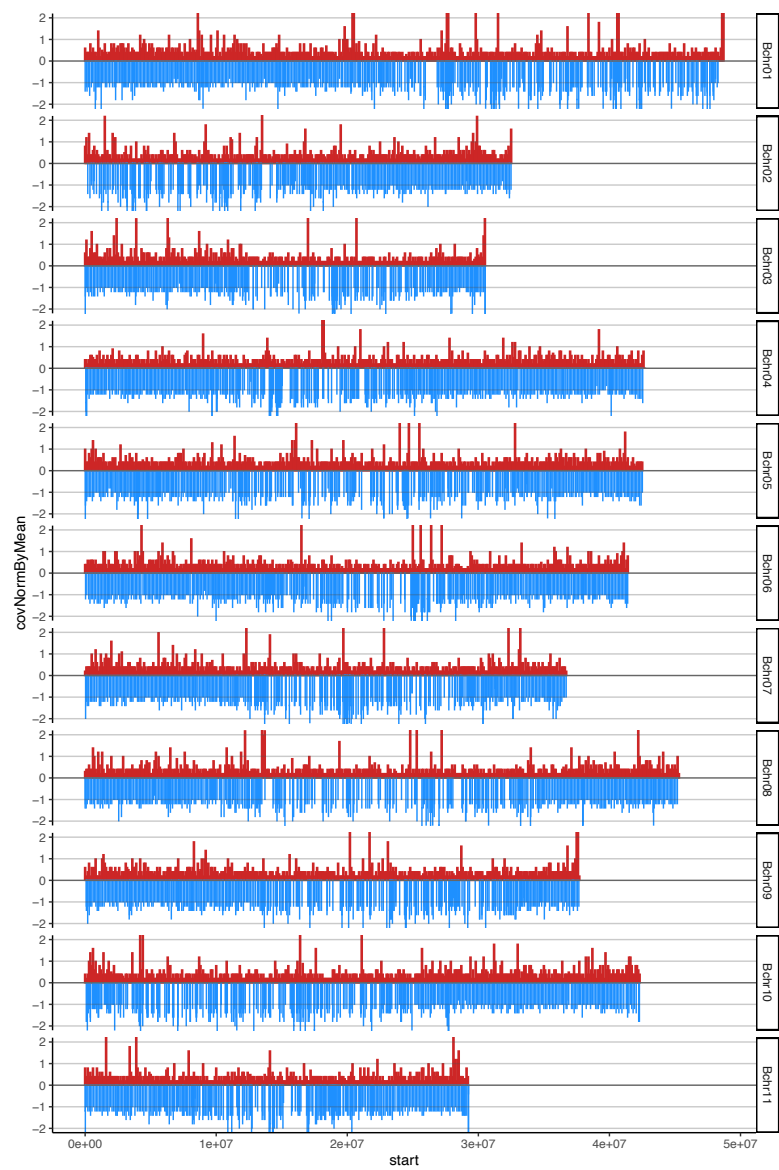

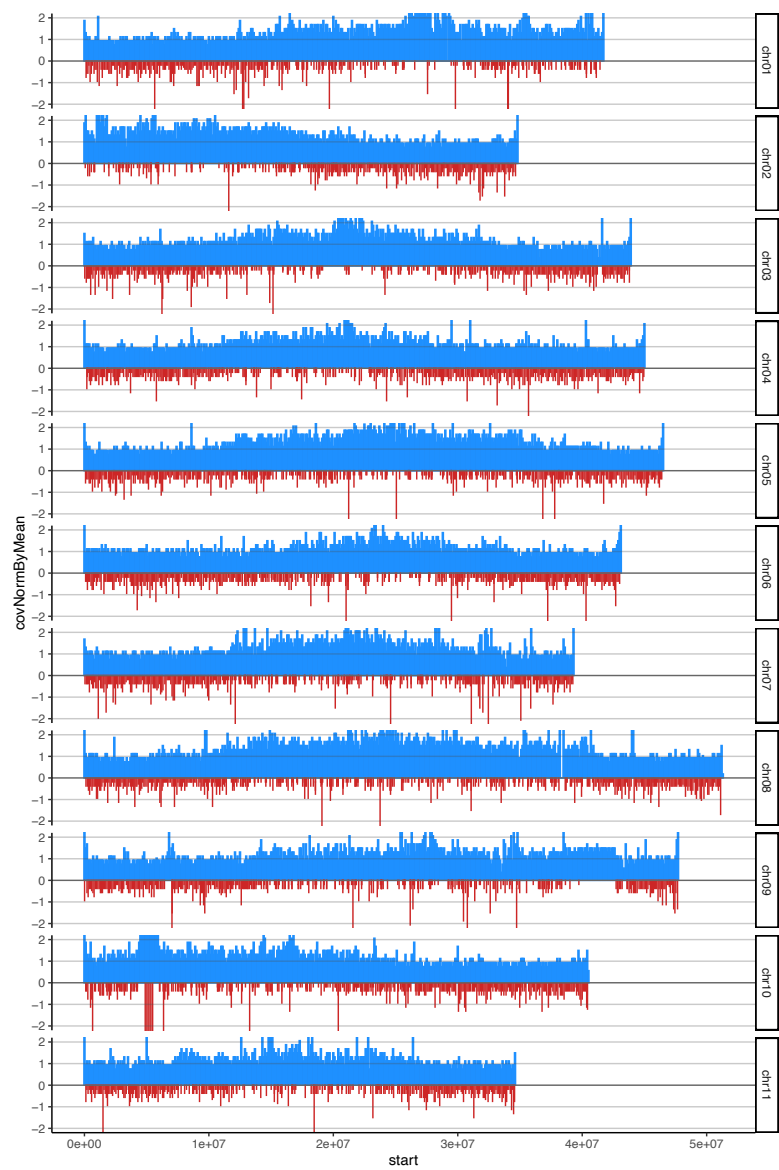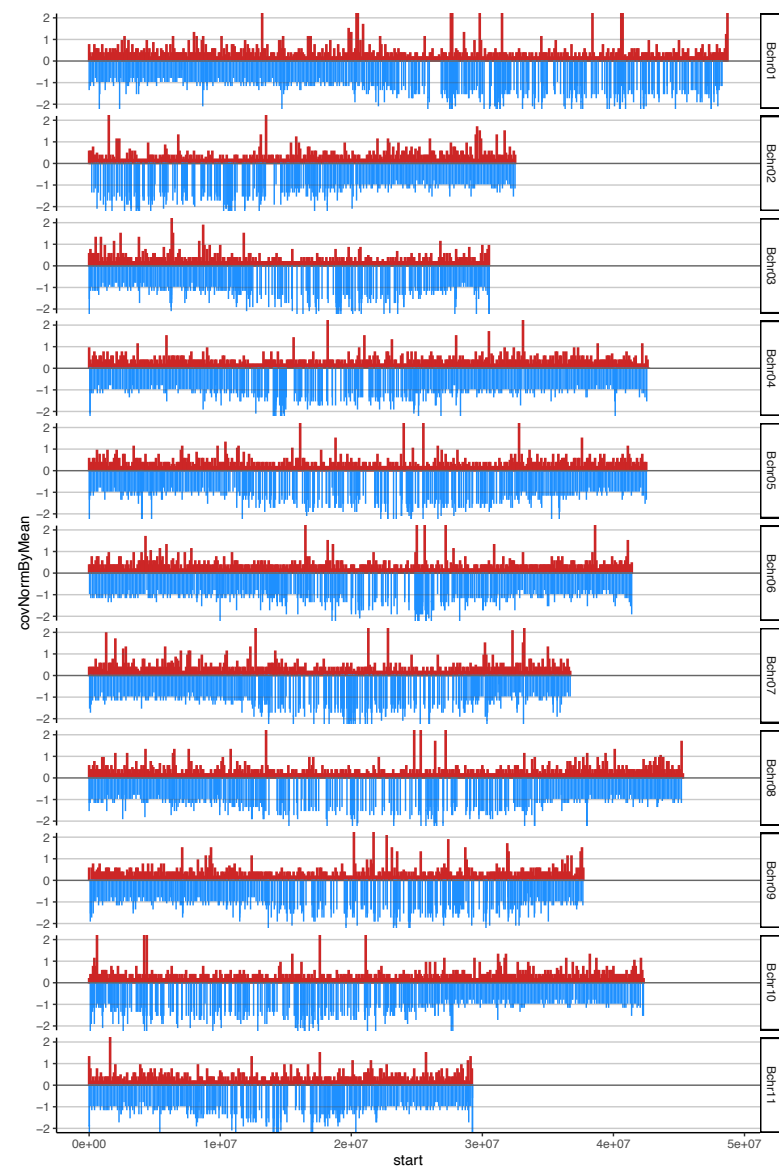

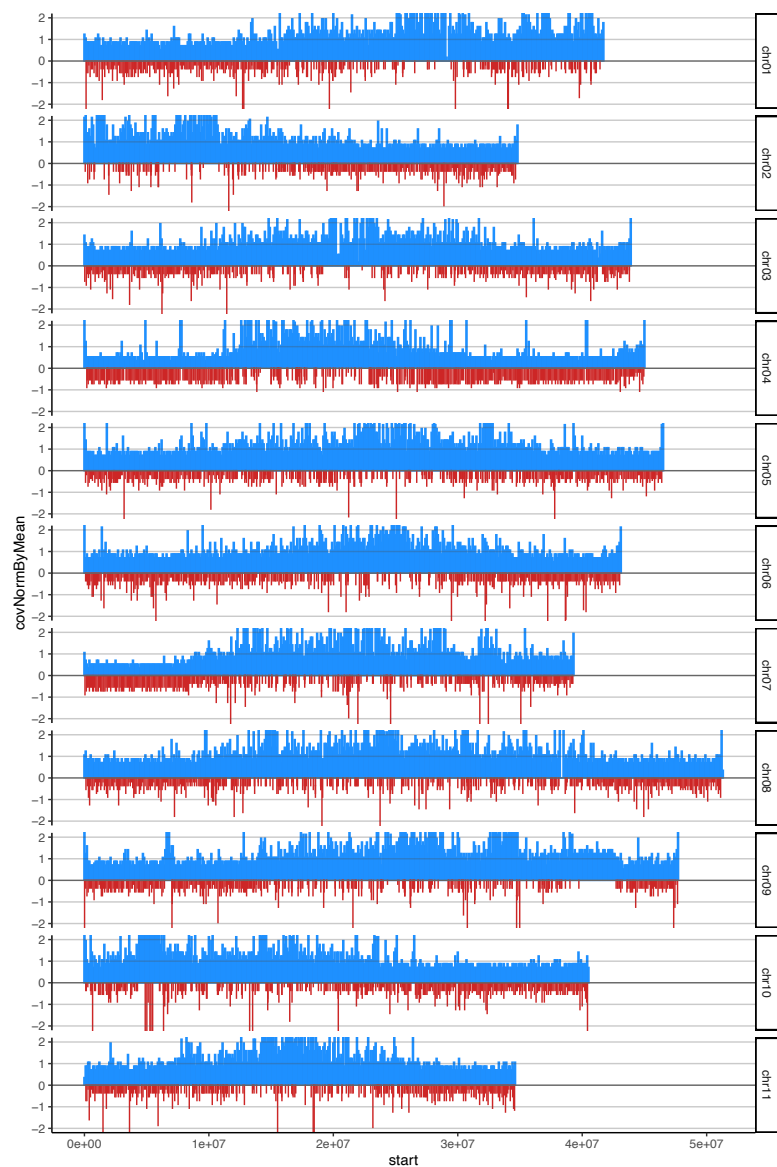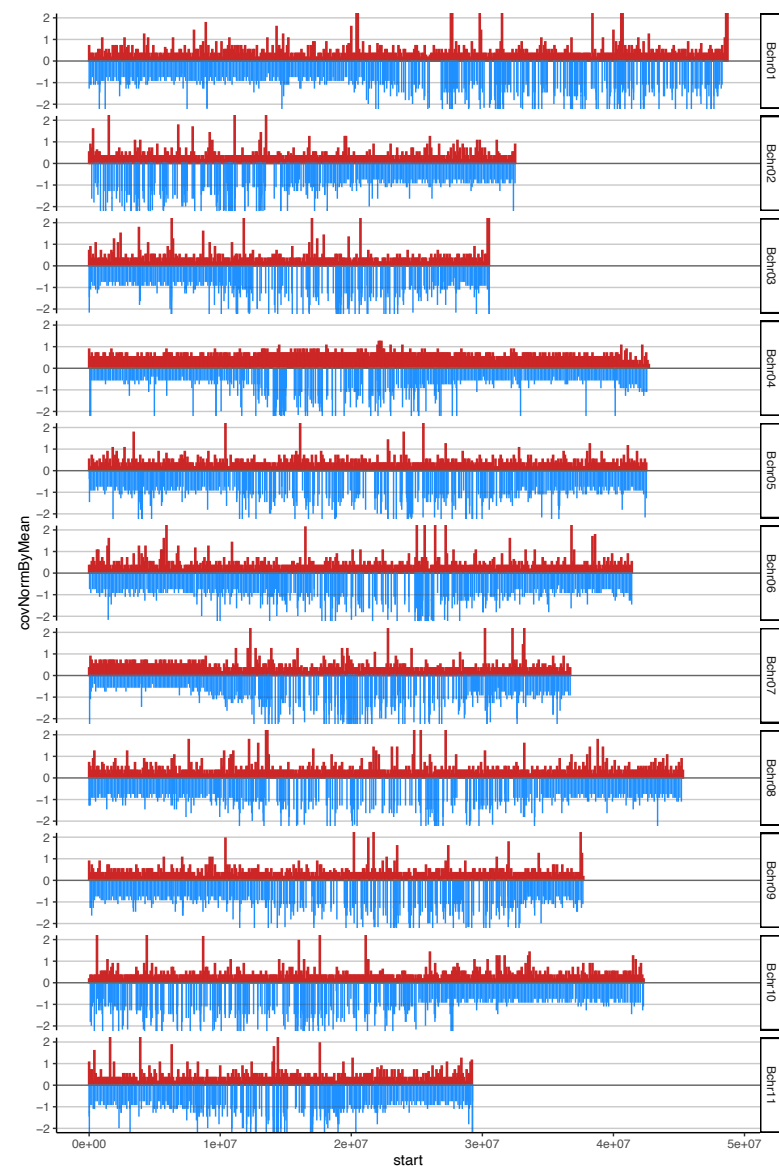

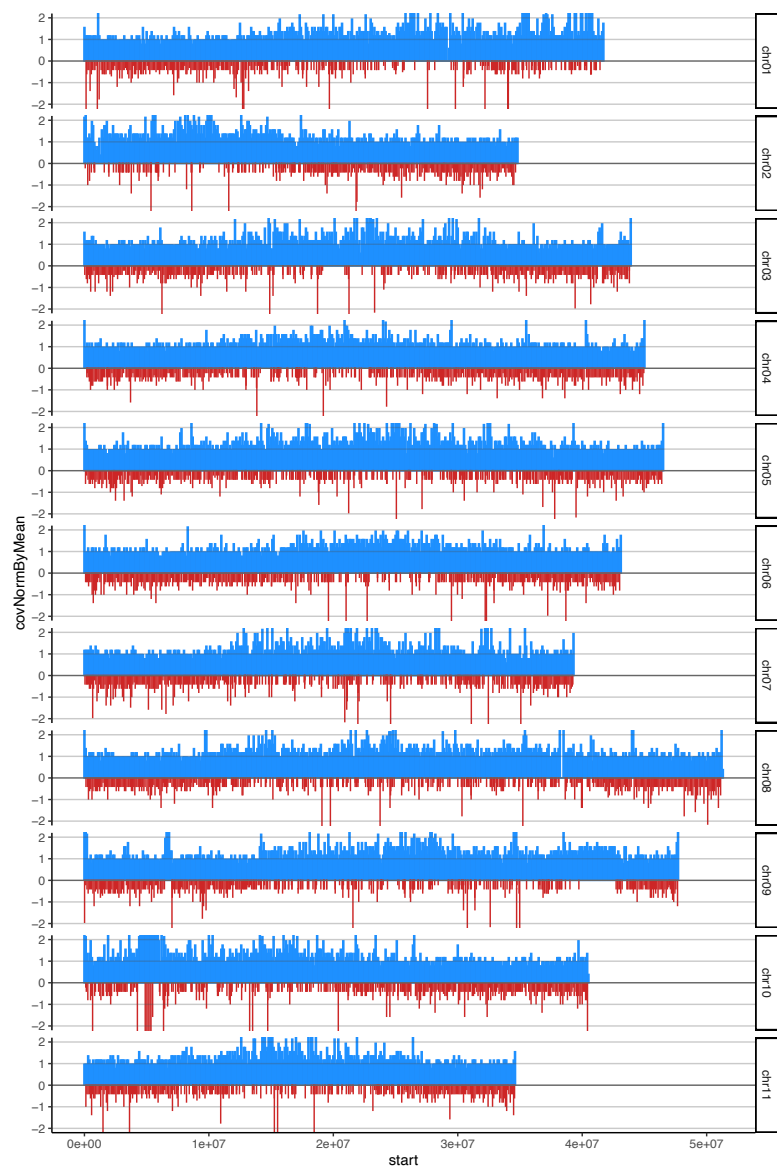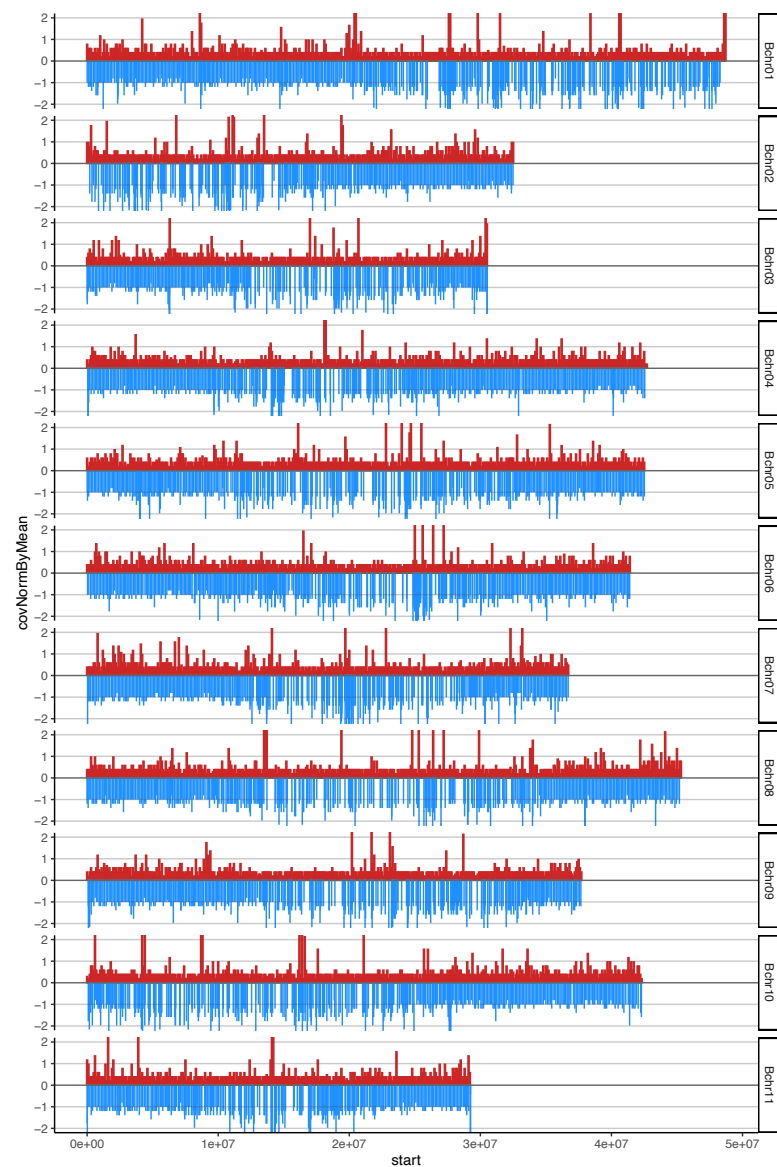

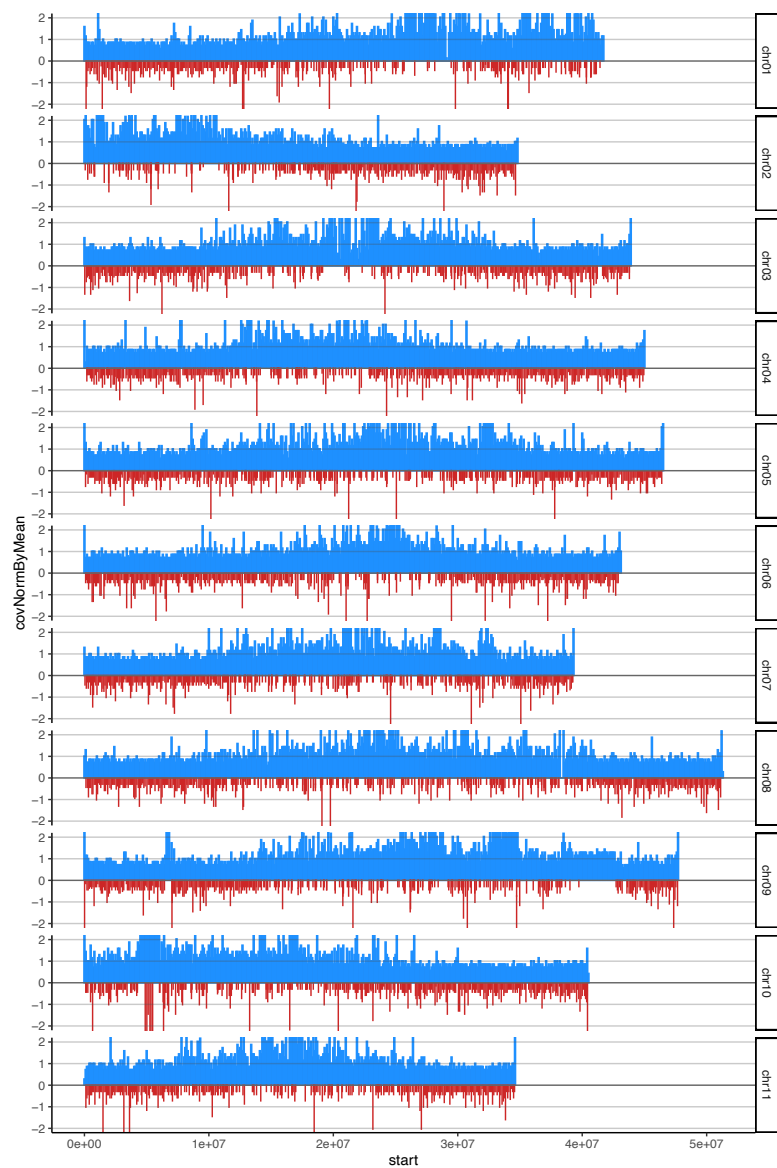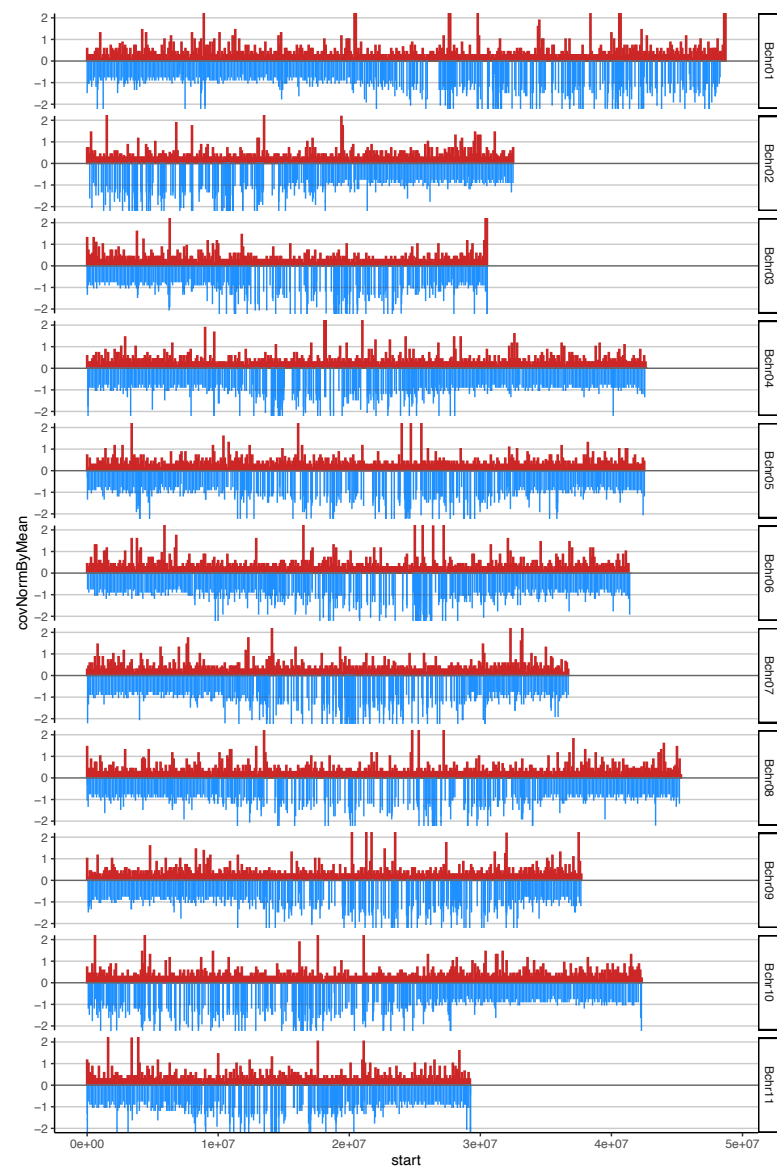

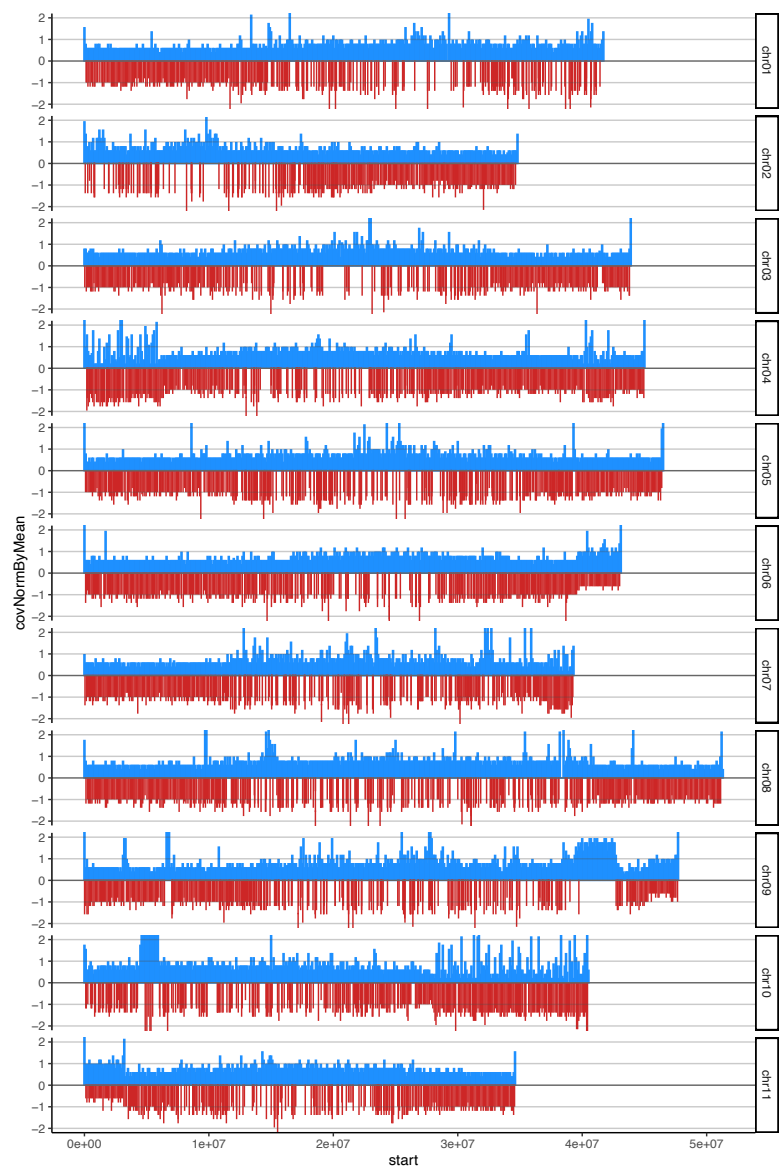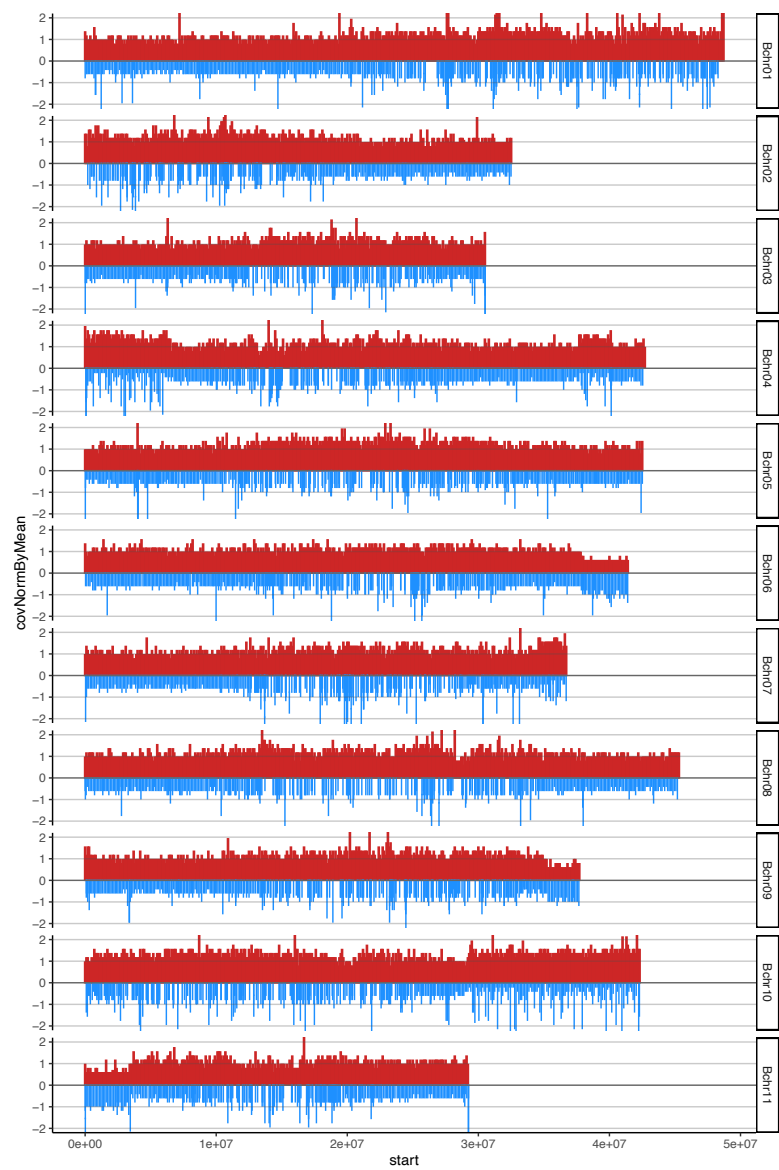

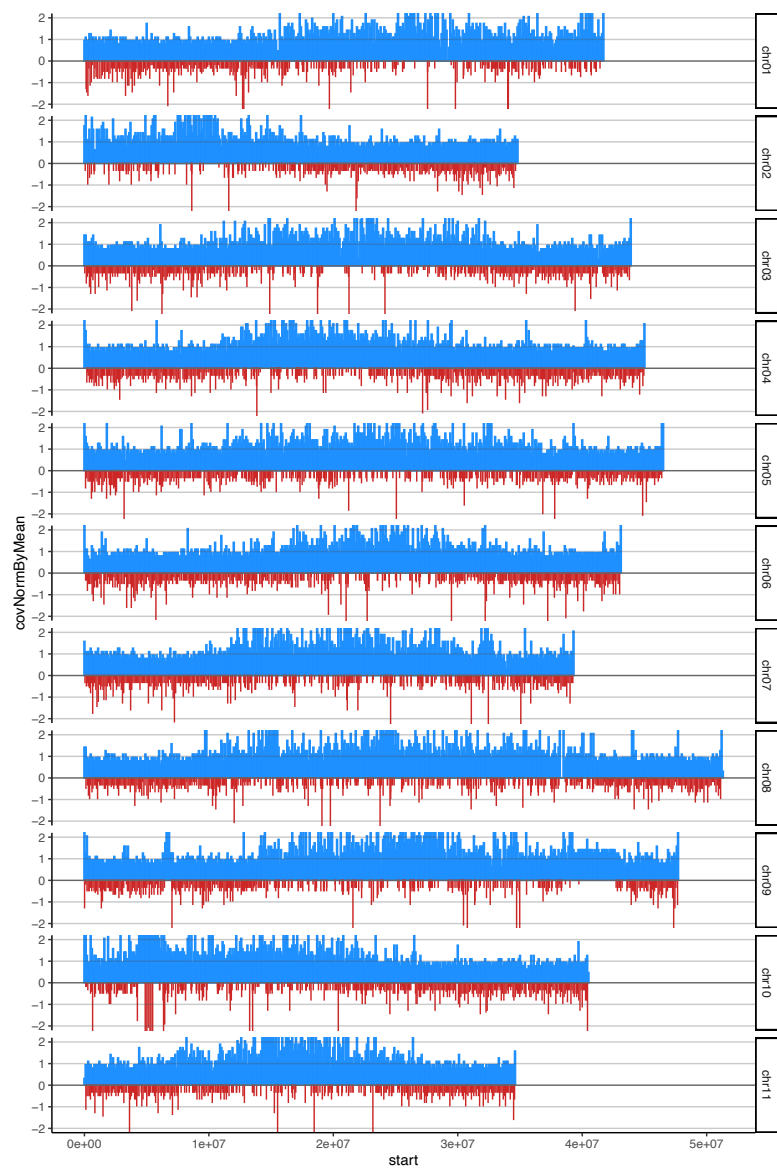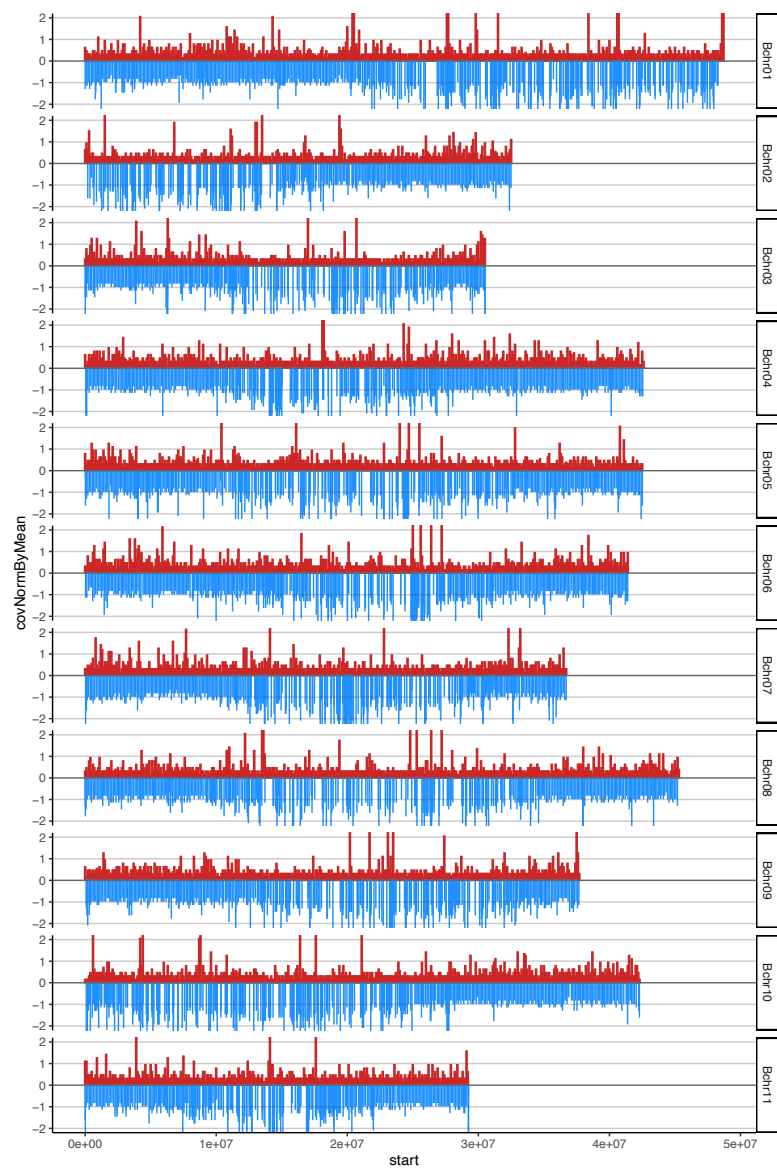

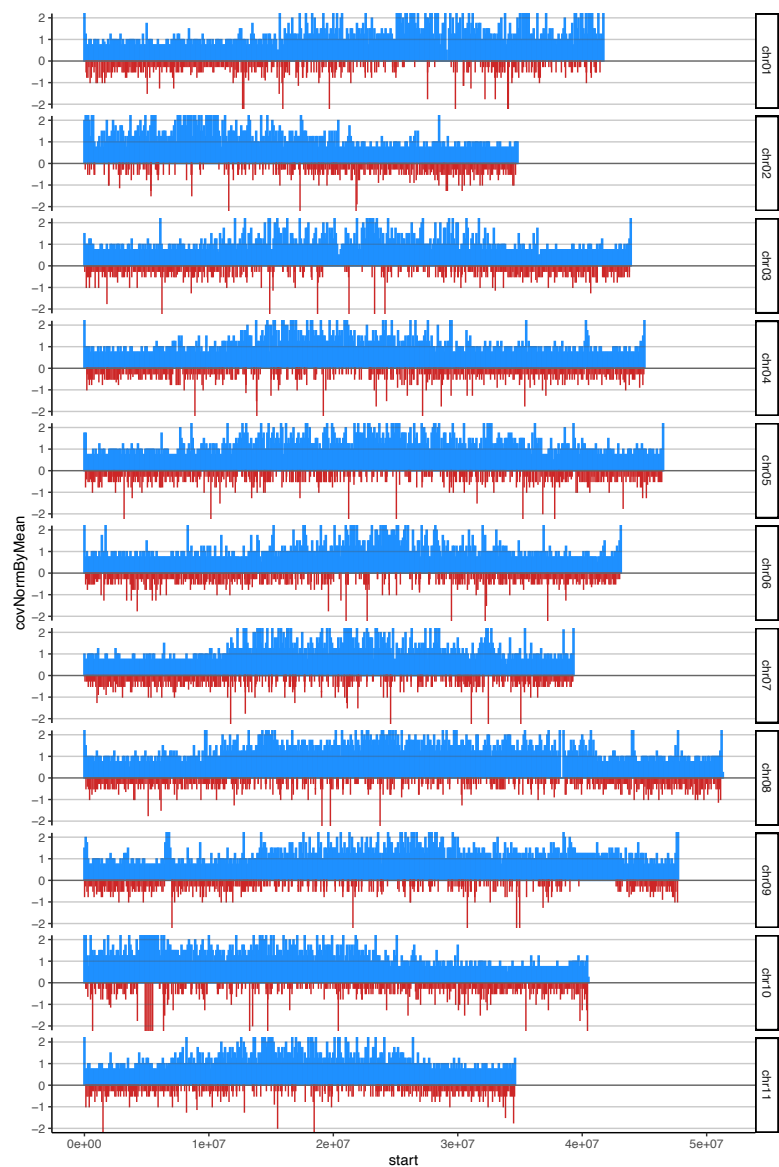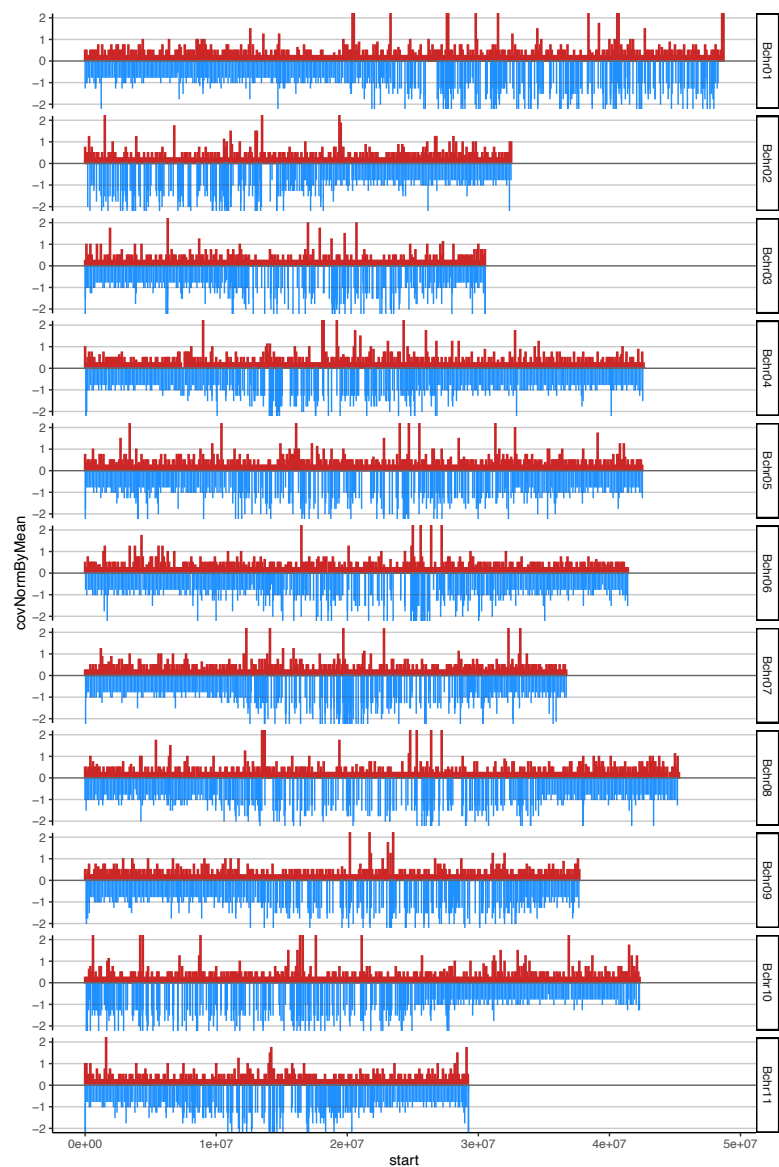

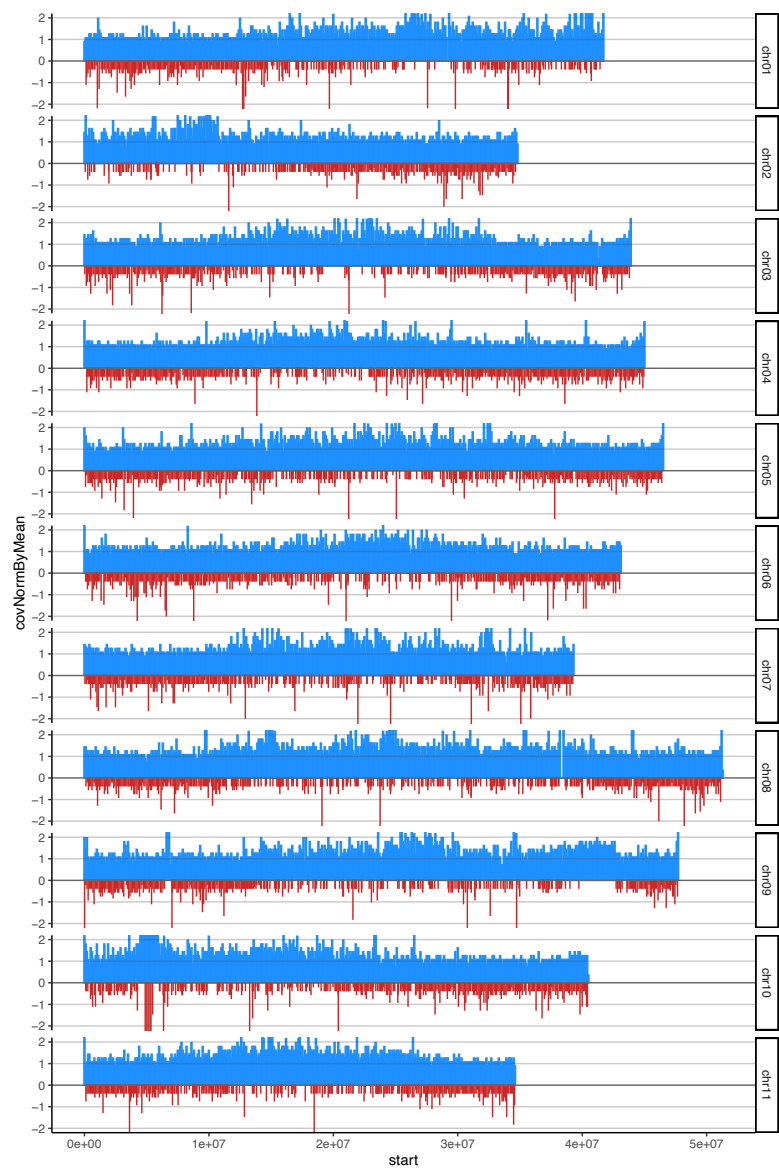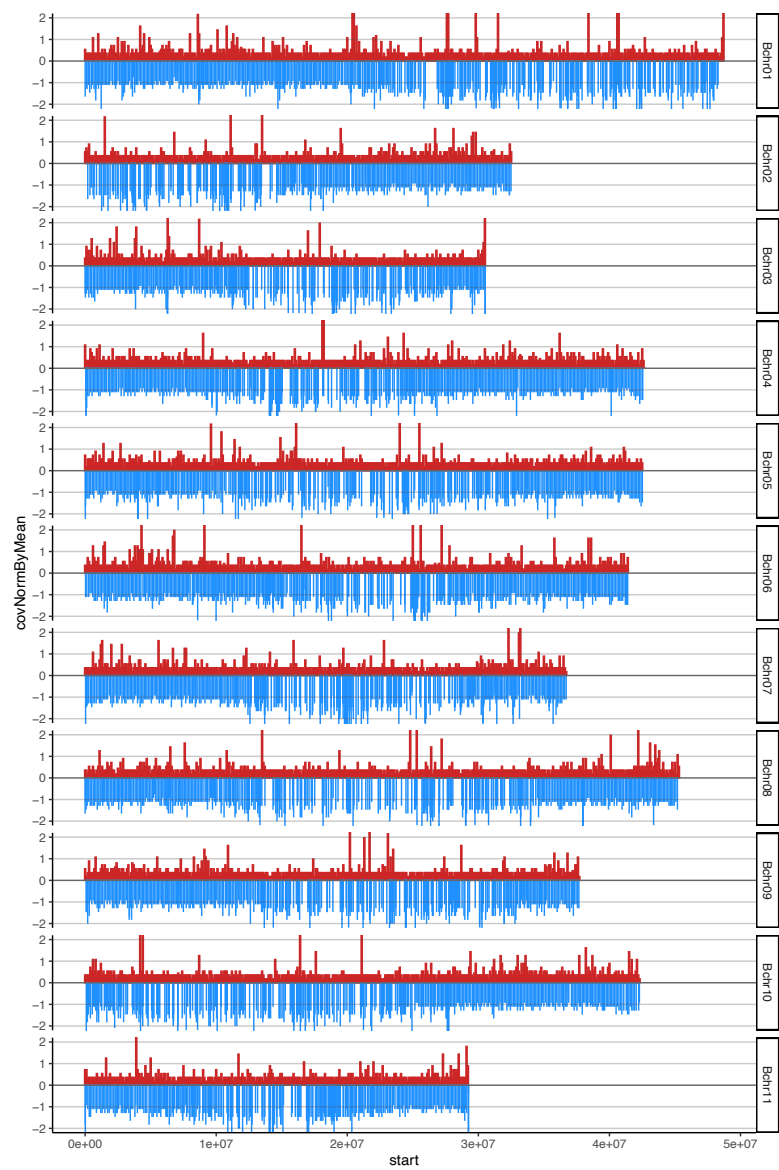

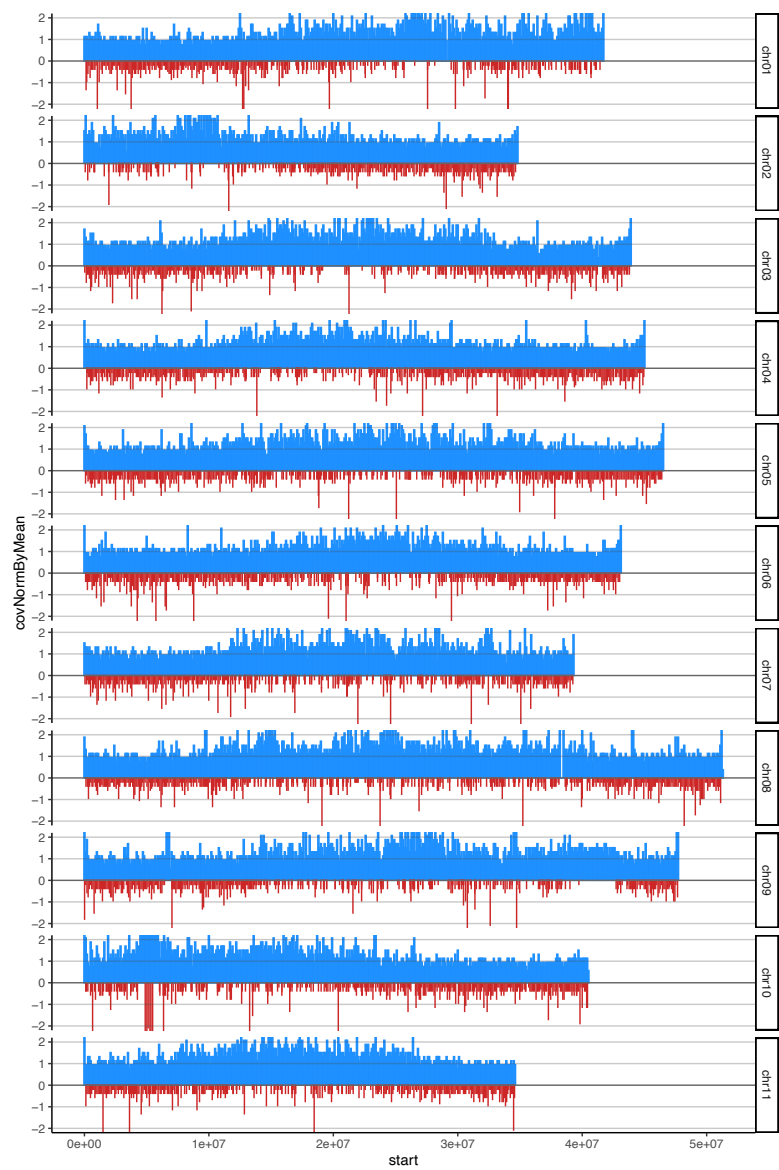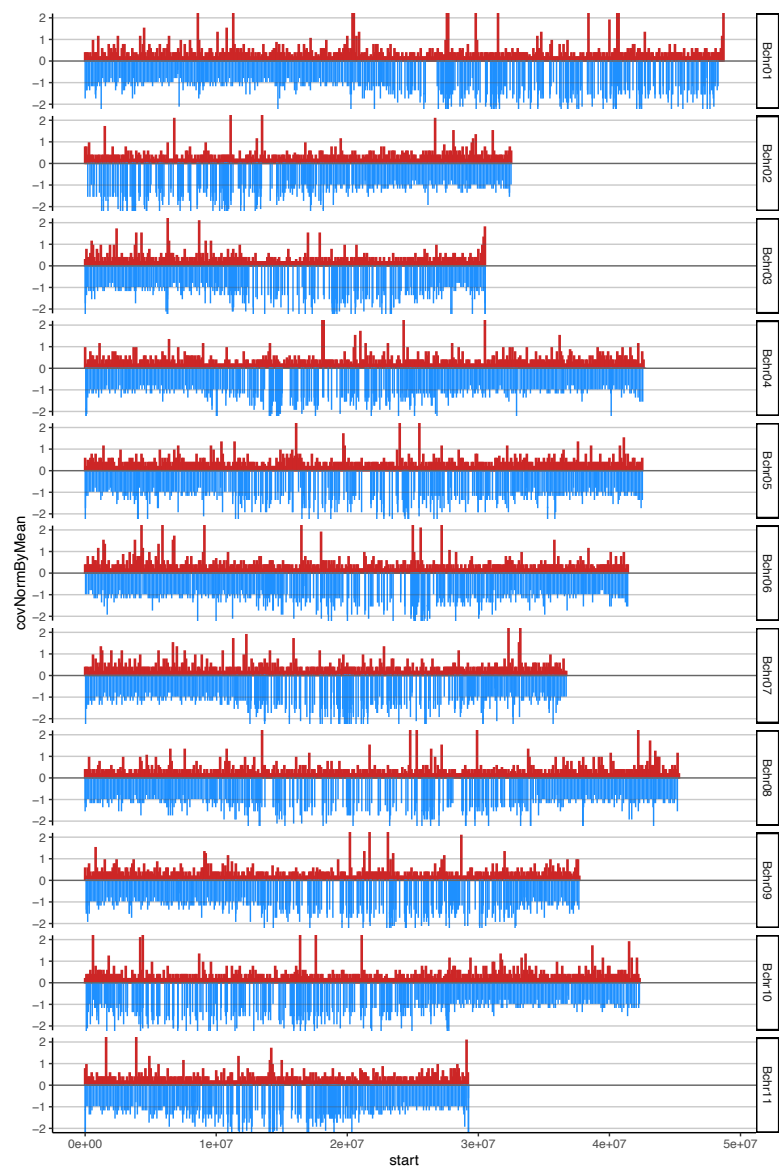

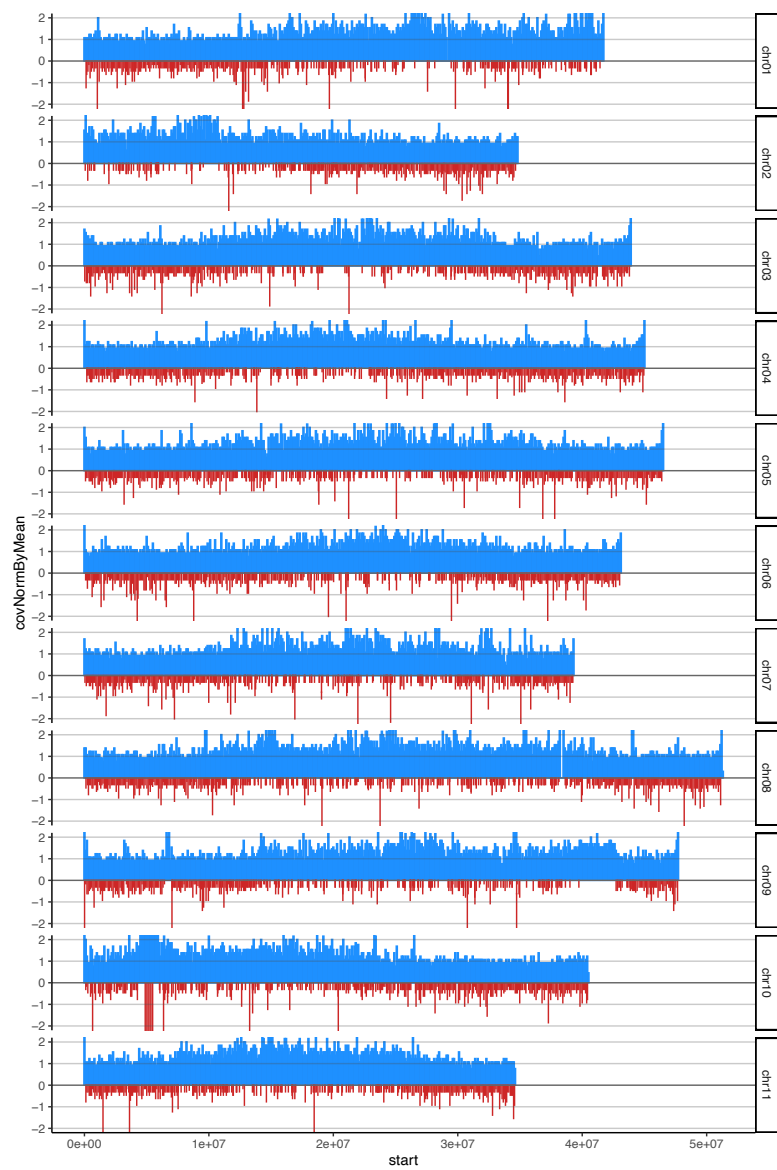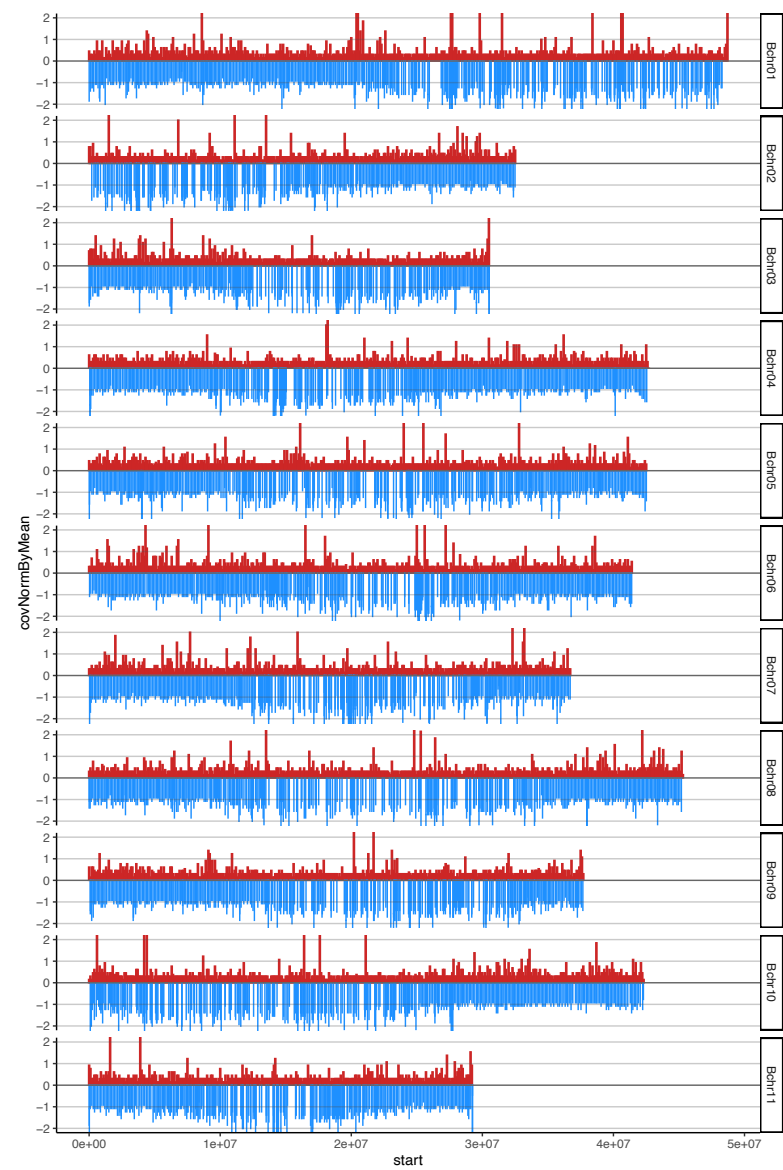

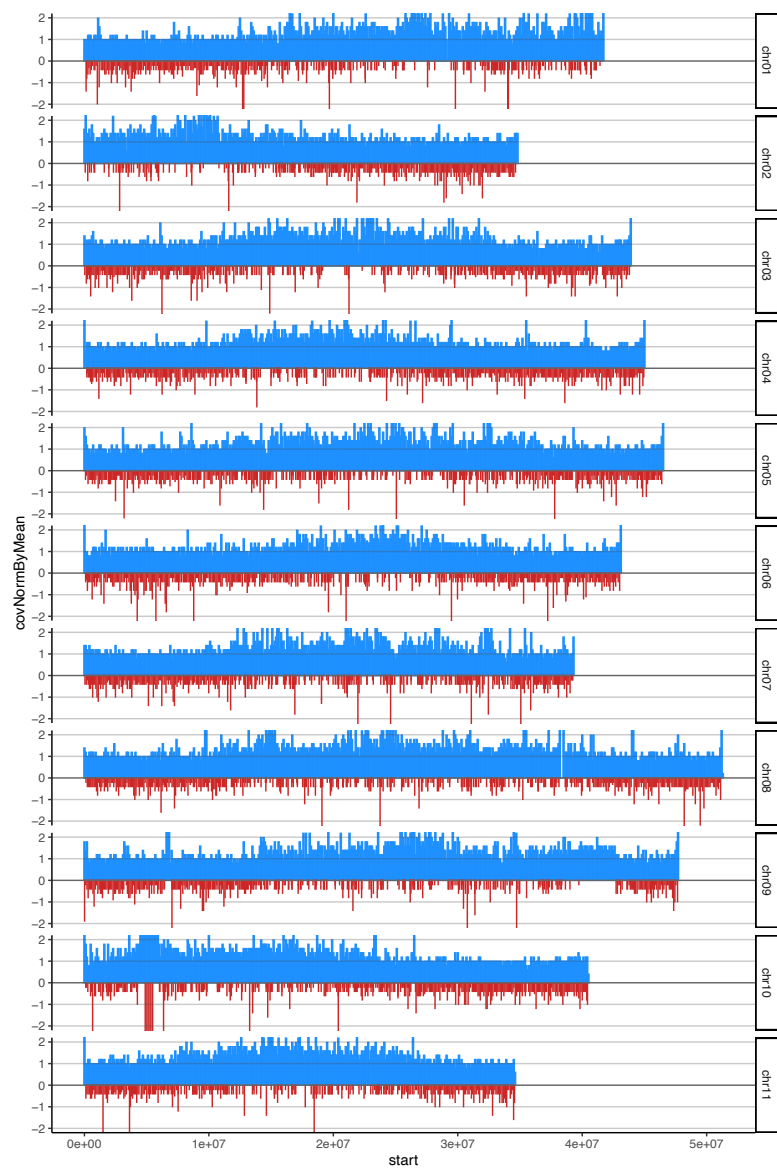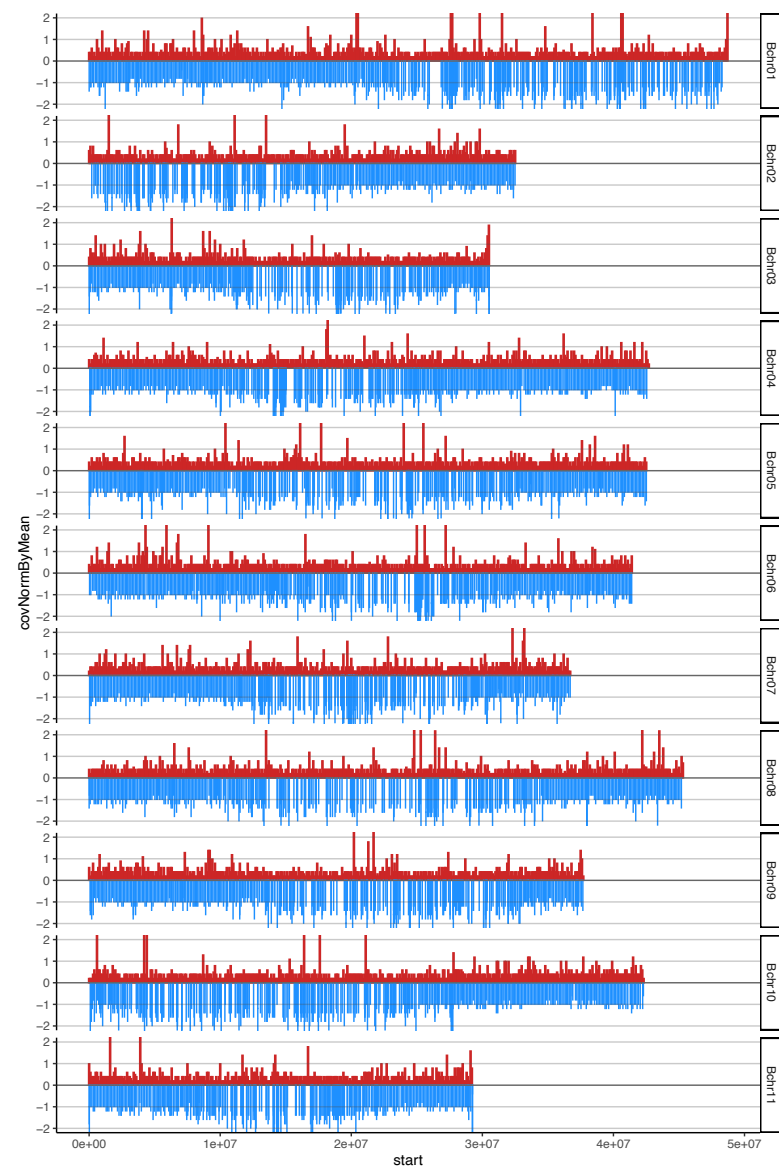

Supplement: mcad192_suppl_Supplementary_File_S5 [file mcad192_suppl_supplementary_file_s5.pdf]

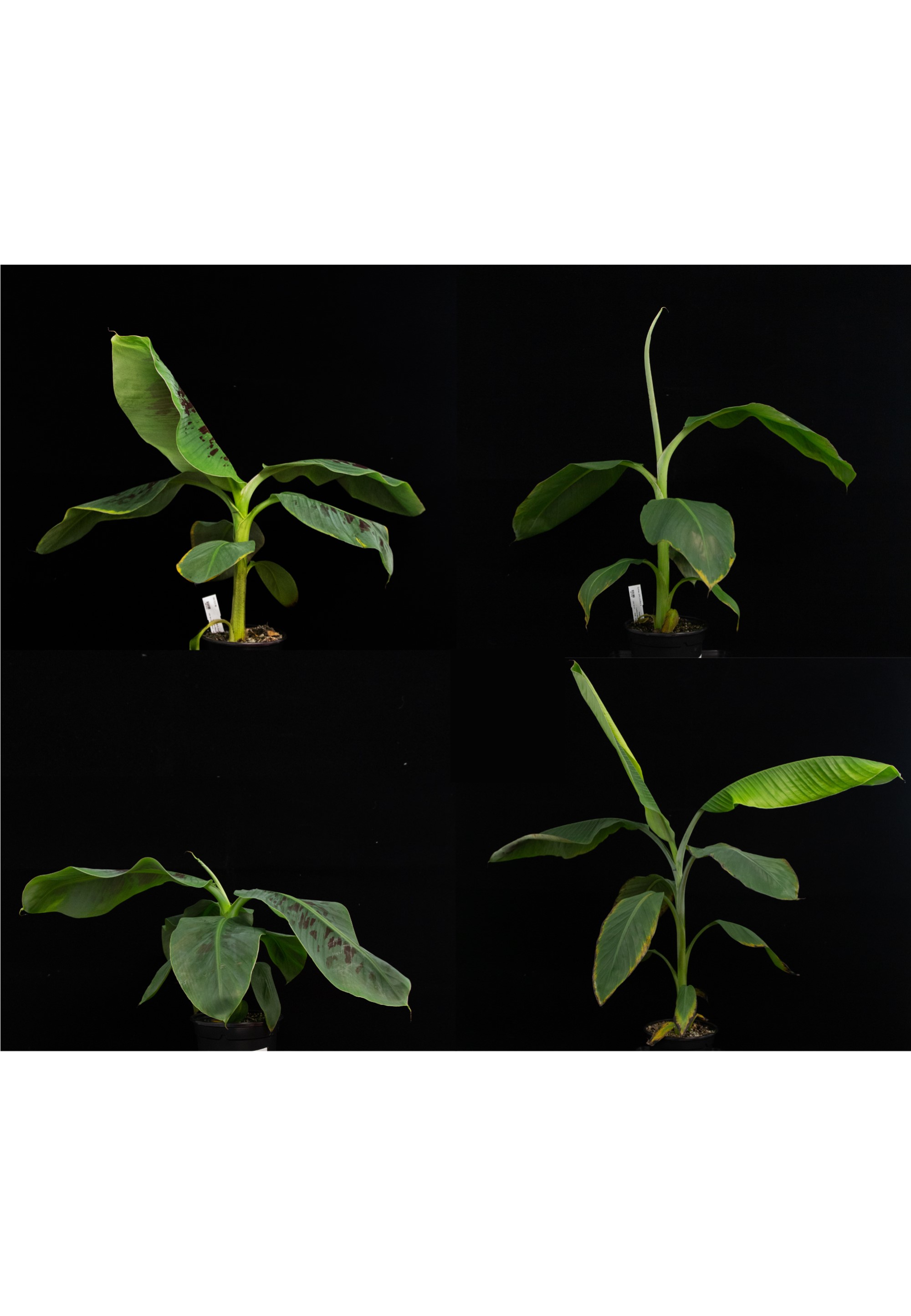

Supplement: mcad192_suppl_Supplementary_Data_S2 [file mcad192_suppl_supplementary_data_s2.jpeg]
