# Supplementary material for: Characterizing subgenome recombination and chromosomal imbalances in banana varietal lineages
Source: Ann Bot. 2023 Dec 14;133(2):349–64. doi: 10.1093/aob/mcad192 (PMC11005773; doi:10.1093/aob/mcad192)
Supplement: mcad192_suppl_Supplementary_File_S2 [file mcad192_suppl_supplementary_file_s2.pdf]

Additional file 2; Characterising subgenome recombination and chromosomal imbalances in banana varietal lineages.

Janet Higgins, Jaime Andrés Osorio-Guarín, Carolina Olave-Achury, Deisy Lisseth Toloza-Moreno, Ayda Enriquez, Federica Di Palma, Roxana Yockteng, Jose De Vega

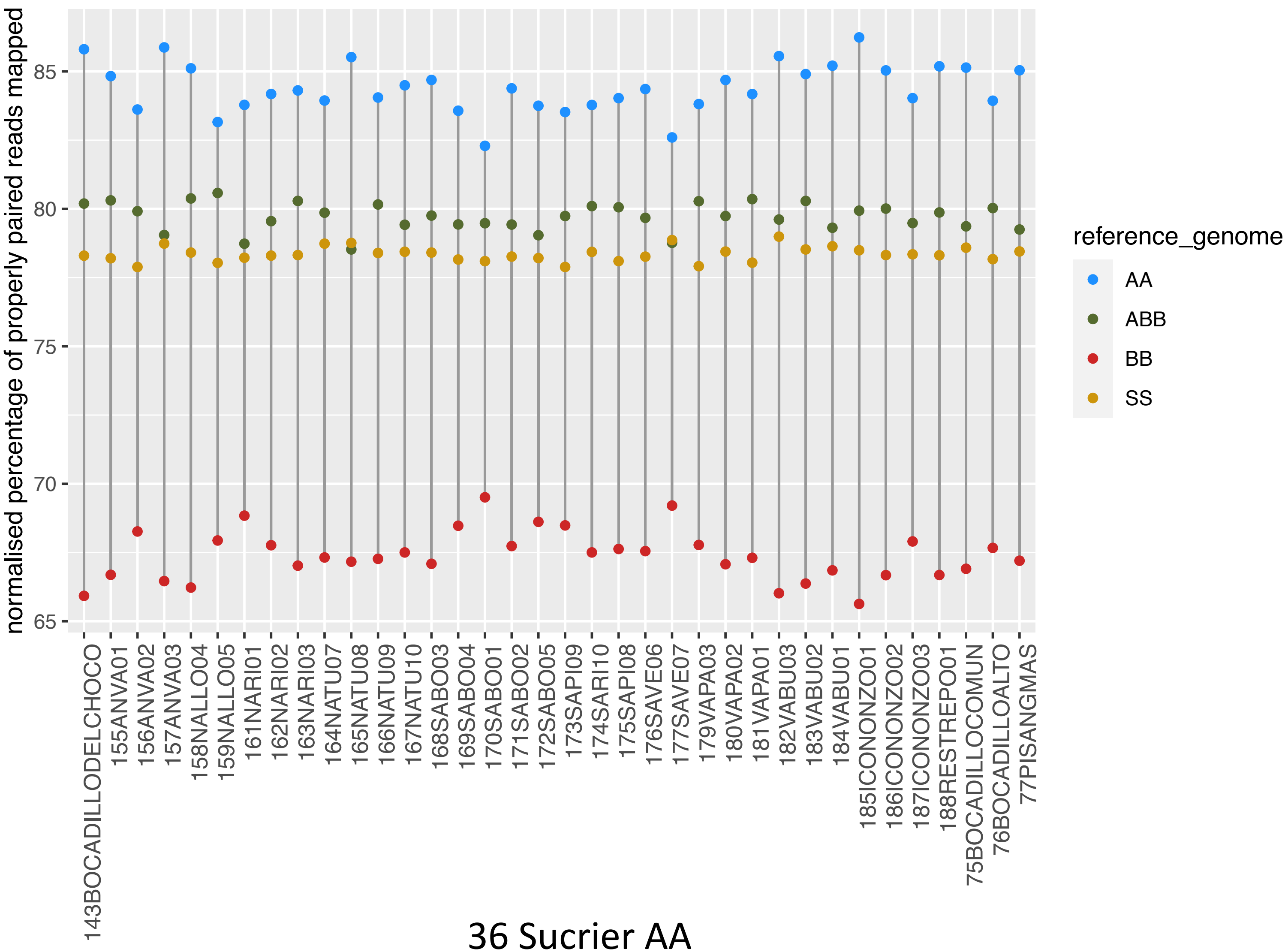

36 Sucrier AA

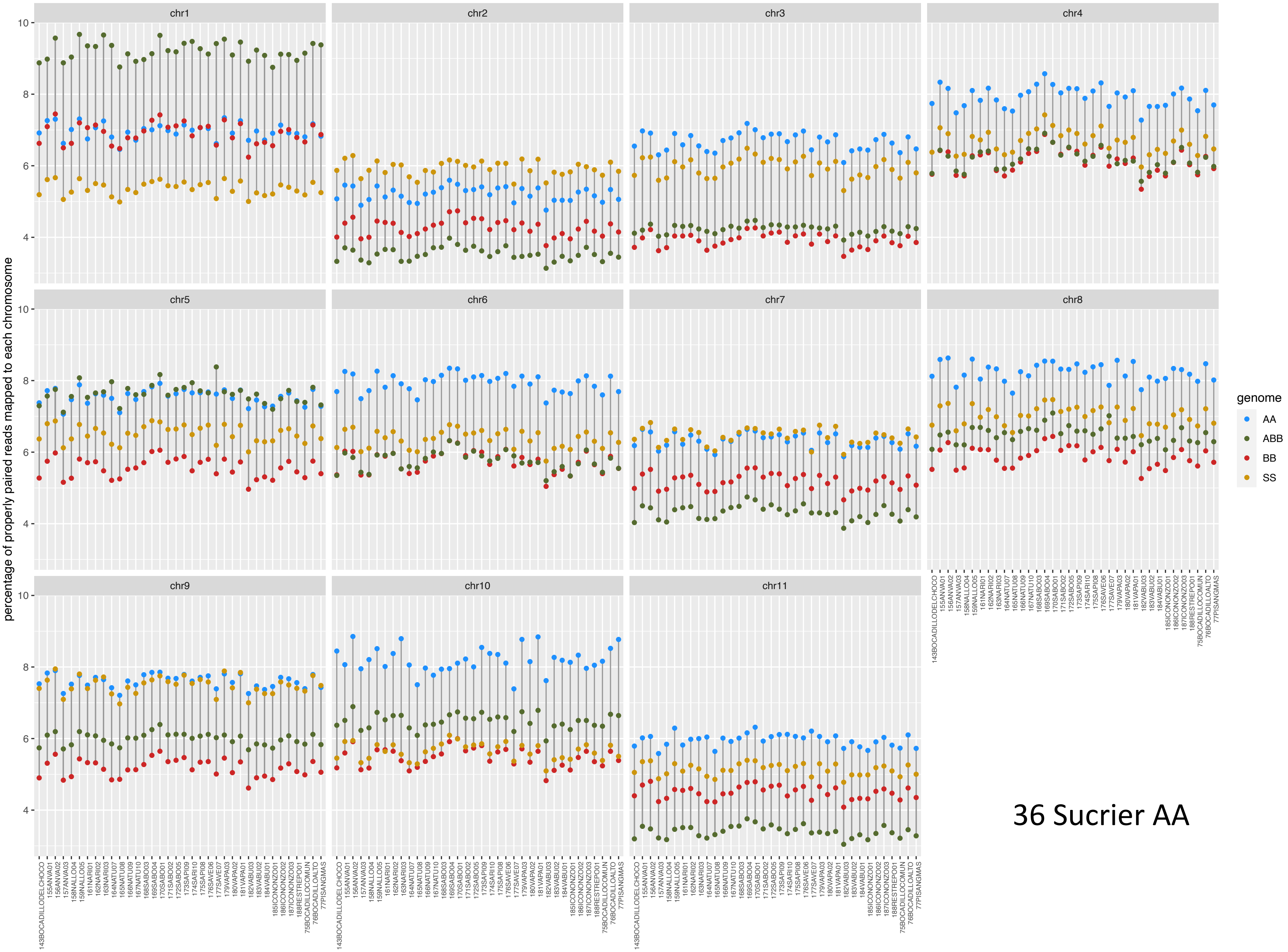

36 Sucrier AA

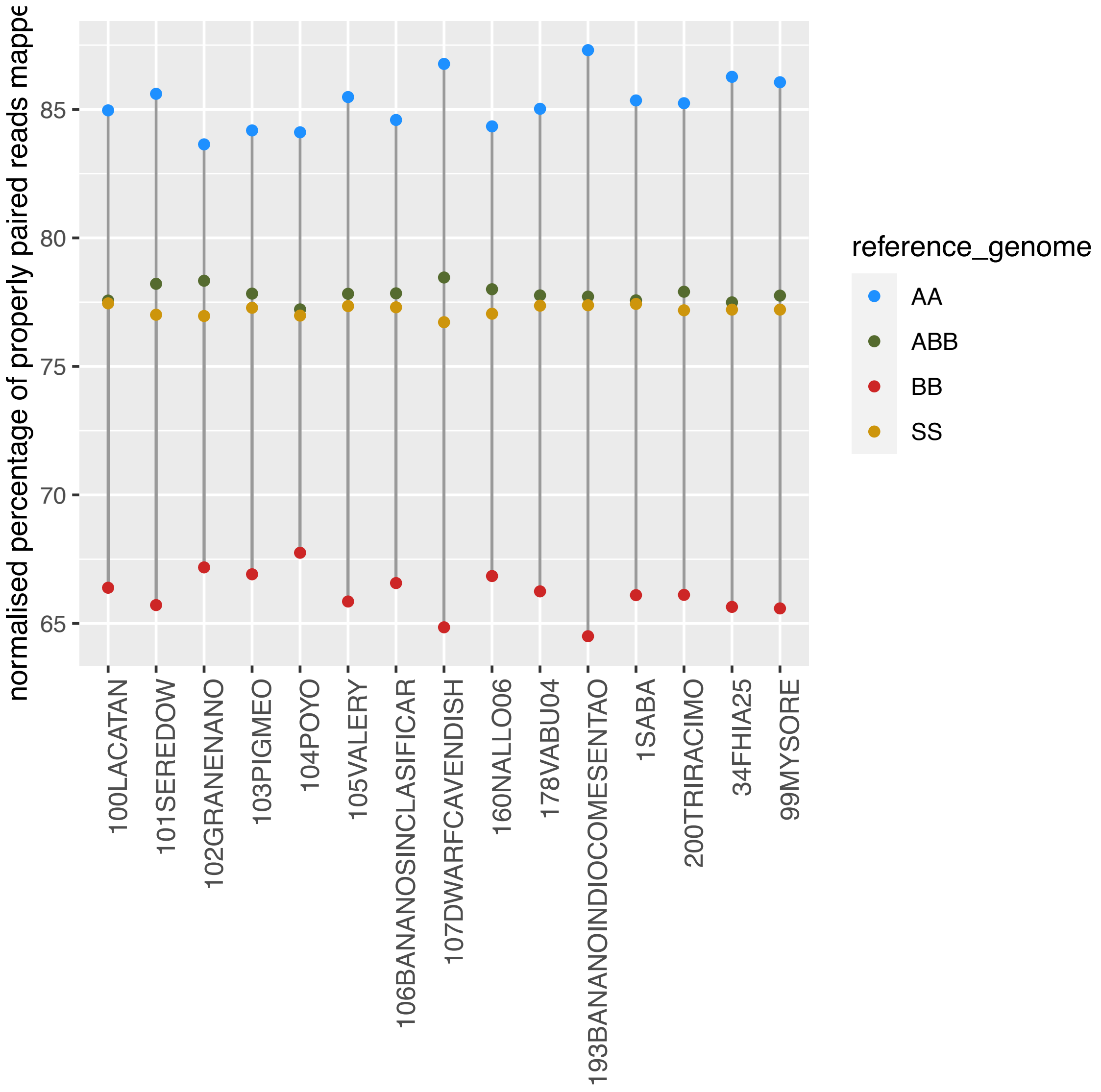

15 Cavendish

rietal lineages.
na Yockteng, Jose De Vega

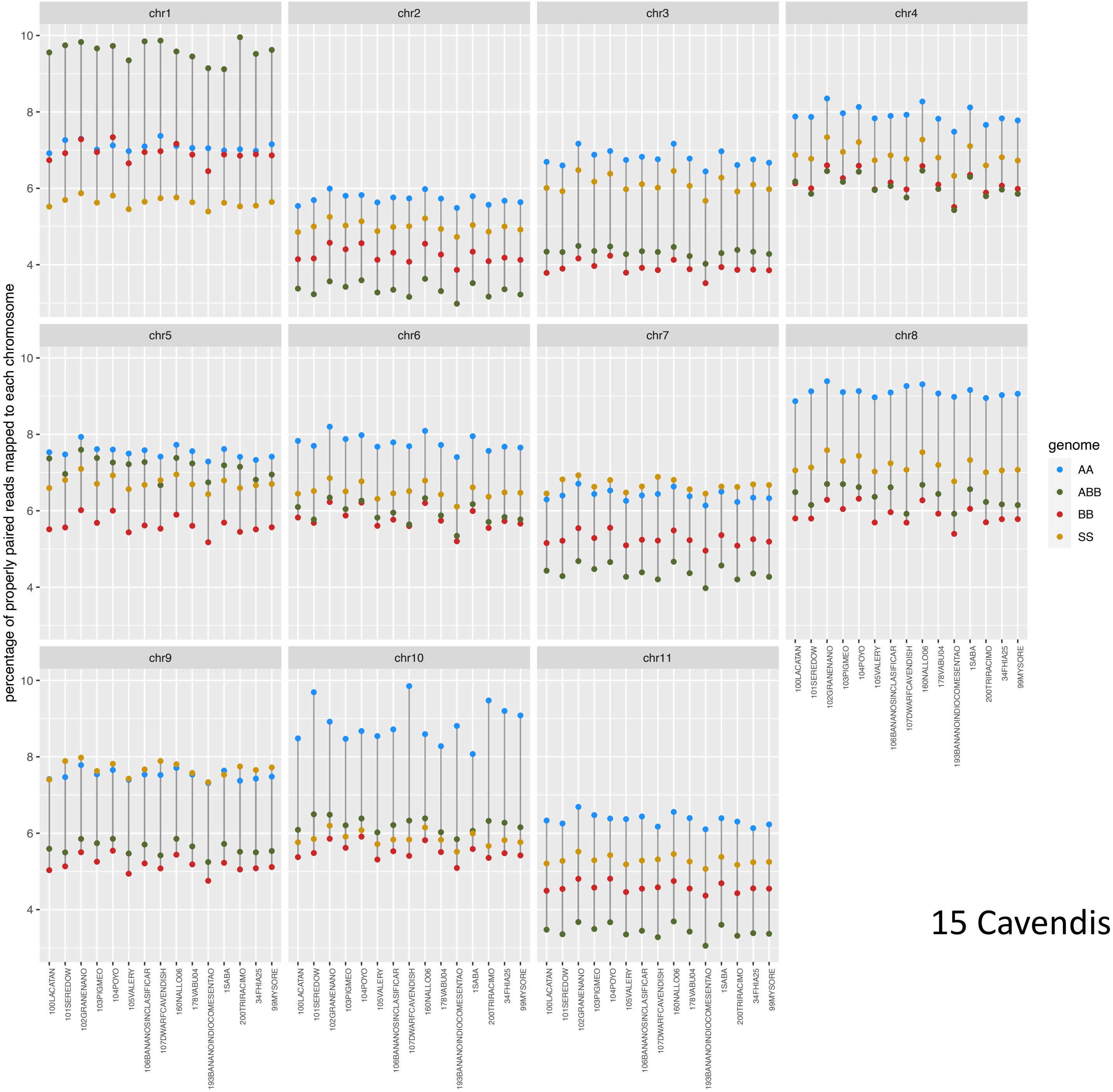

15 Cavendish AAA

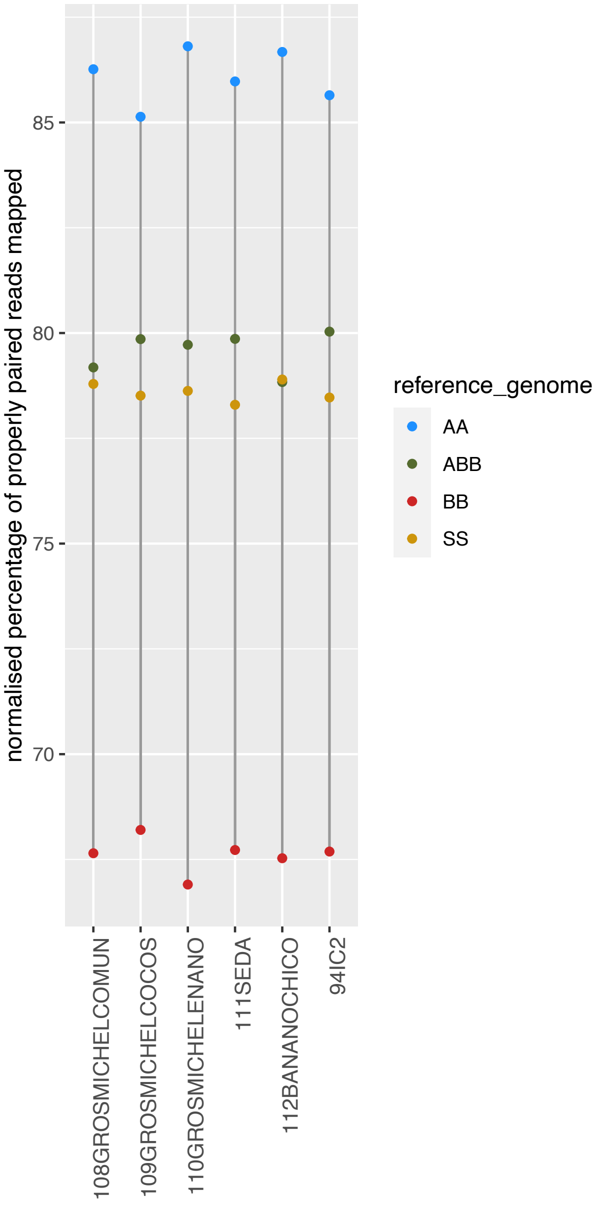

6 Gros Michel AAA

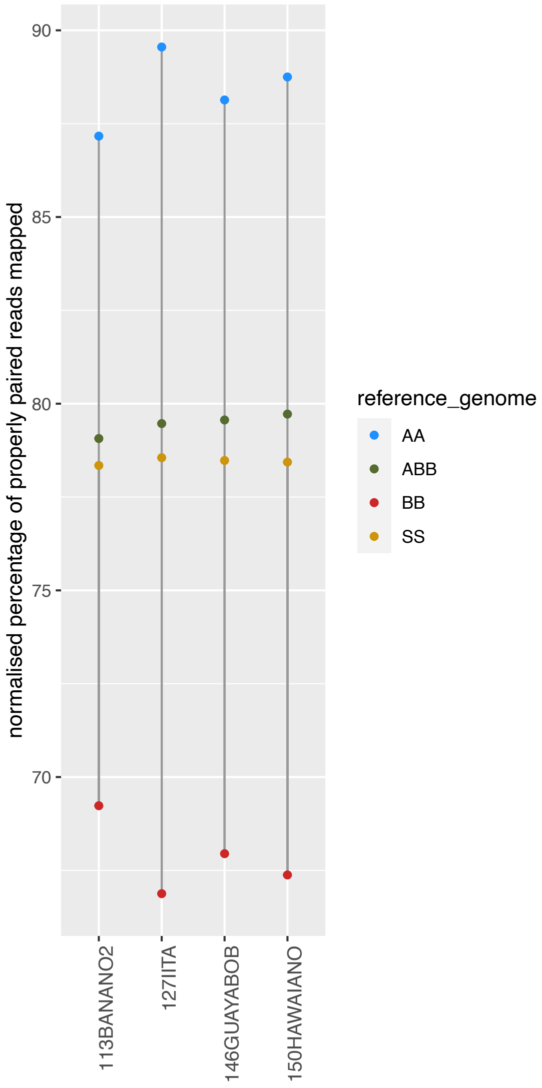

4 unknown AAA

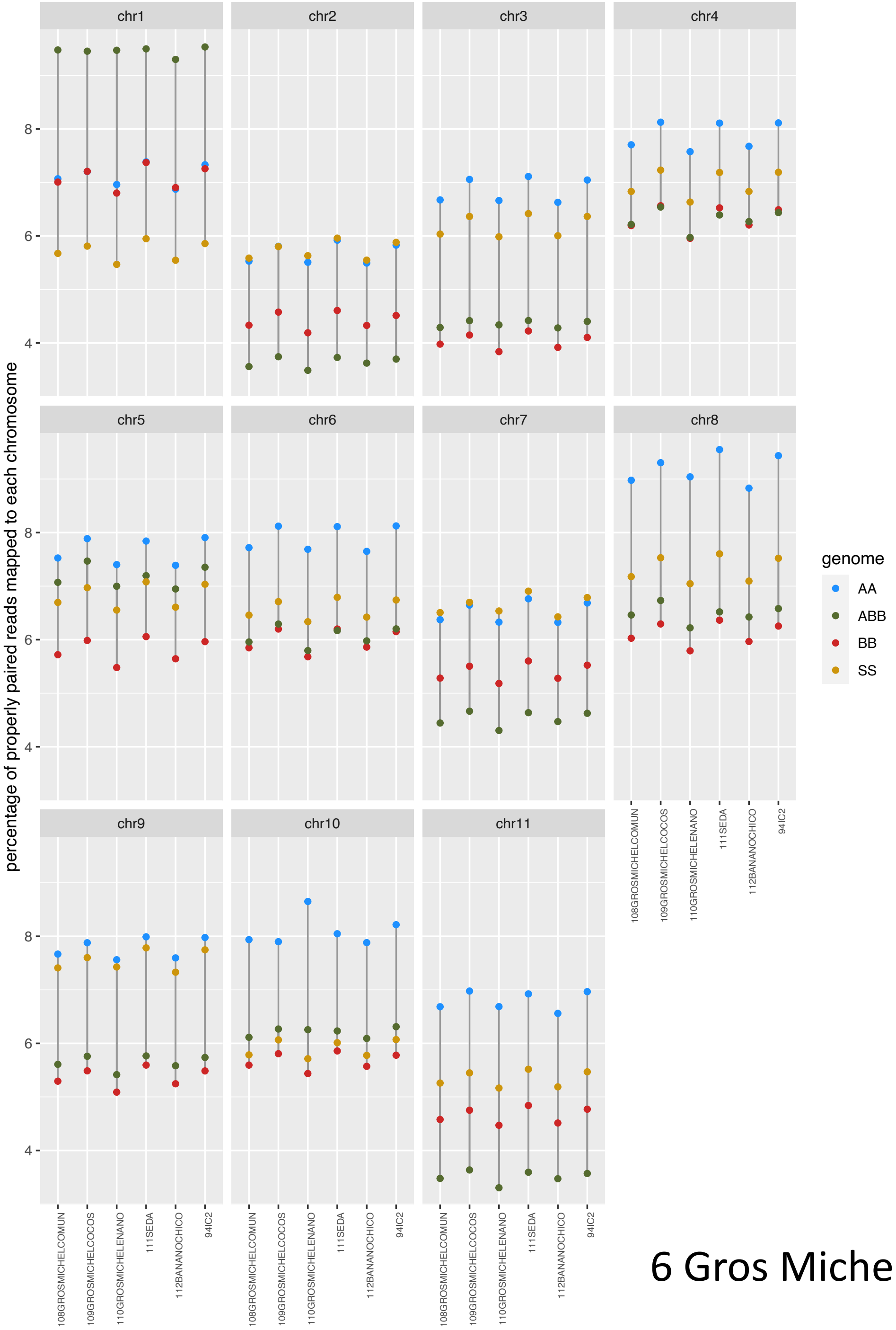

omosoma
oreno, Ayd

6 Gros Michel AAA

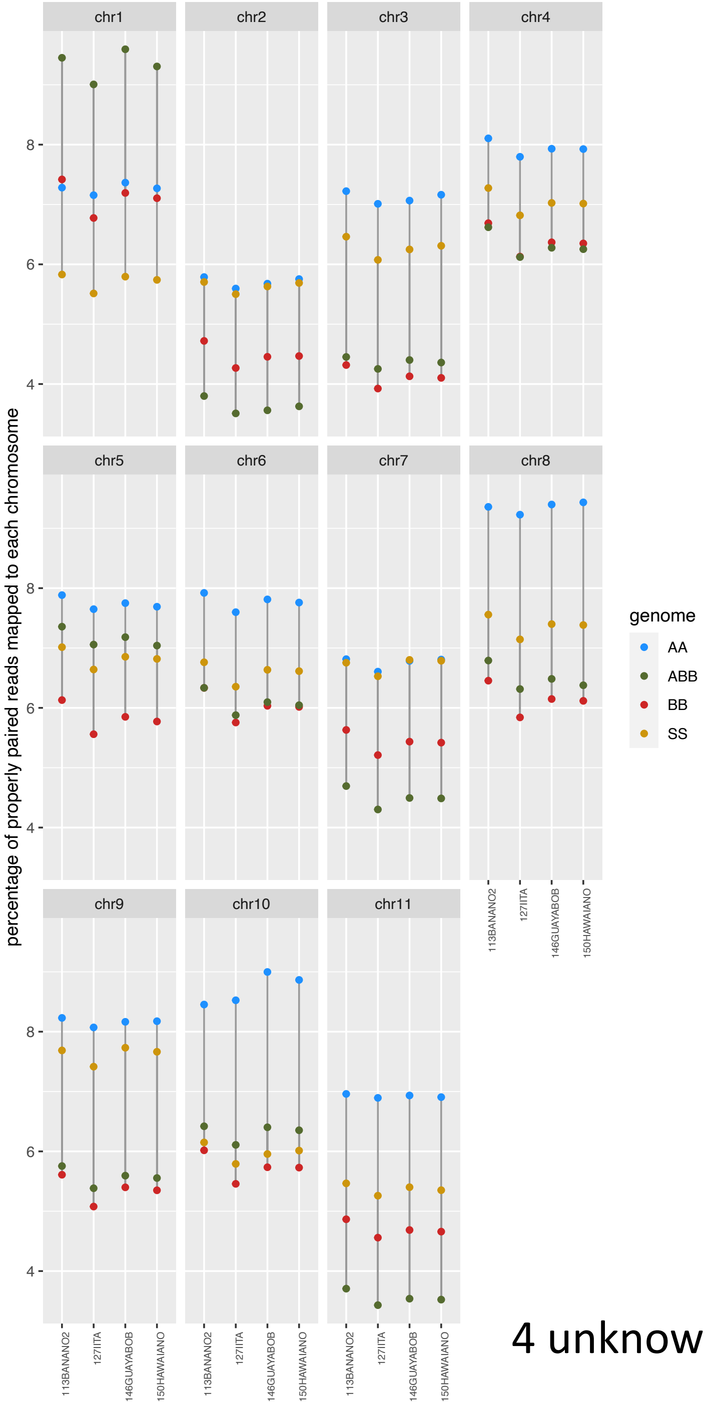

4 unknown AAA

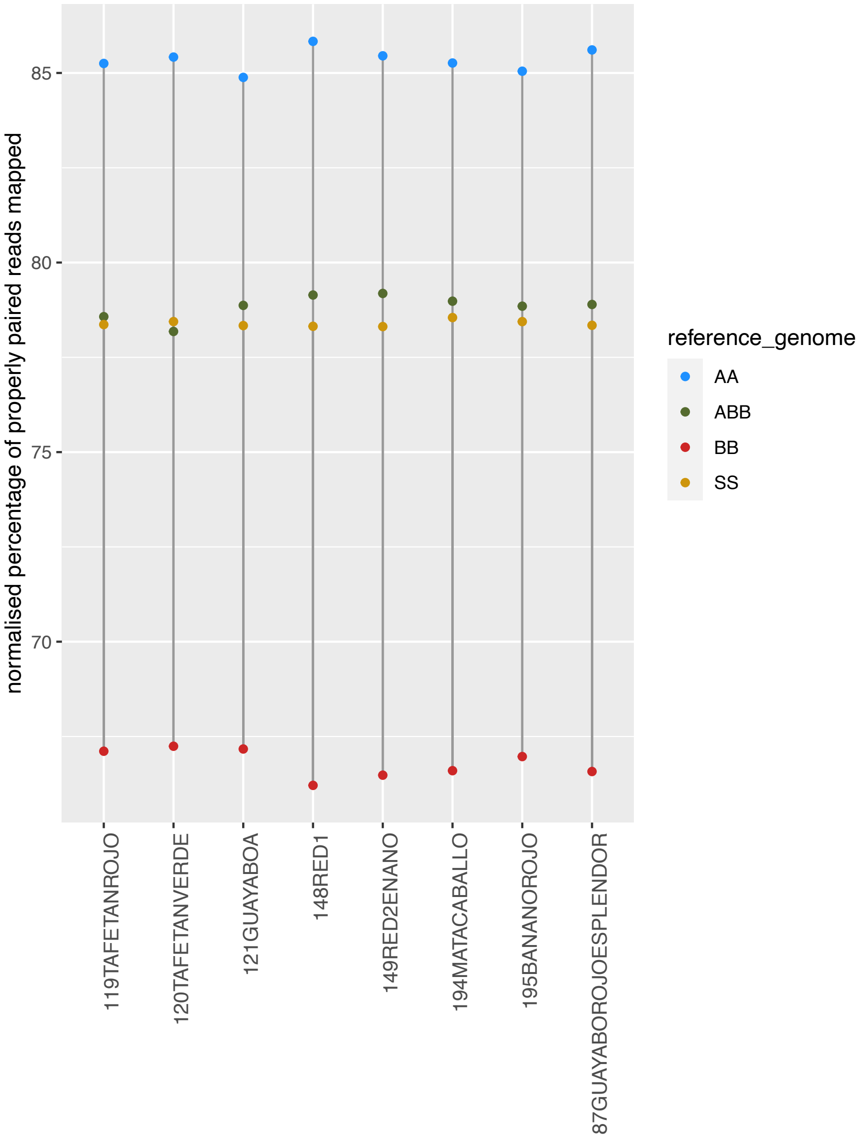

8 Red AAA

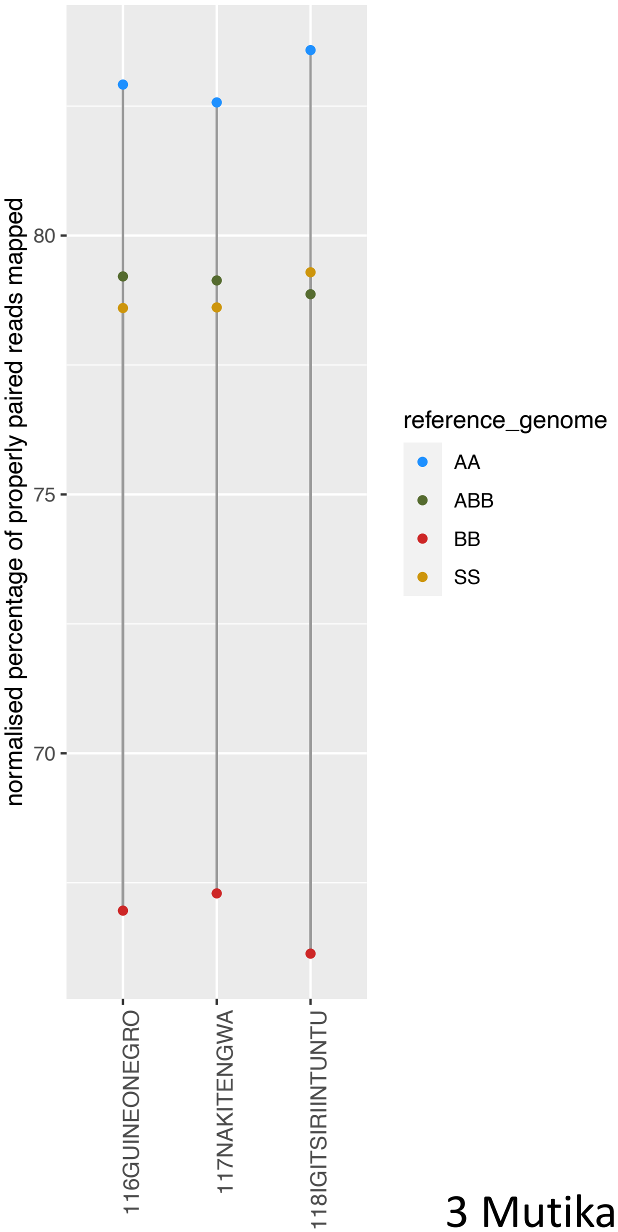

3 Mutika AAA

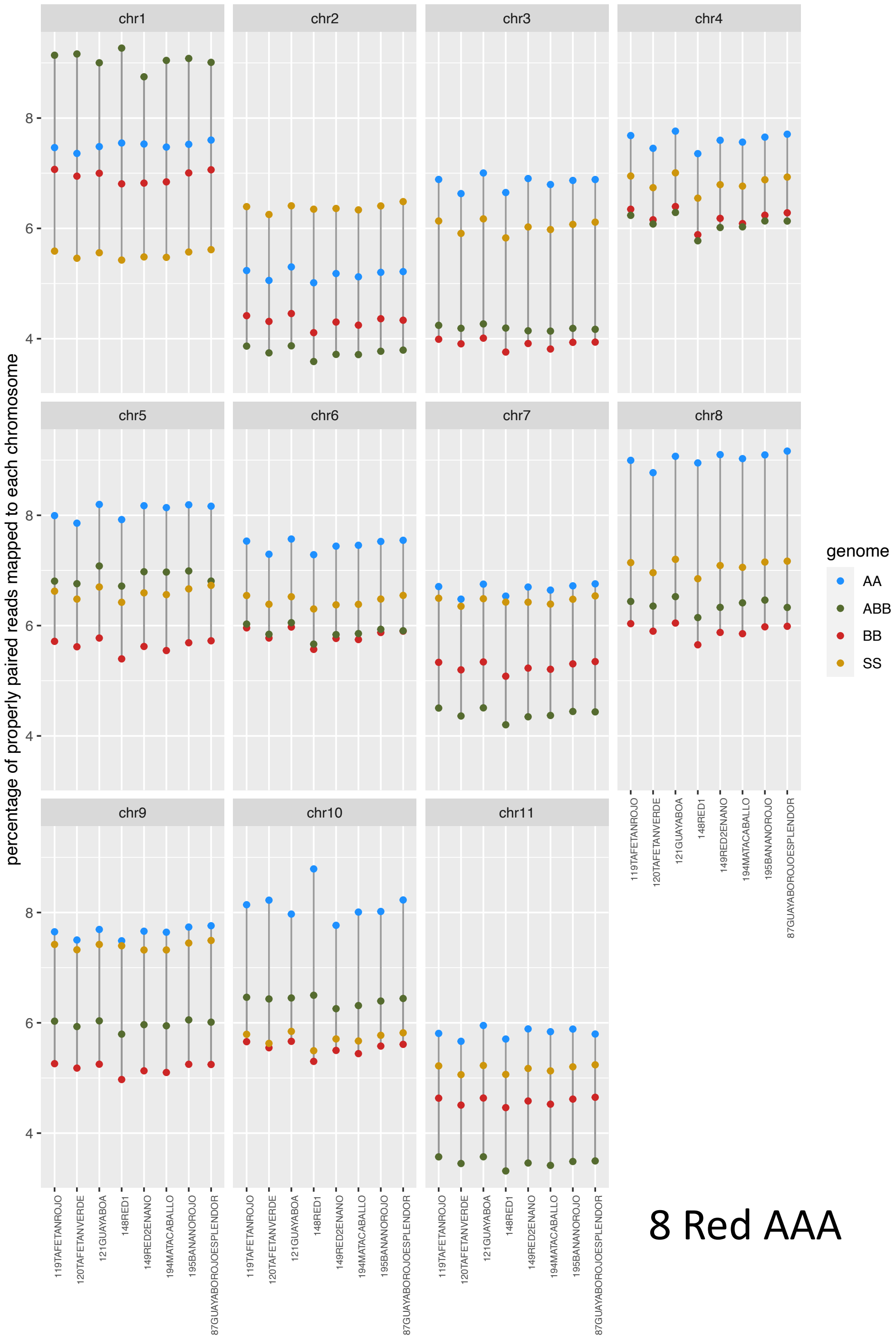

8 Red AAA

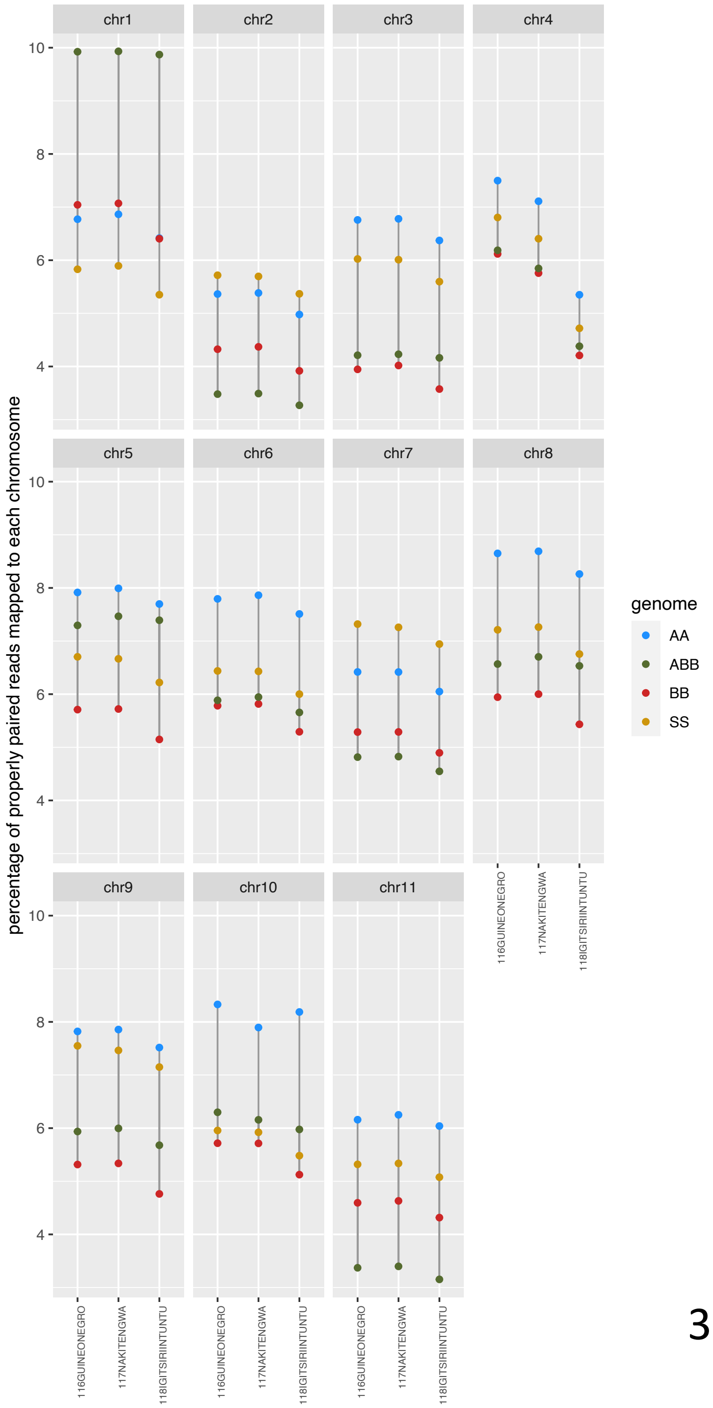

3 Mutika AAA

Additional file 2; Characterising subgenome recombination and chromosomal imbalances in banana varietal lineages.

Janet Higgins, Jaime Andrés Osorio-Guarín, Carolina Olave-Achury, Deisy Lisseth Toloza-Moreno, Ayda Enriquez, Federica Di Palma, Roxana Yockteng, Jose De Vega

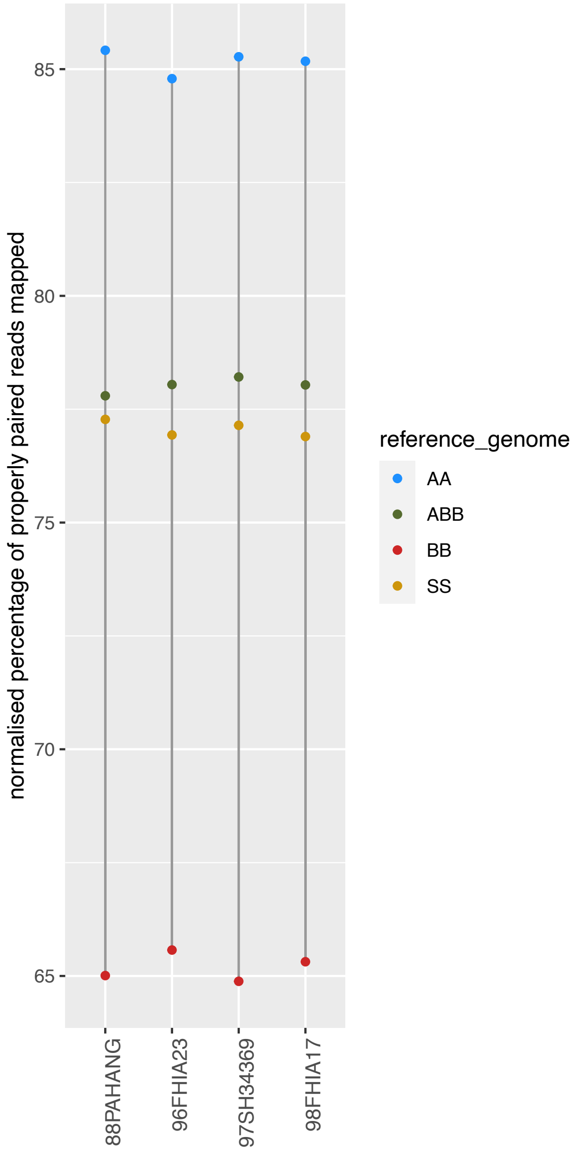

AAAA

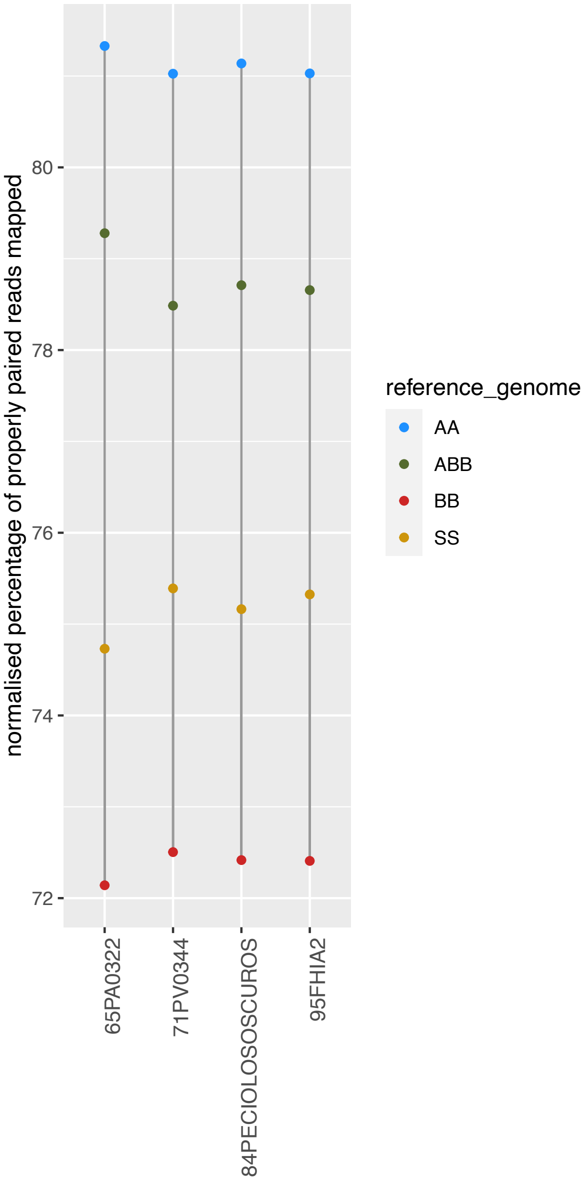

AAAB Pome

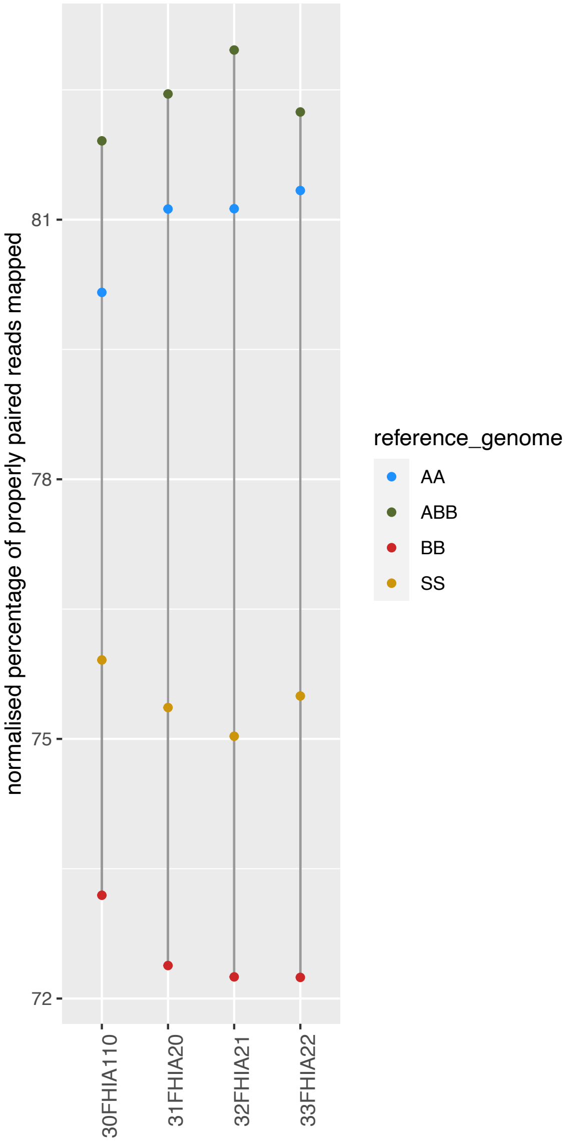

AAAB Africa

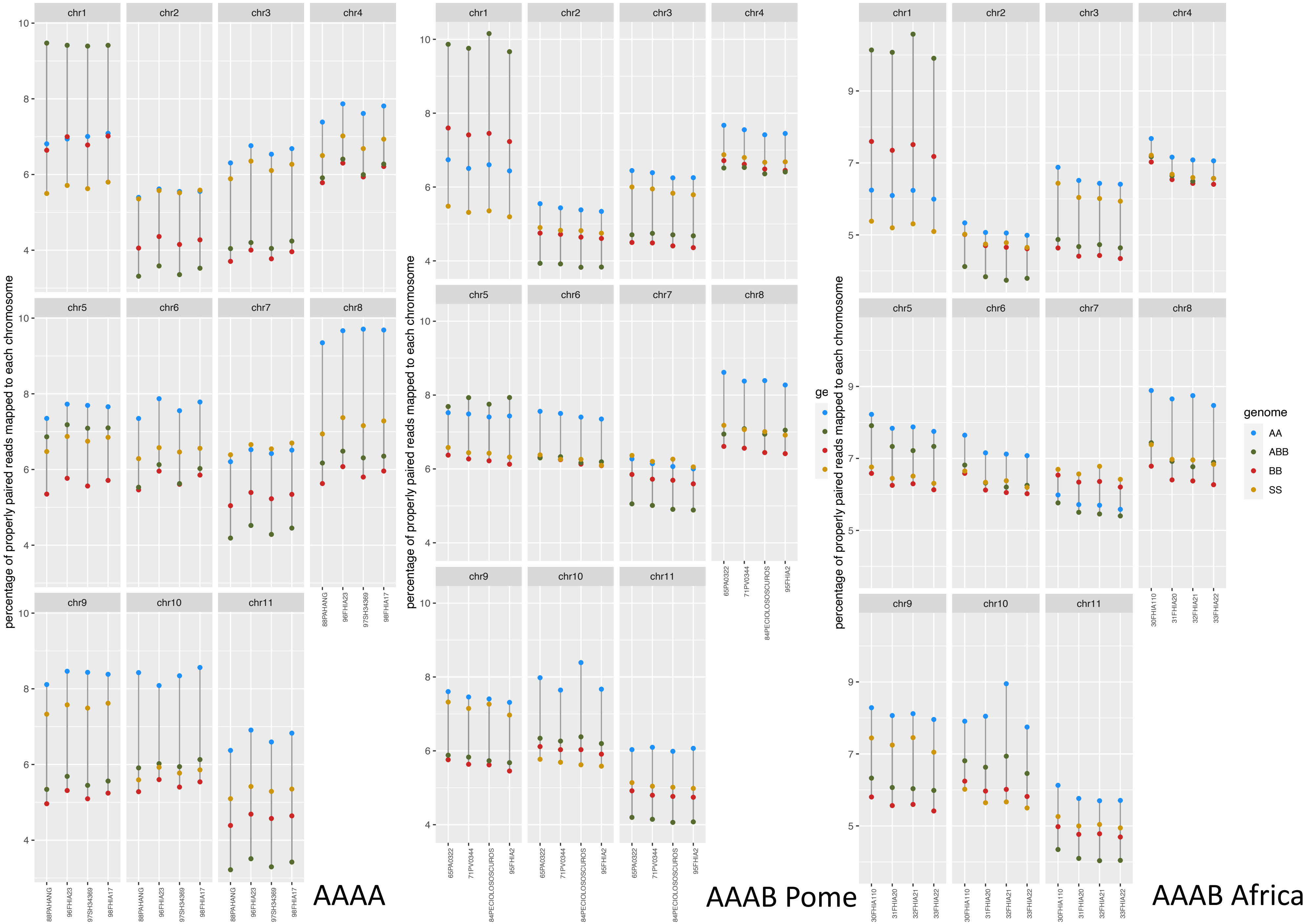

chr1
chr2
chr3
chr4
chr5
chr6
chr7
chr8
chr9
chr10
chr11
percentage of properly paired reads mapped to each chromosome
88PAHANG
96FHIA23
97SH34369
98FHIA17
AAAA
65PA0322
71PV0344
84PECIOLOSOSCUROS
95FHIA2
AAAB Pome
30FHIA110
31FHIA20
32FHIA21
33FHIA22
AAAB Africa
genome
AA
ABB
BB
SS

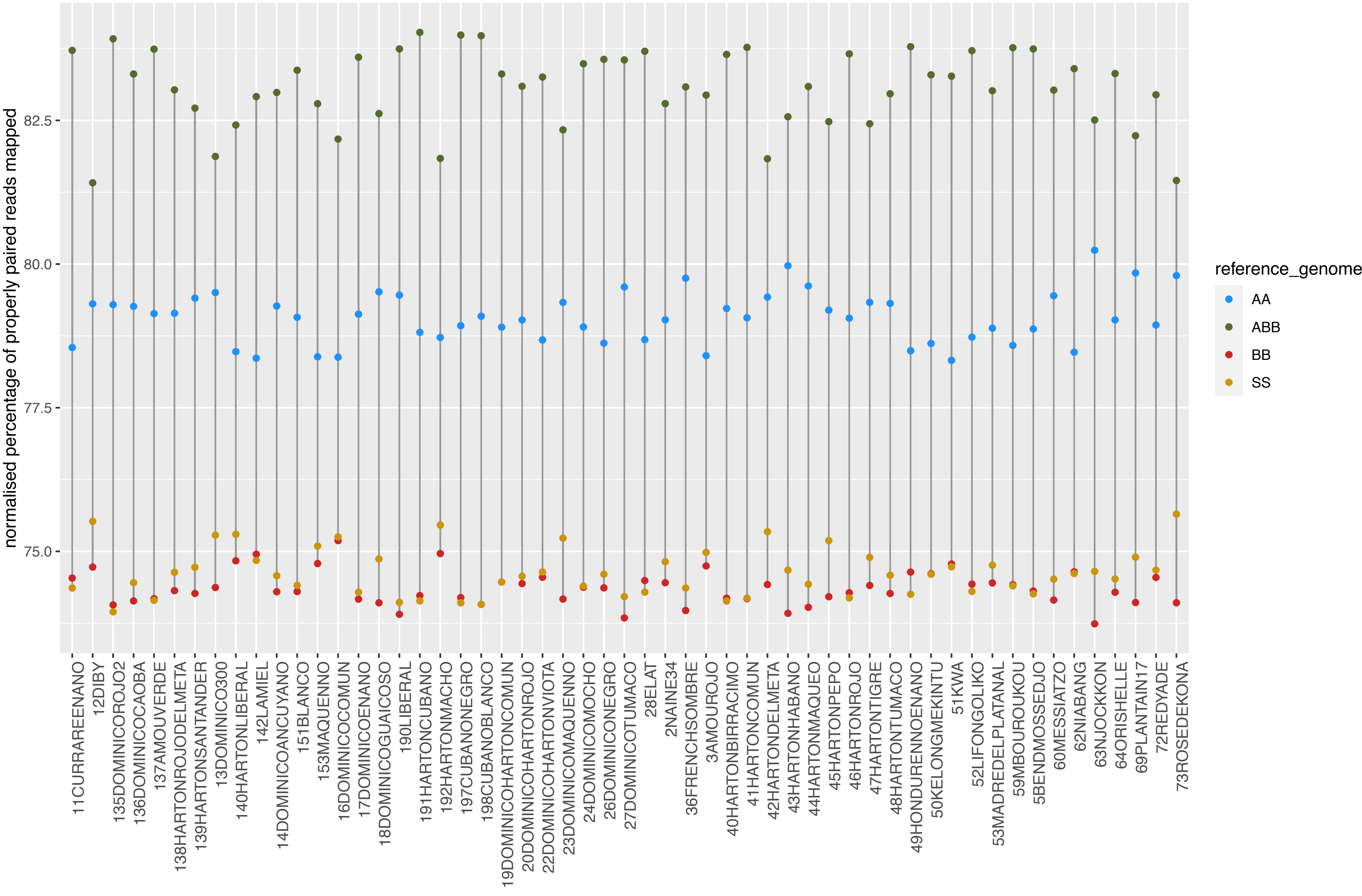

55 Plantain AAB

Additional file 2: Characterising subgenome recombination and chromosomal imbalances in banana varietal lineages

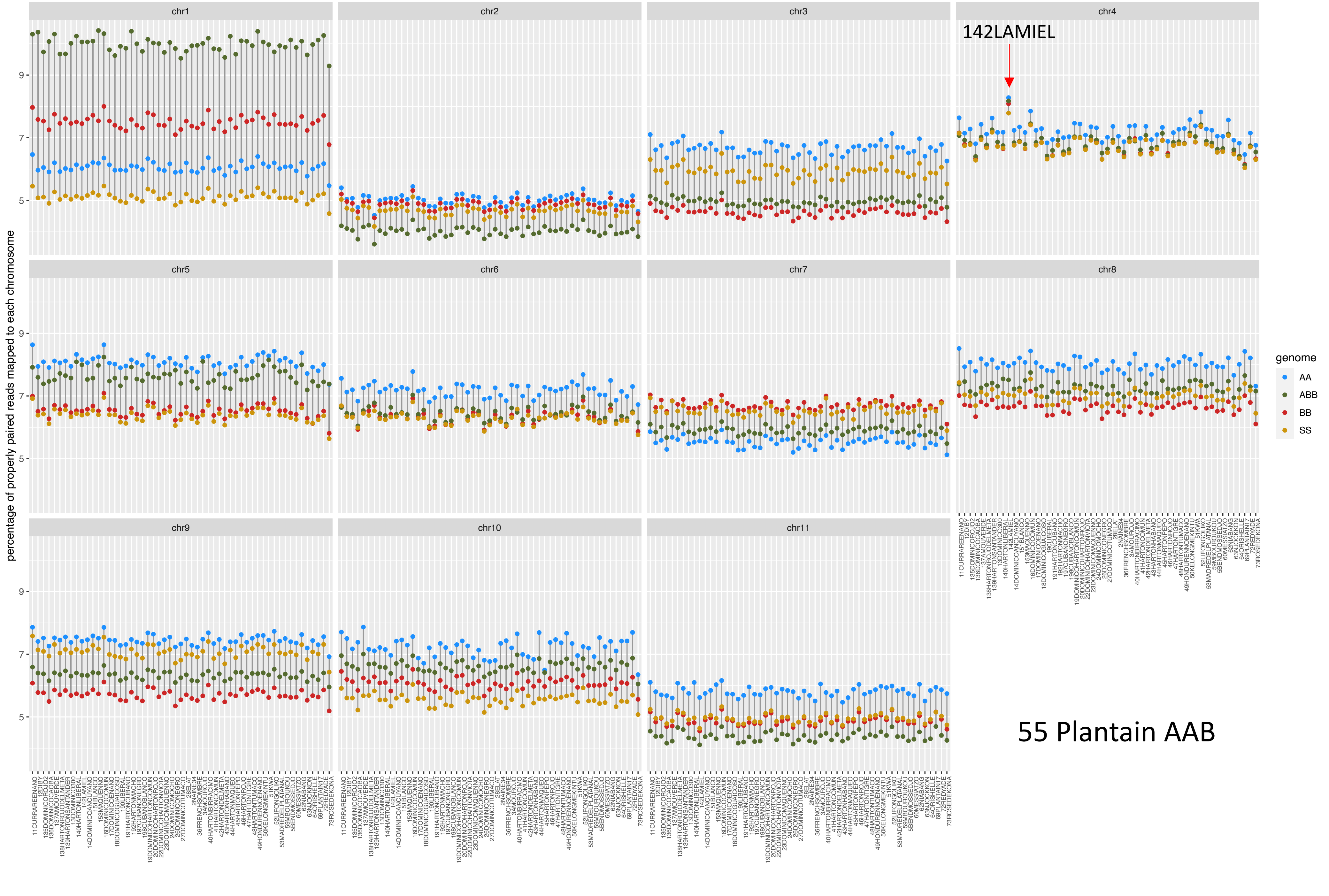

Janet Higgins, Jaime Andrés Osorio-Guarín, Carolina Olave-Achury, Deisy Lisseth Toloza-Moreno, Ayda Enriquez, Federica Di Palma, Roxana Yockteng, Jose De Vega

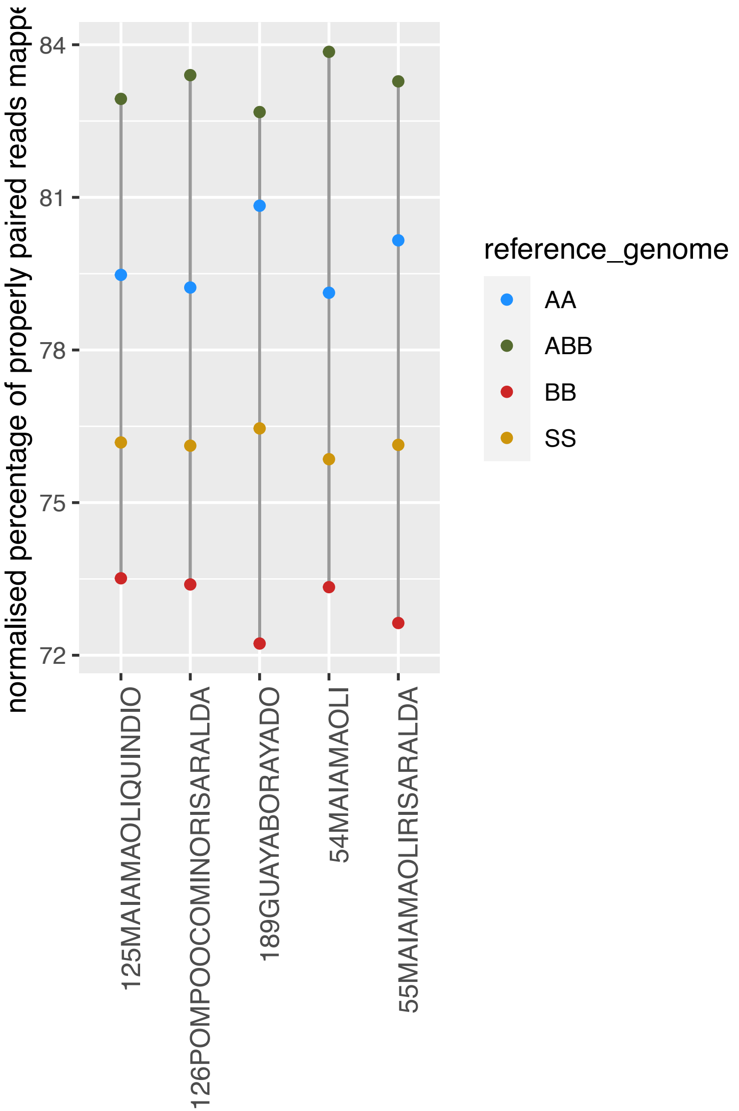

5 Popoulu ABB

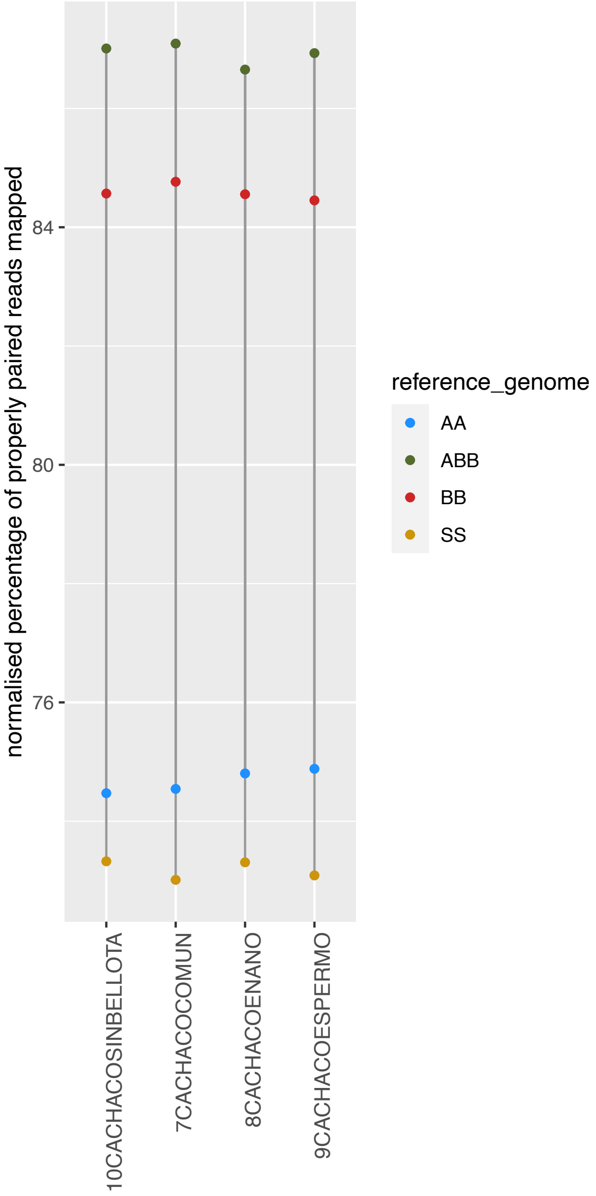

4 Bluggoe ABB

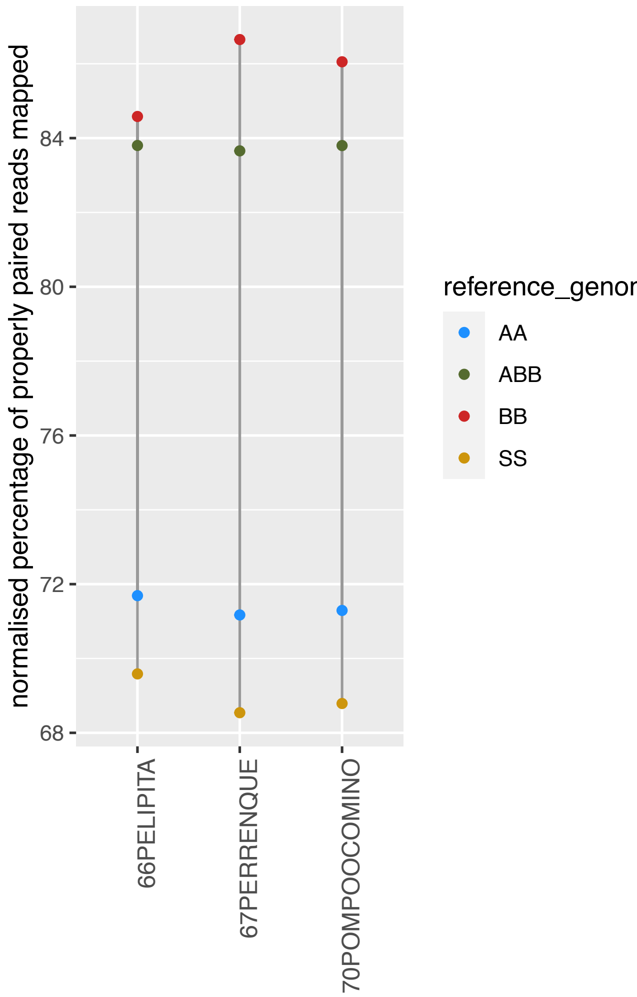

3 Pelipita ABB

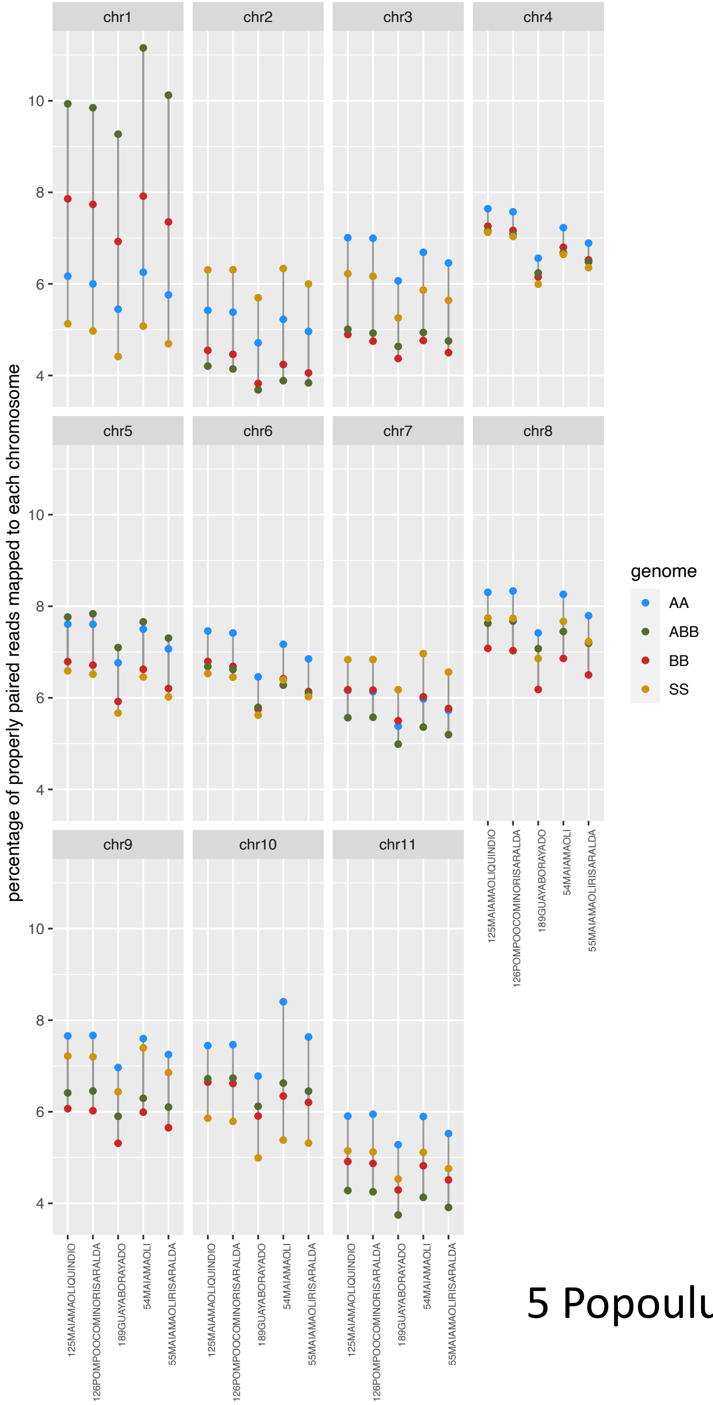

5 Popoulu ABB

mb
ry, D

tal lineages.
Yockteng, Jose De Vega

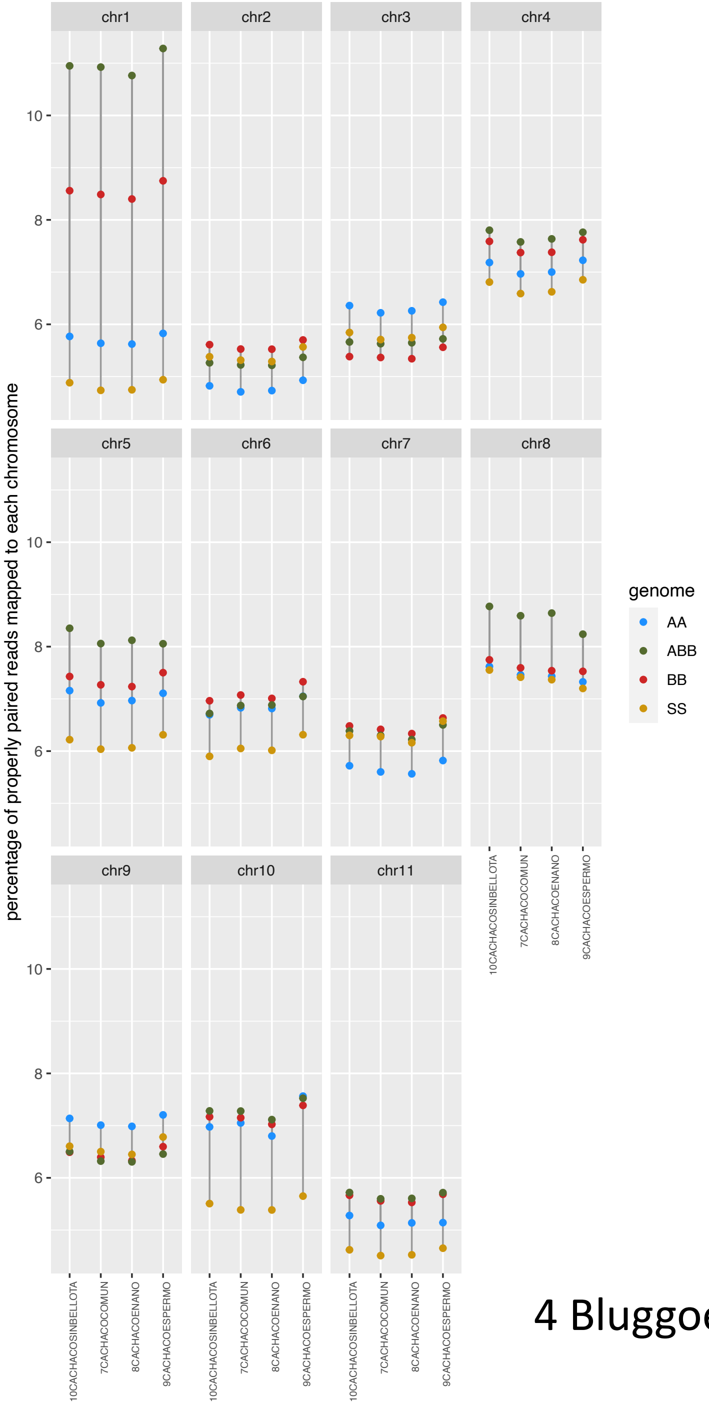

4 Bluggoe ABB

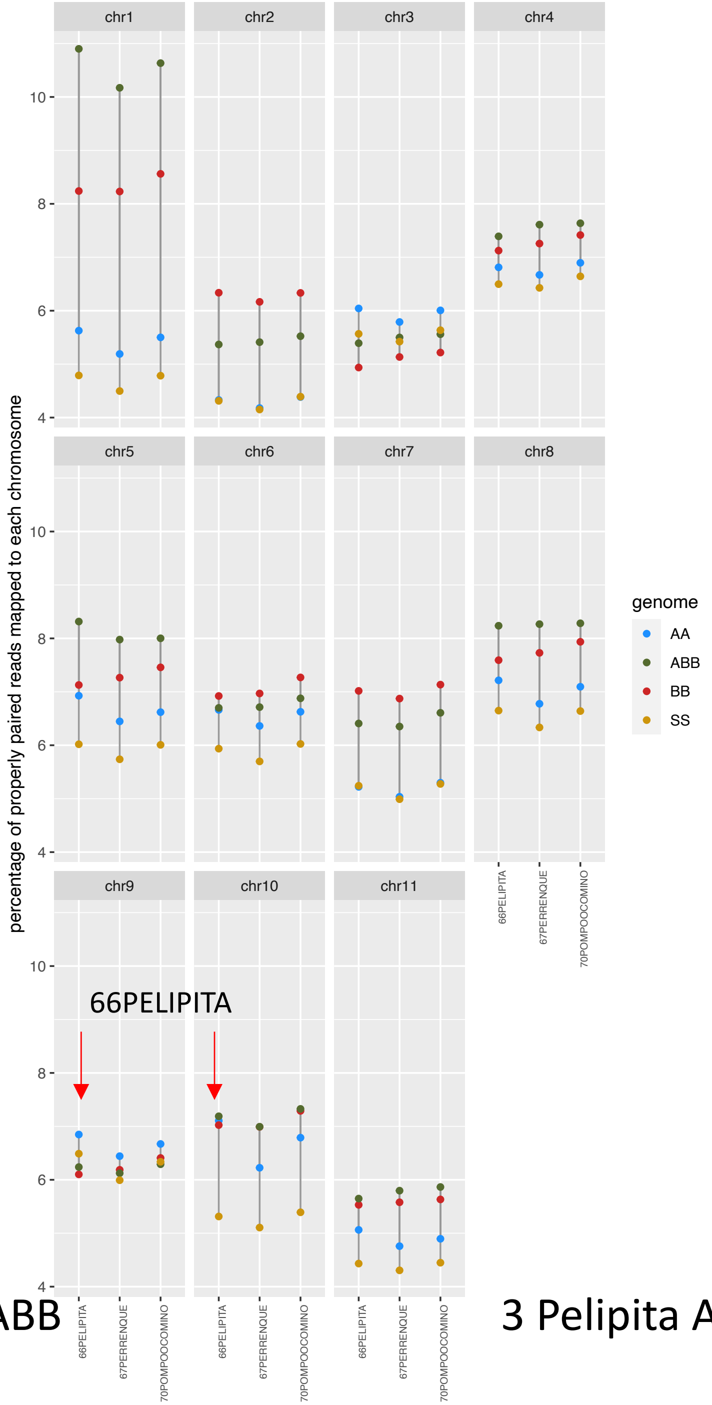

3 Pelipita ABB
