# Supplementary material for: Characterizing subgenome recombination and chromosomal imbalances in banana varietal lineages
Source: Ann Bot. 2023 Dec 14;133(2):349–64. doi: 10.1093/aob/mcad192 (PMC11005773; doi:10.1093/aob/mcad192)
Supplement: mcad192_suppl_Supplementary_File_S3 [file mcad192_suppl_supplementary_file_s3.pdf]

Additional file 3; Characterising subgenome recombination and chromosomal imbalances in banana varietal lineages.

Janet Higgins, Jaime Andrés Osorio-Guarín, Carolina Olave-Achury, Deisy Lisseth Toloza-Moreno, Ayda Enriquez, Federica Di Palma, Roxana Yockteng, Jose De Vega

A Plantain_AAB – 55 accessions

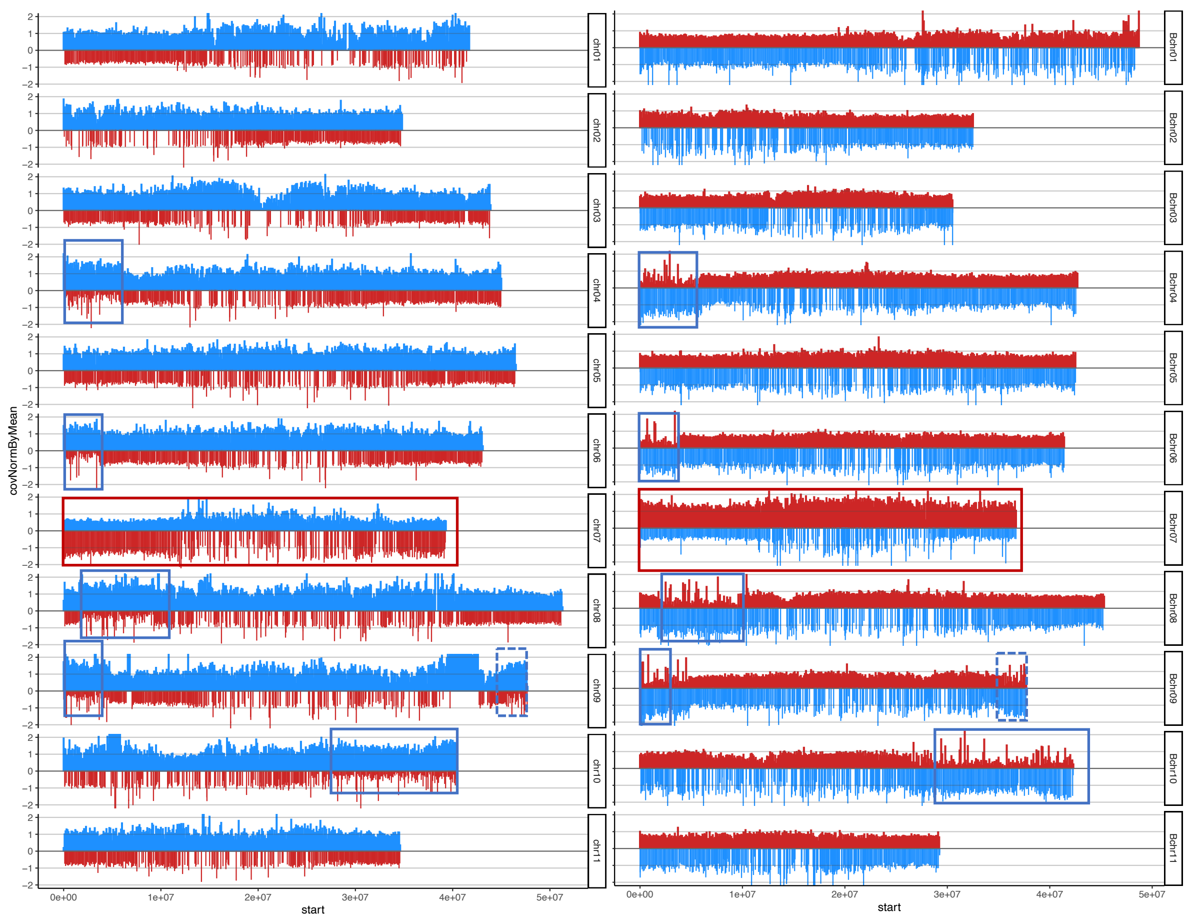

Popoulu_AAB – 5 accessions

B

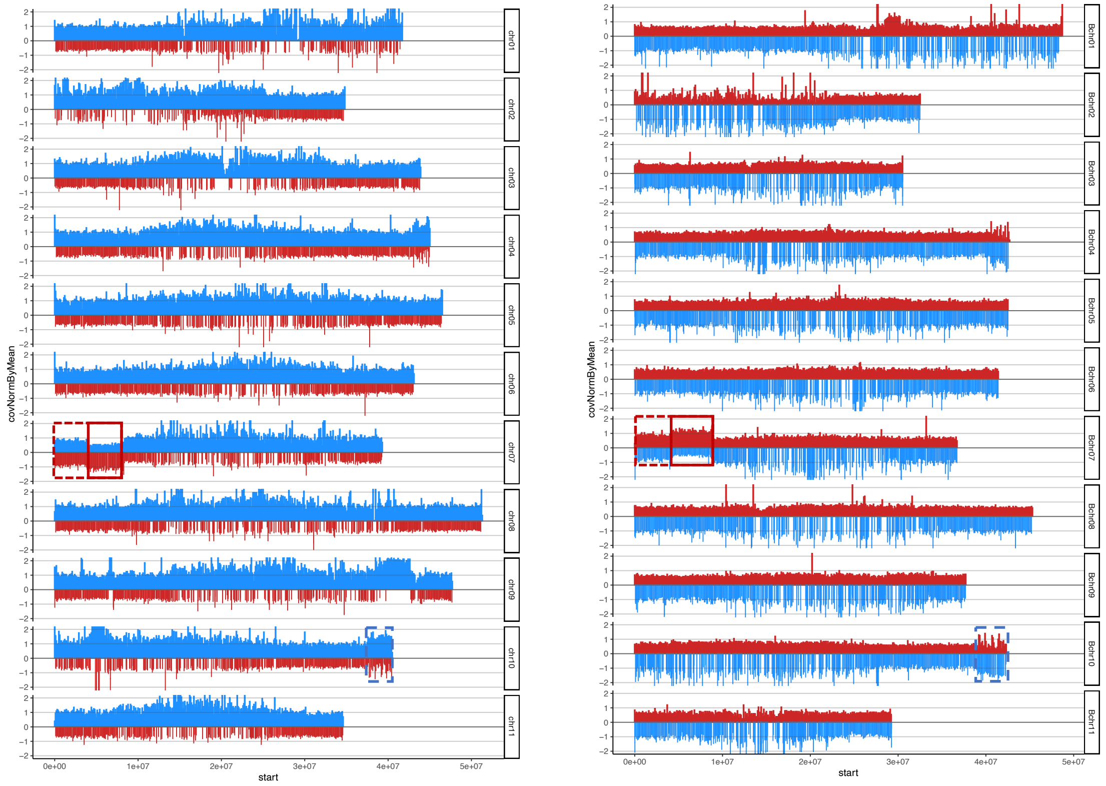

Additional file 3; -cont-

C

Bluggoe_ABB - 4 accessions

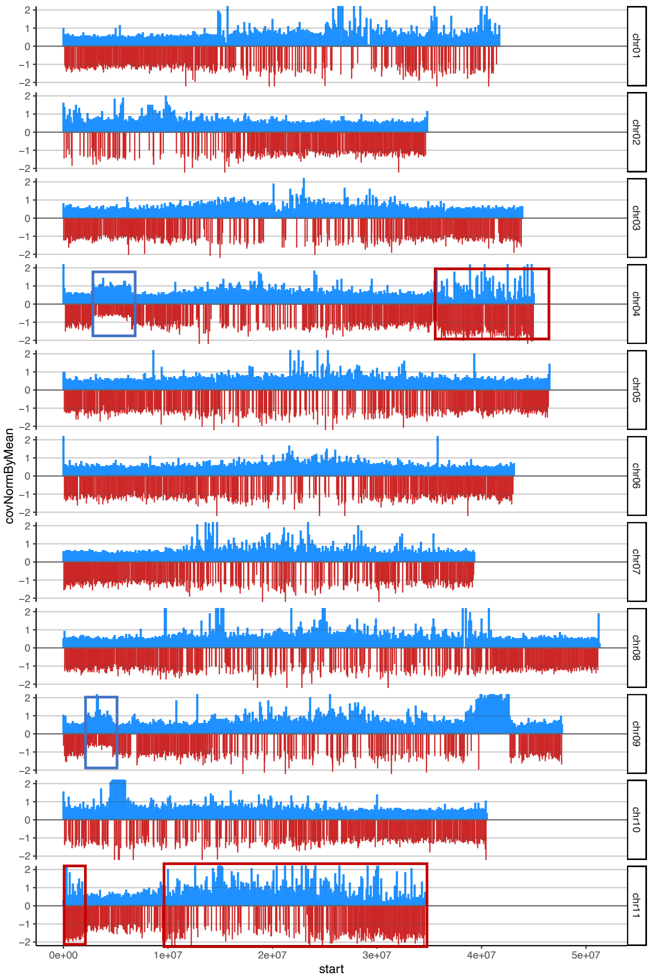

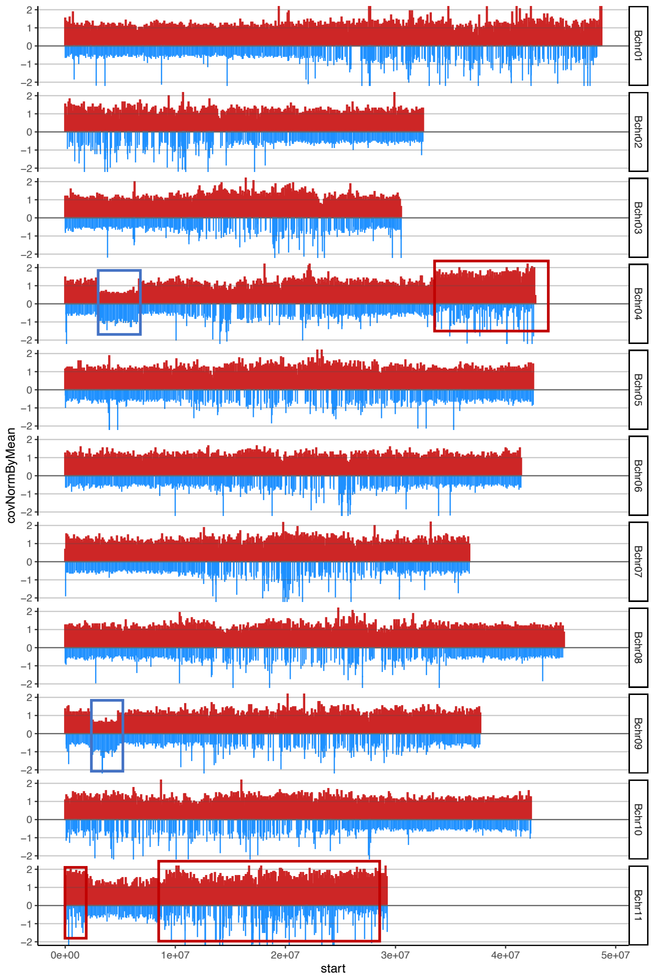

D

Pelipita_ABB - 3 accessions

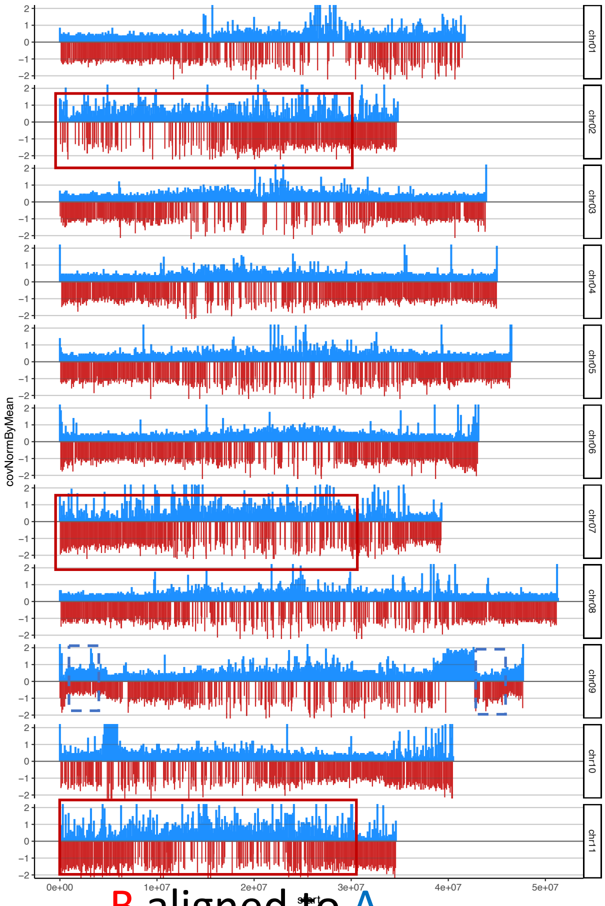

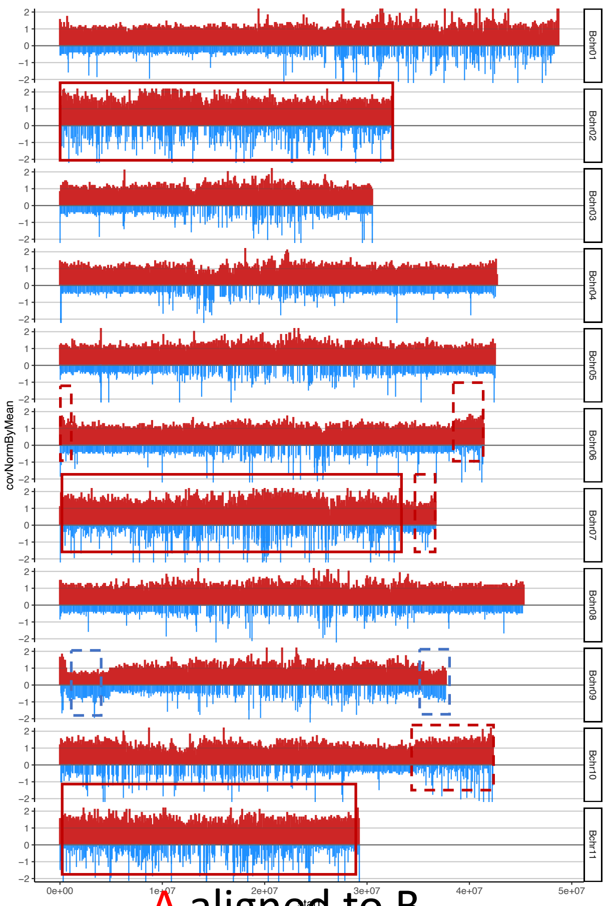

B aligned to A

A aligned to B
