# Supplementary material for: Characterizing subgenome recombination and chromosomal imbalances in banana varietal lineages
Source: Ann Bot. 2023 Dec 14;133(2):349–64. doi: 10.1093/aob/mcad192 (PMC11005773; doi:10.1093/aob/mcad192)

Additional file 6; Higgins. et al. Characterising subgenome recombination and chromosomal imbalances in banana varietal lineages.

Sucrier_AAA

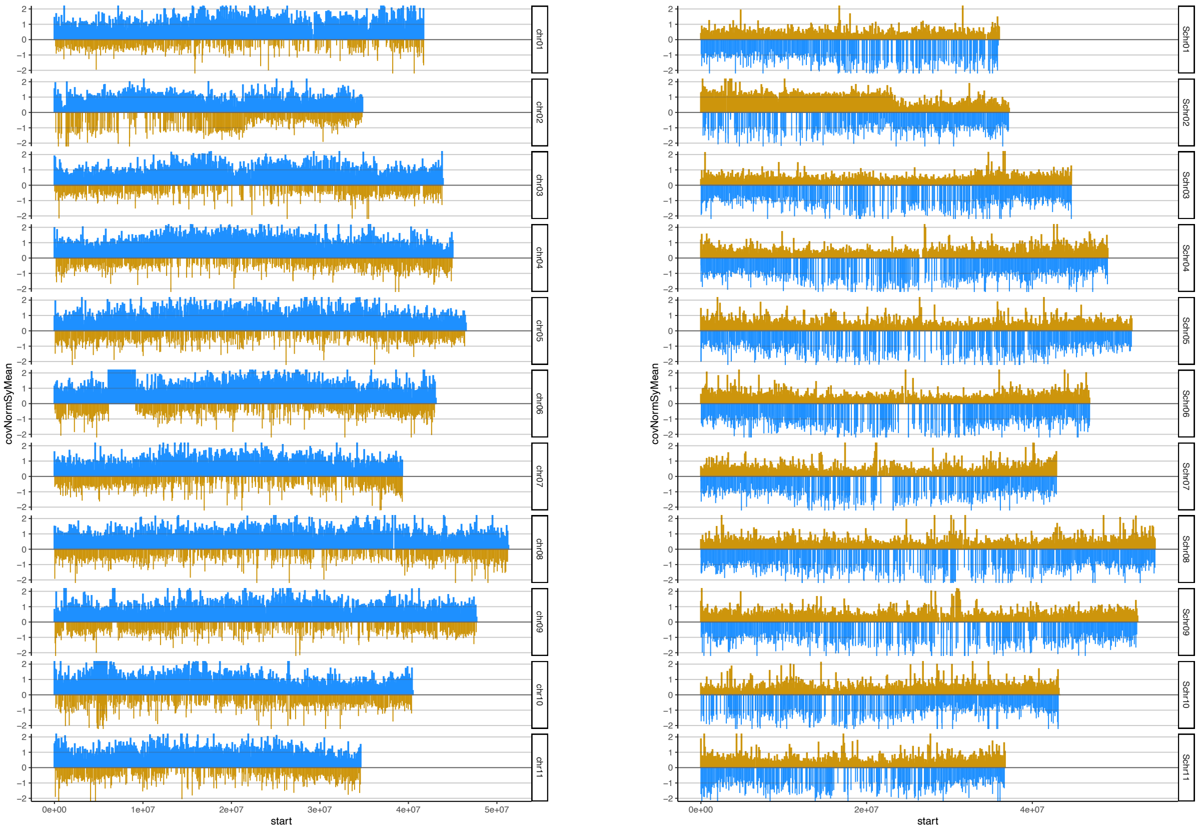

Cavendish_AAA

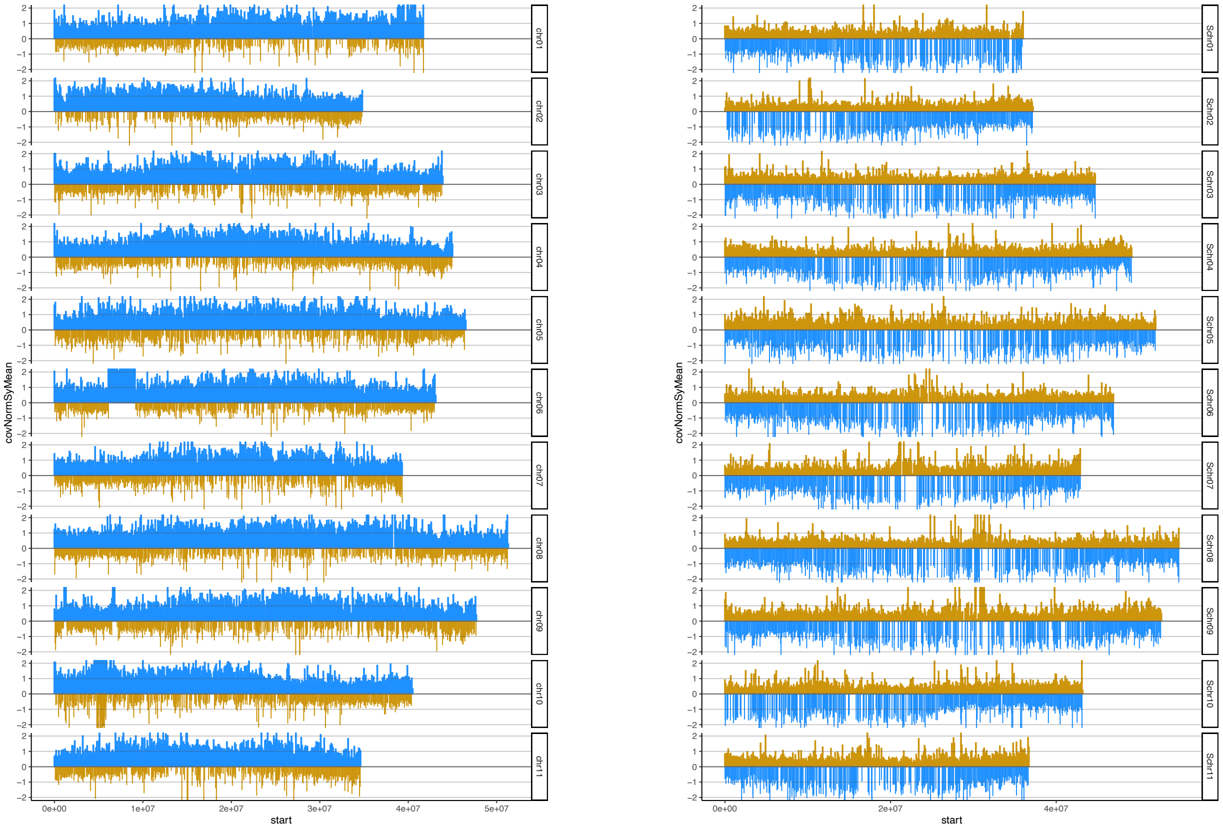

Gros Michel_AAA

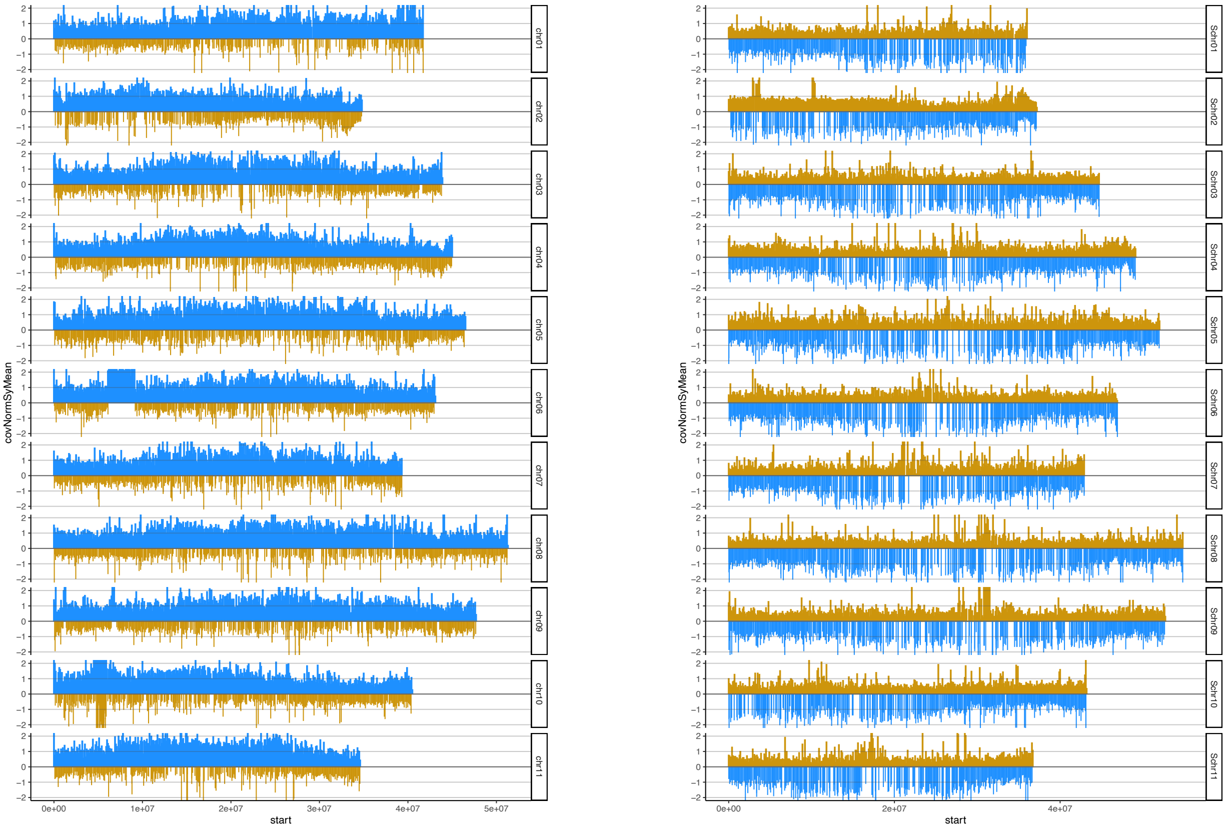

unknown_AAA

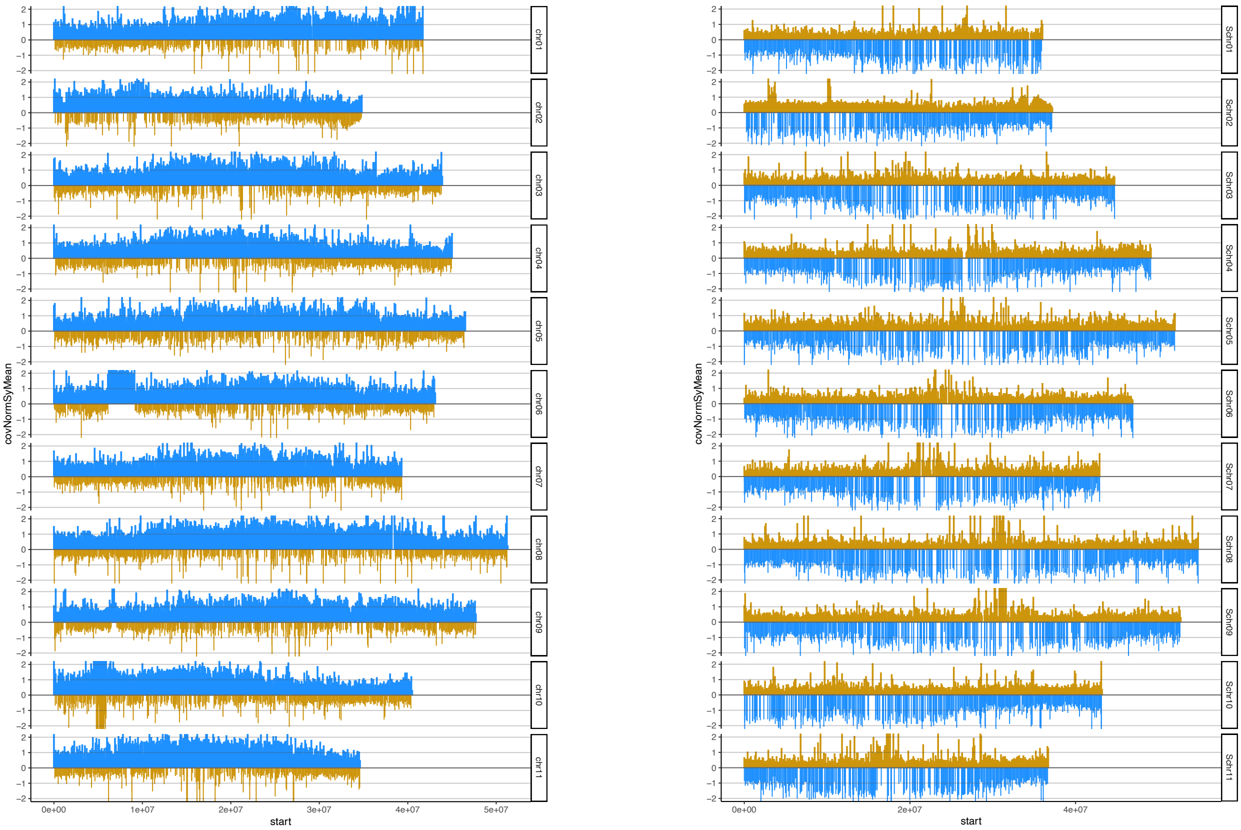

Red_AAA

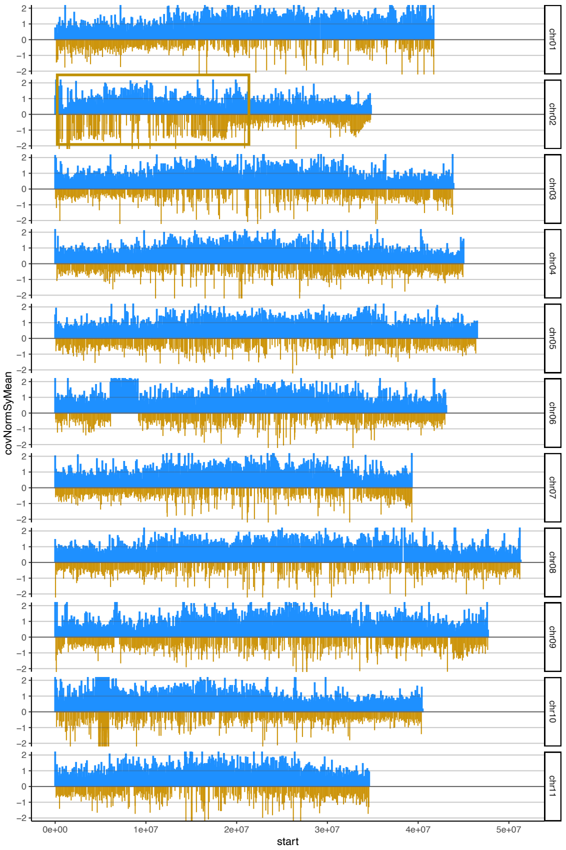

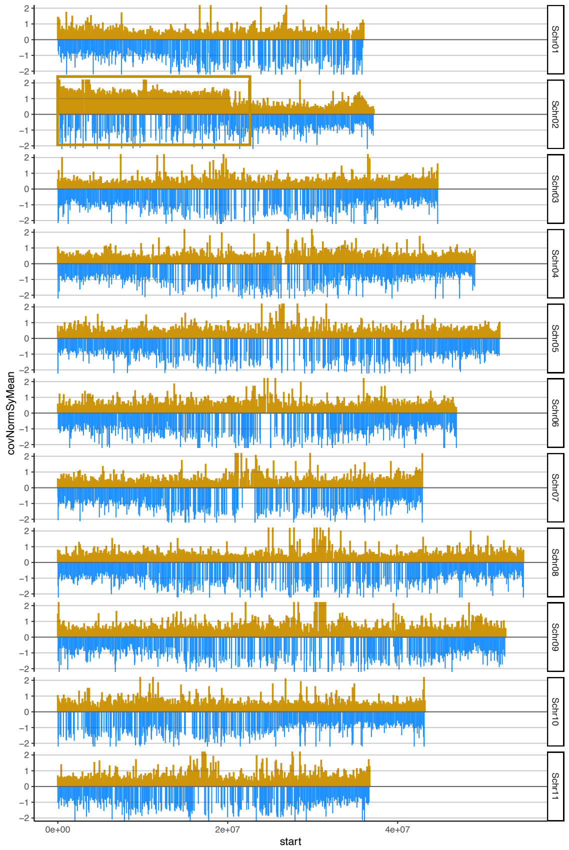

Mutika AAA

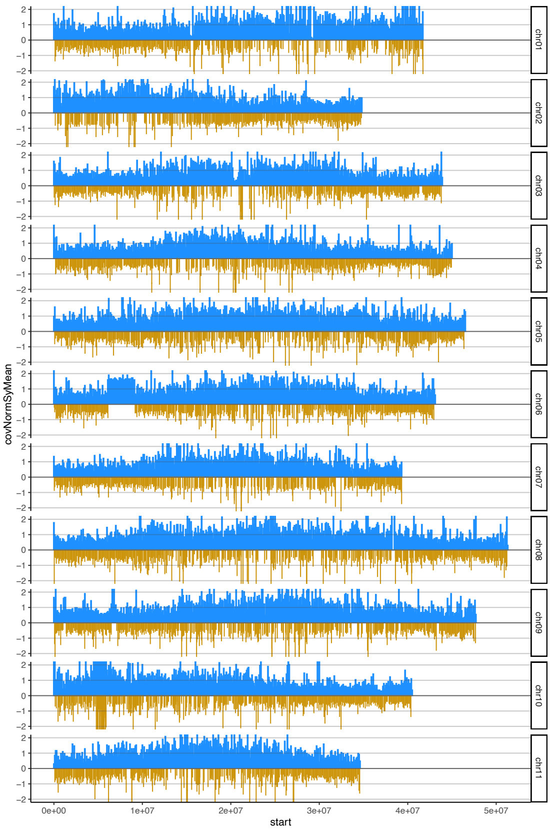

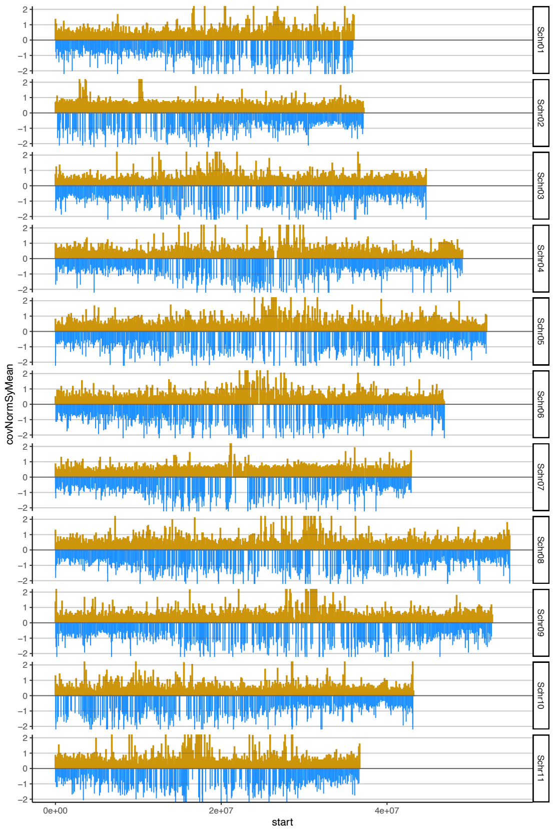

Popoulu AAB

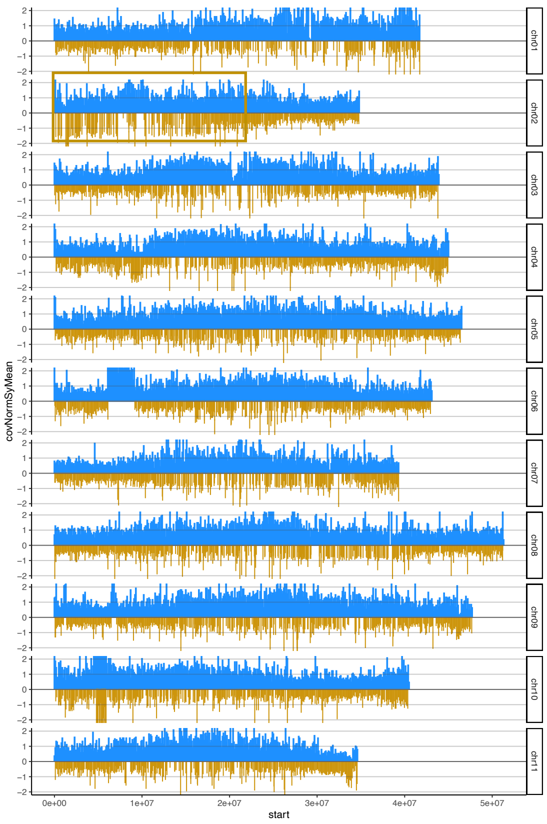

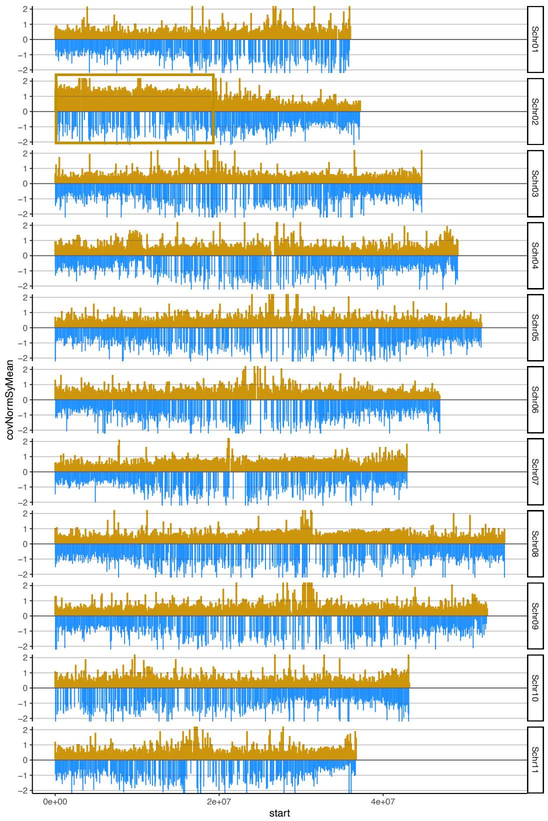

Plantain AAB

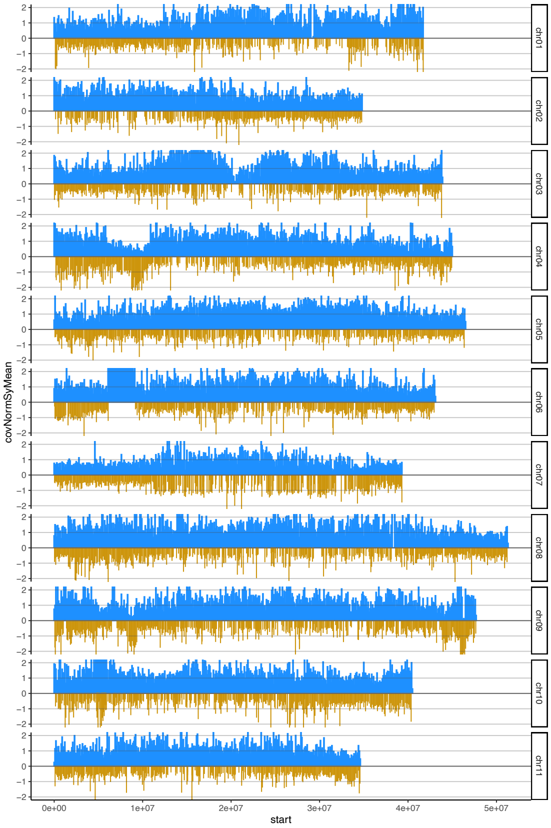

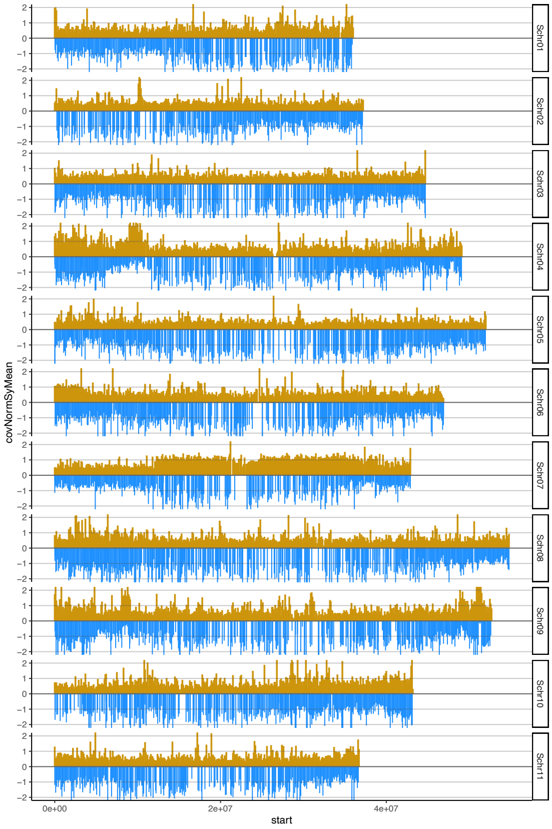

Bluggoe_ABB

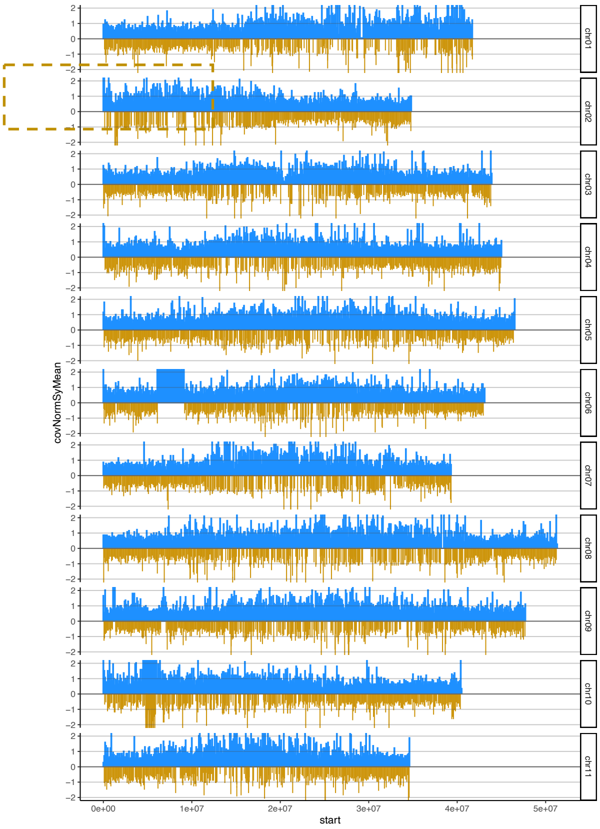

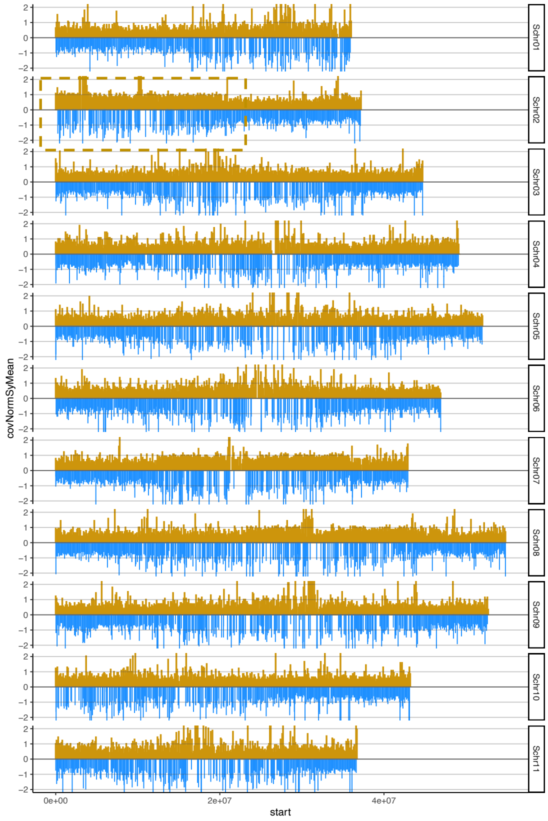

Pelipita_ABB

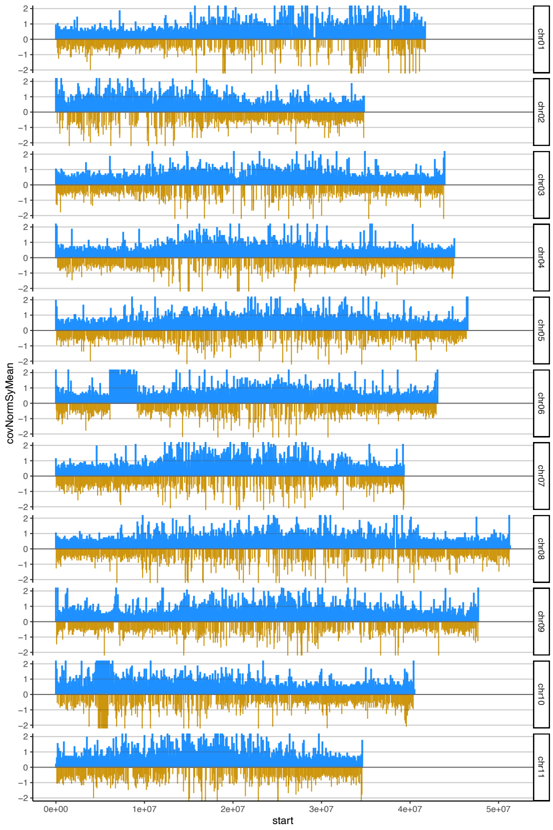

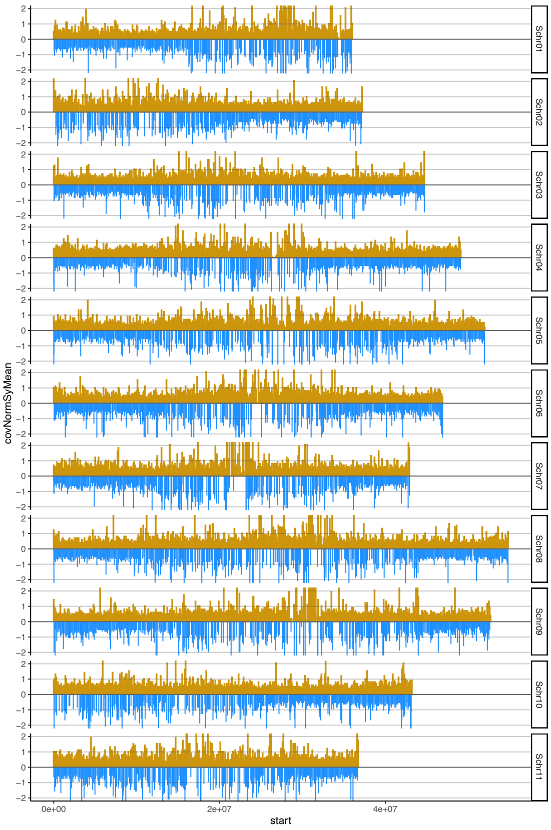

AAAA

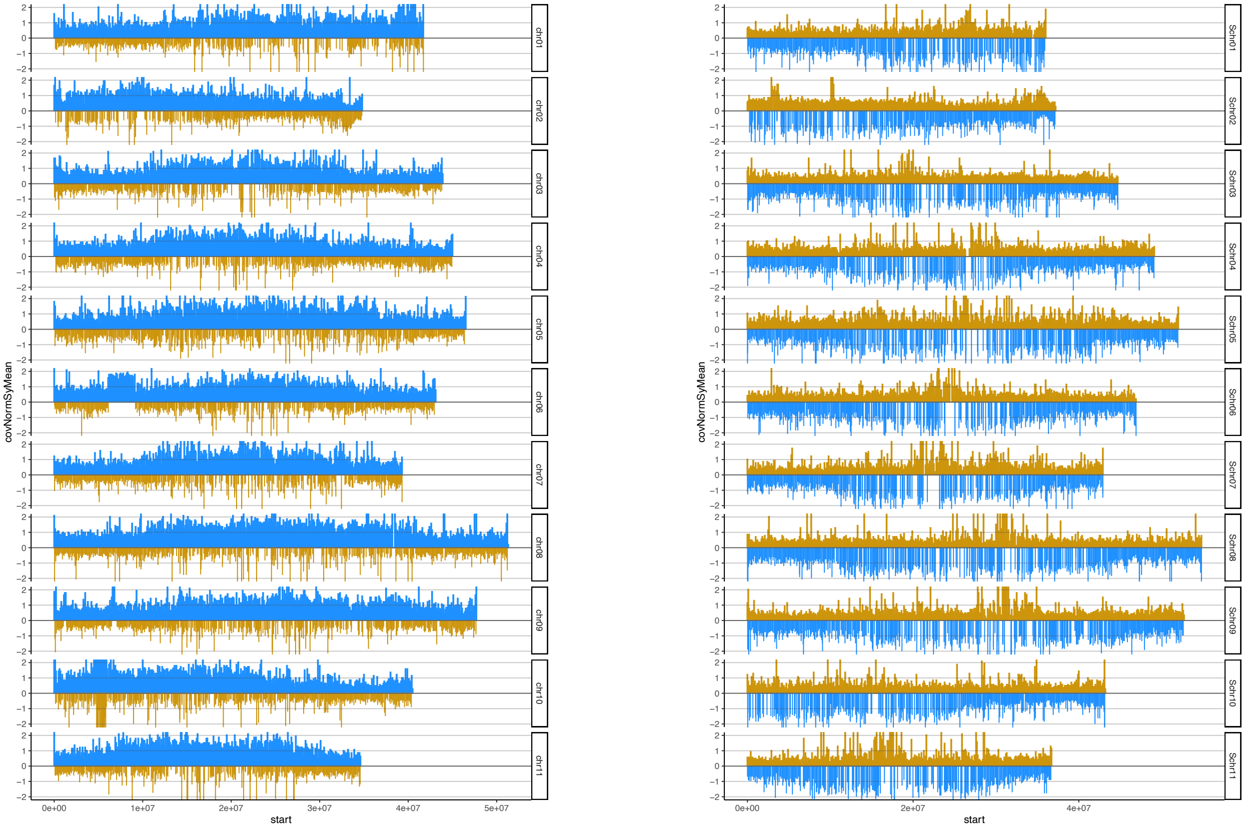
covNormSyMean
chr01
chr02
chr03
chr04
chr05
chr06
chr07
chr08
chr09
chr10
chr11
0e+00
1e+07
2e+07
3e+07
4e+07
5e+07
start
covNormSyMean
Schr01
Schr02
Schr03
Schr04
Schr05
Schr06
Schr07
Schr08
Schr09
Schr10
Schr11
0e+00
2e+07
4e+07
start

AAAB_Pome

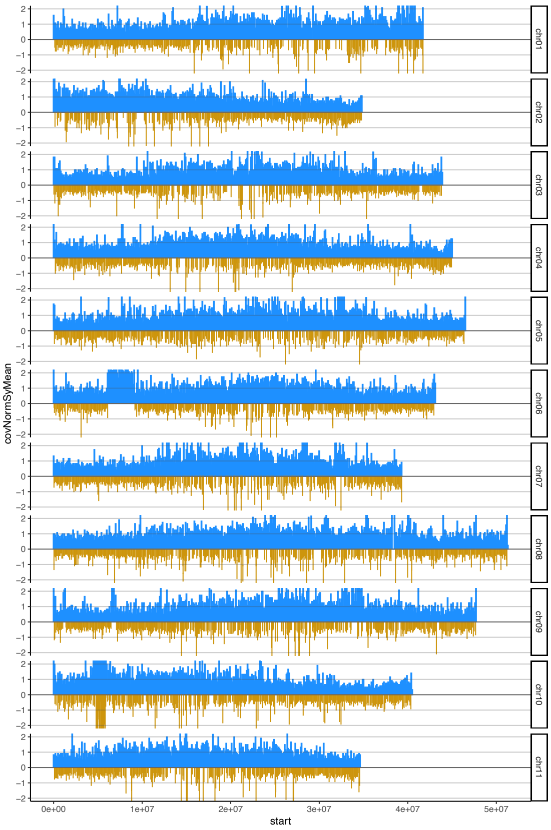

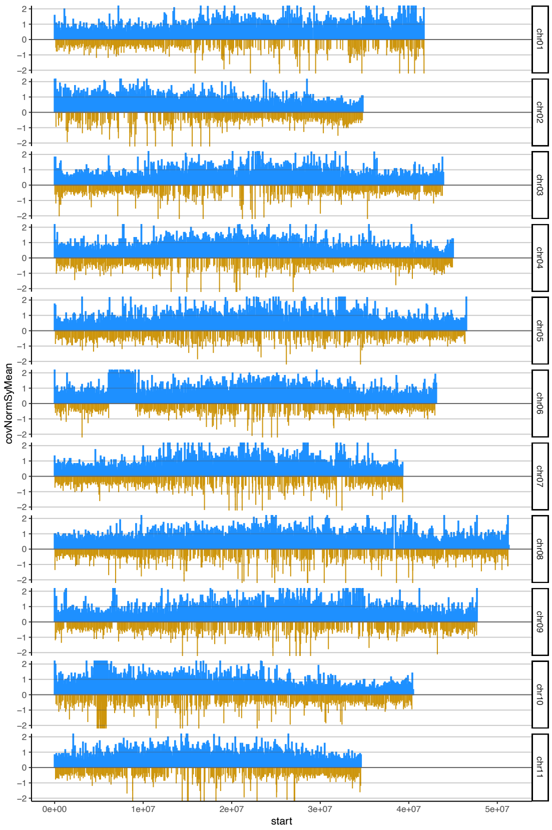

AAAB_Africa

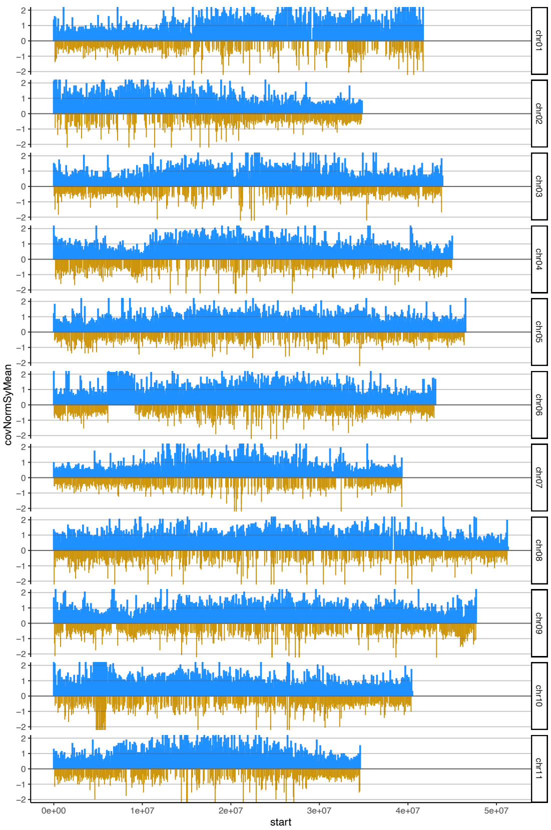

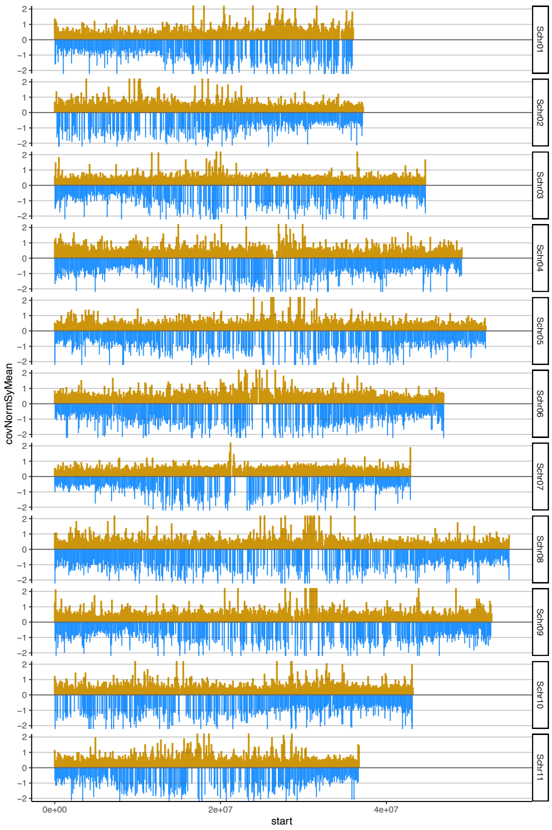

Supplement: mcad192_suppl_Supplementary_File_S6 [file mcad192_suppl_supplementary_file_s6.pdf]
