# Supplementary material for: Characterizing subgenome recombination and chromosomal imbalances in banana varietal lineages
Source: Ann Bot. 2023 Dec 14;133(2):349–64. doi: 10.1093/aob/mcad192 (PMC11005773; doi:10.1093/aob/mcad192)
Supplement: mcad192_suppl_Supplementary_Data_S1 [file mcad192_suppl_supplementary_data_s1.docx]

A Github repository contains the code used in the analysis of this paper:

<https://github.com/jjdevega/Structural-diversity-in-banana-cultivars>

The paper's code is organised into two subfolders:

- Folder Introgression_detection: This subfolder contains the code to obtain and plot the new metrics, called *Relative Averaged Alignment (RAA) and Relative Coverage* starting from BAM alignment files (e.g. from BWA or Bowtie) for each sample.
- Folder Population_structure: SNP calling pipeline (based on BWA, GATK and BCFTOOLS); and bash and R code for Principal Component Analysis, phylogenetic analysis and tree plotting, and bash and R scripts to analyse with STRUCTURE
- **We also include a step by step guideline to calculate and plot RC and RAA in this document.**

## Relative Coverage (RC): Step by step

We used comparisons of read depth, which we called Relative Coverage, to to identify introgressions. It is key to notice that we do not use SNPs. The method builds on the idea that the reads in hybrid, e.g. AAB, need to map twice to the A reference for each one's alignment to the B reference *on average*, and any deviation from this expectation is indicative of introgressions.

- After preprocessing reads to obtain clean trimmed reads (e.g. trim_galore), proceed to align them with BWA-MEM, using the options -M and -k 35, against a synthetic reference genome generated by **CONCATENATING THE REFERENCES FROM THE ANCESTORS** (i.e. one reference Fasta from the concatenation of both ancestors Fasta). Previously, chrs names were renamed to indicate origin, e.g. AA_chr1, BB_chr1, etc.
- awk '/^>/{print ">AA_chr" ++i; next}{print}' A_ancestor.fa > A_renamed.fa
  awk '/^>/{print ">BB_chr" ++i; next}{print}' B_ancestor.fa > B_renamed.fa

  cat A_renamed.fa B_renamed.fa > AB.fa

  bwa index AB.fa AB.idx

  for myID in $(hybrids_list.txt); do
   bwa mem -M -k 35 -R "@RG\tID:${myID}\tSM:${myID}" -t 1 AB.idx \
   <(gzip -dc ${myID}/basic/*_R1_val_1.fq.gz) <(gzip -dc ${myID}/basic/*_R2_val_2.fq.gz) \
   | samtools view -@ 1 -b -S -h - | samtools sort -@ 1 -T ${myID}.tmp -o ${myID}_AB_sort.bam;
  done;
- BAM files were sorted and duplicated reads were removed. Only uniquely mapped reads were retained by excluding reads with the tags 'XA:Z:' and 'SA:Z:', and further filtered to retain only properly mapped paired reads (-f 0x2).
- for file in *.bam; do
   #samtools-1.7
   samtools view -h ${file} | grep -v -e 'XA:Z:' -e 'SA:Z:' | samtools view -h -f 0x2 | samtools view -b > ${file}_unique.bam
  done
- BEDtools genomeCoverageBed with the alignments from each sample (BAM input) to obtain a Bedgraph file for each sample.
- samtools faidx AB.fa
  cat AB.fa.fai | awk '{print $1 "\t" $2}' > AB.chr_lenghts.txt

  for file in *_unique.bam; do
   #bedtools-2.24.0
   genomeCoverageBed -bg -split -ibam ${file} -g AB.chr_lenghts.txt > ${file}.bedgraph
  done;
- BEDtools map to obtain the median of the read coverage or read depth values in the positions within a given 100 Kbp window in the concatenated reference
- bedtools makewindows -g AB.chr_lenghts.txt -w 100000 > AB_windows100kb.bed

  for file in *.bedgraph; do
   #bedtools-2.24.0
   bedtools map -a AB_windows100kb.bed -b ${file} -c 4 -o median > ${file}_median_cov.txt
  done
- All-vs-all every 100 Kbp windows in the A-genome and B-genome were aligned to each other using minimap2 v2.22 (-x asm10) to identify homologous windows for plotting
- #bedtools-2.26.0
  #minimap2-2.22

  bedtools getfasta -fi AA_renamed.fa -bed AB_windows100kb.bed > AA_100kb.fasta
  bedtools getfasta -fi BB_renamed.fa -bed AB_windows100kb.bed > BB_100kb.fasta

  minimap2 -x asm10 AA_100kb.fasta BB_100kb.fasta | sort -k1,1 -k11,11nr | awk '!x[$1]++' > BBquery_AAtarget.paf

  minimap2 -x asm10 BB_100kb.fasta AA_100kb.fasta | sort -k1,1 -k11,11nr | awk '!x[$1]++' > AAquery_BBtarget.paf
- Plot Relative Coverage (RC) using R and ggplot
- library("RColorBrewer")
  library("ggplot2")
  library(dplyr)
  library(tidyr)
  library(stringr)

  #READ THE OUTPUT FROM BEDTOOLS MAP, NORMALISE IT FOR EACH SUBGENOME,
  #AND TRANSFORM ONE SUBGENOME AS NEGATIVE VALUES TO PLOT BELOW THE OTHER
  all<- read.delim ("hybrid1_unique.bam.bedgraph_median_cov.txt", header = FALSE)
  colnames(all) <- c("chrom","start","end","cov")
  all$cov <- as.numeric(all$cov)
  all$cov[is.na(all$cov)] <- 1

  AAchr<- all %>% filter(grepl('AA_chr', chrom)) %>% mutate(chrom = str_replace(chrom,"AA_chr","chr"))>
  AA_chr$covNormByMean <- AA_chr$cov/mean(AA_chr$cov) #normalise by dividing by the total average

  BBchr<- all %>% filter(grepl('BB_chr', chrom)) %>% mutate(chrom = str_replace(chrom,"BB_chr","chr"))>
  BB_chr$covNormByMean <- BB_chr$cov/mean(BB_chr$cov) #normalise by dividing by the total average
  make.negative <- function(x) -1*abs(x) #make all BB negative for plotting
  BBchr.negative <- cbind(BBchr,"cov_neg" = make.negative(BB_chr$covNormByMean))

  #READ THE OUTPUT FROM MINIMAP
  minimap <- read.delim("BB_100kbwindows-over-AAv4.longest_alignmentotal.paf", header=F)
  minimap <- minimap[,c(1,6,8,9)]
  colnames(minimap) <- c("uniqID","inAA_chr","inAA_start","inAA_stop")


  #join left both data frames
  #create UNIQid field needed for merge() in bed format, as chr:startbp-stopbp
  BBchr.negative$uniqID <- paste0(BBchr.negative$chrom,":",BBchr.negative$start,"-",BBchr.negative$end)
  BBchr.negative.join <- merge(x=BBchr.negative, y=minimap, by="uniqID", all.x = FALSE) #false to ignore BBs without AA homologous -join left-

  #PLOT
  AA_chr$facet <- Achr01$chrom
  BBchr.negative.join$facet <- BBchr.negative.join$inAA_chr

  ggplot() + geom_bar(data=Achr01,aes(x=start,y=covNormByMean), stat = "identity",color="dodgerblue") +
  geom_bar(data=Bchr01negJOIN,aes(x=inAA_start,y=cov_neg), stat = "identity",color="firebrick3") +
  coord_cartesian(ylim = c(-2,3)) + theme(axis.text = element_text(size = 5)) +
  geom_hline(yintercept=0, linetype="solid", color = "gray40") +
  geom_hline(yintercept=c(0.5,1,1.5,2), linetype="solid", color = "gray30", alpha=0.3) +
  geom_hline(yintercept=c(-0.5,-1,-1.5,-2), linetype="solid", color = "gray30", alpha=0.3) +
  facet_wrap(~facet,ncol=1,strip.position = "right") +
  theme_classic()

NOTE: When the sequence is equal between the two ancestral genomes (e.g. no sequence divergence between A and B ancestors), some reads mapping over the conserved sequences can be assigned to the incorrect donor, so generating a background signal. On average, over a 100Kb window, there is plenty of variation between the references to distinguish background noise from the proportion of mapping reads evaluated, so it does not affect the method significantly.

## Relative averaged alignment (RAA): Step by step

We established a new method, called RAA, by quantifying the normalised relative alignment from each accession to three reference banana genomes, which are representative of the A, B and S genome donors. We called this normalised alignment metric “Relative averaged alignment” (RAA). The RAA accounts for the technical variation between samples and reference bias, ie. the phylogenetic distance between a variety and a genome reference.

- Preprocessing reads to obtain clean trimmed reads (e.g. trim_galore), then the processed reads were aligned using BWA MEM v0.7.17 (Li, 2013), with the options -M and -R to define read-groups against each genome reference.
- #bwa-0.7.17, samtools-1.7

  for reference in $(cat ref_fasta_list.txt); do
   bwa index reference reference.idx
   for myID in $(hybrids_list.txt); do
   bwa mem -M -R "@RG\tID:${myID}\tSM:${myID}" -t 1 reference.idx ${myID}_1.fq.gz ${myID}_2.fq.gz | \
   samtools view -@ 1 -b -S -h - | samtools sort -@ 1 -T ${myID}.tmp -o ${myID}_${reference}.bam;
   done
  done
- Obtain alignment statistics using Samtools flagstat v1.7 (Li et al. 2009) for the complete genome or separately for each of the 11 chromosomes in each reference.
- #multiple ${myID}_${reference}.bam files
  for file in *bam; do
   samtools flagstat ${file} > ${file}.stats.txt
  done
- For each statistics file extract the name and percentage of properly paired read pairs
- for i in *bam.stats.txt; do
   echo ${i/.bam.stats.txt} $(cat ${i} | grep 'properly paired' | tr '(' '\t' | tr '%' '\t' | cut -f2)
  done
- Let's plot it, but before we will normalise percentage of mapped read pairs to account for the genetic distance of different subpopulations to the reference. For that, the percentage of properly mapped read-pairs is normalised by the average from the ratios in the same genetic cluster and references.
  - The “relative averaged alignment” (RAA) is a normalised percentage of properly paired reads in a sample and reference that accounts for variation in sample quality (PCR duplications, DNA quality, etc) and differences in the genetic distance between varieties and the reference (reference bias). RAA was calculated by dividing the percentage of properly paired reads from a sample in a reference by a weight factor. The weight factor was obtained by averaging the ratios in each reference genome between the properly paired reads in the sample and variety cluster. RAA per chromosome was similarly calculated except for each chromosome's alignment statistics instead of the total genome.
- #R
  AA <- read.csv("AA_align_stats.csv")
  BB <- read.csv("BB_align_stats.csv")
  SS <- read.csv("SS_align_stats.csv")
  tog <- bind_cols(ID=AA$sample, "AA" = AA$pp_mapped, "BB" = BB$pp_mapped, "SS" = SS$pp_mapped, group=AA$genetic_group)

  cavendish <- subset(tog,tog$group == "Cavendish")

  #1/NORMALISE
  #calculate the weight to normalise with by firstly normalising by referece=by column within the genomic group, then calculating the average of the references/columns
  cavendish$normAA <- cavendish$AA/mean(cavendish$AA)
  cavendish$normBB<- cavendish$BB/mean(cavendish$BB)
  cavendish$normSS <- cavendish$SS/mean(cavendish$SS)
  cavendish <- cavendish %>% mutate(nMean = rowMeans(select(., starts_with("norm"))))

  #normalise original sample values by the nMean for each sample/row
  cavendish$AAn <- cavendish$AA/cavendish$nMean
  cavendish$BBn <- cavendish$BB/cavendish$nMean
  cavendish$SSn <- cavendish$SS/cavendish$nMean

  #2/PLOT
  cav <- cavendish %>% select(ID,genetic_group,AAn,BBn,SSn)
  cav <- melt(cav)
  colnames(cav) <- c("ID","group","reference_genome","pp")

  ggplot(cav,aes(ID,pp)) +
  geom_line(col = "grey60") +
  geom_point(aes(colour=reference_genome)) +
  theme(axis.text.x=element_text(angle = 90, hjust = 1, size=10)) +
  xlab("") +
  ylab("normalised percentage of properly paired reads mapped") +
  scale_color_manual(values=c("dodgerblue","darkolivegreen","firebrick3","darkgoldenrod3"))
